# Network-Based Drug Optimization toward the Treatment of Parkinson’s Disease: NRF2, MAO-B, Oxidative Stress, and Chronic Neuroinflammation

**DOI:** 10.1021/acs.jmedchem.4c02659

**Published:** 2025-01-17

**Authors:** Pablo Duarte, Francisco J. Sanchez-Porro, Enrique Crisman, Ángel Cores, Irene Jiménez, Antonio Cuadrado, J. Carlos Menéndez, Rafael León

**Affiliations:** † Consejo Superior de Investigaciones Científicas (IQM-CSIC), Instituto de Química Médica, 28006 Madrid, Spain; ‡ Fundación Teófilo Hernando para la I+D del Medicamento, Las Rozas, 28290 Madrid, Spain; § Unidad de Química Orgánica y Farmacéutica, Departamento de Química en Ciencias Farmacéuticas, Facultad de Farmacia, Universidad Complutense, 28040 Madrid, Spain; ∥ Instituto de Investigación Sanitaria La Paz (IdiPaz) and Departamento de Bioquímica, Facultad de Medicina, UAM, Instituto de Investigaciones Biomédicas “Alberto Sols” UAM-CSIC, 28029 Madrid, Spain; ⊥ Centro de Investigación Biomédica en Red Sobre Enfermedades Neurodegenerativas (CIBERNED), ISCIII, 28029 Madrid, Spain

## Abstract

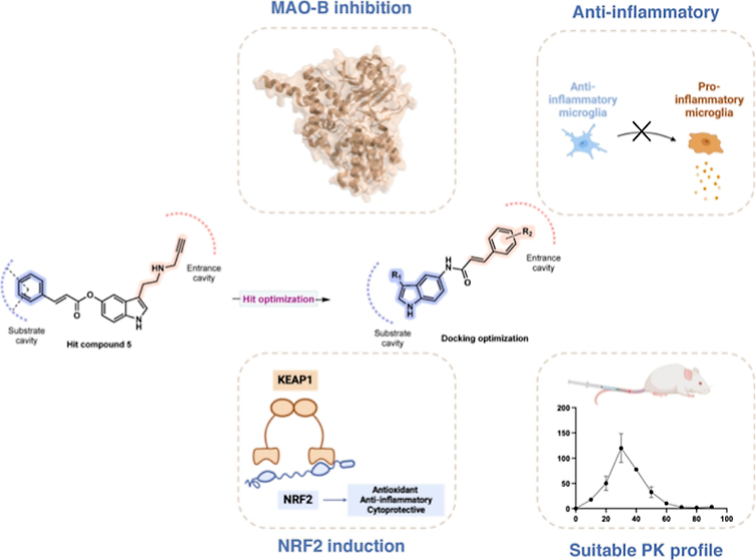

Parkinson’s disease (PD), the second most common neurodegenerative disorder, affects around 10 million people worldwide. It is a multifactorial disease marked by dopaminergic neuron loss with oxidative stress (OS) and neuroinflammation as key pathological drivers. Current treatments focus on dopamine replacement and are symptomatic, underscoring the urgent need for disease-modifying therapies. Here, we present a novel class of dual MAO-B inhibitors and NRF2 inducers with neuroprotective properties in in vitro PD models. Through an optimization program, we enhanced their MAO-B inhibitory potency, selectivity, and NRF2 induction capacity while achieving favorable pharmacokinetic profiles. Virtual library screening identified two core derivatives, leading to the development of compound **11**, which exhibited potent anti-inflammatory and neuroprotective activity in OS-related in vitro models. Compound **11** also demonstrated high liver microsomal stability and favorable pharmacokinetics in mice, making it a promising candidate for further investigation as a potential PD therapy.

## 1 Introduction

**Figure 1:**
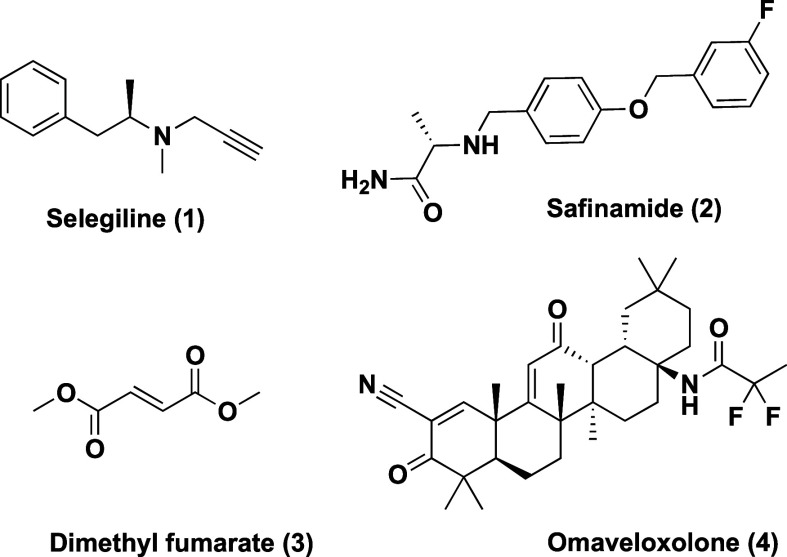
Clinically used MAO-B inhibitors (selegiline and safinamide) and NRF2 inducers (dimethyl fumarate and omaveloxolone).

Parkinson’s disease (PD) is an age-associated neurodegenerative disorder characterized by motor deficits, resting tremor, rigidity, bradykinesia, and cognitive deficit.^1^ At the molecular level, PD pathological markers are protein aggregates, known as Lewy bodies, and composed of α-synuclein, at *substantia nigra pars compacta*, and progressive loss of dopaminergic neurons with the subsequent reduction in dopamine levels.^2^ Despite an intense research effort over the last decades, PD physiopathology is not fully understood, and available treatments are limited to strategies directed to increase dopamine levels, exemplified by levodopa (l-dopa), monoamine oxidase B (MAO-B) inhibitors, and dopamine receptor agonists (Figure 1).^3^ However, these pharmacological alternatives are merely symptomatic, being unable to delay or prevent the disease’s advance. Thus, finding a real PD-modifying drug is an urgent unmet medical need. Recent investigations are shedding light on PD etiology, leading to its classification as a complex disease in which oxidative stress (OS), mitochondrial dysfunction, chronic neuroinflammation, and autophagy failure play a key role in its onset and progression. Moreover, there is evidence of a number of interconnections between each of these pathological pathways to exponentially accelerate neurodegeneration.^4,5^

MAO enzymes (A and B isoforms) are flavin adenine dinucleotide (FAD)-containing proteins in charge of the oxidative deamination of biological amines, particularly dopamine. MAO-B presents elevated activity in PD brains^6^ being implicated in dopamine levels reduction. Simultaneously, its catalytic cycle produces high levels of hydrogen peroxide (H_2_O_2_) and other reactive oxygen species (ROS). Thus, its activity notably contributes to OS exacerbated status and, finally, to neurodegeneration.^7^ Moreover, MAO-B expression is upregulated with increasing age, which is the main PD risk factor. MAO-B is predominantly expressed in glia, and it is augmented due to gliosis; therefore, glial cells neighboring dopaminergic neurons intensify OS by dopamine metabolism.^8,9^ Consequently, while existing MAO-B inhibitors provide only symptomatic relief without altering disease progression, these recent discoveries underscore the therapeutic promise of selective MAO-B inhibition.

Neuroinflammation is a key pathological process in PD onset and advance.^10,11^ The central nervous system possesses its own resident immune cells, of which microglia is described to trigger neuroinflammation.^12^ Glial cells are chronically activated by aberrant α-synuclein aggregates and exacerbated OS contributing to neurodegeneration by increasing ROS and producing proinflammatory cytokines.^13^ Increasing evidence correlates chronic microglial dysregulation to PD complex physiopathology; thus, attempts to find a microglial activation antagonist with therapeutic potential are a hot topic in medicinal chemistry research.

Nuclear factor erythroid 2-related factor (NRF2) regulates the phase II antioxidant response, a key intrinsic defense toward OS and inflammation. It regulates approximately 1% of human genes via the antioxidant response element (ARE) sequences.^14^ Particularly, NRF2 upregulates the expression of heme-oxygenase I (HMOX1), glutamate cysteine-ligase regulatory subunit (GCLm), NAD(P)H/quinone oxidoreductase (NQO1), and sequestosome-2 (SQSTM), among others. In addition, NRF2 activation suppress inflammatory responses by various mechanisms, including a functional cross-talk with NF-κB that inhibits the NF-κB pathway.^15^ Moreover, NRF2 exerts a direct regulation of the expression of pro-inflammatory genes by binding ARE sequences [e.g., CD36 is related in the NLR family pyrin domain containing 3 (NLRP3) inflammasome activation^16,17^] or by inhibiting RNA polymerase II recruitment due to NRF2 binding to the proximity of these genes (e.g., IL-6 and IL-1 IL-1β cytokines).^18^ NRF2-related genes are overexpressed in PD patients in an effort to counteract the high OS and neuroinflammation during disease advance, although natural response is not sufficient to control increased OS,^19^ thus, NRF2 pharmacological activation is considered a beneficial strategy for OS control. Moreover, a protective *NFE2L2* promoter single nucleotide polymorphism haplotype is associated with delayed onset and reduced PD risk.^20^ Considering OS and neuroinflammation roles in PD, NRF2 activation is considered a key pharmacological goal to reduce PD advance.

Recently, our research group reported the first example of a dual NRF2 inducer—MAO-B inhibitor with demonstrated moderate anti-inflammatory properties.^21^ Dual-targeted derivatives significantly reduced neuronal death in two in vitro and two ex vivo PD models by reducing ROS production. However, they showed significant cellular toxicity, compromised stability due to the presence of an ester moiety, and limited BBB permeability with *P*_e_ values close to the threshold in the parallel artificial membrane permeation assay (PAMPA) assay. Therefore, we report the optimization campaign of previously developed multitarget NRF2 inducers-MAO-B inhibitors devoted to developing a novel drug candidate with improved pharmacological, physicochemical, pharmacokinetic, and toxicological properties. Novel modifications are directed to (1) enhance their potency and selectivity toward MAO-B while maintaining their NRF2 activation capacity and (2) improve their drug-like properties to reduce toxicity and increase CNS permeability and compound stability.

## 2 Results and Discussion

### 2.1 In Silico Design Optimization, Molecular Modeling, and Virtual Screening

**Figure 2:**
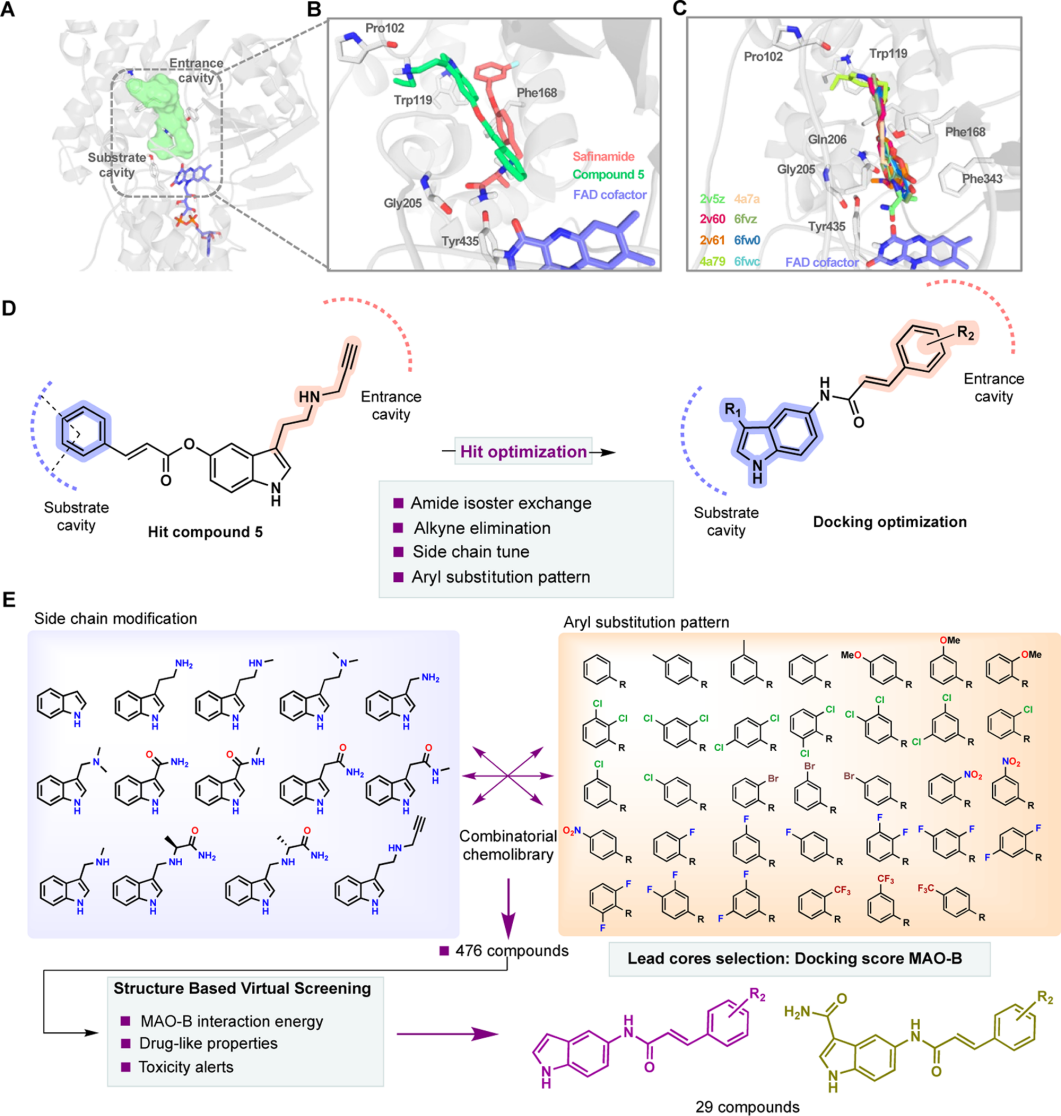
Pharmacophore-based hit optimization process and lead cores selection. (A) Global view of the MAO-B enzyme (PDB-ID 2BK3), highlighting the active site as a pale green surface. (B) Detailed position of safinamide and compound **5** at the MAO-B binding pocket after docking and molecular dynamics (MD) simulation (300 ns), highlighting key interacting residues. (C) Alignment of different MAO-B crystal structures in complex with known inhibitors. MAO-B is represented as a gray cartoon. (D) Schematic representation of the optimization program employed for novel compound design. (E) In-house chemical library submitted to SBVS toward MAO-B.

Based on previous results,^21^ we designed a novel virtual chemical library to comply with three different objectives: (1) to improve MAO-B inhibitory potency and selectivity; (2) to maintain NRF2 induction capacity; and (3) to enhance their BBB crossing capacity and absorption, distribution, metabolism, excretion, and toxicity (ADMET) properties. The optimization process started by modifying the chemical core of our hit molecule (**5**, Figure 2) based on key interactions of highly active MAO-B inhibitors like safinamide or 1,4-diphenyl-2-butene. Safinamide positions its phenyl ring in the vicinity of Phe^168^, interacting with Leu^167^ and Ala^165^, among others. Another important feature is the hydrogen bond with Gln^206^, a remarkable interaction considering that this residue is involved in substrate/inhibitor recognition and orientation at the coenzyme surroundings (B, Figure 2).^22,23^ Considering these interactions, we designed novel substitution patterns at the indole core to shorten its side chain, and the alkyne moiety was removed to include new polar substituents. These were designed to be located close to the FAD coenzyme, at the cavity entrance, aiming to expand their interaction network with protein residues. This modification also restricts undesired interactions at Pro^102^ surroundings (C, Figure 2). Additionally, several selected polar moieties are nonprotonable, aiming to establish polar contacts while avoiding protonated status in order to facilitate BBB permeability. Although in some cases designed polar substituents increase the topological polar surface area (TPSA), the combination of higher affinity toward MAO-B and nonprotonable moieties could offer higher benefits in our optimized derivatives compared to the potential reduction of BBB permeability due to TPSA increase. Importantly, most known MAO-B inhibitors show indeed similar binding modes to safinamide, as illustrated in part C, representing an alignment of the crystal structures of different inhibitors with the safinamide-MAO-B complex structure. Novel structures are in agreement with reported models proposing that large, rigid aryl cores with polar substituents are placed in the substrate cavity, separated by an electron-rich hydrogen-bonding linker from a small hydrophobic aryl moiety with electronegative substituents.^24^ Proposed structural modifications were also restricted to improve their drug-like properties, eliminating putatively toxic, chemically reactive, or metabolically unstable moieties.^25^ The final virtual chemical library was integrated by 476 compounds divided into 14 different core families (Table S1, Supporting Information) and was subjected to a structure-based virtual screening (SBVS) toward MAO-B using three different crystals. These crystal structures had an open conformation of the active site since this class of compounds was not predicted to dock only at the substrate cavity.

Docking score results and ADMET filters resulted in the selection of 29 compounds for synthesis, belonging to two different general families with the lowest mean energies: the 1*H*-indole and 1*H*-indole-3-carboxamide subfamilies. Molecular docking results with the selected poses for each subfamily are summarized in Figure S1 (see Supporting Information). Some of them showed lower energy binding modes with a different and nonpreferred orientation at the MAO-B active site in support of our filtering criteria (Figure S2, Supporting Information).

### 2.2 1*H*-Indole and 1*H*-Indole-3carboxamide Derivatives Synthesis

**Scheme 1:**
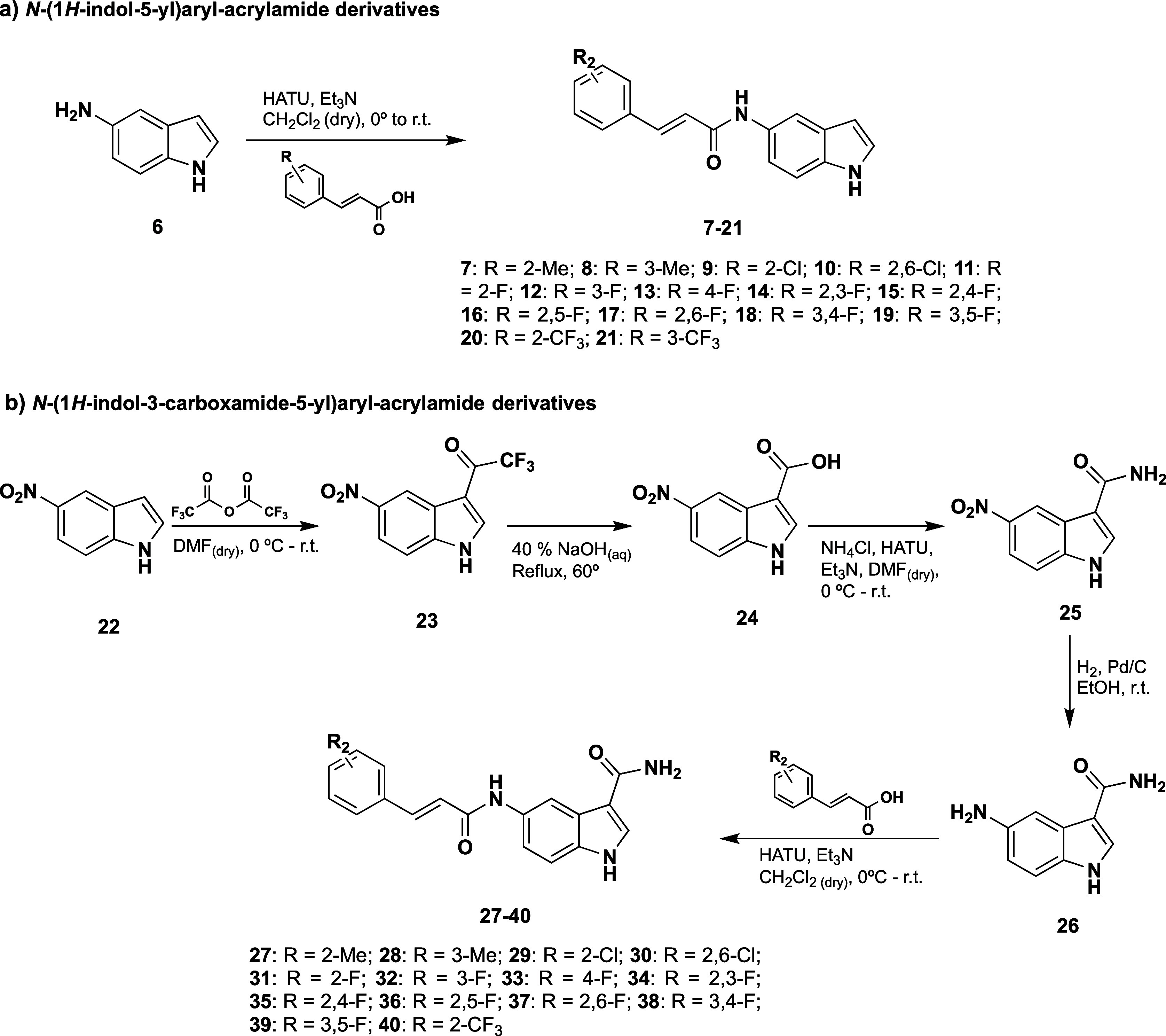
General Synthetic Procedure of *N*-(1*H*-Indol-5-yl)aryl-acrylamide and *N*-(1*H*-Indol-3-carboxamide-5-yl)aryl-acrylamide Compounds

The synthesis of *N*-(1*H*-indol-5-yl)aryl-acrylamide derivatives (**7–21**) was accomplished by catalytic amidation in one step from 5-aminoindole and the corresponding aryl-acrylic acid derivative, using 1-[bis(dimethylamino)methylene]-1*H*-1,2,3-triazolo[4,5-*b*]pyridinium 3-oxide hexafluorophosphate (HATU) as the coupling reagent (a, Scheme 1). Conversely, *N*-(1*H*-indol-3-carboxamide-5-yl)aryl-acrylamide derivatives were obtained following a 5-step synthetic procedure starting from 5-nitroindole (b, Scheme 1). First, the acylation of 22 with trifluoroacetic anhydride (TFAA) led to the 2,2,2-trifluoro-1-(5-nitro-1*H*-indol-3-yl)ethan-1-one (**23**) intermediate, which was then hydrolyzed in aqueous NaOH solution at 60 °C to yield the corresponding 5-nitro-1*H*-indole-3-carboxylic acid (**24**). The carboxylic acid functional group was employed for an amidation reaction with ammonium chloride using HATU as a coupling reagent, affording 5-nitro-1*H*-indole-3-carboxamide (**25**) as a product. Finally, catalytic hydrogenation over palladium on carbon was performed to yield the 5-amino-1*H*-indole-3-carboxamide (**26**), being the common precursor for the *N*-(1*H*-indol-3-carboxamide-5-yl)aryl-acrylamide subfamily (**27–40**). Compound **26** was then coupled with the corresponding aryl-acrylic acid derivatives to obtain the final *N*-(1*H*-indol-3-carboxamide-5-yl)aryl-acrylamide derivatives by using the same amidation conditions previously described. All novel derivatives were obtained in moderate to high yields (Table S2 and Supporting Information).

## 3 Pharmacological Evaluation

### 3.1 NRF2 Induction, MAO Inhibitory Capacity, and Selectivity

Compounds **7–21** and **27–40** included the aryl-acrylate moieties designed to covalently react to KEAP1 cysteine residues, thus activating the NRF2-ARE pathway. Bioisosteric ester-to-amide conversion included in the novel derivatives is expected to maintain NRF2 induction activity, and the different aryl substitutions explored are designed to modulate their potency. Thus, electronegative substituents are expected to increase the electrophilic character of the novel compounds. NRF2 induction capacity of **7–21** and **27–40** derivatives was evaluated in the AREc32 cell line.^26^ AREc32 cells are stably transfected human mammary MCF7 cells with the plasmid pGL-8xARE that contains 8 copies of the ARE sequence followed by luciferase reporter gen; thus, NRF2-ARE pathway activation results in a directly proportional luciferase expression that can be measured by a luminescence assay. CD values (concentration able to double reporter luciferase expression) are given in Table 1.

**Table 1:** Primary Targets Evaluation of Derivatives **7–21** and **27–40**^a^

compound	R_2_	CD (μM) NRF2 induction	IC_50_ MAO-B (μM)	IC_50_ MAO-A (μM)	selectivity index (MAO-A/MAO-B)
melatonin		>30	>100	>100	
safinamide		NE	0.0746 ± 0.00087	>30	395.7
TBHQ		1.82 ± 0.092	NE	NE	
**7**	2-Me	5.43 ± 1.2	5.50 ± 0.82	>30	>5.45
**8**	3-Me	5.51 ± 1.7	17.4 ± 1.7	>30	>1.72
**9**	2-Cl	14.5 ± 2.3	3.66 ± 0.39	>30	>8.20
**10**	2,6-Cl	>20	>30	>30	
**11**	2-F	0.361 ± 0.074	3.61 ± 0.57	>30	>8.31
**12**	3-F	7.75 ± 1.4	15.3 ± 2.9	>30	>1.96
**13**	4-F	>20	3.41 ± 0.21	>30	>8.80
**14**	2,3-F	>20	4.05 ± 0.60	>30	>7.41
**15**	2,4-F	>20	1.35 ± 0.35	14.2 ± 0.57	10.52
**16**	2,5-F	0.840 ± 0.12	5.03 ± 0.48	>30	>5.96
**17**	2,6-F	5.10 ± 1.9	4.91 ± 0.39	>30	>6.11
**18**	3,4-F	>20	7.87 ± 1.1	>30	>3.81
**19**	3,5-F	>20	18.7 ± 1.6	>30	>1.60
**20**	2-CF_3_	7.28 ± 0.96	2.23 ± 0.35	>30	>13.45
**21**	3-CF_3_	>20	>30	>30	
**27**	2-Me	>20	>30	>30	
**28**	3-Me	>20	21.1 ± 2.9	28.0 ± 2.3	1.33
**29**	2-Cl	14.4 ± 1.8	>30	>30	
**30**	2,6-Cl	8.05 ± 0.58	9.95 ± 1.4	>30	>3.02
**31**	2-F	10.0 ± 1.8	>30	>30	
**32**	3-F	>20	20.0 ± 4.8	>30	>1.50
**33**	4-F	>20	26.4 ± 2.6	19.4 ± 4.8	0.73
**34**	2,3-F	4.75 ± 0.86	18.3 ± 2.2	>30	>1.64
**35**	2,4-F	>20	>30	>30	
**36**	2,5-F	>20	21.2 ± 4.2	>30	>1.42
**37**	2,6-F	1.21 ± 0.31	>30	>30	
**38**	3,4-F	>20	21.4 ± 2.9	>30	>1.40
**39**	3,5-F	>20	21.6 ± 3.9	>30	>1.39
**40**	2-CF_3_	>20	>30	25.0 ± 3.5	<0.83

a NRF2 induction capacity and MAO inhibition of **7–21**, **27–40** derivatives and reference compounds. CD and MAO-B IC_50_ values were obtained from dose–response curves. NE: not evaluated. Data are expressed as mean ± SEM of four (NRF2) and five (MAO-B) independent experiments.

Interestingly, we obtained some remarkably potent compounds compared to the previously reported 2-(1*H*-indol-3-yl)ethan-1-amine derivatives, most notably compounds **11** (CD = 0.361 ± 0.074 μM; R_2_ = 2-F) and 16 (CD = 0.840 ± 0.12 μM; R_2_ = 2,5-F), with nanomolar activity and being more potent than reference compound TBHQ. In general, the *N*-(1*H*-indol-5-yl)aryl-acrylamide subfamily with an unsubstituted indole core demonstrated increased NRF2 induction potency compared to *N*-(1*H*-indol-3-carboxamide-5-yl)aryl-acrylamide derivatives. Thus, the inclusion of an amide moiety at 3-position of the indole core for *N*-(1*H*-indol-5-yl)aryl-acrylamide derivatives decreases their affinity toward KEAP1. Considering the substitution pattern at the aromatic ring, their NRF2 activation capacity decreased from ortho to meta and para-substitution, i.e., fluorine monosubstituted compounds: CD = 0.361 ± 0.074 μM for *o*-F (**11**); CD = 7.75 ± 1.4 μM for *m*-F (**12**); and CD > 20 μM for *p*-F (**13**). Focusing on disubstituted derivatives, chlorine inclusion led to no activity below 20 μM, and, in the case of fluorine atoms, only ortho and meta disubstituted derivatives showed significant NRF2 activation. In this sense, only compounds with fluorine atoms at the 2-position and 5 or 6-position of the phenyl ring were active (i.e., CD = 0.840 ± 0.12 μM for compound **16** with 2,5-F substitution; CD = 5.10 ± 1.9 μM for compound **17** with 2,6-F substitution), in good agreement with the substitution pattern effects (ortho > meta > para). Similar results depending on ring location have been reported for different series of NRF2 activators, such as curcumin derivatives^27^ and other Michael acceptors.^28^ This effect is proposed to be determined by steric hindrances at the surroundings of KEAP1-Cys residues, favoring the ortho position. Thus, voluminous methyl and trifluoromethyl groups led to active compounds only at the *o*-Ph position. Additionally, electron-withdrawing groups at the Ph ring might increase the Michael acceptor electrophilicity, as the inductive effect depends on distance, being directly proportional to their potency order (ortho > meta > para). Thus, compound **11** with fluorine at the 2-position combines both favorable steric (i.e., ring location and substituent size) and inductive effects, leading to the most potent derivative of the series (CD = 0.361 ± 0.074 μM).

*N*-(1*H*-Indol-3-carboxamide-5-yl)aryl-acrylamide derivatives partially share the above-mentioned structure–activity relationships. Overall, only ortho substituents led to active compounds [i.e., **29** (R_2_ = 2-Cl, CD = 14.4 ± 1.8 μM); **30** (R_2_ = 2,6-Cl, CD = 8.05 ± 0.58 μM]; **31** (R_2_ = 2-F, CD = 10.0 ± 1.8 μM); **34** (R_2_ = 2,3-F, CD = 4.75 ± 0.86 μM); and **37** (R_2_ = 2,6-F, CD = 1.21 ± 0.31 μM), although in this case, no activity was observed with either methyl or trifluoromethyl groups. As mentioned above, *N*-(1*H*-indol-3-carboxamide-5-yl)aryl-acrylamide compounds showed, in general, reduced NRF2 activity compared to *N*-(1*H*-indol-5-yl)aryl-acrylamide derivatives. As exceptions, compound **30**, with 2,6-Cl substitution, exhibited moderate activity (CD = 8.05 ± 0.58 μM) compared to its related compound **10** from *N*-(1*H*-indol-5-yl)aryl-acrylamide subfamily (CD > 20 μM) and compound **34** with 2,3-F substitution (CD = 4.75 ± 0.86 μM) compared to its related compound **14** (CD > 20 μM). Despite such differences, these compounds with double substitution patterns only in the ortho position (**30**) and ortho/meta position (**34**) continue following the general SAR previously discussed.

Previously described aryl-acrylate moieties linked to the melatonin core led to novel derivatives fulfilling important pharmacophoric features observed in well-known MAO-B inhibitors.^24^ Our novel design is expected to exert higher selectivity and potency toward MAO-B inhibition. Importantly, we selected only trans derivatives, as other structurally similar compounds have demonstrated a stereoselective activity of cis/trans isomers where the cis isomers were selective MAO-A inhibitors, while trans derivatives selectively targeted MAO-B.^29^ In general, the compounds described here showed selective MAO-B inhibitory capacity with IC_50_ values ranging from 1.35 ± 0.35 μM (**15**) to 26.4 ± 2.6 μM (**33**, Table 1), considering only derivatives with potencies lower than 30 μM. In line with our previous results, remarkable differences between *N*-(1*H*-indol-5-yl)aryl-acrylamide and *N*-(1*H*-indol-3-carboxamide-5-yl)aryl-acrylamide subfamilies were obtained. *N*-(1*H*-indol-5-yl)aryl-acrylamide derivatives showed higher potency and selectivity than MAO-B inhibitors. Analyzing structure–activity relationships inside *N*-(1*H*-indol-5-yl)aryl-acrylamide subfamily, inhibition strength seems to be undermined by meta substituents (**8**, 3-Me; **12**, 3-F; **18**, 3,4-F; **19**, 3,5-F; and **21**, 3-CF_3_) and substituent volume (**10**, 2,6-Cl; **21**, 3-CF_3_), being these compounds the poorest inhibitors, favoring in general the ortho position compared to meta and para positions. This hypothesis is supported by SBVS results where most of the substituents showed the best docking scores for ortho-substituted derivatives (i.e., Cl, Me, and CF_3_-substituted compounds; data not shown). Compounds with ortho-monosubstitution exhibited similar potencies (IC_50_ = 5.50 ± 0.82 μM for compound **7** with 2-Me substitution; IC_50_ = 3.66 ± 0.39 μM for compound **9** with 2-Cl substitution; IC_50_ = 3.61 ± 0.57 μM for compound **11** with 2-F substitution; CD = 2.23 ± 0.35 μM for compound **20** with 2-CF_3_ substitution). Interestingly, compound **7**, bearing a nonhalogenated substituent, was the weakest inhibitor of *o*-derivatives. This is in agreement with previous models suggesting that small hydrophobic aryl moieties with electronegative substituents could potentiate the inhibition, fitting in the entrance cavity of the enzyme binding pocket.^24^ Compound **15**, with a double fluorine substitution in the ortho and para positions, was the most potent MAO-B inhibitor of the *N*-(1*H*-indol-5-yl)aryl-acrylamide derivatives with an IC_50_ = 1.35 ± 0.35 μM. Nevertheless, it also showed a moderate potency for MAO-A inhibition (IC_50_ = 14.2 ± 0.57 μM), suggesting that this aryl substitution pattern could enhance MAO activity in general, although with moderate selectivity (SI = 10.5).

As discussed, the 1*H*-indole-3-carboxamide derivatives showed overall reduced potencies toward MAO-B inhibition. Remarkably, ortho-monosubstituted compounds with electronegative atoms displayed at least a 10-fold increased potency comparing 1*H*-indole and 1*H*-indole-3-carboxamide subfamilies. This contrast could be related with key interactions of the indole core at the substrate cavity near the FAD coenzyme. Additionally, increased molecular weight in the 1*H*-indole-3-carboxamide subfamily due to amide inclusion could also be detrimental for MAO-B inhibition regarding the rigid and narrow shape of the enzyme binding pocket.^7^ There are several MAO-B inhibitors, including indole moieties in their structures^30−32^ and also aryl-acrylate derivatives.^33^ Some of them also share other structural features with our compounds, as a recently reported series of *N*-(1*H*-indol-5-yl)benzamides.^34^

### 3.2 Antioxidant Capacity, Anti-inflammatory Effect, and Blood–Brain Barrier Permeation Capacity

Given the structural relationship of our compounds with melatonin, they were anticipated to exert free radical scavenging capability, which would be of interest in view of the OS implication in PD. We used the oxygen radical absorbance capacity (ORAC) assay to evaluate their ROS scavenger capacity (Table 2). Novel compounds showed, overall, higher scavenger capacity than Trolox, and more importantly, all *N*-(1*H*-indol-5-yl)aryl-acrylamide derivatives exhibited similar activity to melatonin, being in most cases more potent. Their scavenging activity ranged from 2.69 ± 0.53 T.equiv of compound **13** to 3.46 ± 0.19 T.equiv of compound **19**. Considering only fluorine derivatives, there is a slight tendency of higher scavenging capacity for disubstituted derivatives. Regarding the 1*H*-indole-3-carboxamide subfamily, compounds showed, in general, comparable activity to Trolox. It is important to highlight the significant differences between *N*-(1*H*-indol-3-carboxamide-5-yl)aryl-acrylamide compounds, with almost 3-fold increased potency for *N*-(1*H*-indol-5-yl)aryl-acrylamide derivatives. Focusing on melatonin free radical-scavenging activity, several mechanisms have been proposed, including the formation of an indolyl cation and direct oxygen species addition to the indole moiety, involving electron or hydrogen atom transfer processes.^35^ Additionally, some studies have proposed that the 5-methoxy and the *N*-acetyl moieties in melatonin do not seem to significantly influence its thermodynamic capacity for scavenging hydroxyl radicals.^36^ All of these observations highlight the key value of the indole core on melatonin scavenging activity. Therefore, the inclusion of an amide group in the *N*-(1*H*-indol-3-carboxamide-5-yl)aryl-acrylamide derivatives might have negative consequences affecting the indole electronic properties and resulting in the reduced activity of these compounds. Furthermore, the high reactivity at the 3-position of the indole core, unsubstituted in the *N*-(1*H*-indol-5-yl)aryl-acrylamide subfamily, could be assisting radical trapping reactions.

**Table 2:** Antioxidant, Anti-inflammatory, and BBB Permeability Properties of **7–21**, **27–40,** and Reference Compounds^a^

				PAMPA	
compound	R_2_	ORAC (T.equiv)	EC_50_ nitrite reduction (μM)	*P*_e_ (10^–6^ cm s^–1^)	prediction
melatonin		2.83 ± 0.18	25.7 ± 2.5	NE	NE
safinamide		NE	NE	25.6 ± 6.7	CNS+
sulforaphane		NE	1.78 ± 0.48	NE	NE
**7**	2-Me	2.95 ± 0.060	11.8 ± 2.4	15.2 ± 1.3	CNS+
**8**	3-Me	3.32 ± 0.40	9.41 ± 0.66	13.5 ± 1.2	CNS+
**9**	2-Cl	3.05 ± 0.091	4.39 ± 1.8	16.7 ± 2.1	CNS+
**10**	2,6-Cl	2.88 ± 0.20	9.18 ± 2.4	15.4 ± 4.4	CNS+
**11**	2-F	2.77 ± 0.18	11.6 ± 1.4	15.1 ± 2.3	CNS+
**12**	3-F	2.87 ± 0.098	>30	13.8 ± 0.38	CNS+
**13**	4-F	2.69 ± 0.53	21.5 ± 2.5	19.4 ± 11	CNS+
**14**	2,3-F	3.10 ± 0.087	15.5 ± 2.5	14.5 ± 0.31	CNS+
**15**	2,4-F	2.75 ± 0.37	15.3 ± 5.5	13.8 ± 1.4	CNS+
**16**	2,5-F	3.15 ± 0.24	26.2 ± 4.6	18.5 ± 1.0	CNS+
**17**	2,6-F	2.75 ± 0.051	20.2 ± 4.3	13.1 ± 1.5	CNS+
**18**	3,4-F	3.36 ± 0.30	>30	15.7 ± 2.2	CNS+
**19**	3,5-F	3.46 ± 0.19	16.3 ± 3.5	25.5 ± 7.8	CNS+
**20**	2-CF_3_	3.04 ± 0.30	>30	13.4 ± 2.6	CNS+
**21**	3-CF_3_	3.01 ± 0.096	>30	18.7 ± 4.2	CNS+
**27**	2-Me	1.23 ± 0.042	16.9 ± 2.3	1.19 ± 0.58	CNS–
**28**	3-Me	1.18 ± 0.096	23.5 ± 2.3	1.10 ± 0.90	CNS–
**29**	2-Cl	1.04 ± 0.065	22.7 ± 4.1	0.919 ± 0.51	CNS–
**30**	2,6-Cl	0.937 ± 0.053	13.0 ± 2.4	1.87 ± 0.60	CNS–
**31**	2-F	1.38 ± 0.020	13.4 ± 1.6	0.860 ± 0.74	CNS–
**32**	3-F	1.01 ± 0.094	22.9 ± 0.83	1.49 ± 1.3	CNS–
**33**	4-F	1.19 ± 0.037	11.0 ± 3.1	1.52 ± 1.0	CNS–
**34**	2,3-F	1.23 ± 0.057	10.5 ± 2.4	0.423 ± 0.29	CNS–
**35**	2,4-F	1.14 ± 0.091	>30	0.05 ± 0.06	CNS–
**36**	2,5-F	1.19 ± 0.10	26.8 ± 2.9	0.645 ± 0.44	CNS–
**37**	2,6-F	0.870 ± 0.14	16.1 ± 2.4	1.95 ± 1.9	CNS–
**38**	3,4-F	1.22 ± 0.038	28.7 ± 0.66	1.33 ± 0.66	CNS–
**39**	3,5-F	1.20 ± 0.14	>30	0.127 ± 0.40	CNS–
**40**	2-CF_3_	0.821 ± 0.083	15.3 ± 2.2	0.507 ± 0.17	CNS–

a Free radical scavenger capacity was calculated from dose–response curves using the ORAC assay. Nitrite production reduction was evaluated in BV2 microglial cells treated with LPS (1 μg/mL) for 18 h. Nitrite reduction EC_50_ values were calculated from dose–response curves. BBB permeability was evaluated by the PAMPA assay expressed as *P*_e_. NE: not evaluated. CNS+ (high BBB permeability): *P*_e_ (10^–6^ cm s^–1^) > 4.0; CNS– (low BBB permeability): *P*_e_ (10^–6^ cm s^–1^) < 2.0; CNS± (uncertain BBB permeability): *P*_e_ (10^–6^ cm s^–1^) between 4.0 and 2.0.^37^ Data are expressed as mean ± SEM of at least 3 independent experiments.

We next assessed their potential anti-inflammatory properties, considering that neuroinflammation is a crucial PD pathophysiology contributor.^38^ As previously described, NRF2 pathway activation reduces inflammatory status; thus, we assessed the ability of novel compounds to decrease nitrite production in LPS-stimulated BV2 cells. LPS leads to NF-κB activation, stimulating the expression of pro-inflammatory genes (e.g., *iNOS*, *NLRP3*, or pro-*IL1β*),^39^ along with polarization of microglia and astrocytes to an inflammatory state.^40,41^ BV2 microglial cells come from an immortalized murine microglia line, retaining microglial morphological and functional characteristics. The NF-κB pathway activation and the increased iNOS protein levels and nitrite production upon LPS activation in these cells have been widely demonstrated.^42,43^ In this regard, our optimized derivatives demonstrated potent anti-inflammatory capacity with EC_50_ values from 4.39 ± 1.8 μM for compound **7** to 28.7 ± 0.66 μM for compound **38** (Table 2). Phenyl ring substitutions do not seem to clearly modify this activity, impeding the establishment of structure–activity relationships, as most of the compounds showed a similar anti-inflammatory capacity. Remarkably, similar nitrite reduction effects, together with several pro-inflammatory mediators decrease (e.g., TNF-α and IL-1β cytokines), have been clearly demonstrated with other well-known NRF2 activators like sulforaphane,^43^ among others.^44^ These studies demonstrated a clear relationship between NRF2 pathway activation and NF-κB inhibition, recovering BV2 microglial cells from LPS-induced inflammatory condition.^44^ However, the activity of derivatives **7–21** and **27–40** in this assay showed no clear correlation with their NRF2 induction activity, indicating a potential complementary mechanism of action to exert its anti-inflammatory activity that might be operating together with NRF2 induction to exert its observed pharmacological effect. This result is in line with previously reported multitarget drugs, in which biological activities are the result of a combination of independent targets. Moreover, known covalent drugs with NRF2 induction properties have been described to exert their anti-inflammatory effect by inhibiting LPS binding to the TLR4-MD2 complex to prevent further TLR4 oligomerization and NF-κB pathway activation.^45^ In this line, we have recently described a complementary anti-inflammatory target on compound ITH12674, a covalent NRF2 inducer for which we have demonstrated its ability to block the LPS binding to the TLR4-MD2 complex, inhibiting TLR4 oligomerization.^46^ This mechanism of action cooperates with its NRF2 induction capacity to exert its anti-inflammatory properties. Compounds **7–21** and **27–40** bear a Michael acceptor moiety; thus, this mechanism might be potentially involved.

Finally, we explored the BBB permeability of compounds **7–21** and **27–40** by the PAMPA assay based on passive diffusion (Table 2). As observed, *N*-(1*H*-indol-5-yl)aryl-acrylamide derivatives are envisaged to cross the BBB, showing permeability (*P*_e_) values clearly higher than 4 × 10^–6^ cm s^–1^.^37^ On average, they exhibited permeability results around 16 × 10^–6^ cm s^–1^, in some cases, overcoming the positive control verapamil (*P*_e_ = 17.5 ± 4.2 × 10^–6^ cm s^–1^; Table S3). In contrast, *N*-(1*H*-indol-3-carboxamide-5-yl)aryl-acrylamide compounds showed poor permeability values, with all of them predicted to be unable to reach the CNS. These dramatic differences between both subfamilies could be explained by several factors. Mainly, it is important to point out that *N*-(1*H*-indol-5-yl)aryl-acrylamide subfamily has a reduced total polar surface area [TPSA; average value of 44.9 Å^2^ for *N*-(1*H*-indol-5-yl)aryl-acrylamide compared to 88.0 Å^2^ for *N*-(1*H*-indol-3-carboxamide-5-yl)aryl-acrylamide compounds] and fewer hydrogen bond donors [HBD; 2 for *N*-(1*H*-indol-5-yl)aryl-acrylamide compared to 3 for *N*-(1*H*-indol-3-carboxamide-5-yl)aryl-acrylamide compounds].^47^ It is well-known that smaller molecules with low TPSA are more likely to penetrate membranes through passive diffusion mechanisms.^48^ This can have a direct impact on brain targeting, and some studies with a set of marketed drugs establish that the median TPSA of CNS drugs (40.5 Å^2^) is lower than the mean value of non-CNS drugs (83.8 Å^2^; *p* < 0.0001).^49^ It is also reported that by increasing both HBD and the related TPSA, a decrease in the passive permeability of compounds is generally observed.^50,51^ Indeed, PAMPA studies suggest that increased HBD count is associated with lower passive permeability, this phenomenon being attributed to the desolvation of the associated hydrogen-bound water molecules, which is necessary for membrane permeability.^49,52^ When using biological membranes, an increased HBD value also raises the risk of P-gp recognition,^51^ which contributes to a decrease in free drug exposure in the brain. All of these observations highlight *N*-(1*H*-indol-5-yl)aryl-acrylamide over *N*-(1*H*-indol-3-carboxamide-5-yl)aryl-acrylamide compounds in terms of potential CNS permeability and may explain the results obtained in the PAMPA assay.

### 3.3 Compounds **7–21** and **27–40** Exert Neuroprotection against In Vitro PD-Related Models

**Figure 3:**
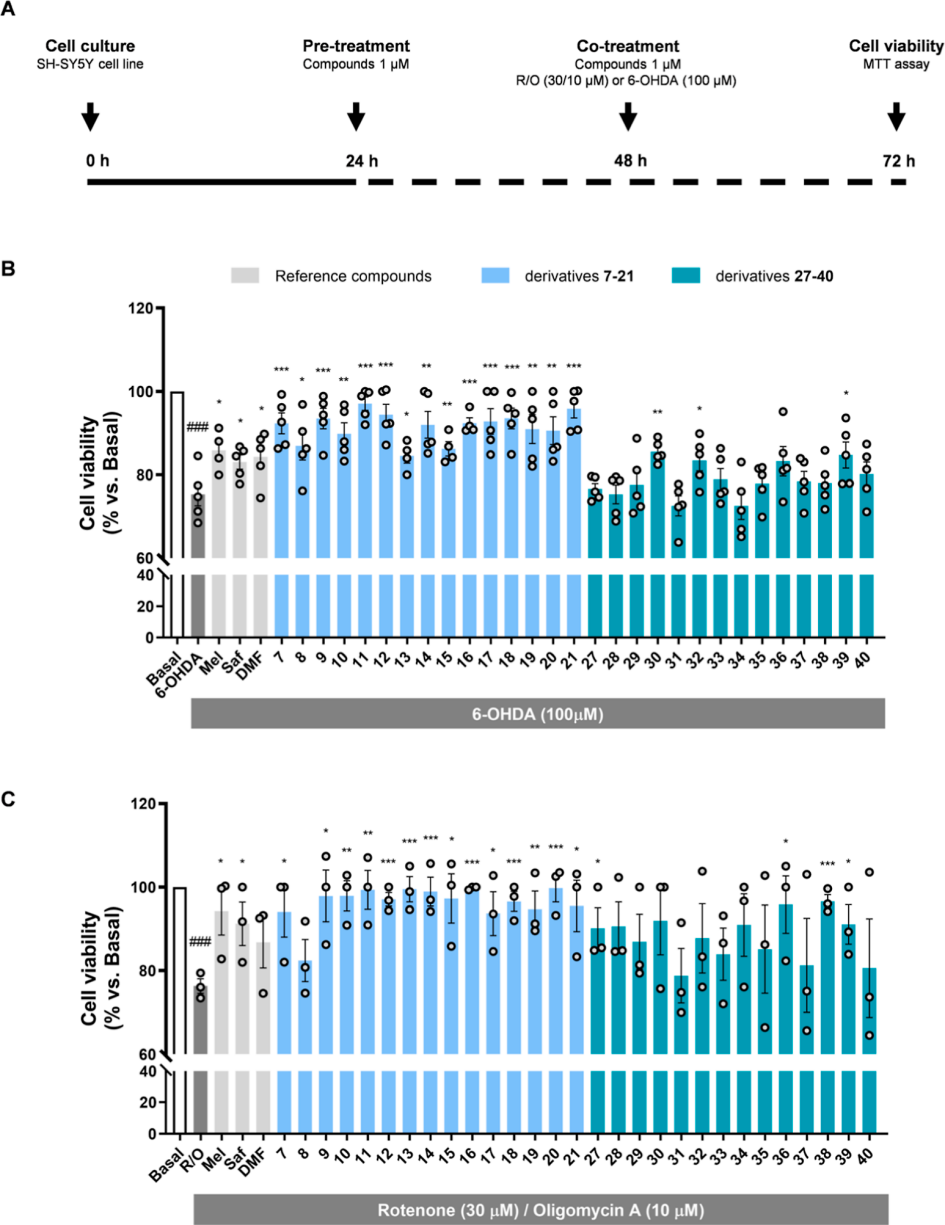
Neuroprotective activity of compounds **7–21** and **27–40** in the 6-hydroxydopamine and rotenone/oligomycin A mixture of OS-related models. (A) Experimental procedure schematic representation. SH-SY5Y cells were treated with compounds **7–21** and **27–40** or reference compounds (melatonin, safinamide, and dimethyl fumarate) (1 μM) for 24 h. Subsequently, cells were cotreated with derivatives **7–21**, **27–40**, or reference compounds (1 μM) and the corresponding toxic stimuli: (B) 6-OHDA (100 μM) or (C) R/O mixture (30/10 μM) for 24 h. Data are expressed as mean ± SEM of 3–5 independent experiments. Statistical analysis one-way ANOVA (*p* < 0.05). ^###^*p* < 0.001 vs basal condition, *p < 0.033, **p < 0.002, and ***p < 0.001 vs toxic condition after Tukey’s posthoc test.

After the preliminary pharmacological screening, demonstrating that novel compounds **7–21** and **27–40** were active at their main targets, we evaluated their neuroprotective potential in OS-related in vitro models. Given that OS is a primary factor contributing to neurodegeneration in PD,^5^ we selected the rotenone/oligomycin A combination and 6-OHDA models (Figure 3A).

As previously discussed, 6-OHDA is a widely used PD pathology model, selectively damaging dopaminergic neurons. 6-OHDA treatment leads to exacerbated OS due to its autoxidation and complex I mitochondrial respiratory chain direct inhibition.^53^ SH-SY5Y cells exposure to 6-OHDA induces a significant viability reduction, widely reproduced in the literature,^54^ and considering their dopaminergic phenotype,^55^ it is widely accepted as a relevant PD model. Pre- and cotreatment with compounds (1 μM) led to significant neuroprotection of the *N*-(1*H*-indol-5-yl)aryl-acrylamide subfamily and compounds **30**, **32**, and **39** from the *N*-(1*H*-indol-3-carboxamide-5-yl)aryl-acrylamide subfamily (Figure 3B). Interestingly, (1*H*-indol-5-yl)aryl-acrylamide derivatives showed a higher neuroprotective capacity than *N*-(1*H*-indol-3-carboxamide-5-yl)aryl-acrylamide derivatives (Figure 3). These differences can be explained by the overall better pharmacological profile of the *N*-(1*H*-indol-5-yl)aryl-acrylamide derivatives, as they present higher activity toward the main targets, antioxidant properties, and permeability. Considering the *N*-(1*H*-indol-5-yl)aryl-acrylamide subfamily, it is important to highlight that the treatment with most of these compounds led to almost a total viability recovery after the 6-OHDA toxic insult, exhibiting cell viabilities over 90% in most cases and neuroprotection values ranging from 36.6% by compound **13** to 89.7% by the most potent compound **11** (Table S4). In this case, there exists a slight correlation between primary targets included in compounds **7–34** and their neuroprotective profile against 6-OHDA, i.e., compound **11**, showed the highest protection capacity (89.7%), being the most potent NRF2 inducer, the second most potent MAO-B inhibitor, and a good scavenger. In this line, compounds with the lowest CD values and/or the lowest MAO-B inhibitory capacity showed the lowest neuroprotective activity, as in the cases of compounds **13** and **15** (36.6 and 44.6% protection). Nonetheless, compounds with low CD values and poor MAO-B inhibitory activity showed high neuroprotection that can be associated with a potent scavenger effect, i.e., compound **19**. Thus, we can hypothesize that their neuroprotective properties are related to the combination of pharmacological targets. This assumption could also be supported by the fact that *N*-(1*H*-indol-5-yl)aryl-acrylamide multitarget compounds displayed globally significantly better protective effects than reference compounds (melatonin as a well-known antioxidant; safinamide as a potent and selective MAO-B inhibitor; DMF as NRF2 activator). DMF had been previously shown to attenuate 6-OHDA neurotoxicity in the neuroblastoma cell line and in in vivo models due to its NRF2-activating activity.^56^ Melatonin^57^ and safinamide^58,59^ have been widely characterized in animal models employing this neurotoxin.

Finally, we selected the rotenone/oligomycin A combination as a complementary OS model.^60^ Pre- and cotreatment with compounds (1 μM) significantly reduced toxicity in most of the cases (Figure 3C). Similar to the previous model, all compounds from the *N*-(1*H*-indol-5-yl)aryl-acrylamide subfamily showed excellent neuroprotection, except compound **8**; however, only compounds **27**, **36**, **38**, and **39** from *N*-(1*H*-indol-3-carboxamide-5-yl)aryl-acrylamide subfamily were able to significantly protect cells toward mitochondrial OS. Again, the overall better profile of *N*-(1*H*-indol-5-yl)aryl-acrylamide compounds is related to a superior performance in this in vitro model, which shares a strong OS implication. In this case, there were fewer differences between *N*-(1*H*-indol-5-yl)aryl-acrylamide subfamily derivatives, with most of the compounds reaching viability values above 95% (Table S4). Similar to the previous model, *N*-(1*H*-indol-5-yl)aryl-acrylamide compounds displayed, in general, better results compared to the reference compounds in support of the multitarget approach.

Remarkably, derivatives **7–21** and **27–40** showed a clear improvement in terms of cytotoxicity compared to previous 2-(1*H*-indol-3-yl)ethan-1-amine derivatives. LD_50_ was at least higher than 100 μM in SH-SY5Y cells for all the compounds, except in the case of derivatives **27** and **31**, which displayed some toxicity at that concentration (Table S5). This accomplishes one of the main goals posed in the initial compounds design.

After completing the preliminary pharmacological screening, we selected compound **11** as the lead derivative considering its overall pharmacological profile. In brief, compound **11** showed the most potent NRF2 induction activity and potent and selective MAO-B inhibition. It also presents good radical scavenging, anti-inflammatory, and permeability properties. Finally, compound **11** shows the best neuroprotection results toward 6-OHDA toxicity, and it is also one of the best compounds in the complementary R/O mixture-based model. Thus, we focused on the exhaustive characterization of the mechanisms of action of this novel and promising derivative.

### 3.4 Compound **11** Mechanism of Action Evaluation: MAO-B Inhibition and NRF2 Induction

**Figure 4:**
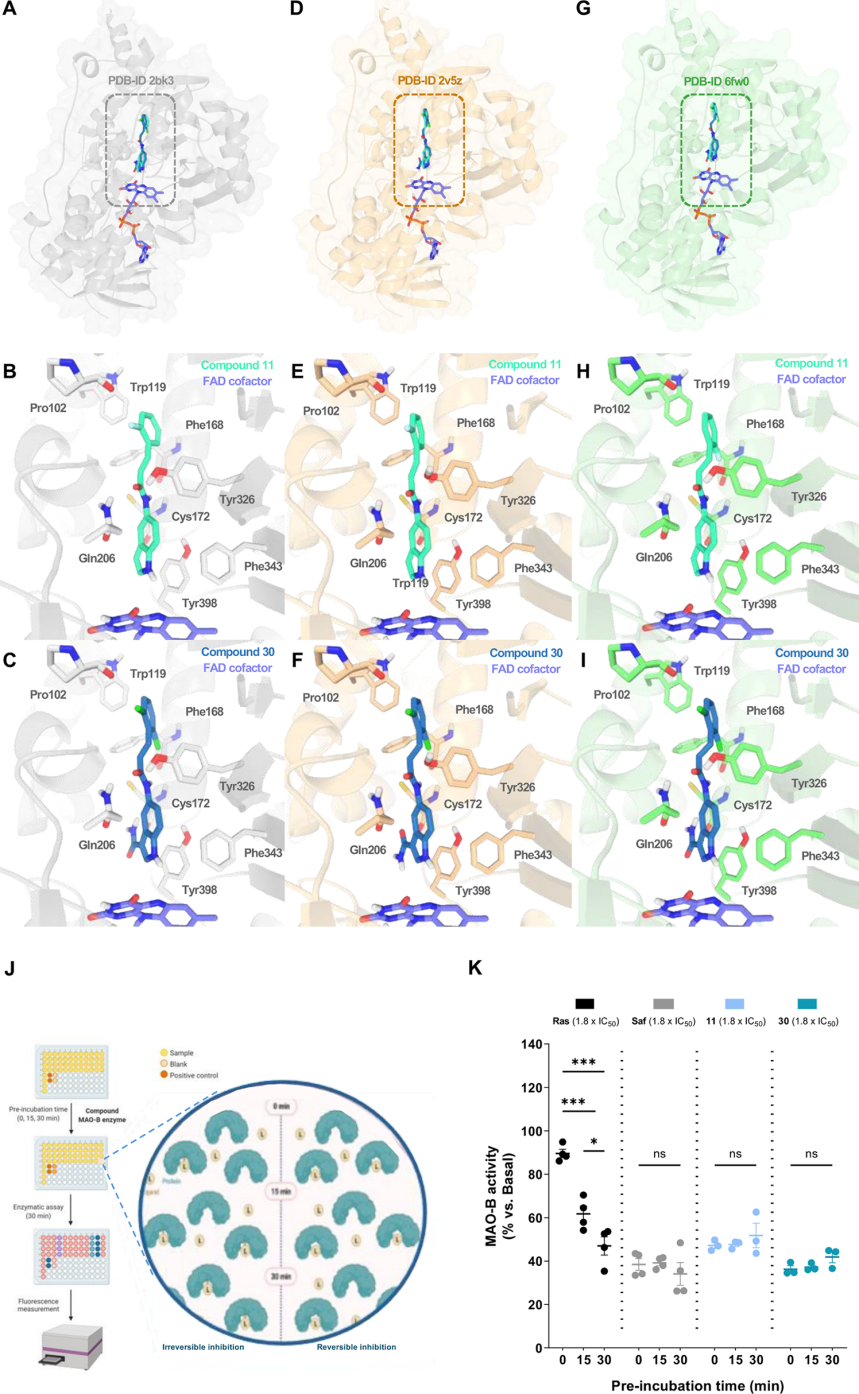
MAO-B binding mode determination for top *N*-(1*H*-indol-5-yl)aryl-acrylamide derivatives **11** and **30**. Overall representation of predicted interaction of **11** and **30** with MAO-B structures (A) PDB-ID 2BK3, (B) 2V5Z, and (C) 6FW0 by molecular docking. Detailed positions of compounds **11** (D–F) and **30** (G–I) at the active site of 2BK3, 2V5Z, and 6FW0, respectively, with key protein residues interacting with ligands (colored sticks). MAO-B is presented as a colored cartoon and surface. Compounds **11** and **30** are represented as light and dark blue sticks, respectively. (J) Reversibility assay experimental procedure of MAO-B kinetics. MAO-B activity was measured at three ligand-enzyme preincubation times (0, 15, and 30 min) using 1.8 × IC_50_ μM concentration for each ligand. Rasagiline and safinamide were employed as reference compounds for irreversible and reversible inhibition, respectively. (K) MAO-B reversibility assay results showing **11** and **30** reversible inhibition kinetics. Data are expressed as mean ± SEM of at least 3 independent experiments. Comparisons were made using a one-way ANOVA test (*p* < 0.05) followed by Newman–Keuls posthoc analysis. *p < 0.033 and ***p < 0.001.

The results obtained from the virtual screening program allowed us to characterize the binding mode of hit compound **11** at the MAO-B active site. For comparative purposes, we also analyzed compound **30**, the most potent inhibitor from the *N*-(1*H*-indol-3-carboxamide-5-yl)aryl-acrylamide subfamily. Figure 4A–I summarizes the structural details of the study performed with these compounds in the three crystal structures employed during virtual screening (PDB-IDs 2BK3, 2V5Z, and 6FW0).

In Figure 4A–C, both compounds **11** and **30** are represented in complex with each of the protein structures after molecular docking experiments. First, it is important to highlight that both ligands share a common location along the bipartite cavity of the MAO-B active site, occupying entrance and substrate pockets with only slight differences between them. They also exhibit almost identical binding modes in the 2BK3, 2V5Z, and 6FW0 MAO-B structures, respectively. Interestingly, novel derivatives showed a similar binding mode as safinamide, placing its phenyl ring at the vicinity of the Phe168 residue. Thus, they do not interact as closely with Pro102 compiling with our optimization program.

As seen in Figure 4D–F for compound **11** and Figure 4G–I for compound **30**, both ligands showed key hydrogen bonds with Gln206 and Tyr326 and hydrophobic interactions with Trp119, Phe168, Leu171, Ile199, Tyr326, and Phe343. Additional parallel displaced π–π interactions are established between both the indole ring of the compounds’ and Tyr398. Moreover, compound **30** showed hydrogen bonding with the Cys172 residue in the complexes with 2BK3 (Figure 4G) and 6FW0 (Figure 4I) and potential halogen bonding with Pro102 and Phe168 residues in all of the structures. It is remarkable that these novel derivatives interact with key residues related to substrate and inhibitor recognition as Gln206 and Tyr326.^22,23,61^ Compounds **11** and **30** also show hydrogen bonding with Tyr326. As shown in X-ray crystallography experiments^62^ and in our MD simulations, safinamide establishes a key hydrogen bond with Gln206 in a similar way to our novel compounds. Moreover, the observation that our novel compounds establish an edge-to-face π–π interaction with the FAD coenzyme is crucial. The indole cores of compounds **11** and **30** are located a short distance from the FAD flavin ring. This interaction, in addition to other contacts with Phe343 and Tyr398 residues, could help to stabilize compounds in this region of the substrate cavity near the FAD cofactor, which is involved in catalysis and substrate specificity.^63^

The predicted binding mode could partially explain the structure–activity relationships previously discussed for the MAO activity assays. In general, compounds with the bulkier substituents (i.e., CF_3_, Me, and Cl) share a common binding pattern in which the indole core maintains the position observed with **11** and **30**; however, the phenyl ring moiety is displaced and shows weaker interactions with Trp119 and Phe168 residues in the meta and more markedly in the para-substituted derivatives. In the case of CF_3_-substituted compounds, the para and meta derivatives also lose their key interaction with Gln206. This hypothesis is supported by docking scores that follow a positioning order (ortho < meta < para) for this series of substituents series, highlighting ortho compounds as the most stable. In fact, *p*-CF_3_, *p*-Me, and both *m*-Cl and *p*-Cl derivatives were not synthesized precisely because of their poor virtual screening results (data not shown). In summary, the higher stability of the interactions displayed by the ortho derivatives explains the improved inhibitory potency observed. For halogen-containing derivatives at phenyl rings, meta-substitution leads to a slight displacement of this aromatic region of the molecule away from the Trp119 and Phe168 surroundings (data not shown). This is also observed with fluorinated compounds in spite of the smaller size of this substituent, possibly due to a repulsive electrostatic interaction between the π-electron-rich aromatic rings of these protein residues and the highly polarized C-halogen dipole.^64^ Additionally, the *ortho*-fluorine substitution could lead to dipole–dipole electrostatic interactions with carbonyl moieties in the near amino acid residues, stabilizing these derivatives at the active site.^64,65^ These observations could serve to explain the structure–activity relationships in the fluorine series of compounds negatively affected by meta positioning.

One of the most relevant results in terms of MAO-B activity is the prominent difference in potency between the *N*-(1*H*-indol-5-yl)aryl-acrylamide and *N*-(1*H*-indol-3-carboxamide-5-yl)aryl-acrylamide subfamilies. *N*-(1*H*-Indol-3-carboxamide-5-yl)aryl-acrylamide subfamily compounds showed, in general, lower potency, and this could be explained by subtle differences in their binding mode. Mainly, amide inclusion at the indole core modifies its location at the substrate cavity. As seen in Figure 4A–C, the indole core of compound **30** is slightly away from the FAD coenzyme compared to compound **11**, and this is a general trend with other derivatives (data not shown). This displacement affects the edge-to-face π–π interaction strength with the FAD coenzyme flavin ring. Additionally, their amide moiety is an electron-withdrawing group that could reverse the direction of the overall quadrupole moment of the aromatic indole core, resulting in a central area of relative electron deficiency. This leads to a preference for face-centered stacking with electron-rich aromatics as the flavin ring of FAD coenzyme.^66^ Given that the π–π interactions between the indole core of the *N*-(1*H*-indol-5-yl)aryl-acrylamide compounds and this flavin ring follow an edge-to-face configuration, amide inclusion could be weakening them. Indeed, edge-to-face interactions are favored when both aromatic systems are electron-rich.^66^

Finally, to further define its inhibitory mechanism of action, we performed a reversibility inhibitory test (Figure 4J). Results are depicted in Figure 4K, demonstrating a reversible inhibition of compounds **11** and **30**, as expected. Upon incubation with these compounds, MAO-B activity remained time-invariant, similarly to safinamide, a widely described reversible inhibitor.^67^ Conversely, treatment with the irreversible inhibitor rasagiline resulted in increased inhibition over time. Reversible inhibitors are considered safer since this class does not induce diamine oxidase to compensate for the loss of MAO-B total activity as observed for irreversible inhibitors, a compensating mechanism that leads to GABA-mediated astrogliosis.^68^

**Figure 5:**
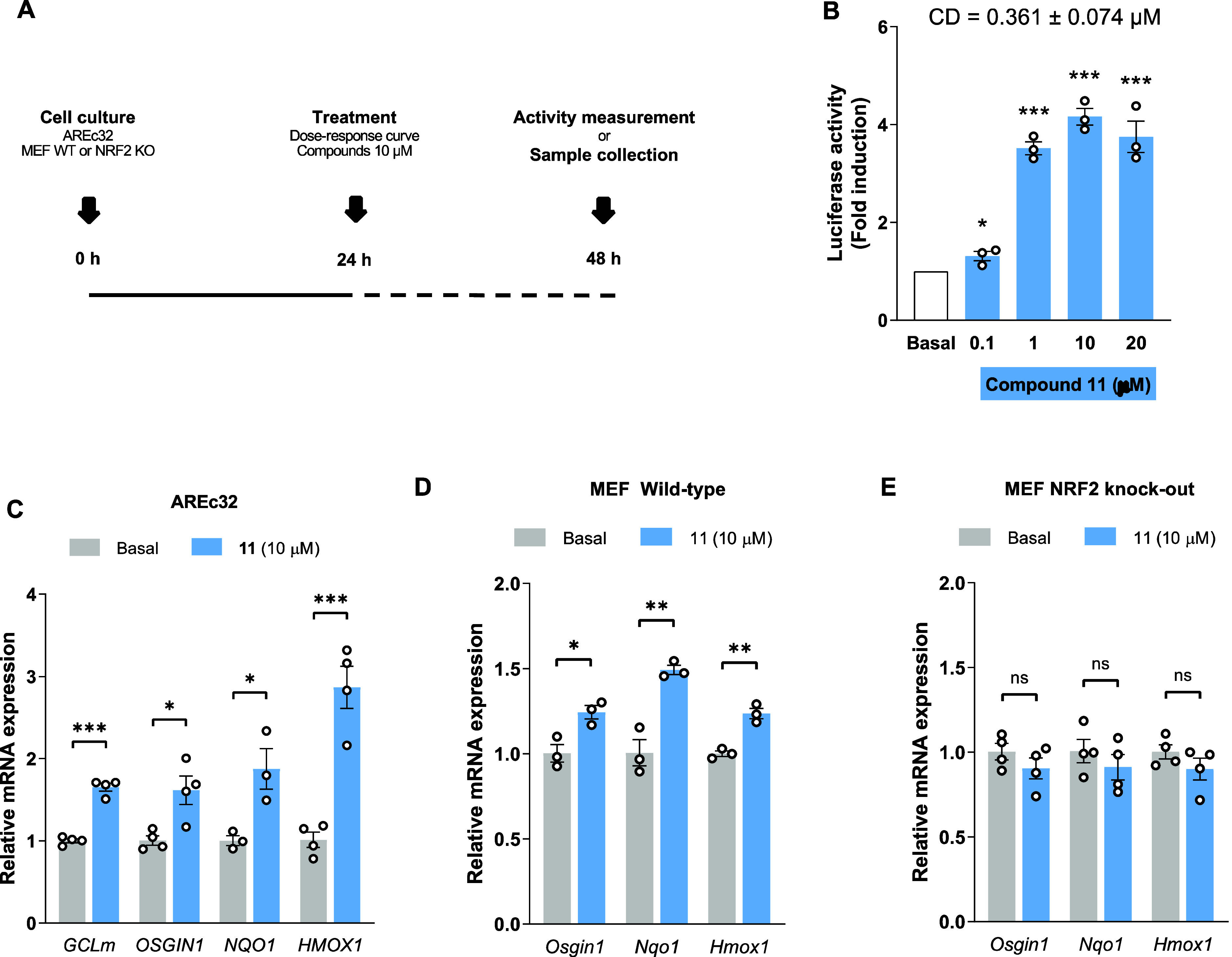
Compound **11** upregulates the phase II antioxidant pathway via NRF2. (A) Experimental protocol. (B) Dose–response luciferase activity in AREc32 cells upon compound **11** treatment (24 h). Relative NRF2-dependent gene mRNA expression levels in (C) AREc32, (D) MEF wild-type, and (E) MEF NRF2 knockout cells. Data are expressed as mean ± SEM of 3 or 4 independent experiments. Statistical analysis was performed with one-way ANOVA test (*p* < 0.05) followed by Tukey’s posthoc analysis (B) or unpaired *t*-test (C–E). *p < 0.033, **p < 0.002, and ***p < 0.001.

Compound **11** demonstrated a potent NRF2 induction capacity; therefore, we evaluated its ability to upregulate the expression of NRF2-dependent genes. AREc32 cells treated with compound **11** significantly increased luciferase activity at 0.1, 1, 10, and 20 μM (Figure 5B) showing a CD value of 0.361 ± 0.074 μM, higher than other well-known NRF2 activators such as curcumin, resveratrol, or sulforaphane, the latter of which has been subjected to an intense clinical investigation.^69^ Then, we analyzed the expression levels of different NRF2-regulated genes, such as OS-induced growth inhibitor 1 (*OSGIN1*), *GCLM*, *NQO1*, and *HMOX1*. Compound **11** induced a clear and significant overexpression of all evaluated genes after 24 h of treatment with compound **11** at 10 μM concentration (Figure 5C). Expression levels of *GCLM*, *OSGIN1*, and *NQO1* were increased almost 2-fold, and *HMOX1* levels showed a 3-fold increase compared to untreated conditions. To confirm that this derivative exerts these effects through direct NRF2 activation, we employed WT and NRF2 KO Mouse embryonic fibroblast (MEF) cells. This direct effect has been clearly demonstrated for other inducers, such as sulforaphane in the same cells^21^ and for DMF in mice.^70^ Similarly, compound **11** increased the level of gene expression of NRF2-dependent *Osgin1*, *Nqo1*, and *Hmox*1 in WT-MEF cells (Figure 5D). In contrast, we were not able to increase their expression in NRF2 KO-MEF cells (Figure 5E), validating that compound **11** directly activates NRF2.

### 3.5 ADME Evaluation. Pharmacokinetics Analysis, In Vitro Microsomal Metabolism, and Solubility

Considering the encouraging complementary pharmacological profile of compound **11**, we further characterized its ADMET profile (Table 3) by evaluating its metabolic stability in liver microsomes (human, dog, and mouse) and its solubility at physiological pH (pH = 7.4). Importantly, compound **11** showed a good solubility of 196.5 mg/L; thus, we proceeded to evaluate its microsomal stability. Compound **11** was incubated with pulled liver microsomes (human, dog, or mouse) at 1 μM during 2 h, and samples were collected and processed at increasing timings. Finally, the remaining compound **11** was calculated by LC/MS. Compound **11** showed a high stability in human and dog liver microsomes with half-life values of 53.6 ± 0.6 and 49.9 ± 1.9 min, respectively. These values were correlated with low CL_int_ values in both species. Nonetheless, its stability was considerably lower in mouse liver microsomes with a half-life of 4.4 min and a high CL_int_ value. This result is in line with previous reports in which mouse liver microsomes exert higher metabolism toward tested compounds. However, lower metabolism in human liver microsomes is preferred in drug development as it is associated with lower drug–drug interactions and lower induction of drug-metabolizing enzymes induction, particularly those of the cytochrome P450 family. Moreover, low turnover in human liver microsomes is also related to higher and sustained plasma levels over time, facilitating its dosage.

**Table 3:** In Vitro Liver Microsome Stability and Solubility of Compound **11**

human		dog		mouse			
a*t*_1/2_	bCl_int_	*t*_1/2_	Cl_int_	*t*_1/2_	Cl_int_	sol. (mg/L)	
53.6 ± 0.6	24.9 ± 0.3	49.9 ± 1.9	26.8 ± 1.0	4.4 ± 0.1	304.0 ± 0.9	196.5 ± 19.6	

a min.

b l/min mg.

Preliminary in vitro stability results combined with a positive BBB crossing indicate a promising pharmacokinetic (PK) profile for compound **11**. Additionally, compound **11** did not exert any neurotoxicity in the neuroblastoma cell line SH-SY5Y with LD_50_ values higher than 100 μM. In this line, it did not show toxicity toward the AREc32 cell line or the BV2 microglial cell line, demonstrating a good preliminary safety profile.

Considering its positive profile, we evaluated its PK profile in C57BL/6 mice following a single intravenous (IV) administration at 10 mg/kg and orally (PO) at 50 mg/kg (Table 4). Following a single IV administration, compound **11** showed a moderate plasma clearance (54.3 mL/min/kg), lower than the normal liver blood flow in mice (90 mL/min/kg) and a moderate volume of distribution (1.24-fold higher than total body water, 0.7 L/kg), indicating an enrichment in body tissues. Finally, terminal elimination plasma half-life was 0.36 h, demonstrating a moderately fast elimination, as predicted by mice liver microsomes data. Interestingly, PO administration showed the highest plasma levels at the first sampling point (*t*_max_: 0.25 h), indicating a rapid absorption via the gastric intestinal tract with the corresponding peak plasma concentration (*C*_max_) of 1143.95 ng/mL. In this case, PO administration showed an increased half-life of 1.83 h and detectable plasma concentrations up to 8 h after oral administration. In these conditions, oral bioavailability was determined to be 11%, indicating a promising potential to be optimized via oral formulations.

**Table 4:** Pharmacokinetic Plasma Concentration–Time Profile of Compound **11** after Intravenous or Oral Administration in C57-BL/6 Mice

matrix	route	dose (mg/kg)	*T*_max_ (h)	a*C*_0_/*C*_max_ (ng/mL)	AUC_last_ (h ng/mL)	*T*_1/2_ (h)	CL (mL/min/kg)	*V*_ss_ (L/kg)	% *F*
plasma	IV	10		10,683.87	3179.82	0.36	54.30	0.87	
	PO	50	0.25	1143.95	1743.29	1.83			11

a Back extrapolated concentration in IV arm. IV, intravenous administration; PO, oral administration; *C*_max_, maximum concentration; *T*_max_, time to reach the maximum concentration; *T*_1/2_, elimination half-life; AUC, area under the curve; *V*_ss_, volume of distribution at steady state; Cl, clearance; *C*_max_, maximum drug concentration; and *F*, oral bioavailability.

## 4 Conclusions

Previously, we reported a novel multitarget 2-(1*H*-indol-3-yl)ethan-1-amine core which combined MAO-B selective inhibition and NRF2 induction activities that demonstrated neuroprotective capacity in in vitro and ex vivo PD models. Hit compound **5** also demonstrated anti-neuroinflammatory properties, reducing nitrite production and IL-1β expression in primary mixed glial cell cultures challenged with LPS. However, hit derivative 5 showed moderate MAO-B inhibitory potency combined with limited BBB permeability predictions, with *P*_e_ values in the threshold limit and, in some cases, significant cellular toxicity. Moreover, NRF2-related activity was achieved by the inclusion of an ester moiety, reducing bioavailability when used in vivo. The present study describes the hit-to-lead optimization program of our preliminary hit derivative 5 to improve its multitarget pharmacological profile together with its PK-ADME profile and develop a promising candidate for advanced PD preclinical models.

Our novel derivatives were designed following a 2-step optimization program including an SBVS approach. Based on docking calculations of derivative 5-MAO-B docking, we proposed **11** different indole core modifications in order to optimize its MAO-B inhibitory potency. Thereafter, **29** core substitution patterns were selected and combinatorially merged with indole cores to generate a virtual chemolibrary of 476 compounds obtained after being filtered by BBB crossing potential, secondary effects alerts, and PK parameters. All compounds were subjected to SBVS toward the MAO-B enzyme, resulting in two different core families synthesized.

Our novel compounds, and especially the *N*-(1*H*-indol-5-yl)aryl-acrylamide derivatives, showed an overall improved pharmacological profile compared to former 2-(1*H*-indol-3-yl)ethan-1-amine derivatives. In general, they showed an enhanced NRF2 induction potency, MAO-B potency, and selectivity, CNS permeability by passive diffusion, and a better neuroprotective profile in PD-related models linked to OS. Importantly, they also exhibited a reduced cell toxicity, and they were able to maintain the complementary antioxidant and anti-inflammatory properties displayed by previous compounds. Compared to hit compound **5**, compound **11** showed: (i) 14-fold more potent NRF2 activation; (ii) 4.7-fold more potent MAO-B inhibition, being at least 2.5-fold more selective than 5 toward the B isozyme; (iii) 1.4-fold more potent ROS scavenging activity; (iv) 1.6-fold increased potency for nitrite reduction in primary mixed glial cultures; (v) 3.6-fold increase in CNS permeability; (vi) neuroprotection values of 89.6 and 94.2% in the 6-OHDA and R/O models in SH-SY5Y, respectively, compared to 71.7 and 53.3% for compound **11**; (vii) 1.5-fold reduction at least in LD_50_ in SH-SY5Y.

In conclusion, the improved pharmacological profile of hit compound **11**, targeting key molecular pathways related to PD pathophysiology, positions it as a promising drug candidate for the development of a disease-modifying treatment for PD.

## 5 Experimental Section

### 5.1 Chemistry (General)

All reagents were commercial compounds with a high purity. Argon atmosphere was employed for all reactions. Triethylamine (Et_3_N) and dichloromethane (DCM) were dried by distillation over NaOH and CaH_2_, respectively. Acetonitrile (MeCN), tetrahydrofuran (THF), and dimethylformamide were purchased anhydrous. Analytical thin-layer chromatography was carried out on aluminum plates with a Merck Silicagel 60 F254 instrument and visualized by UV irradiation (254 nm). Flash column chromatography was performed with a Merck Kieselgel 60 (230–400 mesh). Proton nuclear magnetic resonance (^1^H NMR) spectra were recorded in dimethyl sulfoxide (DMSO) at room temperature in BRUKER AVANCE III HD-400, JEOL JNM-ECZ400R, Bruker DRX-500 or VARIAN system-500 instruments. The proton spectra were reported as follows: chemical shifts (δ) in ppm (number of protons, multiplicity, coupling constant *J* in Hz, assignment). Carbon nuclear magnetic resonance (^13^C NMR) spectra were recorded in DMSO-*d*_6_ at room temperature by using the same spectrometers at 100 or 125 MHz, respectively. Purity of the compounds was analyzed by high-performance liquid chromatography (HPLC), coupled with high-resolution mass spectrometry electrospray with positive mode detection for mass determination, using a mass spectrometer with a quadrupole time-of-flight analyzer (QTOF) model QSTAR pulsar I (Applied Biosystems). All compounds are >95% pure by HPLC analysis.

#### 5.1.1 General Procedure for the Synthesis of *N*-(1*H*-Indol-5-yl)aryl-acrylamide Derivatives (**1**)

A solution of the corresponding aryl-acrylic acid derivative (1.05 equiv), HATU (1.05 equiv), and Et_3_N (1.05 equiv) in DCM under argon was stirred at room temperature for 15 min. Then, it was added dropwise to a solution of 5-amino-1*H*-indole (1.00 equiv) in DCM at 0 °C. The resulting mixture was stirred at room temperature for 24 h. When completed, final products were purified by flash chromatography on silica gel using petroleum ether/DCM (0–100%) and DCM/MeOH mixtures (0–5%) as eluents to afford the corresponding *N*-(1*H*-indol-5-yl)cinnamamide derivative.

#### 5.1.2 (*E*)-*N*-(1*H*-Indol-5-yl)-3-(*o*-tolyl)acrylamide (**7**)

General procedure 1, 5-amino-1*H*-indole (50.0 mg, 0.378 mmol) in DCM (10 mL), (*E*)-3-(*o*-tolyl)acrylic acid (64.4 mg, 0.397 mmol), HATU (151.0 mg, 0.387 mmol), and Et_3_N (55 μL, 0.397 mmol) were added for 24 h, followed by flash chromatography on silica gel (petroleum ether/DCM 0–100%:MeOH 0–5%) to afford compound **7** as a yellow solid (90% yield). ^1^H NMR (400 MHz, DMSO): δ_H_ 11.03 (1H, s, NH), 10.02 (1H, s, NHCO), 8.03 (1H, d, *J* = 2.0 Hz, 4-H), 7.79 (1H, d, *J* = 15.5 Hz, 3′-H), 7.60 (1H, dd, *J* = 7.3, 2.6 Hz, 6-H), 7.38–7.23 (6H, m, 2-H, 7-H, 3″-H, 4″-H, 5″-H, 6″-H), 6.78 (1H, d, *J* = 15.5 Hz, 2′-H), 6.41–6.39 (1H, m, 3-H), 2.42 (3H, s, Ph–CH_3_). ^13^C NMR (100 MHz, DMSO): δ_C_ 163.03, 136.82, 136.61, 133.81, 132.79, 131.30, 130.77, 129.29, 127.53, 126.46, 126.13, 125.99, 124.13, 114.72, 111.30, 110.66, 101.18, 19.50. HPLC–HRMS (ES^+^) calcd mass for C_18_H_17_N_2_O [(M + H)^+^], 277.1335; found [(2M + H)^+^], 553.2596; purity >99.00% (HPLC).

#### 5.1.3 (*E*)-*N*-(1*H*-Indol-5-yl)-3-(*m*-tolyl)acrylamide (**8**)

General procedure 1, 5-amino-1*H*-indole (50.0 mg, 0.378 mmol) in DCM (10 mL), (*E*)-3-(*m*-tolyl)acrylic acid (64.3 mg, 0.397 mmol), HATU (151.0 mg, 0.387 mmol), and Et_3_N (55 μL, 0.397 mmol) were added, 24 h, followed by flash chromatography on silica gel (petroleum ether/DCM 0–100%:MeOH 0–5%) to afford compound **8** as a yellow solid (83% yield). ^1^H NMR (400 MHz, DMSO): δ_H_ 11.02 (1H, s, NH), 9.99 (1H, s, NHCO), 8.02 (1H, d, *J* = 1.8 Hz, 4-H), 7.51 (1H, d, *J* = 15.7 Hz, 3′-H), 7.46–7.26 (6H, m, 2-H, 6-H, 7-H, 2″-H, 5″-H, 6″-H), 7.22 (1H, *J* = 7.5 Hz, 4″-H), 6.86 (1H, d, *J* = 15.7 Hz, 2′-H), 6.43–6.37 (1H, m, 3-H), 2.35 (3H, s, 1H, Ph–CH_3_). ^13^C NMR (100 MHz, DMSO): δ_C_ 163.03, 139.18, 138.17, 134.93, 132.77, 131.34, 130.26, 128.93, 128.10, 127.52, 125.98, 124.87, 122.80, 114.67, 111.30, 110.58, 101.18, 20.97. HPLC–HRMS (ES^+^) calcd mass for C_18_H_17_N_2_O [(M + H)^+^], 277.1335; found [(M + H)^+^], 277.1333; found [(2M + H)^+^] 553.2594; purity >99.00% (HPLC).

#### 5.1.4 (*E*)-3-(2-Chlorophenyl)-*N*-(1*H*-indol-5-yl)acrylamide (**9**)

General procedure 1, 5-amino-1*H*-indole (50.0 mg, 0.378 mmol) in DCM (10 mL), (*E*)-3-(2-chlorophenyl)acrylic acid (72.5 mg, 0.397 mmol), HATU (151.0 mg, 0.387 mmol), and Et_3_N (55 μL, 0.397 mmol) were added, 24 h, followed by flash chromatography on silica gel (petroleum ether/DCM 0–100%:MeOH 0–5%) to afford compound **9** as a yellow solid (95% yield). ^1^H NMR (400 MHz, DMSO): δ_H_ 11.05 (1H, s, NH), 10.14 (1H, s, NHCO), 8.05 (1H, d, *J* = 2.1 Hz, 4-H), 7.87 (1H, d, *J* = 15.6 Hz, 3′-H), 7.82–7.73 (1H, m, 6-H), 7.59–7.50 (1H, m, 7-H), 7.49–7.36 (2H, m, 3″-H, 6″-H), 7.37–7.29 (3H, m, 2-H, 4″-H, 5″-H), 6.94 (1H, d, *J* = 15.6 Hz, 2′-H), 6.44–6.38 (1H, m, 3-H). ^13^C NMR (100 MHz, DMSO): δ_C_ 162.46, 134.36, 133.36, 132.88, 133.80, 131.13, 131.00, 130.08, 127.87, 127.70, 127.61, 127.53, 126.06, 114.71, 111.35, 110.74, 101.22. HPLC–HRMS (ES^+^) calcd mass for C_17_H_13_ClN_2_O [(M + H)^+^], 297.0789; found [(M + H)^+^], 297.0788; calcd for [(M + Na)^+^], 319.0609; found [(M + Na)^+^], 319.0609; purity 99.06% (HPLC).

#### 5.1.5 (*E*)-3-(2,6-Dichlorophenyl)-*N*-(1*H*-indol-5-yl)acrylamide (**10**)

General procedure 1, 5-amino-1*H*-indole (50.0 mg, 0.378 mmol) in DCM (10 mL), (*E*)-3-(2,6-dichlorophenyl)acrylic acid (86.2 mg, 0.397 mmol), HATU (151.0 mg, 0.387 mmol), and Et_3_N (55 μL, 0.397 mmol) were added, 24 h, followed by flash chromatography on silica gel (petroleum ether/DCM 0–100%:MeOH 0–5%) to afford compound **10** as a yellow solid (72% yield). ^1^H NMR (400 MHz, DMSO): δ_H_ 11.06 (1H, s, NH), 10.22 (1H, s, NHCO), 8.06 (1H, d, *J* = 2.1 Hz, 4-H), 7.63 (1H, d, *J* = 16.0 Hz, 3′-H), 7.57 (2H, d, *J* = 8.2 Hz, 3″-H, 5″-H), 7.42–7.30 (4H, m, 2-H, 6-H, 7-H, 4″-H), 6.98 (1H, d, *J* = 16.0 Hz, 2′-H), 6.43–6.39 (1H, m, 3-H). ^13^C NMR (100 MHz, DMSO): δ_C_ 162.00, 133.89, 132.93, 132.57, 132.10, 131.08, 131.01, 130.51, 129.20, 127.52, 126.08, 114.69, 111.37, 110.80, 101.24. HPLC–HRMS (ES^+^) calcd mass for C_17_H_13_Cl_2_N_2_O [(M + H)^+^], 331.0399; found [(M + H)^+^], 331.0394; purity >99% (HPLC).

#### 5.1.6 (*E*)-3-(2-Fluorophenyl)-*N*-(1*H*-indol-5-yl)acrylamide (**11**)

General procedure 1, 5-amino-1*H*-indole (50.0 mg, 0.378 mmol) in DCM (10 mL), (*E*)-3-(2-fluorophenyl)acrylic acid (66.0 mg, 0.397 mmol), HATU (151.0 mg, 0.387 mmol), and Et_3_N (55 μL, 0.397 mmol) were added, 24 h, followed by flash chromatography on silica gel (petroleum ether/DCM 0–100%:MeOH 0–5%) to afford compound **11** as a yellow solid (88% yield). ^1^H NMR (400 MHz, DMSO): δ_H_ 11.03 (1H, s, NH), 10.12 (1H, s, NHCO), 8.04 (1H, d, *J* = 2.0 Hz, 4-H), 7.71 (1H, td, *J* = 7.8, 1.8 Hz, 5″-H), 7.63 (1H, d, *J* = 15.9 Hz, 3′-H), 7.50–7.40 (1H, m, 6″-H), 7.39–7.24 (5H, m, 2-H, 6-H, 7-H, 3″-H, 4″-H), 6.99 (1H, d, *J* = 15.9 Hz, 2′-H), 6.44–6.38 (1H, m, 3-H). ^13^C NMR (100 MHz, DMSO): δ_C_ 162.74, 160.55 (d, *J* = 250.5 Hz), 132.85, 131.59 (d, *J* = 2.5 Hz), 131.37 (d, *J* = 8.8 Hz), 131.22, 129.38 (d, *J* = 3.4 Hz), 127.52, 126.01, 125.81 (d, *J* = 6.3 Hz), 125.08 (d, *J* = 3.4 Hz), 122.62 (d, *J* = 11.6 Hz), 116.18 (d, *J* = 21.7 Hz), 114.72, 111.32, 110.72, 101.21. ^19^F NMR (375 MHz, DMSO): δ_F_ −116.75. HPLC–HRMS (ES^+^) calcd mass for C_17_H_14_FN_2_O [(M + H)^+^], 281.1085; found [(M + H)^+^], 281.1084; [(2M + H)^+^], 561.2092; purity >99% (HPLC).

#### 5.1.7 (*E*)-3-(3-Fluorophenyl)-*N*-(1*H*-indol-5-yl)acrylamide (**12**)

General procedure 1, 5-amino-1*H*-indole (50.0 mg, 0.378 mmol) in DCM (10 mL), (*E*)-3-(3-fluorophenyl)acrylic acid (66.0 mg, 0.397 mmol), HATU (151.0 mg, 0.387 mmol), and Et_3_N (55 μL, 0.397 mmol) were added, 24 h, followed by flash chromatography on silica gel (petroleum ether/DCM 0–100%:MeOH 0–5%) to afford compound **12** as a yellow solid (85% yield). ^1^H NMR (400 MHz, DMSO): δ_H_ 11.03 (1H, s, NH), 10.04 (1H, s, NHCO), 8.02 (1H, d, *J* = 2.0 Hz, 4-H), 7.56 (1H, d, *J* = 15.6 Hz, 3′-H), 7.53–7.41 (3H, m, 3H, 6-H, 5″-H, 6″-H), 7.39–7.27 (4H, m, 2-H, 7-H, 2″-H, 4″-H), 6.90 (1H, d, *J* = 15.6 Hz, 2′-H), 6.43–6.37 (1H, m, 3-H). ^13^C NMR (100 MHz, DMSO): δ_C_ 162.67, 162.50 (d, *J* = 243.8 Hz) 137.79, 137.62 (d, *J* = 7.9 Hz), 132.83, 131.19, 130.99 (d, *J* = 8.5 Hz), 127.51, 126.02, 124.55, 123.68 (d, *J* = 2.2 Hz), 116.19 (d, *J* = 21.0 Hz), 114.70, 114.05 (d, *J* = 21.6 Hz), 111.32, 110.68, 101.19. ^19^F NMR (375 MHz, DMSO): δ_F_ −113.85. HPLC–HRMS (ES^+^) calcd mass for C_17_H_14_FN_2_O [(M + H)^+^], 281.1085; found [(M + H)^+^], 281.1084; [(2M + H)^+^], 561.2093; purity >99% (HPLC).

#### 5.1.8 (*E*)-3-(4-Fluorophenyl)-*N*-(1*H*-indol-5-yl)acrylamide (**13**)

General procedure 1, 5-amino-1*H*-indole (50.0 mg, 0.378 mmol) in DCM (10 mL), (*E*)-3-(4-fluorophenyl)acrylic acid (66.0 mg, 0.397 mmol), HATU (151.0 mg, 0.387 mmol), and Et_3_N (55 μL, 0.397 mmol) were added, 24 h, followed by flash chromatography on silica gel (petroleum ether/DCM 0–100%:MeOH 0–5%) to afford compound **13** as a yellow solid (91% yield). ^1^H NMR (400 MHz, DMSO): δ_H_ 11.02 (1H, s, NH), 10.00 (1H, s, NHCO), 8.02 (1H, d, *J* = 2.1 Hz, 4-H), 7.68 (2H, dd, *J* = 8.9, 5.5 Hz, 2″-H, 6″-H), 7.56 (1H, d, *J* = 15.9 Hz, 3′-H), 7.38–7.23 (5H, m, 2-H, 6-H, 7-H, 3″-H, 5″-H), 6.81 (1H, d, *J* = 15.7 Hz, 2′-H), 6.43–6.37 (1H, m, 3-H). ^13^C NMR (100 MHz, DMSO): δ_C_ 162.93, 162.74 (d, *J* = 247.1 Hz) 132.79, 131.64 (d, *J* = 3.2 Hz), 131.28, 129.76 (d, *J* = 8.5 Hz), 127.52, 125.98, 124.98, 122.85, 115.99 (d, *J* = 21.8 Hz), 114.72,111.29, 110.66, 101.17. ^19^F NMR (375 MHz, DMSO): δ_F_ −112.46. HPLC–HRMS (ES^+^) calcd mass for C_17_H_14_FN_2_O [(M + H)^+^], 281.1085; found [(M + H)^+^], 281.1087; [(2M + H)^+^], 561.2091; purity >99% (HPLC).

#### 5.1.9 (*E*)-3-(2,3-Difluorophenyl)-*N*-(1*H*-indol-5-yl)acrylamide (**14**)

General procedure 1, 5-amino-1*H*-indole (50.0 mg, 0.378 mmol) in DCM (10 mL), (*E*)-3-(2,3-difluorophenyl)acrylic acid (73.1 mg, 0.397 mmol), HATU (151.0 mg, 0.387 mmol), and Et_3_N (55 μL, 0.397 mmol) were added, 24 h, followed by flash chromatography on silica gel (petroleum ether/DCM 0–100%:MeOH 0–5%) to afford compound **14** as a yellow solid (79% yield). ^1^H NMR (400 MHz, DMSO): δ_H_ 11.04 (1H, s, NH), 10.17 (1H, s, NHCO), 8.04 (1H, d, *J* = 2.1 Hz, 4-H), 7.61 (1H, d, *J* = 15.9 Hz, 3′-H), 7.56–7.42 (2H, m, 5-H″, 6″-H), 7.41–7.24 (4H, m, 2-H, 6-H, 7-H, 4″-H), 7.02 (1H, d, *J* = 15.9 Hz, 2′-H), 6.45–6.35 (1H, m, 3-H). ^13^C NMR (100 MHz, DMSO): δ_C_ 162.37, 149.44 (dd, *J* = 2445.8, 12.0 Hz), 147.01 (dd, *J* = 251.4, 13.1 Hz), 132.89, 131.10, 130.52, 127.51, 127.19 (d, *J* = 6.5 Hz), 126.05, 125.29 (dd, *J* = 6.1, 4.5 Hz), 124.94 (d, *J* = 8.3 Hz), 124.55, 118.08 (d, *J* = 17.1 Hz), 114.70, 111.34, 110.75, 101.22. ^19^F NMR (375 MHz, DMSO): δ_F_ −138.42 to −138.75 (m), −141.82 to −142.21 (m). HPLC–HRMS (ES^+^) calcd mass for C_17_H_13_F_2_N_2_O [(M + H)^+^], 299.0990; found [(M + H)^+^], 299.0992; [(2M + H)^+^], 597.1906; purity 97.58% (HPLC).

#### 5.1.10 (*E*)-3-(2,4-Difluorophenyl)-*N*-(1*H*-indol-5-yl)acrylamide (**15**)

General procedure 1, 5-amino-1*H*-indole (50.0 mg, 0.378 mmol) in DCM (10 mL), (*E*)-3-(2,4-difluorophenyl)acrylic acid (73.1 mg, 0.397 mmol), HATU (151.0 mg, 0.387 mmol), and Et_3_N (55 μL, 0.397 mmol) were added, 24 h, followed by flash chromatography on silica gel (petroleum ether/DCM 0–100%:MeOH 0–5%) to afford compound **15** as a yellow solid (80% yield). ^1^H NMR (400 MHz, DMSO): δ_H_ 11.03 (1H, s, NH), 10.10 (1H, s, NHCO), 8.03 (1H, d, *J* = 2.0 Hz, 4-H), 7.77 (1H, td, *J* = 8.8, 6.5 Hz, 6″-H), 7.57 (1H, d, *J* = 15.9 Hz, 3′-H), 7.44–7.26 (4H, m, 2-H, 6-H, 7-H, 3″-H), 7.20 (1H, td, *J* = 8.1, 2.9 Hz, 5″-H), 6.94 (1H, d, *J* = 15.9 Hz, 2′-H), 6.44–6.34 (1H, m, 3-H). ^13^C NMR (100 MHz, DMSO): δ_C_ 162.69 (dd, *J* = 250.0, 13.0 Hz), 162.68, 160.75 (dd, *J* = 253.3, 12.5 Hz), 132.84, 131.18, 130.97 (dd, *J* = 10.1, 4.7 Hz), 130.80, 127.50, 126.01, 125.52 (d, *J* = 6.7 Hz), 119.47 (dd, *J* = 11.8, 3.8 Hz), 114.70, 112.48 (dd, *J* = 21.6, 3.5 Hz), 111.31, 110.71, 104.75 (t, *J* = 26.2 Hz), 101.19 ^19^F NMR (375 MHz, DMSO): δ_F_ −108.74 (d, *J* = 8.9 Hz), −112.02 (d, *J* = 8.9 Hz). HPLC–HRMS (ES^+^) calcd mass for C_17_H_13_F_2_N_2_O [(M + H)^+^], 299.0990; found [(M + H)^+^], 299.0985; [(2M + H)^+^], 597.1903; purity >99% (HPLC).

#### 5.1.11 (*E*)-3-(2,5-Difluorophenyl)-*N*-(1*H*-indol-5-yl)acrylamide (**16**)

General procedure 1, 5-amino-1*H*-indole (50.0 mg, 0.378 mmol) in DCM (10 mL), (*E*)-3-(2,5-difluorophenyl)acrylic acid (73.1 mg, 0.397 mmol), HATU (151.0 mg, 0.387 mmol), and Et_3_N (55 μL, 0.397 mmol) were added, 24 h, followed by flash chromatography on silica gel (petroleum ether/DCM 0–100%:MeOH 0–5%) to afford compound **16** as a yellow solid (82% yield). ^1^H NMR (400 MHz, DMSO): δ_H_ 11.03 (1H, s, NH), 10.14 (1H, s, NHCO), 8.03 (1H, d, *J* = 2.1 Hz, 4-H), 7.63–7.50 (2H, m, 3′-H, 6-H), 7.42–7.24 (5H, m, 2-H, 7-H, 3″-H, 4″-H, 6″-H), 7.00 (1H, d, *J* = 15.9 Hz, 2′-H), 6.44–6.35 (1H, m, 3-H). ^13^C NMR (100 MHz, DMSO): δ_C_ 162.45, 158.33 (d, *J* = 240.1 Hz), 156.76 (d, *J* = 246.2 Hz), 132.89, 131.11, 130.70, 127.51, 127.32 (d, *J* = 6.9 Hz), 126.05, 124.23 (dd, *J* = 14.3, 8.4 Hz), 117.82 (dd, *J* = 25.4, 8.8 Hz), 117.53 (dd, *J* = 25.6, 8.5 Hz), 115.39 (dd, *J* = 24.8, 3.7 Hz), 114.72, 111.33, 110.77, 101.22. ^19^F NMR (375 MHz, DMSO): δ_F_ −117.89 to −118.16 (m), −120.57 to −120.89 (m). HPLC–HRMS (ES^+^) calcd mass for C_17_H_13_F_2_N_2_O [(M + H)^+^], 299.0990; found [(M + H)^+^], 299.0990; [(2M + H)^+^], 597.1902; purity 97.84% (HPLC).

#### 5.1.12 (*E*)-3-(2,6-Difluorophenyl)-*N*-(1*H*-indol-5-yl)acrylamide (**17**)

General procedure 1, 5-amino-1*H*-indole (50.0 mg, 0.378 mmol) in DCM (10 mL), (*E*)-3-(2,6-difluorophenyl)acrylic acid (73.1 mg, 0.397 mmol), HATU (151.0 mg, 0.387 mmol), and Et_3_N (55 μL, 0.397 mmol) were added, 24 h, followed by flash chromatography on silica gel (petroleum ether/DCM 0–100%:MeOH 0–5%) to afford compound **17** as a yellow solid (80% yield). ^1^H NMR (400 MHz, DMSO): δ_H_ 11.04 (1H, s, NH), 10.22 1H, s, NHCO), 8.05 (1H, d, *J* = 1.8 Hz, 4-H), 7.59 (1H, d, *J* = 16.0 Hz, 3′-H), 7.54–7.43 (1H, m, 4″-H), 7.39–7.29 (3H, m, 2-H, 6-H, 7-H), 7.23 (2H, t, *J* = 8.7 Hz, 3″-H, 5″-H), 7.13 (1H, d, *J* = 16.0 Hz, 2′-H), 6.44–6.36 (1H, m, 3-H). ^13^C NMR (100 MHz, DMSO): δ_C_ 162.58, 160.81 (dd, *J* = 252.2, 6.9 Hz), 132.90, 131.38 (t, *J* = 10.9 Hz), 131.16, 128.83 (t, *J* = 7.7 Hz), 127.52, 126.04, 124.82, 114.70, 112.30 (dd, *J* = 24.2, 4.6 Hz), 112.00 (d, *J* = 15.6 Hz), 111.34, 110.75, 101.24. ^19^F NMR (375 MHz, DMSO): δ_F_ −112.70. HPLC–HRMS (ES^+^) calcd mass for C_17_H_12_F_2_N_2_O [(M + H)^+^], 299.0990; found [(M + H)^+^], 299.0991; calcd for [(M + Na)^+^], 321.0810; found [(M + Na)^+^], 321.0810; purity 98.89% (HPLC).

#### 5.1.13 (*E*)-3-(3,4-Difluorophenyl)-*N*-(1*H*-indol-5-yl)acrylamide (**18**)

General procedure 1, 5-amino-1*H*-indole (50.0 mg, 0.378 mmol) in DCM (10 mL), (*E*)-3-(3,4-difluorophenyl)acrylic acid (73.1 mg, 0.397 mmol), HATU (151.0 mg, 0.387 mmol), and Et_3_N (55 μL, 0.397 mmol) were added, 24 h, followed by flash chromatography on silica gel (petroleum ether/DCM 0–100%:MeOH 0–5%) to afford compound **18** as a yellow solid (65% yield). ^1^H NMR (400 MHz, DMSO): δ_H_ 11.02 (1H, s, NH), 10.03 (1H, s, NHCO), 8.01 (1H, d, *J* = 1.9 Hz, 4-H), 7.76–7.66 (1H, m, 6″-H), 7.58–7.46 (3H, m, 3′-H, 2″-H, 5″-H), 7.38–7.26 (3H, m, 2-H, 6-H, 7-H), 6.84 (1H, d, *J* = 15.7 Hz, 2′-H), 6.43–6.37 (1H, m, 3-H). ^13^C NMR (100 MHz, DMSO): δ_C_ 162.64, 150.29 (dd, *J* = 248.3, 12.1 Hz), 148.48 (dd, *J* = 246.5, 12.6 Hz), 136.96, 132.99 (dd, *J* = 6.5, 4.3 Hz), 132.84, 131.18, 127.52, 126.03, 124.65 (dd, *J* = 6.8, 4.4 Hz), 124.40, 118.15 (d, *J* = 17.4 Hz), 116.39 (d, *J* = 17.3 Hz), 114.73, 111.32, 110.72, 101.19. ^19^F NMR (375 MHz, DMSO): δ_F_ −133.26 (d, *J* = 21.8 Hz), −134.25 (d, *J* = 22.5 Hz). HPLC–HRMS (ES^+^) calcd mass for C_17_H_13_F_2_N_2_O [(M + H)^+^], 299.0990; found [(M + H)^+^], 299.0990; [(2M + H)^+^], 597.1902; purity >99% (HPLC).

#### 5.1.14 (*E*)-3-(3,5-Difluorophenyl)-*N*-(1*H*-indol-5-yl)acrylamide (**19**)

General procedure 1, 5-amino-1*H*-indole (50.0 mg, 0.378 mmol) in DCM (10 mL), (*E*)-3-(3,5-difluorophenyl)acrylic acid (73.1 mg, 0.397 mmol), HATU (151.0 mg, 0.387 mmol), and Et_3_N (55 μL, 0.397 mmol) were added, 24 h, followed by flash chromatography on silica gel (petroleum ether/DCM 0–100%:MeOH 0–5%) to afford compound **19** as a yellow solid (70% yield). ^1^H NMR (400 MHz, DMSO): δ_H_ 11.04 (1H, s, NH), 10.07 (1H, s, NHCO), 8.02 (1H, d, *J* = 2.2 Hz, 4-H), 7.55 (1H, d, *J* = 15.8 Hz, 3′-H), 7.41–7.24 (6H, m, 2-H, 6-H, 7-H, 2″-H, 4″-H, 6″-H), 6.93 (1H, d, *J* = 15.8 Hz, 2′-H), 6.44–6.33 (1H, m, 3-H). ^13^C NMR (100 MHz, DMSO): δ_C_ 162.71(dd, *J* = 246.1, 13.4 Hz), 162.40, 138.95 (t, *J* = 9.8 Hz), 136.72, 132.89, 131.10, 127.52, 126.06, 126.00, 114.72, 111.35, 110.76, 110.54 (dd, *J* = 19.5, 6.8 Hz), 104.63 (t, *J* = 26.0 Hz), 101.22. ^19^F NMR (375 MHz, DMSO): δ_F_ −110.45. HPLC–HRMS (ES^+^) calcd mass for C_17_H_13_F_2_N_2_O [(M + H)^+^], 299.0990; found [(M + H)^+^], 299.0994; [(2M + H)^+^], 597.1902; purity >99% (HPLC).

#### 5.1.15 (*E*)-*N*-(1*H*-Indol-5-yl)-3-(2-(trifluoromethyl)phenyl)acrylamide (**20**)

General procedure 1, 5-amino-1*H*-indole (50.0 mg, 0.378 mmol) in DCM (10 mL), (*E*)-3-(2-(trifluoromethyl)phenyl)acrylic acid (86.2 mg, 0.397 mmol), HATU (151.0 mg, 0.387 mmol), and Et_3_N (55 μL, 0.397 mmol) were added, 24 h, followed by flash chromatography on silica gel (petroleum ether/DCM 0–100%:MeOH 0–5%) to afford compound **20** as a yellow solid (95% yield). ^1^H NMR (400 MHz, DMSO): δ_H_ 11.05 (1H, s, NH), 10.16 (1H, s, NHCO), 8.03 (1H, d, *J* = 1.8 Hz, 4-H), 7.90 (1H, d, *J* = 7.8 Hz, 3″-H), 7.87–7.74 (3H, m, 3′-H, 5″-H, 6″-H), 7.62 (1H, t, *J* = 7.6 Hz, 4″-H), 7.39–7.28 (3H, m, 2-H, 6-H, 7-H), 6.94 (1H, d, *J* = 15.3 Hz, 2′-H), 6.44–6.38 (1H, m, 3-H). ^13^C NMR (100 MHz, DMSO): δ_C_ 162.14, 133.82, 133.55, 133.17, 132.91, 131.03, 129.63, 127.84, 127.54 (d, *J* = 2.1 Hz), 126.99, 126.70, 126.21 (q, *J* = 5.8 Hz), 126.08, 124.25 (q, *J* = 275.3 Hz), 114.68, 111.36, 110.76, 101.22. ^19^F NMR (375 MHz, DMSO): δ_F_ −58.71. HPLC–HRMS (ES^+^) calcd mass for C_18_H_14_F_3_N_2_O [(M + H)^+^], 331.1053; found [(M + H)^+^], 331.1051; [(2M + H)^+^], 661.2022; purity >99% (HPLC).

#### 5.1.16 (*E*)-*N*-(1*H*-Indol-5-yl)-3-(3-(trifluoromethyl)phenyl)acrylamide (**21**)

General procedure 1, 5-amino-1*H*-indole (50.0 mg, 0.378 mmol) in DCM (10 mL), (*E*)-3-(3-(trifluoromethyl)phenyl)acrylic acid (86.2 mg, 0.397 mmol), HATU (151.0 mg, 0.387 mmol), and Et_3_N (55 μL, 0.397 mmol) were added, 24 h, followed by flash chromatography on silica gel (petroleum ether/DCM 0–100%:MeOH 0–5%) to afford compound **21** as a yellow solid (78% yield). ^1^H NMR (400 MHz, DMSO): δ_H_ 11.03 (1H, s, NH), 10.06 (1H, s, NHCO), 8.04 (1H, d, *J* = 2.1 Hz, 4-H), 7.98 (1H, s, 2″-H), 7.93 (1H, d, *J* = 7.8 Hz, 4″-H), 7.75 (1H, d, *J* = 7.8 Hz, 6″-H), 7.69 (1H, t, *J* = 7.8 Hz, 5″-H), 7.65 (1H, d, *J* = 15.7 Hz, 3′-H), 7.39–7.27 (3H, m, 2-H, 6-H, 7-H), 7.00 (1H, d, *J* = 15.7 Hz, 2′-H), 6.44–6.37 (1H, m, 3-H). ^13^C NMR (100 MHz, DMSO): δ_C_ 162.55, 137.35, 136.21, 133.85, 131.45, 131.20, 130.16, 129.78 (q, *J* = 31.2 Hz), 127.53, 126.04, 125.79 (q, *J* = 3.9 Hz), 125.16, 124.09 (q, *J* = 272.6 Hz), 123.84 (q, *J* = 4.6 Hz), 114.64, 111.35, 111.63, 101.20. ^19^F NMR (375 MHz, DMSO): δ_F_ −61.17. HPLC–HRMS (ES^+^) calcd mass for C_18_H_14_F_3_N_2_O [(M + H)^+^], 331.1053; found [(M + H)^+^], 331.1050; [(2M + H)^+^], 661.2027; purity >99% (HPLC).

#### 5.1.17 2,2,2-Trifluoro-1-(5-nitro-1*H*-indol-3-yl)ethan-1-one (**23**)

To a solution of 5-nitro-1*H*-indole (5.00 g, 30.84 mmol) in dimethylformamide, 2,2,2-trifluoroacetic anhydride (6.53 mL, 46.26 mmol) was added dropwise at 0 °C. The resulting solution was stirred at room temperature for 48 h. The mixture was poured into ice-water and filtered. The filter cake was washed with water and concentrated under vacuum to yield the desired compound **23** as a white solid (100% yield). No further purification was needed. ^1^H NMR (500 MHz, DMSO): δ_H_ 13.25 (1H, s, NH), 9.00 (1H, d, *J* = 2.2 Hz, 4-H), 8.78 (1H, d, *J* = 1.8 Hz, 2-H), 8.24 (1H, dd, *J* = 8.9, 2.3 Hz, 6-H), 7.80 (1H, d, *J* = 8.9 Hz, 7-H). ^13^C NMR (125 MHz, DMSO): δ_C_ 174.94 (q, *J* = 34.9 Hz), 144.20, 141.52 (q, *J* = 4.5 Hz), 140.41, 125.75, 120.18, 118.13 (q, *J* = 291.4 Hz), 117.65, 114.42, 110.46.

#### 5.1.18 5-Nitro-1*H*-indole-3-carboxylic Acid (**24**)

A solution of 2,2,2-trifluoro-1-(5-nitro-1*H*-indol-3-yl)ethan-1-one (4.00 g, 15.50 mmol) in 40% aqueous NaOH solution was stirred at 60 °C for 3 h. Then, the reaction was acidified by adding 1 N aqueous HCl solution at 0 °C until the pH of the solution drops below 3. The mixture was filtered, and the filter cake was washed with water and concentrated under vacuum to yield the desired compound **24** as a white solid (100% yield). No further purification was needed. ^1^H NMR (500 MHz, DMSO): δ_H_ 12.46 (2H, s_br_, NH, and COOH), 8.89 (1H, s, 4-H), 8.27 (1H, s, 2-H), 8.08 (1H, dd, *J* = 9.0, 2.3 Hz, 6-H), 7.66 (1H, dd, *J* = 9.0, 3.4 Hz, 7-H). ^13^C NMR (125 MHz, DMSO): δ_C_: 165.62, 142.61, 140.11, 136.24, 125.82, 120.10, 117.98, 113.56, 109.89.

#### 5.1.19 5-Nitro-1*H*-indole-3-carboxamide (**25**)

To a solution of 5-nitro-1*H*-indole-3-carboxylic acid (**24**, 1.00 g, 4.85 mmol) in dimethylformamide under argon, HATU (2.21 g, 5.82 mmol), Et_3_N (0.94 mL, 6.79 mmol), and NH_4_Cl (311 mg, 5.82 mmol) were added at 0 °C. The resulting solution was stirred at room temperature for 72 h. Then, the reaction was extracted with AcOEt and washed with a 10% aqueous LiCl solution. The organic layers were dried in MgSO_4_ and filtered. Final products were purified by flash chromatography on silica gel using petroleum ether/EtOAc (0–100%) and EtOAc/MeOH mixtures (0–3%) as eluents to afford to yield the desired compound **25** as a yellow solid (60% yield). ^1^H NMR (500 MHz, DMSO): δ_H_ 12.15 (1H, s, NH), 9.08 (1H, d, *J* = 2.3 Hz, 4-H), 8.26 (1H, s, 2-H), 8.05 (1H, dd, *J* = 9.0, 2.4 Hz, 6-H), 7.62 (1H, d, *J* = 9.0 Hz, 7-H), 7.08 (2H, s_br_, *NH*_2_CO). ^13^C NMR (125 MHz, DMSO): δ_C_: 165.99, 142.22, 139.75, 132.17, 126.24, 118.40, 117.69, 112.99, 112.95.

#### 5.1.20 5-Amino-1*H*-indole-3-carboxamide (**26**)

A solution of 5-nitro-1*H*-indole-3-carboxamide (930 mg) in EtOH was added to a suspension of 10% palladium on activated carbon (500 mg, 10% mol) in the same solvent. The air was removed under vacuum, and hydrogen was introduced into the flask at 1 atm. When completed, the reaction mixture was filtered over Celite, and the solvent was removed, affording the desired compound **26** as a purple solid (80% yield). No further purification was needed. ^1^H NMR (500 MHz, DMSO): δ_H_ 10.29 (1H, s, NH), 7.89 (1H, s, 2-H), 7.02 (1H, d, *J* = 8.4 Hz, 7-H), 6.94 (1H, d, *J* = 2.2 Hz, 4-H), 6.64 (2H, s_br_, *NH*_2_CO), 6.46 (1H, dd, *J* = 8.4, 2.2 Hz, 6-H), 4.54 (2H, s_br_, *NH*_2_). ^13^C NMR (125 MHz, DMSO): δ_C_ 169.40, 141.04, 130.57, 128.49, 122.77, 112.24, 111.90, 110.75, 101.98.

#### 5.1.21 General Procedure for the Synthesis of *N*-(1*H*-Indol-3-carboxamide-5-yl)aryl-acrylamide Derivatives (**2**)

A solution of the corresponding cinnamic acid derivative (1.20 equiv), HATU (1.20 equiv), and Et_3_N (1.20 equiv) in DCM under argon was stirred at room temperature for 15 min. Then, it was added dropwise to a solution of 5-amino-1*H*-indole-3-carboxamide (1.00 equiv) in DCM at 0 °C. The resulting mixture was stirred at room temperature for 24 h. When completed, final products were purified by flash chromatography on silica gel using petroleum ether: EtOAc (0–100%) and EtOAc/MeOH mixtures (0–3%) as eluents to afford the corresponding 5-cinnamamido-1*H*-indole-3-carboxamide derivative.

#### 5.1.22 (*E*)-5-(3-(*o*-Tolyl)acrylamido)-1*H*-indole-3-carboxamide (**27**)

General procedure 2, 5-amino-1*H*-indole-3-carboxamide (30.0 mg, 0.171 mmol) in DCM (8 mL), (*E*)-3-(*o*-tolyl)acrylic acid (33.0 mg, 0.205 mmol), HATU (78.0 mg, 0.205 mmol), and Et_3_N (29 μL, 0.205 mmol) were added, 24 h, followed by flash chromatography on silica gel (petroleum ether/EtOAc 0–100%:MeOH 0–3%) to afford compound **27** as a yellow solid (75% yield). ^1^H NMR (400 MHz, DMSO): δ_H_ 11.48 (1H, d, *J* = 2.9 Hz, NH), 10.15 (1H, s, NHCO), 8.41 (1H, d, *J* = 2.1 Hz, 4-H), 8.01 (1H, d, *J* = 2.9 Hz, 2-H), 7.79 (1H, d, *J* = 15.5 Hz, 3′H), 7.68 (1H, dd, *J* = 8.8, 2.1 Hz, 6-H), 7.58 (1H, dd, *J* = 7.4, 1.9 Hz, 6″-H), 7.39–7.23 (4H, m, 3″-H, 4″-H, 5″-H, NH_2a_CO), 7.37 (1H, d, *J* = 8.7 Hz, 7-H), 6.79 (1H, d, *J* = 15.6 Hz, 2′-H), 6.77 (1H, s_br_, NH_2b_CO), 2.42 (3H, s, Ph–CH_3_). ^13^C NMR (100 MHz, DMSO): δ_C_ 166.52, 163.04, 136.80, 136.70, 133.78, 133.04, 132.94, 132.76, 130.75, 129.27, 128.89, 126.48, 126.04, 124.10, 115.40, 111.82, 111.53, 110.34, 19.48. HPLC–HRMS (ES^+^) calcd mass for C_19_H_17_N_3_O_2_ [(M + H)^+^], 320.1394; found [(M + H)^+^], 320.1390; calcd for [(M + Na)^+^], 342.1213; found [(M + Na)^+^], 342.1212; purity 98.89% (HPLC).

#### 5.1.23 (*E*)-5-(3-(*m*-Tolyl)acrylamido)-1*H*-indole-3-carboxamide (**28**)

General procedure 2, 5-amino-1*H*-indole-3-carboxamide (20.0 mg, 0.114 mmol) in DCM (8 mL), (*E*)-3-(*m*-tolyl)acrylic acid (22.0 mg, 0.137 mmol), HATU (52.0 mg, 0.137 mmol), and Et_3_N (19 μL, 0.137 mmol) were added, 24 h, followed by flash chromatography on silica gel (petroleum ether/EtOAc 0–100%:MeOH 0–3%) to afford compound **28** as a yellow solid (65% yield). ^1^H NMR (400 MHz, DMSO): δ_H_ 11.49 (1H, d, *J* = 3.1 Hz, NH), 10.13 (1H, s, NHCO), 8.41 (1H, d, *J* = 2.2 Hz, 4-H), 8.02 (1H, d, *J* = 2.9 Hz, 2-H), 7.67 (1H, dd, *J* = 8.7, 2.2 Hz, 6-H), 7.53 (1H, d, *J* = 15.6 Hz, 3′-H), 7.44–7.30 (5H, m, 7-H, 2″-H, 5″-H, 6″-H, NH_2a_CO), 7.22 (1H, d, *J* = 7.8 Hz, 4″-H), 6.87 (1H, d, *J* = 15.6 Hz, 2′-H), 6.80 (1H, s_br_, NH_2b_CO), 2.34 (3H, s, Ph–CH_3_). ^13^C NMR (100 MHz, DMSO): δ_C_ 166.97, 163.48, 139.68, 138.61, 135.36, 133.47, 133.24, 130.71, 129.37, 128.58, 126.91, 125.26, 123.25, 115.83, 115.10, 112.21, 111.97, 110.79, 21.41. HPLC–HRMS (ES^+^) calcd mass for C_19_H_17_N_3_O_2_, 320.1394; found [(M + H)^+^], 320.1388; calcd for [(M + Na)^+^], 342.1213; found [(M + Na)^+^], 342.1214; purity 98.97% (HPLC).

#### 5.1.24 (*E*)-5-(3-(2-Chlorophenyl)acrylamido)-1*H*-indole-3-carboxamide (**29**)

General procedure 2, 5-amino-1*H*-indole-3-carboxamide (30.0 mg, 0.171 mmol) in DCM (8 mL), (*E*)-3-(2-chlorophenyl)acrylic acid (38.0 mg, 0.205 mmol), HATU (78.0 mg, 0.205 mmol), and Et_3_N (29 μL, 0.205 mmol) were added, 24 h, followed by flash chromatography on silica gel (petroleum ether/EtOAc 0–100%:MeOH 0–3%) to afford compound **29** as a yellow solid (78% yield). ^1^H NMR (400 MHz, DMSO): δ_H_ 11.50 (d, *J* = 3.1 Hz, 1H NH), 10.26 (1H, s, NHCO), 8.41 (d, *J* = 2.2 Hz, 4-H), 8.02 (1H, d, *J* = 2.9 Hz, 2-H), 7.86 (1H, d, *J* = 15.7 Hz, 3′-H), 7.76 (1H, dd, *J* = 7.2, 2.4 Hz, 3″-H), 7.68 (1H, dd, *J* = 8.8, 2.1 Hz, 6-H), 7.59–7.51 (1H, m, 6″-H), 7.49–7.40 (2H, m, 4″-H, 5″-H), 7.39 (2H, s_br_, NH_2a_CO), 7.38 (1H, d, *J* = 8.8 Hz, 7-H), 6.94 (1H, d, *J* = 15.5 Hz, 2′-H), 6.79 (2H, s_br_, NH_2b_CO). ^13^C NMR (100 MHz, DMSO): δ_C_ 166.57, 162.50, 134.45, 133.37, 133.15, 132.78, 132.63, 131.02, 130.08, 129.00, 127.88, 127.61, 126.48, 126.05, 115.40, 111.89, 111.63, 110.37. HPLC–HRMS (ES^+^) calcd mass for C_18_H_14_ClN_3_O_2_, 340.0847; found [(M + H)^+^], 340.0852; calcd for [(M + Na)^+^], 362.0667; found [(M + Na)^+^], 362.0669; purity 97.80% (HPLC).

#### 5.1.25 (*E*)-5-(3-(2,6-Dichlorophenyl)acrylamido)-1*H*-indole-3-carboxamide (**30**)

General procedure 2, 5-amino-1*H*-indole-3-carboxamide (30.0 mg, 0.171 mmol) in DCM (8 mL), (*E*)-3-(2,6-dichlorophenyl)acrylic acid (44.5 mg, 0.205 mmol), HATU (78.0 mg, 0.205 mmol), and Et_3_N (29 μL, 0.205 mmol) were added, 24 h, followed by flash chromatography on silica gel (petroleum ether/EtOAc 0–100%:MeOH 0–3%) to afford compound **30** as a yellow solid (40% yield). ^1^H NMR (400 MHz, DMSO): δ_H_ 11.50 (1H, d, *J* = 2.9 Hz, NH), 10.34 (1H, s, NHCO), 8.42 (1H, d, *J* = 2.2 Hz, 4-H), 8.02 (1H, d, *J* = 2.9 Hz, 2-H), 7.69 (1H, dd, *J* = 8.8, 2.1 Hz, 6-H), 7.62 (1H, d, *J* = 16.0 Hz, 3′-H), 7.58 (2H, d, *J* = 8.1 Hz, 3″-H, 5″-H), 7.48–7.27 (3H, m, 7-H, 4″-H, NH_2a_CO), 6.99 (1H, d, *J* = 15.9 Hz, 2′-H), 6.79 (1H, s_br_, NH_2b_CO). ^13^C NMR (100 MHz, DMSO): δ_C_ 167.05, 162.57, 134.43, 133.72, 133.17, 133.02, 132.58, 131.61, 131.04, 129.72, 129.52, 126.99, 115.87, 112.46, 112.16, 110.91. HPLC–HRMS (ES^+^) calcd mass for C_18_H_13_Cl_2_N_3_O_2_, 374.0458; found [(M + H)^+^], 374.0455; calcd for [(M + Na)^+^], 396.0277; found [(M + Na)^+^], 396.0277; purity 95.20% (HPLC).

#### 5.1.26 (*E*)-5-(3-(2-Fluorophenyl)acrylamido)-1*H*-indole-3-carboxamide (**31**)

General procedure 2, 5-amino-1*H*-indole-3-carboxamide (30.0 mg, 0.171 mmol) in DCM (8 mL), (*E*)-3-(2-fluorophenyl)acrylic acid (34.0 mg, 0.205 mmol), HATU (78.0 mg, 0.205 mmol), and Et_3_N (29 μL, 0.205 mmol) were added, 24 h, followed by flash chromatography on silica gel (petroleum ether/EtOAc 0–100%:MeOH 0–3%) to afford compound **31** as a yellow solid (45% yield). ^1^H NMR (400 MHz, DMSO): δ_H_ 11.50 (1H, d, *J* = 2.9 Hz, NH), 10.26 (1H, s, NHCO), 8.41 (1H, d, *J* = 2.1 Hz, 4-H), 8.02 (1H, d, *J* = 2.9 Hz, 2-H), 7.74–7.66 (2H, m, 6-H, 6″-H), 7.63 (1H, d, *J* = 15.8 Hz, 3′-H), 7.50–7.41 (1H, m, 3″-H), 7.38 (1H, d, *J* = 8.8 Hz, 7-H), 7.37–7.24 (3H, m, 4″-H, 5″-H, NH_2a_CO), 7.00 (1H, d, *J* = 15.9 Hz, 2′-H), 6.80 (1H, s_br_, NH_2b_CO). ^13^C NMR (100 MHz, DMSO): δ_C_ 166.56, 162.77, 160.54 (d, *J* = 250.4 Hz), 133.11, 132.70, 131.60, 131.40 (d, *J* = 8.7 Hz), 129.30 (d, *J* = 2.9 Hz), 128.97, 126.47, 125.81 (d, *J* = 6.3 Hz), 125.09 (d, *J* = 3.5 Hz), 122.61 (d, *J* = 11.5 Hz), 116.18 (d, *J* = 21.7 Hz), 115.42, 111.89, 111.58, 110.36. ^19^F NMR (375 MHz, DMSO): δ_F_ −115.78. HPLC–HRMS (ES^+^) calcd mass for C_18_H_14_FN_3_O_2_ [(M + H)^+^], 324.1143; found [(M + H)^+^], 324.1134; calcd for [(M + Na)^+^], 346.0962; found [(M + Na)^+^], 346.0963; purity 98.12% (HPLC).

#### 5.1.27 (*E*)-5-(3-(3-Fluorophenyl)acrylamido)-1*H*-indole-3-carboxamide (**32**)

General procedure 2, 5-amino-1*H*-indole-3-carboxamide (30.0 mg, 0.171 mmol) in DCM (8 mL), (*E*)-3-(3-fluorophenyl)acrylic acid (34.0 mg, 0.205 mmol), HATU (78.0 mg, 0.205 mmol), and Et_3_N (29 μL, 0.205 mmol) were added, 24 h, followed by flash chromatography on silica gel (petroleum ether/EtOAc 0–100%:MeOH 0–3%) to afford compound **32** as a yellow solid (50% yield). ^1^H NMR (400 MHz, DMSO): δ_H_ 11.49 (1H, d, *J* = 2.9 Hz, 1H), 10.18 (1H, s, NHCO), 8.41 (1H, d, *J* = 2.2 Hz, 4-H), 8.03 (1H, d, *J* = 2.9 Hz, 2-H), 7.69 (1H, dd, *J* = 8.8, 2.1 Hz, 6-H), 7.57 (1H, d, *J* = 15.7 Hz, 3′-H), 7.52–7.31 (4H, m, 2″-H, 5″-H, 6″-H, NH_2a_CO), 7.38 (1H, d, *J* = 8.8 Hz, 7-H), 7.27–7.19 (1H, m, 4″-H), 6.92 (1H, d, *J* = 15.7 Hz, 2′-H), 6.81 (1H, s_br_, NH_2b_CO). ^13^C NMR (100 MHz, DMSO): δ_C_ 166.57, 162.71, 162.49 (d, *J* = 243.7 Hz), 137.87 (d, *J* = 2.6 Hz), 137.60 (d, *J* = 7.8 Hz), 133.11, 132.67, 130.97 (d, *J* = 8.5 Hz), 128.96, 126.47, 124.56, 123.67 (d, *J* = 2.6 Hz), 116.19 (d, *J* = 21.3 Hz), 115.45, 114.03 (d, *J* = 21.8 Hz), 111.89, 111.58, 110.36. ^19^F NMR (375 MHz, DMSO): δ_F_ −113.86. HPLC–HRMS (ES^+^) calcd mass for C_18_H_14_FN_3_O_2_ [(M + H)^+^], 324.1143; found [(M + H)^+^], 324.1153; calcd for [(M + Na)^+^], 346.0962; found [(M + Na)^+^], 346.0963; purity 98.81% (HPLC).

#### 5.1.28 (*E*)-5-(3-(4-Fluorophenyl)acrylamido)-1*H*-indole-3-carboxamide (**33**)

General procedure 2, 5-amino-1*H*-indole-3-carboxamide (20.0 mg, 0.114 mmol) in DCM (8 mL), (*E*)-3-(4-fluorophenyl)acrylic acid (23.0 mg, 0.137 mmol), HATU (52.0 mg, 0.137 mmol), and Et_3_N (19 μL, 0.137 mmol) were added, 24 h, followed by flash chromatography on silica gel (petroleum ether/EtOAc 0–100%:MeOH 0–3%) to afford compound **33** as a yellow solid (52% yield). ^1^H NMR (400 MHz, DMSO): δ_H_ 11.49 (1H, d, *J* = 2.8 Hz, NH), 10.14 (1H, s, NHCO), 8.40 (1H, d, *J* = 2.1 Hz, 4-H), 8.03 (1H, d, *J* = 2.8 Hz, 2-H), 7.74–7.62 (3H, m, 6-H, 2″-H, 6″-H), 7.57 (1H, d, *J* = 15.7 Hz, 3′-H), 7.38 (1H, d, *J* = 8.8 Hz, 7-H), 7.34 (1H, s_br_, NH_2a_CO), 7.28 (2H, t, *J* = 8.8 Hz, 3″-H, 5″-H), 6.83 (1H, d, *J* = 15.7 Hz, 2′-H), 6.80 (1H, s_br_, NH_2b_CO). ^13^C NMR (100 MHz, DMSO): δ_C_ 166.63, 164.00, 162.27 (d, *J* = 146.9 Hz), 138.05, 133.09, 132.80, 131.63 (d, *J* = 3.0 Hz), 129.79 (d, *J* = 8.6 Hz), 128.98, 126.50, 122.86, 116.02 (d, *J* = 21.7 Hz), 115.49, 111.86, 111.60, 110.36. HPLC–HRMS (ES^+^) calcd mass for C_18_H_14_FN_3_O_2_ [(M + H)^+^], 324.1143; found [(M + H)^+^], 324.1153; calcd for [(M + Na)^+^], 346.0962; found [(M + Na)^+^], 346.0963; purity 97.24% (HPLC).

#### 5.1.29 (*E*)-5-(3-(2,3-Difluorophenyl)acrylamido)-1*H*-indole-3-carboxamide (**34**)

General procedure 2, 5-amino-1*H*-indole-3-carboxamide (30.0 mg, 0.171 mmol) in DCM (8 mL), (*E*)-3-(2,3-difluorophenyl)acrylic acid (38.0 mg, 0.205 mmol), HATU (78.0 mg, 0.205 mmol), and Et_3_N (29 μL, 0.205 mmol) were added, 24 h, followed by flash chromatography on silica gel (petroleum ether/EtOAc 0–100%:MeOH 0–3%) to afford compound **34** as a yellow solid (60% yield). ^1^H NMR (400 MHz, DMSO): δ_H_ 11.49 (1H, d, *J* = 2.9 Hz, NH), 10.30 (1H, s, NHCO), 8.42 1H, (d, *J* = 2.2 Hz, 4-H), 8.02 (1H, d, *J* = 2.8 Hz, 2-H), 7.69 (1H, dd, *J* = 8.7, 2.1 Hz, 6-H), 7.61 (1H, d, *J* = 15.9 Hz, 3′-H), 7.56–7.42 (2H, m, 5″-H, 6″-H), 7.47 (1H, s_br_, NH_2a_CO), 7.38 (1H, d, *J* = 8.7 Hz, 7-H), 7.34–7.23 (1H, m, 4″-H), 7.04 (1H, d, *J* = 15.9 Hz, 2′-H), 6.82 (1H, s_br_, NH_2b_CO). ^13^C NMR (100 MHz, DMSO): δ_C_ 166.57, 162.43, 150.16 (dd, *J* = 245.6, 12.5 Hz), 148.28 (dd, *J* = 251.8, 13.4 Hz), 133.18, 132.60, 130.58, 129.01, 127.20 (d, *J* = 6.3 Hz), 126.49, 125.31 (dd, *J* = 7.5, 4.5 Hz), 124.94 (d, *J* = 8.3 Hz), 124.50, 118.12 (d, *J* = 17.3 Hz), 115.42, 111.94, 111.63, 110.38. ^19^F NMR (375 MHz, DMSO): δ_F_ −139.66 (d, *J* = 21.1 Hz), −143.14 (d, *J* = 21.1 Hz). HPLC–HRMS (ES^+^) calcd mass for C_18_H_13_F_2_N_3_O_2_ [(M + H)^+^], 342.1049; found [(M + H)^+^], 342.1043; calcd for [(M + Na)^+^], 364.0868; found [(M + Na)^+^], 364.0869; purity 97.64% (HPLC).

#### 5.1.30 (*E*)-5-(3-(2,4-Difluorophenyl)acrylamido)-1*H*-indole-3-carboxamide (**35**)

General procedure 2, 5-amino-1*H*-indole-3-carboxamide (30.0 mg, 0.171 mmol) in DCM (8 mL), (*E*)-3-(2,4-difluorophenyl)acrylic acid (38.0 mg, 0.205 mmol), HATU (78.0 mg, 0.205 mmol), and Et_3_N (29 μL, 0.205 mmol) were added, 24 h, followed by flash chromatography on silica gel (petroleum ether/EtOAc 0–100%:MeOH 0–3%) to afford compound **35** as a yellow solid (78% yield). ^1^H NMR (400 MHz, DMSO): δ_H_ 11.48 (1H, d, *J* = 2.9 Hz, NH), 10.23 (1H, s, NH), 8.40 (1H, d, *J* = 2.1 Hz, 4-H), 8.01 (1H, d, *J* = 2.9 Hz, 2-H), 7.76 (1H, dd, *J* = 8.7, 6.5 Hz, 6″-H), 7.68 (1H, dd, *J* = 8.8, 2.1 Hz, 6-H), 7.58 (1H, d, *J* = 15.9 Hz, 3′-H), 7.43–7.32 (3H, m, 7-H, 3″-H, NH_2a_CO), 7.21 (1H, td, *J* = 8.5, 2.8 Hz, 5″-H), 6.95 (1H, d, *J* = 15.9 Hz, 2′-H), 6.77 (1H, s_br_, NH_2b_CO). ^13^C NMR (100 MHz, DMSO): δ_C_ 166.54, 162.71, 162.71 (dd, *J* = 249.9, 12.7 Hz), 160.75 (dd, *J* = 253.1, 12.5 Hz), 133.11, 132.67, 130.94 (d, *J* = 4.8 Hz), 130.84, 128.95, 126.46, 125.51 (d, *J* = 6.9 Hz), 119.47 (dd, *J* = 11.9, 3.8 Hz), 115.41, 112.49 (dd, *J* = 21.7, 3.4 Hz), 111.89, 111.57, 110.36, 104.75 (t, *J* = 26.1 Hz). ^19^F NMR (375 MHz, DMSO): δ_F_ −108.70 (d, *J* = 8.9 Hz), −112.06 (d, *J* = 8.9 Hz). HPLC–HRMS (ES^+^) calcd mass for C_18_H_13_F_2_N_3_O_2_ [(M + H)^+^], 342.1049; found [(M + H)^+^], 342.1047; calcd for [(M + Na)^+^], 364.0868; found [(M + Na)^+^], 364.0869; purity 99.29% (HPLC).

#### 5.1.31 (*E*)-5-(3-(2,5-Difluorophenyl)acrylamido)-1*H*-indole-3-carboxamide (**36**)

General procedure 2, 5-amino-1*H*-indole-3-carboxamide (30.0 mg, 0.171 mmol) in DCM (8 mL), (*E*)-3-(2,5-difluorophenyl)acrylic acid (38.0 mg, 0.205 mmol), HATU (78.0 mg, 0.205 mmol), and Et_3_N (29 μL, 0.205 mmol) were added, 24 h, followed by flash chromatography on silica gel (petroleum ether/EtOAc 0–100%:MeOH 0–3%) to afford compound **36** as a yellow solid (52% yield). ^1^H NMR (400 MHz, DMSO): δ_H_ 11.48 (1H, d, *J* = 3.0 Hz, NH), 10.27 (1H, s, NHCO), 8.40 (1H, d, *J* = 2.1 Hz, 4-H), 8.01 (1H, d, *J* = 2.9 Hz, 2-H), 7.67 (1H, dd, *J* = 8.8, 2.1 Hz, 6-H), 7.60–7.50 (2H, m, 3′-H, 6″-H), 7.45–7.19 (3H, m, 7-H, 3″-H, 4″-H), 7.35 (1H, s_br_, NH_2a_CO), 7.01 (1H, d, *J* = 15.9 Hz, 2′-H), 6.79 (1H, s_br_, NH_2b_CO). ^13^C NMR (100 MHz, DMSO): δ_C_ 166.62, 162.54, 158.35 (d, *J* = 240.4 Hz). 156.78 (d, *J* = 246.5 Hz), 133.20, 132.63, 130.80, 129.04, 127.31 (d, *J* = 6.7 Hz), 126.50, 124.24 (dd, *J* = 14.1, 8.2 Hz), 118.19–117.43 (2C, m), 115.49, 115.20 (d, *J* = 3.7 Hz), 112.01, 111.66, 110.40. ^19^F NMR (375 MHz, DMSO): δ_F_ −119.12 (d, *J* = 18.4 Hz), −121.83 (d, *J* = 18.4 Hz). HPLC–HRMS (ES^+^) calcd mass for C_18_H_13_F_2_N_3_O_2_ [(M + H)^+^], 342.1049; found [(M + H)^+^], 342.1048; calcd for [(M + Na)^+^], 364.0868; found [(M + Na)^+^], 364.0868; purity 96.07% (HPLC).

#### 5.1.32 (*E*)-5-(3-(2,6-Difluorophenyl)acrylamido)-1*H*-indole-3-carboxamide (**37**)

General procedure 2, 5-amino-1*H*-indole-3-carboxamide (30.0 mg, 0.171 mmol) in DCM (8 mL), (*E*)-3-(2,6-difluorophenyl)acrylic acid (38.0 mg, 0.205 mmol), HATU (78.0 mg, 0.205 mmol), and Et_3_N (29 μL, 0.205 mmol) were added, 24 h, followed by flash chromatography on silica gel (petroleum ether/EtOAc 0–100%:MeOH 0–3%) to afford compound **37** as a yellow solid (80% yield). ^1^H NMR (400 MHz, DMSO): δ_H_ 11.49 (1H, d, *J* = 2.9 Hz, NH), 10.35 (1H, s, NHCO), 8.43 (1H, d, *J* = 2.1 Hz, 4-H), 8.02 (1H, d, *J* = 2.9 Hz, 2-H), 7.69 (1H, dd, *J* = 8.8, 2.1 Hz, 6-H), 7.59 (1H, d, *J* = 16.1 Hz, 3′-H), 7.54–7.44 (1H, m, 4″-H), 7.38 (d, *J* = 8.7 Hz, 7-H), 7.32 (1H, s_br_, NH_2a_CO), 7.23 (2H, t, *J* = 8.7 Hz, 3″-H, 5″-H), 7.15 (1H, d, *J* = 16.1 Hz, 2′-H), 6.79 (1H, s_br_, NH_2b_CO). ^13^C NMR (100 MHz, DMSO): δ_C_ 166.56, 163.63, 160.82 (dd, *J* = 252.0, 7.0 Hz), 133.17, 132.65, 131.42 (t, *J* = 10.9 Hz), 129.00 (d, *J* = 1.6 Hz), 128.88 (t, *J* = 7.6 Hz), 126.48, 124.91, 115.39, 112.31 (dd, *J* = 24.3, 4.6 Hz), 112.60, 111.93, 111.62, 110.40. ^19^F NMR (375 MHz, DMSO): δ_F_ −112.68. HPLC–HRMS (ES^+^) calcd mass for C_18_H_13_F_2_N_3_O_2_ [(M + H)^+^], 342.1049; found [(M + H)^+^], 342.1037; calcd for [(M + Na)^+^], 364.0868; found [(M + Na)^+^], 364.0868; purity 97.33% (HPLC).

#### 5.1.33 (*E*)-5-(3-(3,4-Difluorophenyl)acrylamido)-1*H*-indole-3-carboxamide (**38**)

General procedure 2, 5-amino-1*H*-indole-3-carboxamide (30.0 mg, 0.171 mmol) in DCM (8 mL), (*E*)-3-(3,4-difluorophenyl)acrylic acid (38.0 mg, 0.205 mmol), HATU (78.0 mg, 0.205 mmol), and Et_3_N (29 μL, 0.205 mmol) were added, 24 h, followed by flash chromatography on silica gel (petroleum ether/EtOAc 0–100%:MeOH 0–3%) to afford compound **38** as a yellow solid (55% yield). ^1^H NMR (400 MHz, DMSO): δ_H_ 11.48 (1H, d, *J* = 2.9 Hz, NH), 10.16 (1H, s, NHCO), 8.38 (1H, d, *J* = 2.1 Hz, 4-H), 8.01 (1H, d, *J* = 2.9 Hz, 2-H), 7.75–7.64 (2H, m, 6-H, 2″-H), 7.58–7.45 (3H, m, 3′-H, 5″-H, 6″-H), 7.37 (1H, d, *J* = 8.7 Hz, 7-H), 7.35 (1H, s_br_, NH_2a_CO), 6.85 (1H, d, *J* = 15.7 Hz, 2′-H), 6.77 (1H, s_br_, NH_2b_CO). ^13^C NMR (100 MHz, DMSO): δ_C_ 166.56, 162.67, 149.99 (dd, *J* = 248.5, 12.5 Hz), 149.71 (dd, *J* = 245.9, 12.8 Hz), 137.05, 133.11, 133.04–132.90 (m), 132.66, 128.97, 126.48, 124.65 (dd, *J* = 6.7, 3.4 Hz), 124.39, 118.17 (d, *J* = 17.3 Hz), 116.42 (d, *J* = 17.3 Hz), 115.45, 111.90, 111.59, 110.35. ^19^F NMR (375 MHz, DMSO): δ_F_ −137.01 (d, *J* = 21.8 Hz), −138.02 (d, *J* = 21.8 Hz). HPLC–HRMS (ES^+^) calcd mass for C_18_H_13_F_2_N_3_O_2_ [(M + H)^+^], 342.1049; found [(M + H)^+^], 342.1046; calcd for [(M + Na)^+^], 364.0868; found [(M + Na)^+^], 364.0870; purity 98.35% (HPLC).

#### 5.1.34 (*E*)-5-(3-(3,5-Difluorophenyl)acrylamido)-1*H*-indole-3-carboxamide (**39**)

General procedure 2, 5-amino-1*H*-indole-3-carboxamide (30.0 mg, 0.171 mmol) in DCM (8 mL), (*E*)-3-(3,5-difluorophenyl)acrylic acid (38.0 mg, 0.205 mmol), HATU (78.0 mg, 0.205 mmol), and Et_3_N (29 μL, 0.205 mmol) were added, 24 h, followed by flash chromatography on silica gel (petroleum ether/EtOAc 0–100%:MeOH 0–3%) to afford compound **39** as a yellow solid (55% yield). ^1^H NMR (400 MHz, DMSO): δ_H_ 11.50 (1H, d, *J* = 3.0 Hz, NH), 10.21 (1H, s, NHCO), 8.40 (1H, d, *J* = 2.1 Hz, 4-H), 8.02 (1H, d, *J* = 2.9 Hz, 2-H), 7.67 (1H, dd, *J* = 8.8, 2.1 Hz, 6-H), 7.55 (1H, d, *J* = 15.7 Hz, 3′-H), 7.46–7.32 (4H, m, 7-H, 2″-H, 6″-H, NH_2a_CO), 7.32–7.21 (1H, m, 4″-H), 6.94 (1H, d, *J* = 15.7 Hz, 2′-H), 6.81 (1H, s_br_, NH_2b_CO). ^13^C NMR (100 MHz, DMSO): δ_C_ 166.61, 162.73 (dd, *J* = 246.2, 13.3 Hz), 162.46, 138.93 (t, *J* = 9.8 Hz), 136.84, 133.19, 132.59, 129.03, 126.50, 126.00, 115.48, 111.98, 111.65, 110.57 (dd, *J* = 19.0, 6.7 Hz), 110.38, 104.67 (t, *J* = 26.1 Hz). ^19^F NMR (375 MHz, DMSO): δ_F_ −110.44. HPLC–HRMS (ES^+^) calcd mass for C_18_H_13_F_2_N_3_O_2_ [(M + H)^+^], 342.1049; found [(M + H)^+^], 342.1047; calcd for [(M + Na)^+^], 364.0868; found [(M + Na)^+^], 364.0868; purity 97.64% (HPLC).

#### 5.1.35 (*E*)-5-(3-(2-(Trifluoromethyl)phenyl)acrylamido)-1*H*-indole-3-carboxamide (**40**)

General procedure 2, 5-amino-1*H*-indole-3-carboxamide (30.0 mg, 0.171 mmol) in DCM (8 mL), (*E*)-3-(2-(trifluoromethyl)phenyl)acrylic acid (44.0 mg, 0.205 mmol), HATU (78.0 mg, 0.205 mmol), and Et_3_N (29 μL, 0.205 mmol) were added, 24 h, followed by flash chromatography on silica gel (petroleum ether/EtOAc 0–100%:MeOH 0–3%) to afford compound **40** as a yellow solid (75% yield). ^1^H NMR (400 MHz, DMSO): δ_H_ 11.51 (1H, d, *J* = 2.9 Hz, NH), 10.30 (1H, s, NHCO), 8.43 (1H, d, *J* = 2.0 Hz, 4-H), 8.03 (1H, d, *J* = 2.9 Hz, 2-H), 7.89–7.76 (4H, m, 3′-H, 3″-H, 5″-H, 6″-H), 7.68 (1H, dd, *J* = 8.8, 2.1 Hz, 6-H), 7.61 (1H, t, *J* = 7.6 Hz, 4″-H), 7.39 (1H, d, *J* = 8.7 Hz, 7-H), 7.37 (1H, s_br_, NH_2a_CO), 6.95 (1H, d, *J* = 15.3 Hz, 2′-H), 6.77 (1H, s_br_, NH_2b_CO). ^13^C NMR (100 MHz, DMSO): δ_C_ 166.55, 162.18, 134.95, 1333.51, 133.17, 132.52, 129.65, 129.01, 128.33, 127.83, 127.48, 127.30, 127.01, 126.42 (q, *J* = 5.6 Hz), 122.88 (q, *J* = 274.0 Hz), 115.36, 111.91, 111.65, 110.38. ^19^F NMR (375 MHz, DMSO): δ_F_ −57.63. HPLC–HRMS (ES^+^) calcd mass for C_19_H_14_F_3_N_3_O_2_ [(M + H)^+^], 374.1111; found [(M + H)^+^], 374.1105; calcd for [(M + Na)^+^], 396.0930; found [(M + Na)^+^], 396.0928; purity 96.73% (HPLC).

#### 5.1.36 Molecular Docking on MAO-B

Docking was performed with Schrödinger software using Glide.^71^ First, ligand states were produced at pH 7.4 by using Epik. Afterward, they were prepared and minimized using the Lig-Prep module. Receptor PDB-ID structures were prepared and minimized with the Protein Preparation Wizard tool in Maestro using the Optimized Potentials for Liquid Simulations 3 (OPLS3) force field. The box for docking calculations was placed to cover the bipartite MAO-B cavity (both the entrance and substrate cavities). Best poses were visually inspected and ranked by the energy. The glide ligand docking algorithm was used with standard precision as the docking method. All images were constructed with the PyMOL software.

#### 5.1.37 Molecular Dynamics Simulations

The selected complexes from molecular docking experiments were employed for 300 ns molecular dynamics (MD) simulations using the Amber18 suite.^72^ Antechamber package in Amber18 was used for obtaining ligand parameters. In brief, partial charges were assigned with the AM1-BCC charge method and the general AMBER force field atom types. Ff14SB force field was used for protein parameters, and the TIP3P water model was used for protein–ligand complex solvation. Sodium and chloride ions were included to get a physiological concentration of 0.15 M. Once prepared, the system was submitted to 500 steps of the steepest descent algorithm followed by 500 steps of the conjugate gradient algorithm. An initial 100 kcal mol^–1^ A^–2^ harmonic potential restriction was applied to the complex, and it was gradually lowered. Then, the whole system was minimized, and it was heated from 0 to 310 K using the Langevin thermostat in the canonical ensemble (*NVT*) with a complex harmonic potential restriction of 2 kcal mol^–1^ A^–2^. Before starting the production run, the system was finally equilibrated at 310 K in the isothermal–isobaric ensemble (*NPT*) without harmonic limitation. Analysis of the trajectories was performed using the cpptraj module of Amber18, and they were visualized using VMD software. Images of MD simulations were constructed using Pymol software.

#### 5.1.38 Virtual Screening

For the virtual screening program, ligands and receptor structures were prepared as previously described in Section 5.1.36-Molecular Docking on MAO-B. Then, molecular docking was performed with AutoDock Vina.^73^ Ligands from an in-house library were submitted to docking calculations with three different and representative MAO-B structures. Briefly, the available crystallized structures of MAO-B protein present in the PDB database, without relevant mutations in the active site, were clustered based on root-mean-square deviation of atomic positions (RMSD) and visual inspection. 2BK3 and 6FW0 PDB-ID structures were selected from the bigger cluster 1, and the 2V5Z structure was selected from cluster 2. After molecular docking experiments, the docking score and visual inspection (correct positioning along the bipartite cavity and residue interactions) were employed for results analysis.

#### 5.1.39 Cell Culture and Reagents

AREc32 cells, shared by CR Wolf, were grown in Dulbecco’s modified Eagle medium (DMEM) with GlutaMAX (Gibco, Invitrogen, Spain), supplemented with 10% (v/v) filtered fetal bovine serum (FBS; Gibco, Invitrogen, Spain), 1% of antibiotics penicillin/streptomycin, and Geneticin (0.8 mg/mL G418; Gibco, Invitrogen, Spain). The SH-SY5Y human neuroblastoma cell line (ECACC, 94030304) was grown in a modified Minimum Essential Medium (MEM) (4.765 g/L MEM; 2.5% MEM-Non-Essential Amino acids (Invitrogen, Madrid, Spain); Ham’s F12 Nutrients Mix (Thermo Fisher, EEUU); 0.5 mM sodium pyruvate (Sigma-Aldrich, Spain); 2 g/L NaHCO_3_ (PanReac, Barcelona, Spain); 10% (v/v) filtered FBS (Gibco, Invitrogen, Spain); and 100 U/mL of penicillin/streptomycin (Invitrogen, Spain). MEFs obtained from NRF2^+/+^ (wild-type; WT) and NRF2^–/–^ (knockout; KO) mice^74^ were grown in DMEM with GlutaMAX (Gibco, Invitrogen, Spain), supplemented with 10% (v/v) FBS (Gibco, Invitrogen, Spain), and 1% of antibiotics penicillin/streptomycin. Cells were maintained at 37 °C under a humidified atmosphere (5% CO_2_ and 95% relative humidity). They were cultured in flasks (Corning, EEUU) until reaching 80% confluence and then subcultured using 0.25% EDTA–trypsin (Thermo Fisher, EEUU) for 5 min and centrifugation at 800 rpm for 10 min (slight variations depending on the cells). Cells were used from the 3rd to 12th passage.

#### 5.1.40 Ethics for Animal Experimentation

All experimental procedures were performed following the Guide for the Care and Use of Laboratory Animals, in accordance with the European Guidelines for the use and care of animals for research, in accordance with the European Union Directive of 22 September 2010 (2010/63/UE), and with the Spanish Royal Decree of 1 February 2013 (53/2013).

#### 5.1.41 Luciferase Activity

##### 5.1.41.1 NRF2 Induction

AREc32 cells are stably transfected with the plasmid pGL-8xARE that contains 8 copies of the ARE sequence, followed by luciferase reporter gen. Consequently, NRF2 induction can be related to the activation of ARE sequences by measuring the luciferase activity in terms of luminescence production. For that purpose, cells were seeded in 96 white flat-bottom well plates (20,000 cells/well). After 24 h, cells were incubated with the selected compounds at the desired concentrations in duplicate for 24 h. The Luciferase Assay System (Promega E1500) was used according to provider protocol, and luminescence was quantified in an Orion II microplate luminometer (Berthold, Germany). Luciferase activity was normalized to basal conditions, and data were expressed as CD values, which means the concentration needed to duplicate the luciferase activity compared to basal conditions. CD values were calculated from dose–response curves fitted by nonlinear regression for each compound after logarithmic transformation of the data using GraphPad Prism 8.0 software. *tert*-Butylhydroquinone (TBHQ) was used as a positive control.

#### 5.1.42 Monoamine Oxidase A and B (MAO-A and MAO-B) Inhibition

Assays were performed in 96 black flat-bottom plates in a final volume of 200 μL/well. Compounds were preincubated at the desired concentrations with human recombinant monoamine oxidase enzymes hrMAO-A or hrMAO-B (Sigma-Aldrich, Spain) at final concentrations of 0.0180 or 0.0135 U/mL, respectively, during 30 min at 37 °C. The enzymatic reaction was started by adding Amplex UltraRed reagent (12.5 μM final concentration; Invitrogen, Spain), horseradish peroxidase (0.02 U/mL final concentration; Sigma-Aldrich, Spain), and tyramine (0.5 mM final concentration; Sigma-Aldrich, Spain). Resorufin production was measured at 530/590 nm (excitation/emission) over 30 min in a FluoStar Optima (BMG Labtech) fluorescence plate reader. Sodium phosphate buffer 25 mM pH 7.4 was employed for preparing all of the solutions. Clorgyline (MAO-A-selective irreversible inhibitor), rasagiline (MAO-B-selective irreversible inhibitor), and safinamide (MAO-B-selective reversible inhibitor) were used as positive controls. IC_50_ values for MAO-A and MAO-B inhibition were calculated from dose–response curves obtained by nonlinear regression fitting after logarithmic transformation of the data using GraphPad Prism 8.0 software.

#### 5.1.43 MAO-A and MAO-B Inhibition Reversibility Assay

Assays were performed following the general procedure 3.2.3. Inhibition of monoamine oxidase enzymes (MAO-A and MAO-B), modifying the preincubation time. Briefly, 0, 15, and 30 min preincubation times were used to study MAO inhibition activity differences. MAO inhibition activity varies over time for irreversible inhibitors; however, it remains stable for reversible-type inhibitors. Rasagiline and safinamide were used as references of irreversible and reversible inhibition, respectively, for comparative purposes, and each compound was incubated at 1.8 × IC_50_ μM final concentration to observe adequate MAO inhibition.

#### 5.1.44 Oxygen Radical Absorbance Capacity Assay

The ORAC assay^75^ was carried out to evaluate the oxygen free radical scavenger capacity of the novel compounds. (±)-6-Hydroxy-2,5,7,8-tetramethylchromane-2-carboxylic acid (Trolox) was used as the reference, and melatonin was used as the positive control and for comparative purposes. Compounds at desired concentrations were diluted in phosphate-buffered saline (PBS, 10 mM, pH 7.4) at 37 °C and placed in a 96 black flat bottom well plate. 150 μL of fluorescein (70 nM final concentration), 25 μL of PBS for blank, 25 μL of Trolox solution for standard, and 25 μL of melatonin or compound were added to each well. An initial fluorescence measurement was recorded to determine the intensity of the basal signal. Then, 25 μL of 2,2′-azobis-amidinopropane dihydrochloride (AAPH, 12 mM final concentration) were added. All samples were evaluated in duplicates. Fluorescence was recorded at 485/520 nm (excitation/emission) for 90 min at 37 °C to obtain the area under the fluorescence decay curve (AUC) in a FLUOstar Optima plate reader (BMG Labtech). Results were expressed in terms of Trolox equivalents (T.equiv), obtained by plotting net AUC versus compound concentration and then performing linear regression and normalizing slopes of the compounds considering Trolox as the reference.

#### 5.1.45 Blood–Brain Barrier Permeation Assay

The PAMPA was employed to test the potential ability of the novel compounds to cross the BBB by passive diffusion.^37^ Briefly, the permeability of the compounds was measured at 100 μM, including positive and negative controls (Supporting Information; Table S3). To model the BBB, the filter membrane of the 96-well donor plate (Multiscreen IP sterile clear plate PDVF membrane, pore size 0.45 μM) was filled with 4 μL of a porcine brain lipid (PBL, Avanti Polar Lipids, Inc.) solution in dodecane (20 mg/mL, Sigma-Aldrich, Madrid, Spain). After 5 min, 180 μL of each compound solution (*V*_D_) in PBS (10 mM, pH 7.4) were added to determine their ability to pass through the membrane. The 96-well acceptor plate (Multiscreen, Millipore Corp.) was loaded with 180 μL of PBS (*V*_A_). Then, the donor filter plate was carefully placed on the acceptor plate to form a sandwich-like system, which was left undisturbed for 4 h at room temperature. After the incubation time, the donor plate was removed, and the absorbance at the maximum absorption wavelength for each compound, which was recorded in both the acceptor and donor wells (150 μL/well) using a UV plate reader, SPECTROstar Nano (BMG Labtech). Concentration of the compounds in the donor and acceptor plates and equilibrium concentration were calculated and expressed as permeability (*P*_e_) according to the eq 1.
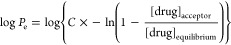
1where .

#### 5.1.46 Neuroprotection Studies in the Neuroblastoma Cell Line

SH-SY5Y cells were seeded at a density of 60,000 cells/well in 96-well transparent plates for 24 h. Then, cells were preincubated with compounds at the desired concentrations. After 24 h, cells were treated with compounds at the desired concentrations plus the selected toxic stimuli, namely, a mixture of rotenone and oligomycin A (R/O; 30 μM/10 μM, respectively; Sigma-Aldrich, Spain), 6-hydroxydopamine (6-OHDA; 100 μM; Sigma-Aldrich, Spain), or 1-methyl-4-phenylpyridinium (MPP^+^; 2.5 mM; Sigma-Aldrich, Spain). The coincubation treatment was maintained for another 24 h. Finally, cell viability was assessed by the 3-(4,5-dimethylthiazol-2-yl)-2,5-diphenyltetrazolium bromide (MTT) method, considering basal conditions as 100% survival. Melatonin, rasagiline, and safinamide were used as references for comparative purposes.

#### 5.1.47 MTT Method for Cell Viability Measurement

When required, cell viability was assessed by an MTT assay. Briefly, cells were incubated for 120 min with tetrazolium salt (3-(4,5-dimethylthiazol-2-yl)-2,5-diphenyltetrazolium bromide solution (MTT, 0.5 mg/mL), which is reduced to purple insoluble formazan crystals by oxidoreductase enzymes from viable cells. Then, the formazan crystals were solubilized with DMSO, and absorbance was measured at 535 nm in a SPECTROstar Nano microplate reader (BMG Labtech). Basal absorbance was set to 100%, and results were normalized to basal conditions.

#### 5.1.48 Nitrite Production Measurement

Primary glial cells or BV2 cell line cells were pretreated with compounds at the desired concentrations for 24 h. Then, cells were incubated with lipopolysaccharide (LPS, 1 μg/mL; Sigma-Aldrich, Spain) and compounds at the desired concentrations for 18 h. After that, nitrite production was assessed by the modified Griess method.^76^ Briefly, 150 μL of each sample were mixed with 75 μL of 4,4′-diamino-diphenylsulfone (Dapsone) and 75 μL of *N*-(1-naphthyl)ethylendiamine (Neda). The resulting mixture was incubated at room temperature for 5 min. Then, absorbance was measured at 550 nm in a microplate reader, SPECTROstar Nano (BMG Labtech). Data were normalized to basal conditions and considered as 100% of nitrite production. EC_50_ values were calculated from dose–response curves obtained from representing the percentage of nitrites reduction vs compound concentration.

#### 5.1.49 Real-Time Quantitative Polymerase Chain Reaction (RT-qPCR)

Total RNA from cell samples was extracted with the TRIzol reagent (Sigma-Aldrich, Spain), and 1 μg was reverse transcribed using PrimeScriptTM RT Reagent Kit (perfect Real Time) (Takara). RT-qPCR was performed with qPCRBIO SyGreen Mix LoRox polymerase (Cultek) in a 7500 Fast Real-Time PCR System (Applied Biosystems). Thermal cycling conditions used were according to the instruction of the SyGreen Mix protocol, and the relative expression levels were calculated using the comparative ΔΔCt method. The primers were obtained from Sigma-Aldrich (Supporting Information; Table S6).

#### 5.1.50 Liver Microsomes Metabolic Stability

The pooled mixed gender HLMs and Pooled CD1 mouse liver microsomes (female) were purchased from Tebubio. The NADPH Regenerating System was purchased from Promega. All other reagents and solvents were of special or analytical grade and were commercially available. The assays were performed according to previously reported protocol^77^ with slight changes. Briefly, the test compound (1 μM) was preincubated with the NADPH regenerating system in a thermoblock with constant agitation (750 rpm) for 5 min at 37 °C in 0.1 M phosphate buffer, pH 7.4. Reactions were initiated by adding liver microsomes at a final concentration of 0.5 mg/mL in a total volume of 250 μL. Samples were collected at 0, 10, 15, 30, and 60 min incubation times at 37 °C with constant agitation (750 rpm). The reaction was stopped by adding 250 μL of cold (−20 °C) acetonitrile. The samples were sonicated for 5 min and then centrifuged for 5 min at 10,000*g*, 4 °C. The supernatants were analyzed with LC/MS for the amount of parent compound remaining, and the CL_int_ and *t*_1/2_ were determined.

#### 5.1.51 Pharmacokinetic Evaluation

Pharmacokinetic evaluation was externalized to SAI Life Sciences. Briefly, Healthy Male C57BL/6 Mice (8–12 weeks old) weighing between 20 ands 28 g. A total of six male mice were divided into two groups as Group 1 (*n* = 3) and Group 2 (*n* = 3), with 3 mice/dose group serial sampling design. Animals in Group 1 were administered intravenously with a solution formulation of **11** at a 10 mg/kg dose. Animals in Group 2 were administered through an oral route with a hazy uniform suspension formulation of PD46 at a 50 mg/kg dose. The formulation vehicle for the IV group was 5% DMSO, 5% Solutol HS-15, and 90% normal saline, and for the PO group was 0.5% Tween 80 and 99.5% of 0.5% NaCMC in RO water. The dosing volumes for intravenous and oral administration were 5 and 10 mL/kg, respectively. Blood samples (approximately 30 μL) were collected through the saphenous vein from a set of three mice at 0.083 (for IV only), 0.25, 0.5, 1, 2, 4, 6 (only for PO), 8, and 24 h. Immediately after blood collection, plasma was harvested by centrifugation at 10,000 rpm for 10 min at 4 °C, and samples were stored at −70 ± 10 °C until bioanalysis. A 100 μL of internal standard prepared in acetonitrile (Cetirizine, 20 ng/mL) was added except for the blank, where 100 μL of acetonitrile was added in a 96-well Solvinert filter plate, followed by 10 μL of study sample plasma or spiked calibration standard was added. The filter plate is then centrifuged for 5 min at a speed of 2500 rpm at 4 °C with a 96-well sample collection plate. Following centrifugation, collected samples were analyzed by using LC–MS/MS (LLOQ = 1.46 ng/mL). The noncompartmental analysis tool of Phoenix WinNonlin (Version 8.3) was used to assess the pharmacokinetic parameters. Peak plasma concentration (*C*_max_) and time for the peak plasma concentration (*T*_max_) were the observed values. The areas under the concentration time curve (AUC_last_ and AUC_inf_) were calculated by the linear trapezoidal rule. The terminal elimination rate constant, *k*_e_ was determined by regression analysis of the linear terminal portion of the log plasma concentration–time curve. The terminal half-life (*T*_1/2_) was estimated at 0.693/*k*_e_. CLIV = Dose/AUC_inf_; *V*_ss_ = MRT × CLIV; % *F* = [mean AUCPO × Dose IV)/(mean AUCIV × Dose PO)] × 100.

#### 5.1.52 Statistical analysis

Data are represented as mean ± SEM. IC_50_ and LD_50_ values were calculated by nonlinear regression analysis of individual dose–response curves. Experimental and control groups were compared using the *t*-test, and multiple groups were compared using a one-way analysis of variance test (one-way ANOVA) followed by a Newman–Keuls or Tukey’s post hoc test. Statistical significance was set at *p < 0.033, **p < 0.002, and ***p < 0.001. Data was analyzed using GraphPad Prism 8.0 software.

## Supporting Information Available

Primer sequences for RT-qPCR, experimental procedures and original data for PK evaluation, copies of ^1^H NMR and ^13^C NMR spectra and HPLC traces, and exact mass analysis (PDF)
Molecular formula strings (CSV)
PDB files of docking complexes of compound **11** and **30** with MAO-B crystal structures PDB-IDs 2BK3, 2V5Z, and 6FW0 (ZIP)
PDB files of docking structures of virtual screening families VS2–14 with the MAO-B crystal structure PDB-ID 2BK3 (ZIP)
PDB files of docking structures of virtual screening families with alternative conformations VS3, 4, 6, 9, 11, 12, 13, and 14 with MAO-B crystal structure PDB-ID 2BK3 (ZIP)

## Abbreviations

6-OHDA: 6-hydroxydopamine
ADMET: absorption, distribution, metabolism, excretion, and toxicology
BBB: blood–brain barrier
BIM; CD: concentration required to double the specific luciferase reporter activity
CNS: central nervous system
DMT: disease modifying therapy
EC50: half maximal effective concentration
ARE: antioxidant response element
HATU: 1-[bis(dimethylamino)methylene]-1*H*-1,2,3-triazolo[4,5-*b*]pyridinium 3-oxide hexafluorophosphate
HMOX1: heme oxygenase-1
hMAO-A: human monoamine oxidase A
hMAO-B: human monoamine oxidase B
IC50: half maximal inhibitory concentration
IL: interleukin
iNOS: inducible nitric oxide synthase
LPS: lipopolysaccharide; multi-target directed ligand
NDD: neurodegenerative disease
NQO1: NAD(P)H dehydrogenase quinone 1
NRF2: nuclear factor (erythroid-derived 2)-like 2 transcription factor
OA: Okadaic acid
ORAC: oxygen radical absorbance capacity
OS: oxidative stress
PD: Parkinsons disease
RNS: reactive nitrogen species
ROS: reactive oxygen species
SAR: structure–activity relationship
T.equiv: Trolox equivalent
TNF-α: tumor necrosis factor α
TPSA: topological polar surface area
Trolox: (±)-6-hydroxy-2,5,7,8-tetramethylchromane-2-carboxylic acid
WT: wildtype
