# Supplementary material for: Network-Based Drug Optimization toward the Treatment of Parkinson’s Disease: NRF2, MAO-B, Oxidative Stress, and Chronic Neuroinflammation
Source: J Med Chem. 2025 Jan 17;68(3):3495–517. doi: 10.1021/acs.jmedchem.4c02659 (PMC11881042; doi:10.1021/acs.jmedchem.4c02659)

Supporting Information

Network-based drug optimization towards the treatment of Parkinson's disease: NRF2, MAO-B, oxidative stress and chronic neuroinflammation

Pablo Duarte,[1,&] Francisco J. Sanchez-Porro,[1,&] Enrique Crisman,[1,2] Ángel Cores,[3] Irene Jiménez,[1,2] Antonio Cuadrado,[4,5] J. Carlos Menéndez[3] and Rafael León[1,*]

[1]Instituto de Química Médica, Consejo Superior de Investigaciones Científicas (IQM-CSIC), 28006 Madrid, Spain.

[2]Fundación Teófilo Hernando para la I+D del Medicamento, 28290 Las Rozas, Madrid, Spain.

[3]Unidad de Química Orgánica y Farmacéutica, Departamento de Química en Ciencias Farmacéuticas, Facultad de Farmacia, Universidad Complutense, 28040 Madrid, Spain.

[4]Instituto de Investigaciones Biomédicas "Alberto Sols" UAM-CSIC, Instituto de Investigación Sanitaria La Paz (IdiPaz) and Departamento de Bioquímica, Facultad de Medicina, UAM, 28029 Madrid, Spain.

[5]Centro de Investigación Biomédica en Red Sobre Enfermedades Neurodegenerativas (CIBERNED), ISCIII, 28029 Madrid, Spain.

*Senior corresponding Author: Rafael León, PhD (rafael.leon@iqm.csic.es)

[&]These authors contributed equally to this work.

TABLE OF CONTENTS

Table S1. Average docking score of SBVS results. S2
Figure S1. Docking images of representative examples of SBVS results. S3
Figure S2. Docking images of non-preferred positions of SBVS results. S4
Table S2. Selected top compounds from SBVS with summarized yields. S5
Table S3. PAMPA values of control compounds expressed as P_e ± SEM. S5
Table S4. Neuroprotective activity of novel derivatives. S6
Table S5. Cytotoxicity of novel derivatives in SH-SH5Y, AREc32 and BV2 cells. S7
Table S6. Primer sequences for RT-qPCR. S8
Compound 11 PK evaluation original data. S9
Copies of spectra (^{1}H-NMR, 13-NMR) S22
Copies HPLC chromatograms and HRMS S55

Table S1. Average docking score energies (kcal/mol) for the different subfamilies submitted to the SBVS program.

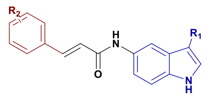

Subfamily	R_1	Docking score mean (kcal/mol)
1*H*-indole derivatives (VS1)	-H	-11.256
2-(1*H*-indol-3-yl)ethan-1-amine derivatives (VS2)	$-(CH_2)_2NH_2$	-10.448
2-(1*H*-indol-3-yl)-*N*-methylethan-1-amine derivatives (VS3)	$-(CH_2)_2NHCH_3$	-9.388
2-(1*H*-indol-3-yl)-*N*,*N*-dimethylethan-1-amine derivatives (VS4)	$CH_2)_2NH(CH_3)_2$	-8.491
(1*H*-indol-3-yl)methanamine derivatives (VS5)	$-CH_2NH_2$	-10.553
1-(1*H*-indol-3-yl)-*N*-methylmethanamine derivatives (VS6)	$-CH_2NHCH_3$	-10.190
1-(1*H*-indol-3-yl)-*N*,*N*-dimethylmethanamine derivatives (VS7)	$-CH_2NH(CH_3)_2$	-9.051
1*H*-indole-3-carboxamide derivatives (VS8)	$-CONH_2$	-11.252
N-methyl-1*H*-indole-3-carboxamide derivatives (VS9)	$-CONHCH_3$	-10.693
2-(1*H*-indol-3-yl)acetamide derivatives (VS10)	$-CH_2CONH_2$	-10.571
2-(1*H*-indol-3-yl)-*N*-methylacetamide derivatives (VS11)	$-CH_2CONHCH_3$	-9.553
(*S*)-2-(((1*H*-indol-3-yl)methyl)amino)propanamide derivatives (VS12)	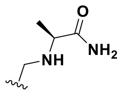	-8.599
(*R*)-2-(((1*H*-indol-3-yl)methyl)amino)propanamide derivatives (VS13)	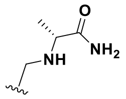	-8.259
N-(2-(1*H*-indol-3-yl)ethyl)-*N*-methylprop-2-yn-1-amine derivatives (VS14)	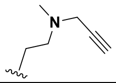	-7.540

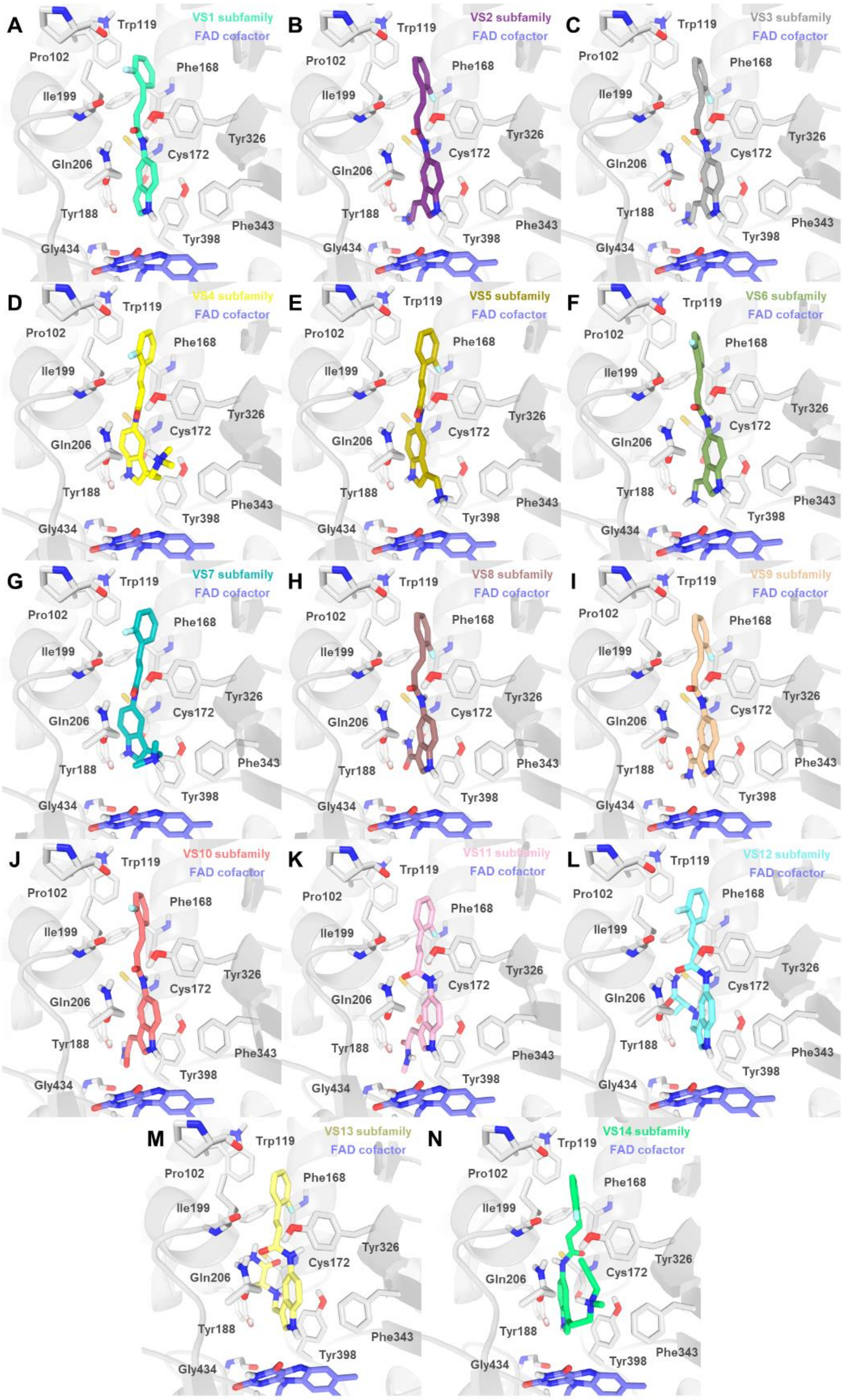

Figure S1. Molecular docking results for the different subfamilies submitted to the virtual screening program. (**A**-**N**) Detailed positions of the different **VS1**-**VS14** subfamily representative compounds in complex with MAO-B structure PDB-ID **2BK3**. MAO-B protein is represented as gray colored cartoon and compounds are represented as colored sticks.

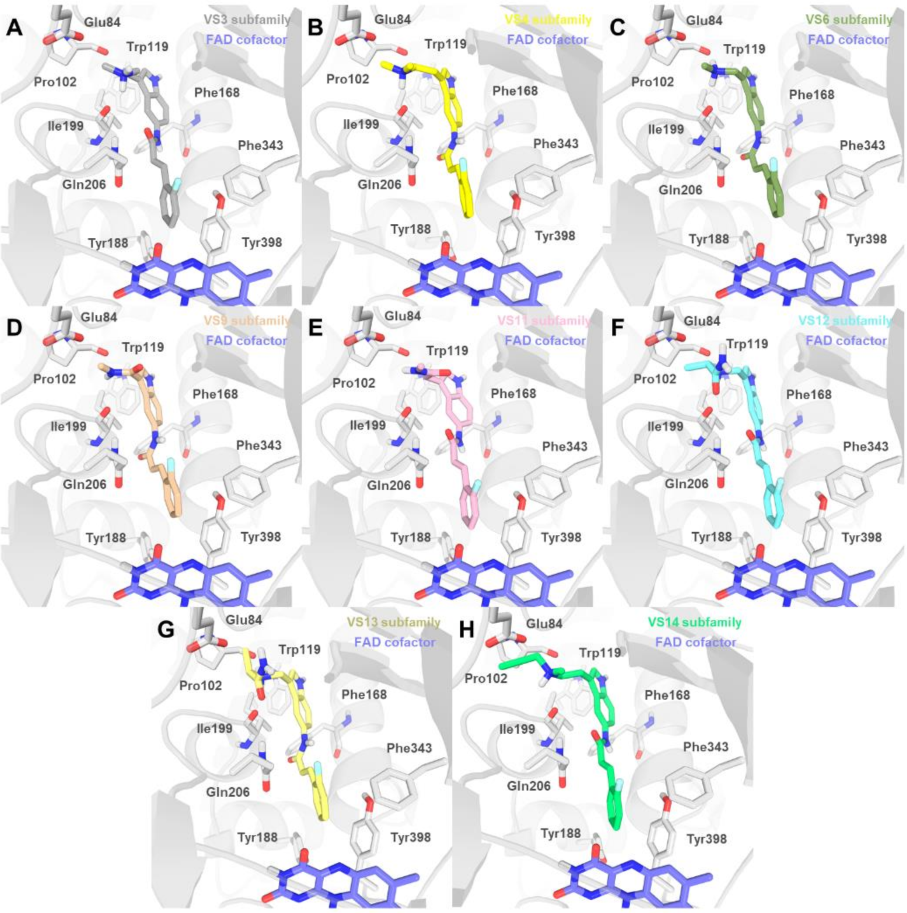

Figure S2. Molecular docking results for some subfamilies showing binding modes with different and non-preferred compound orientation. (**A-H**) Detailed positions of different subfamily representative compounds in complex with MAO-B structure PDB-ID **2BK3**. MAO-B protein is represented as gray colored cartoon and compounds are represented as colored sticks.

Table S2. Selected top compounds from the SBVS program with summarized yields.

Compound	R_1	R_2	Yield (%)
**7**	-H	2-Me	90
**8**	-H	3-Me	83
**9**	-H	2-Cl	95
**10**	-H	2,6-Cl	72
**11**	-H	2-F	88
**12**	-H	3-F	85
**13**	-H	4-F	91
**14**	-H	2,3-F	79
**15**	-H	2,4-F	80
**16**	-H	2,5-F	82
**17**	-H	2,6-F	80
**18**	-H	3,4-F	65
**19**	-H	3,5-F	70
**20**	-H	2-CF_3	95
**21**	-H	3-CF_3	78
**27**	-$CONH_2$	2-Me	75
**28**	-$CONH_2$	3-Me	65
**29**	-$CONH_2$	2-Cl	78
**30**	-$CONH_2$	2,6-Cl	40
**31**	-$CONH_2$	2-F	45
**32**	-$CONH_2$	3-F	50
**33**	-$CONH_2$	4-F	52
**34**	-$CONH_2$	2,3-F	60
**35**	-$CONH_2$	2,4-F	78
**36**	-$CONH_2$	2,5-F	52
**37**	-$CONH_2$	2,6-F	80
**38**	-$CONH_2$	3,4-F	55
**39**	-$CONH_2$	3,5-F	55
**40**	-$CONH_2$	2-CF_3	75

Table S3. Prediction of the BBB passive permeability of control compounds expressed as P_e ± SEM

Compound	PAMPA	
	Pe (10^{-6} cm s^{-1})	Prediction
**Verapamil**	17.5 ± 4.2	CNS +
**Caffeine**	1.65 ± 1.5	CNS -

Table S4. Neuroprotective activity of novel *N*-(1*H*-indol-5-yl)cinnamamide compounds against the toxicity exerted by rotenone/oligomycin A mixture (30/10 µM) and 6-hydroxydopamine (100 µM). SH-SY5Y cells were treated with the corresponding derivatives (1 µM) or reference compounds (melatonin and rasagiline, 1 µM) during 24 h. Thereafter, cells were treated with novel compounds, melatonin or rasagiline (1 µM) and the corresponding toxic stimuli for 24 h. Cell viability was assessed by the MTT assay.

Compound	R_2	R/O (30/10 µM) Cell viability (%)	R/O (30/10 µM) Protection (%)	6-OHDA (100 µM) Cell viability (%)	6-OHDA (100 µM) Protection (%)
**Basal**		100		100	
**Toxic**		76.3 ± 2.1		75.3 ± 3.1	
**Melatonin**		94.2 ± 7.0	78.4*	86.4 ± 3.4	50.7*
**Safinamide**		91.2 ± 6.4	63.9*	83.0 ± 1.9	26.6*
**DMF**		86.7 ± 7.5	46.6	84.2 ± 3.1	34.0*
**7**	2-Me	94.0 ± 7.3	70.9*	92.3 ± 2.8	71.0***
**8**	3-Me	82.4 ± 6.2	27.3	86.9 ± 3.8	44.5*
**9**	2-Cl	95.4 ± 5.6	82.7**	93.5 ± 2.8	73.1***
**10**	2,6-Cl	97.9 ± 4.4	88.7**	89.8 ± 2.9	60.8**
**11**	2-F	99.3 ± 5.7	94.2**	97.1 ± 1.8	89.7***
**12**	3-F	97.1 ± 2.0	88.5***	94.4 ± 2.8	76.0***
**13**	4-F	99.5 ± 3.7	99.8***	84.5 ± 2.0	36.6*
**14**	2,3-F	98.9 ± 4.2	97.5***	92.0 ± 3.6	63.5**
**15**	2,4-F	97.3 ± 7.2	84.6*	86.2 ± 2.0	44.6**
**16**	2,5-F	99.8 ± 0.27	99.1***	92.3 ± 1.5	66.1***
**17**	2,6-F	93.6 ± 6.4	74.1*	92.8 ± 3.5	68.2***
**18**	3,4-F	96.6 ± 2.9	86.8***	93.5 ± 2.8	73.9***
**19**	3,5-F	94.7 ± 5.4	77.3**	91.0 ± 3.9	65.5**
**20**	2-CF_3	99.7 ± 4.0	96.8***	90.6 ± 3.7	64.6**
**21**	3-CF_3	95.5 ± 7.5	76.9*	95.8 ± 2.4	85.1***
**27**	2-Me	90.1 ± 6.1	57.4*	76.6 ± 1.4	NA
**28**	3-Me	90.6 ±7.2	59.2	75.3 ± 2.5	NA
**29**	2-Cl	86.9 ± 8.0	48.2	77.6 ± 3.7	7.53
**30**	2,6-Cl	91.9 ± 9.9	69.5	85.5 ± 1.3	37.7**
**31**	2-F	78.8 ± 8.0	12.9	72.5 ± 2.7	NA
**32**	3-F	87.7 ± 10	53.0	83.4 ± 2.8	31.4*
**33**	4-F	83.9 ± 7.6	35.1	78.9 ± 2.9	7.13
**34**	2,3-F	90.9 ± 9.2	65.3	72.5 ± 3.6	NA
**35**	2,4-F	85.1 ± 13	39.4	77.9 ± 2.5	3.96
**36**	2,5-F	95.8 ± 8.4	85.9*	83.3 ± 3.9	29.2
**37**	2,6-F	81.3 ± 14	20.8	78.4 ± 2.7	11.4
**38**	3,4-F	96.6 ± 2.0	85.0***	78.0 ± 2.7	7.25
**39**	3,5-F	91.0 ± 5.8	60.4*	84.7 ± 3.5	36.9*
**40**	2-CF_3	80.6 ± 14	23.4	80.2 ±3.2	16.1

NA: not active Data are expressed as mean ± SEM of 3-5 independent experiments. Statistical analysis was performed following one-way ANOVA ($p < 0.05$). ###$p < 0.001$ *vs* basal condition, *$p < 0.033$, **$p < 0.002$ and ***$p < 0.001$ *vs* the toxic condition after Tukey's post-hoc test.

Table S5. Cytotoxicity elicited by novel *N*-(1*H*-indol-5-yl)cinnamamide compounds. Viability was measured as MTT reduction in presence of increasing concentrations of derivatives. LD_{50} values were calculated from dose-response curves. Data are expressed as mean ± SEM of at least 3 independent experiments.

Compound	LD_{50} (μM) SH-SY5Y	LD_{50} (μM) AREc32	LD_{50} (μM) BV2
**Melatonin**	> 100	> 30	> 30
**Rasagiline**	> 100	> 30	NE
**7**	> 100	> 20	> 30
**8**	> 100	> 20	> 30
**9**	> 100	> 20	> 30
**10**	> 100	> 20	> 30
**11**	> 100	> 20	> 30
**12**	> 100	> 20	> 30
**13**	> 100	> 20	> 30
**14**	> 100	17.2 ± 0.49	> 30
**15**	> 100	> 20	> 30
**16**	> 100	> 20	> 30
**17**	> 100	> 20	24.5 ± 3.2
**18**	> 100	17.0 ± 0.78	> 30
**19**	> 100	> 20	> 30
**20**	> 100	> 20	> 30
**21**	> 100	> 20	> 30
**27**	26.4 ± 7.0 % @ 100 μM	> 20	> 30
**28**	> 100	> 20	> 30
**29**	> 100	> 20	> 30
**30**	> 100	> 20	> 30
**31**	46.0 ± 3.4 % @ 100 μM	> 20	> 30
**32**	> 100	> 20	> 30
**33**	> 100	> 20	> 30
**34**	> 100	> 20	> 30
**35**	> 100	> 20	> 30
**36**	> 100	> 20	> 30
**37**	> 100	> 20	> 30
**38**	> 100	> 20	> 30
**39**	> 100	> 20	> 30
**40**	> 100	> 20	> 30

NE: not evaluated

Table S6. Primer sequences for RT-qPCR.

Gene	Forward (5`- 3`)	Reverse (5`- 3`)
hGCLM	TCAAACTCTTCATCATCAAC	TTCTAATTCCTCCCAGTAAG
hHMOX1	TGCTCAACATCCAGCTCTTTGA	GCAGAATCTTGCACTTTGTTGC
hNQO1	GTTCATAGGAGAGTTTGCTT	CCTTGCAGAGAGTACATGGA
hOSGIN1	ATGCAGAAGAAGCGAAGAGGT	CCCAGACCCTTCTTGACCAC
hACTB	TCCTTCCTGGGCATGGAG	AGGAGGAGCAATGATCTTGATCTT
mHmox1	CACAGATGGCGTCACTTCGTC	GTGAGGACCCACTGGAGGAG
mNqo1	GGTAGCGGCTCCATGTACTC	CATCCTTCCAGGATCTGCAT
mOsgin1	CGGTGACATCGCCCACTAC	GCTCGGACTTAGCCCACTC
mB2m	ACCCTGGTCTTTCTGGTGCTT	TAGCAGTTCAGTATGTTCGGCTT

m: mouse; h: human.

PK evaluation compound 11

1.0 STUDY OBJECTIVE

To determine the plasma pharmacokinetics study of compound **11** in Male C57BL/6 Mice following a single Intravenous administration at 10 mg/kg and oral administration at 50 mg/kg dose.

2.0 COMPLIANCE AND ANIMAL WELFARE

The study was conducted at AAALAC accredited facility of Sai Life Sciences Limited, Hyderabad, India, in accordance with the Study Protocol SAIDMPK/PK-24-03-0374. All procedures of the present study were in accordance with the guidelines provided by the Committee for the Purpose of Control and Supervision of Experiments on Animals (CPCSEA) as published in The Gazette of India, December 15, 1998. Prior approval of the Institutional Animal Ethics Committee (IAEC) was obtained before initiation of the study. The study was conducted as non-GLP; however, all appropriate documentation was maintained in the study file.

3.0 MATERIAL AND METHODS

3.1 Test Item

The test item compound **11**; Mol. Wt.: 280.30; Purity: Considered as 97% was received from sponsor.

3.2 Test System

Healthy Male C57BL/6 Mice (8-12 weeks old) weighing between 20 to 28 g were procured from Global, India. Three mice were housed in each cage. Temperature and humidity were maintained at 22 ± 3 ºC and 30-70%, respectively and illumination was controlled to give a sequence of 12 h light and 12 h dark cycle. Temperature and humidity were recorded by auto-controlled data logger system. All the animals were provided with a laboratory rodent diet. Reverse osmosis water treated with ultraviolet light was provided by *ad libitum*.

3.3 Study Design

Total six male mice were divided into two groups as Group 1 (n=3) and Group 2 (n=3) with 3 mice/dose group serial sampling design. Animals in Group 1 were administered Intravenously with solution formulation of compound **11** at 10 mg/kg dose. Animals in Group 2 were administered through oral route with hazy uniform suspension formulation of compound **11** at 50 mg/kg dose. The formulation vehicle for IV group was 5% DMSO, 5% Solutol HS-15 and 90% Normal saline and for the PO group was 0.5% Tween 80 and 99.5% of 0.5% NaCMC in RO water. The dosing volume for Intravenous and oral administration were 5 mL/kg and 10 mL/kg respectively. The assignment of animals was shown in the table below:

Table S7. Study design.

Group	Route	Dose (mg/kg)	Matrix	Animal ID
1	IV	10	Plasma	55-57
2	PO	50		58-60

3.4 Formulation Preparation

IV (2 mg/mL): Accurately weighed quantity (3.05 mg) of compound **11** for IV dosing was added in a labeled bottle and corrected for purity. Excipients volumes were calculated to prepare solution formulation of compound **11** at strength of 2.00 mg/mL. The volume 0.074 mL of DMSO was added and vortexed followed by sonication to get a clear solution. Further, 0.074 mL of Solutol HS-15 and 1.331 mL of normal saline were added, vortexed and sonicated for ~2 minutes to get a clear solution. The amount measured and calculation details are as below:

Table S8. Formulation for IV administration.

Ingredients	% Content	Quantity
COMPOUND **11**	-	3.05 mg
DMSO	5	0.074 mL
Solutol HS-15	5	0.074 mL
Normal saline	90	1.331 mL

PO (5 mg/mL): Accurately weighed quantity (7.66 mg) of compound **11** for PO dosing was added in a labeled bottle and corrected for purity. Excipients volumes were calculated to prepare suspension formulation of compound **11** at strength of 5 mg/mL.

To this, 0.007 mL of Tween 80 and 1.479 mL of NaCMC in RO water were added, vortexed and sonicated for ~2 minutes and the physical appearance of the final formulation was a hazy uniform suspension. The amount measured and calculation details are as below:

Table S9. Formulation for PO administration.

Ingredients	% content	Quantity
COMPOUND **11**	-	7.66 mg
Tween 80	0.5	0.007 mL
0.5% NaCMC in RO water	99.5	1.479 mL

3.5 Formulation Analysis Results

After preparation of formulation, a volume of 200 µL was aliquot for analysis. The formulations were analyzed and found to be within the acceptance criteria (in-house acceptance criteria is ± 20% from the nominal value). Formulations were prepared freshly prior to dosing.

Table S10. Formulation analysis.

Compound	Theoretical Conc. (mg/mL)	Conc. Found (mg/mL)	% Change
COMPOUND **11**	2.00	2.05	2.50
	5.00	4.32	-13.60

3.6 Clinical Observations

Following a single Intravenous at 10 mg/kg and oral at 50 mg/kg dose administration, animals were normal without any clinical signs.

3.7 Sample Collection

Blood samples (approximately 30 µL) were collected through saphenous vein from a set of three mice at 0.083 (For IV only), 0.25, 0.5, 1, 2, 4, 6 (only for PO), 8 and 24 h. Immediately after blood collection, plasma was harvested by centrifugation at 10000 rpm, 10 min at 4ºC and samples were stored at -70±10ºC until bioanalysis.

3.8 Bioanalysis

Concentrations of compound **11** in mice plasma samples were determined by fit for purpose LC-MS/MS method. The sample processing and extraction procedure, chromatographic and mass spectrometric conditions were presented in Annexure I.

4.0 DATA ANALYSIS

The Non-Compartmental-Analysis tool of Phoenix WinNonlin® (Version 8.3) was used to assess the pharmacokinetic parameters. Peak plasma concentration (Cmax) and time for the peak plasma concentration (Tmax) were the observed values. The areas under the concentration time curve (AUClast and AUCinf) were calculated by linear trapezoidal rule. The terminal elimination rate constant, ke was determined by regression analysis of the linear terminal portion of the log plasma concentration-time curve. The terminal half-life (T1/2) was estimated at 0.693/ke. CLIV= Dose/AUCinf; Vss= MRT X CLIV; %F = [Mean AUCPO × Dose IV) / (Mean AUCIV × Dose PO)] × 100.

5.0 RESULTS

Following a single intravenous administration of compound **11** in Male C57BL/6 Mice at 10 mg/kg dose, compound showed moderate plasma clearance (60% than the normal liver blood flow in mice: 90 mL/min/kg) and moderate volume of distribution (1.24-folds higher than total body water: 0.7 L/kg) with terminal elimination plasma half-life of 0.36 h.

Following oral administration at 50 mg/kg, the peak plasma concentrations were observed at 0.25 h, suggesting rapid absorption with the corresponding peak plasma concentration (C_{max}) of 1143.95 ng/mL. The oral bioavailability was found to be 11 %.

Table S11: Pharmacokinetic data of compound **11** in Male C57BL/6 Mice following a single Intravenous and oral administration (Dose: 10 mg/kg, IV and 50 mg/kg, PO)

Matrix	Route	Dose (mg/kg)	T_{max} (h)	$^{a}C0/C_{max}$ (ng/mL)	AUC_{last} (h*ng/mL)	$T_{1/2}$ (h)	CL (mL/min/kg)	Vss (L/kg)	%F
Plasma	IV	10	-	10683.87	3179.82	0.36	54.30	0.87	-
	PO	50	0.25	1143.95	1743.29	1.83	-	-	11

a-back extrapolated concentration in IV arm.

Table S12: Individual pharmacokinetic parameters of compound **11** in Male C57BL/6 Mice following a single intravenous administration (Dose: 10 mg/kg)

Animal ID	$^{a}C_0$ (ng/mL)	AUC_{last} (h*ng/mL)	$T_{1/2}$ (h)	CL (mL/min/kg)	Vss (L/kg)
55	13387.13	3479.05	0.22	47.85	0.70
56	9031.01	2394.10	0.42	69.60	1.15
57	9633.47	3666.32	0.45	45.44	0.76
**Mean**	**10683.87**	**3179.82**	**0.36**	**54.30**	**0.87**
SD	2360.39	686.87	0.12	13.30	0.24
CV%	22	22	34	25	28

a – Back extrapolated concentration in IV group.

Table S13: Individual pharmacokinetic parameters of compound **11** in Male C57BL/6 Mice following a single oral administration (Dose: 50 mg/kg)

Animal ID	T_{max} (h)	C_{max} (ng/mL)	AUC_{last} (h*ng/mL)	$T_{1/2}$ (h)
58	0.25	1299.36	1649.18	2.28
59	0.25	1073.21	2023.98	1.00
60	0.25	1059.28	1556.70	2.22
**Mean**	**0.25**	**1143.95**	**1743.29**	**1.83**
SD	0.00	134.77	247.45	0.72
CV%	0	12	14	39

Table S14: Individual plasma concentration-time data of compound **11** in Male C57BL/6 Mice following a single intravenous administration (Dose: 10 mg/kg)

Animal ID	Plasma Concentration (ng/mL)							
	Time (h)							
	0.083	0.25	0.5	1	2	4	8	24
55	9279.21	4438.54	1761.88	227.09	14.25	BLQ	BLQ	1.57[e]
56	6374.95	3163.26	884.69	273.39	16.43	1.46	BLQ	BLQ
57	8407.68	6393.68	1738.72	258.39	23.03	2.13	BLQ	BLQ
**Mean**	**8020.61**	**4665.16**	**1461.76**	**252.96**	**17.90**	**1.80[d]**	**NA**	**NA**
SD	1490.32	1627.09	499.89	23.62	4.57	NA	NA	NA
CV%	19	35	34	9	26	NA	NA	NA

LLOQ = 1.46 ng/mL; NA- Not applicable; BLQ-below limit of quantification; d-average of two values reported; e-excluded from data analysis.

Table S15: Individual plasma concentration-time data of compound **11** in Male C57BL/6 Mice following a single oral administration (Dose: 50 mg/kg)

**Animal ID**	**Plasma Concentration (ng/mL)**							
	**Time (h)**							
	**0.25**	**0.5**	**1**	**2**	**4**	**6**	**8**	**24**
58	1299.36	1180.79	563.27	191.41	56.82	20.77	16.80	BLQ
59	1073.21	970.59	762.29	274.09	161.99	37.35	10.17	BLQ
60	1059.28	964.52	529.36	246.58	55.55	18.14	15.91	BLQ
**Mean**	**1143.95**	**1038.63**	**618.31**	**237.36**	**91.45**	**25.42**	**14.29**	**NA**
SD	134.77	123.15	125.84	42.10	61.09	10.42	3.60	NA
CV%	12	12	20	18	67	41	25	NA

LLOQ = 1.46 ng/mL; NA- Not applicable; BLQ- below limit of quantification.

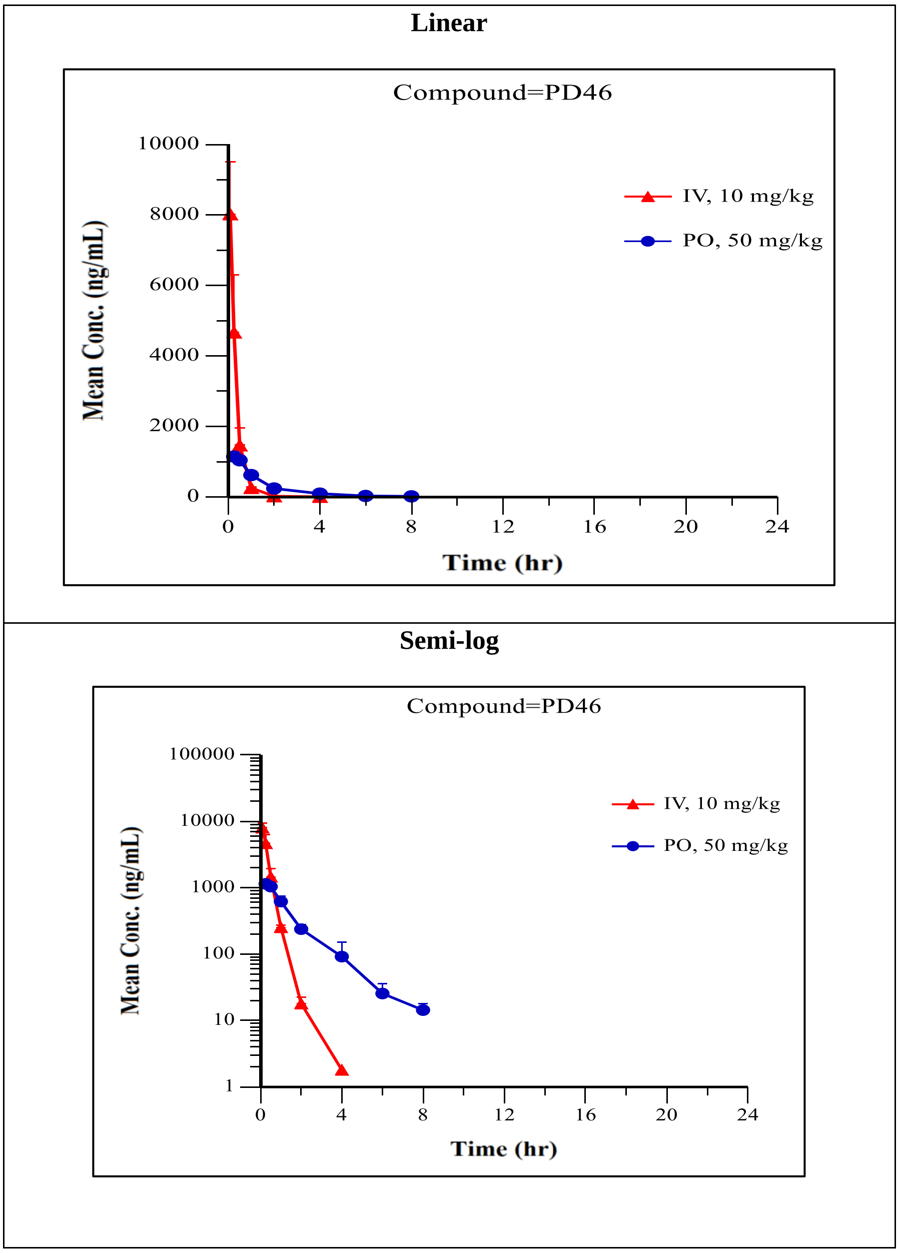

Figure S3: Plasma concentrations-time profiles (mean + SD) of compound **11** in Male C57BL/6 Mice following a single Intravenous and oral administration (Dose: 10 mg/kg, IV and 50 mg/mg, PO).

6.0 ANNEXURE I

Bioanalytical Summary

LC Conditions:

Mobile Phase A: 0.1% Formic acid in Acetonitrile
B: 10 mM Ammonium formate

Column: ACQUITY UPLC BEH C18, 30 X 2.1 mm, 1.7 µm

Injection Volume (µL) : 0.5

Column Oven Temperature (°C) : 45

Retention Time (in min) : **Analyte:** Compound **11** : 1.06
IS: Cetirizine : 0.97

Table S16: LC Gradient Used

Time (Minutes)	Flow Rate (mL/min)	PUMP A (% Conc)	PUMP B (% Conc)
Initial	0.600	5	95
0.30	0.600	5	95
1.00	0.600	95	5
1.20	0.600	95	5
1.40	0.600	5	95
1.60	0.600	5	95

Mass Conditions

Table S17: MRM Transitions:

**Analyte ID / IS ID**	**Q1**	**Q3**	**Cone V**	**CE**	**Dwell time (sec)**
Compound **11**	281.01	132.94	40	20	0.011
Cetirizine	389.13	200.95	10	20	0.011

Table S18: Source Parameter:

Polarity	Positive
Cone Voltage	20
Cone Gas Flow(L/hr)	150
Nebulizer Gas Flow(L/hr)	7
Desolvation Gas Flow(L/hr)	1000
Capillary Voltage(kV)	3.27
Desolvation Temperature	500
Source Temperature	150

Extraction Procedure:

The extraction procedure for plasma samples and the respective spiked plasma calibration standards was similar:

A 100 µL of internal standard prepared in acetonitrile (cetirizine, 20 ng/mL) was added except for blank, where 100 µL of acetonitrile was added in 96 well Solvinert filter plate followed by 10 µL of study sample plasma or spiked calibration standard was added. Filter plate is then centrifuged for 5 minutes at a speed of 2500 rpm at 4 °C with 96 well sample collection plate. Following centrifugation, collected samples were analyzed using LC-MS/MS.

Representative Chromatograms:

Quantify Sample Report **MassLynx V4.2 SCN1012** Page 1 of 183
SAI LIFE SCIENCES LTD

Dataset: W:\CSIC_2024.PRO\PeakDB\PK_24_03_0374_375_PD46_SAB1_MICE_PLM_290324.qld

Last Altered: Monday, April 01, 2024 17:02:04 India Standard Time
Printed: Monday, April 01, 2024 17:42:58 India Standard Time

Method: D:\Projects\CSIC_2024.PRO\MethDB\CSIC_MIX_290324.mdb 01 Apr 2024 17:01:53
Calibration: 01 Apr 2024 17:02:04

ID: PD46_SAB1_2 _MICE_PLM_BLK, Date: 29-Mar-2024

PD46 (1) **Cetrizine (1)**

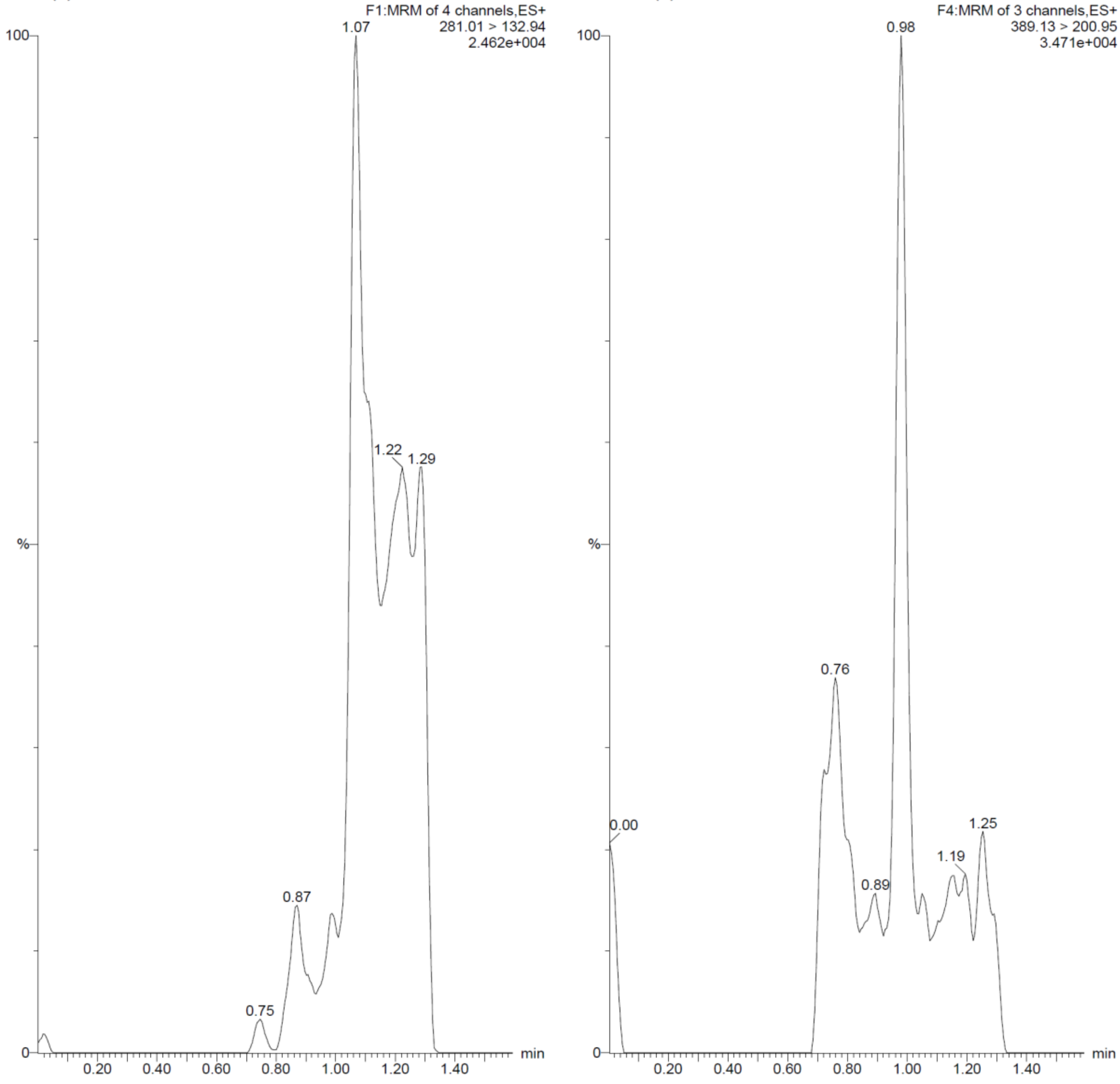

Figure S4: Representative chromatogram of blank mice plasma in the LC-MS/MS analysis of compound **11.**

Quantify Sample Report **MassLynx V4.2 SCN1012** Page 5 of 183
SAI LIFE SCIENCES LTD

Dataset: W:\CSIC_2024.PRO\PeakDB\PK_24_03_0374_375_PD46_SAB1_MICE_PLM_290324.qld

Last Altered: Monday, April 01, 2024 17:02:04 India Standard Time
Printed: Monday, April 01, 2024 17:42:58 India Standard Time

ID: PD46_SAB1_2 _MICE_PLM_CS_02, Date: 29-Mar-2024

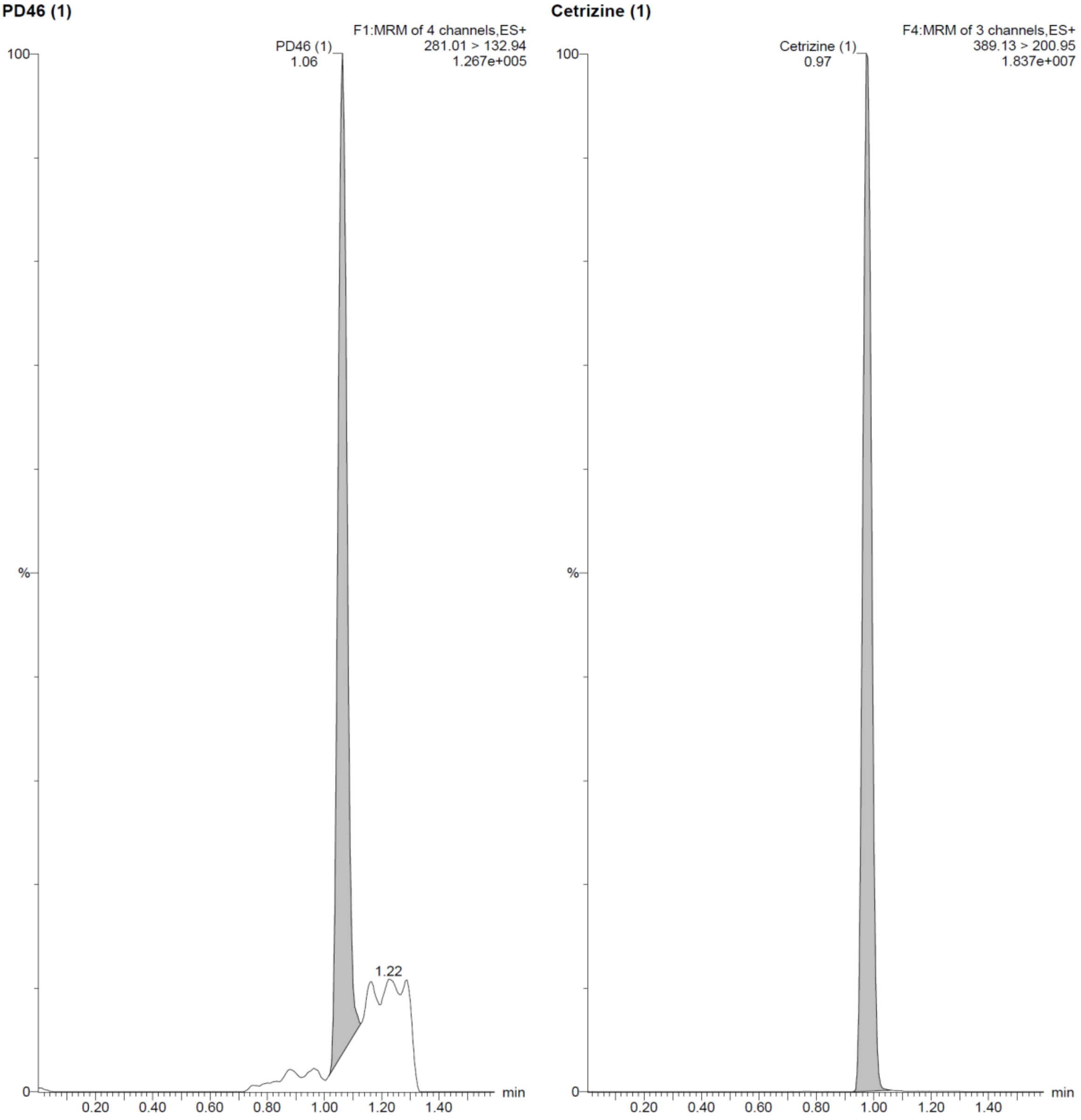

Figure S5: Representative LC-MS/MS chromatogram of compound **11** LLOQ standard and Cetirizine (IS) in mice plasma

Quantify Sample Report **MassLynx V4.2 SCN1012** Page 12 of 183
SAI LIFE SCIENCES LTD

Dataset: W:\CSIC_2024.PRO\PeakDB\PK_24_03_0374_375_PD46_SAB1_MICE_PLM_290324.qld

Last Altered: Monday, April 01, 2024 17:02:04 India Standard Time
Printed: Monday, April 01, 2024 17:42:58 India Standard Time

ID: PD46_SAB1_2 _MICE_PLM_CS_09, Date: 29-Mar-2024

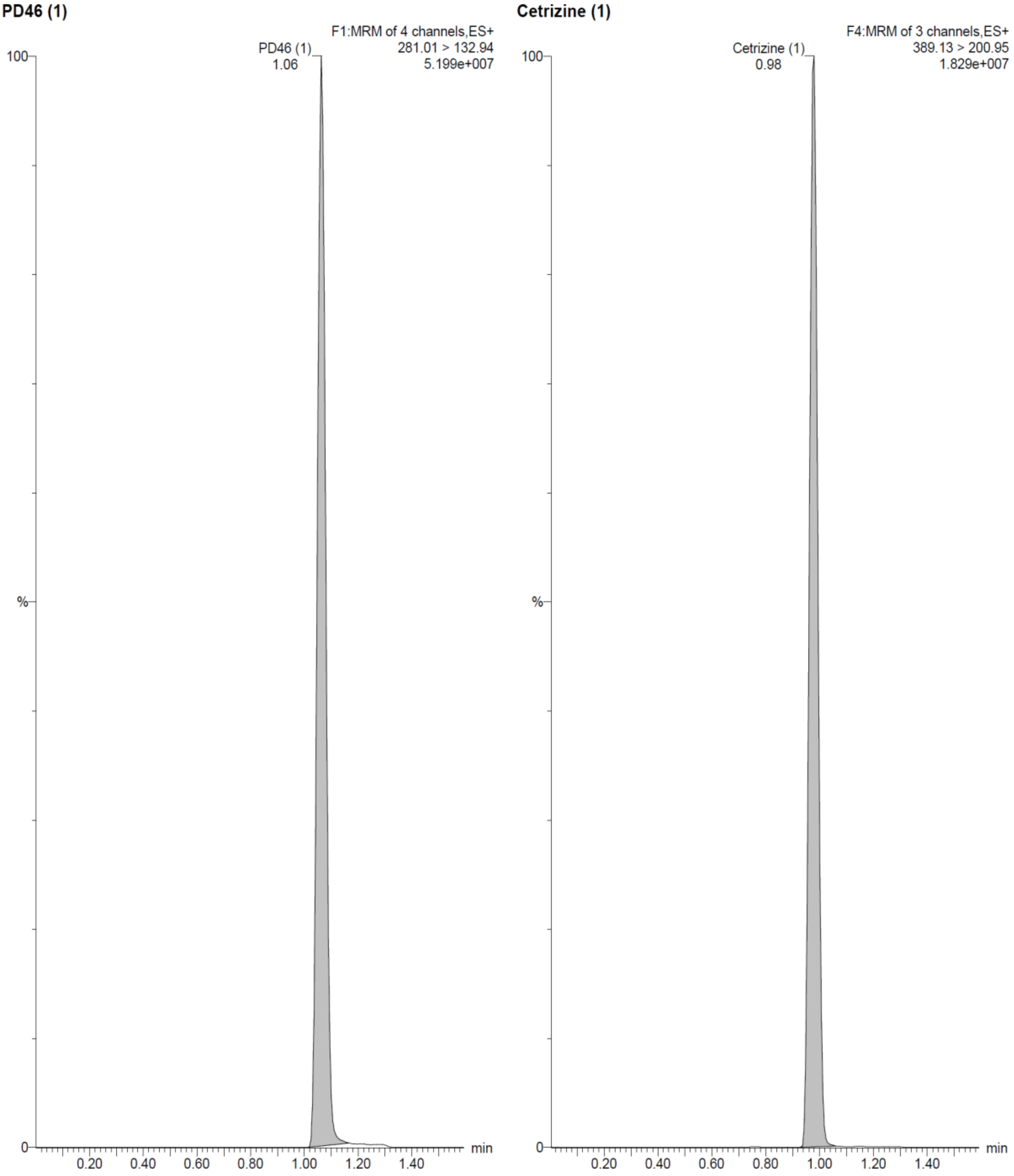

Figure S6: Representative LC-MS/MS chromatogram of compound **11** ULOQ standard and Cetirizine (IS) in mice plasma

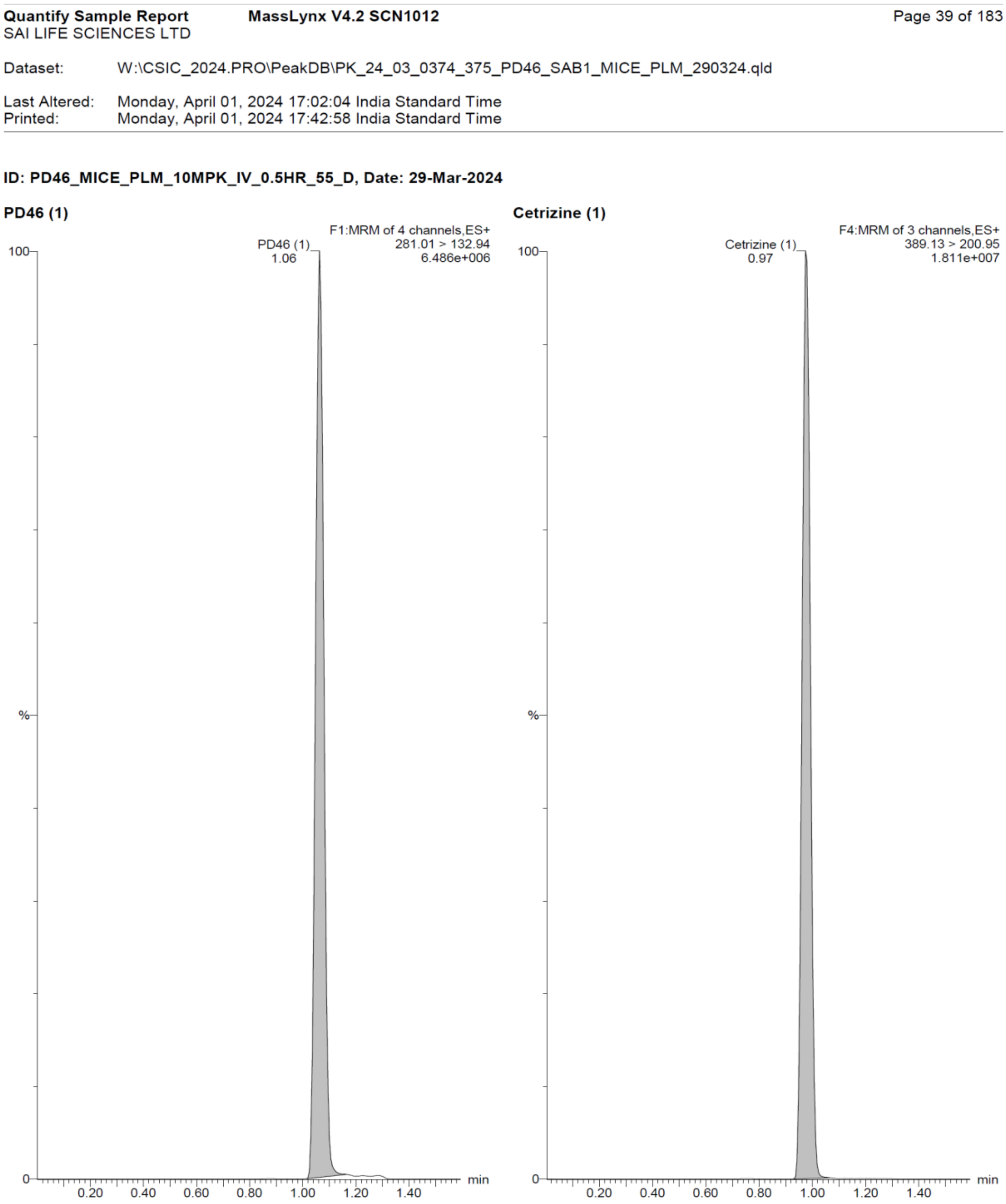

Figure S7: Representative LC-MS/MS chromatogram of compound **11** study sample (mice plasma) and Cetirizine (IS)

Quantify Calibration Report **MassLynx V4.2 SCN1012** Page 1 of 1
SAI LIFE SCIENCES LTD

Dataset: W:\CSIC_2024.PRO\PeakDB\PK_24_03_0374_375_PD46_SAB1_MICE_PLM_290324.qld

Last Altered: Monday, April 01, 2024 17:02:04 India Standard Time
Printed: Monday, April 01, 2024 17:43:51 India Standard Time

Method: D:\Projects\CSIC_2024.PRO\MethDB\CSIC_MIX_290324.mdb 01 Apr 2024 17:01:53
Calibration: 01 Apr 2024 17:02:04

Compound name: PD46 (1)
Correlation coefficient: r = 0.996748, r^2 = 0.993506
Calibration curve: 0.00204866 * x + 0.000816541
Response type: Internal Std (Ref 4), Area * (IS Conc. / IS Area)
Curve type: Linear, Origin: Exclude, Weighting: 1/x^2, Axis trans: None

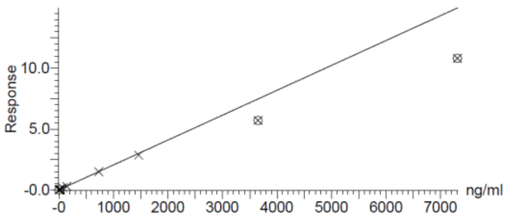

Figure S8: Calibration curve of compound **11** in mice plasma.

Copies of spectra

(*E*)-*N*-(1*H*-indol-5-yl)-3-(*o*-tolyl)acrylamide (7)

^{1}H NMR (400 MHz, DMSO)

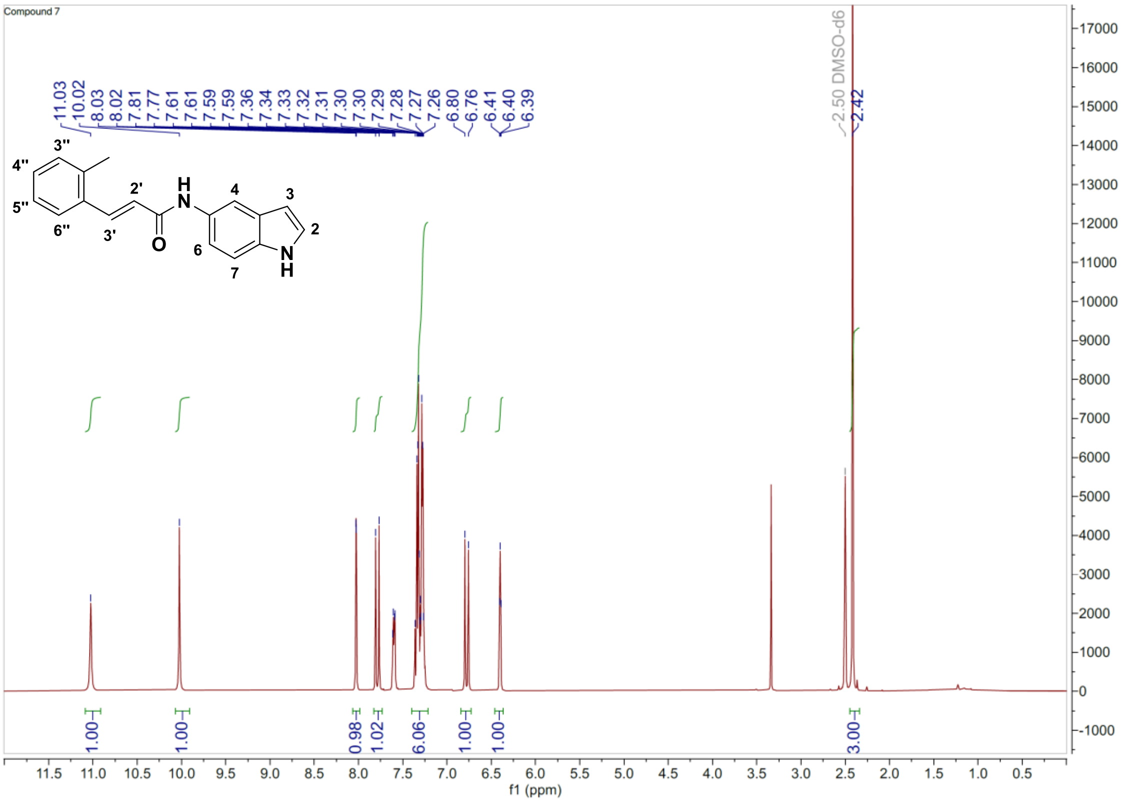

^{13}C NMR (100 MHz, DMSO)

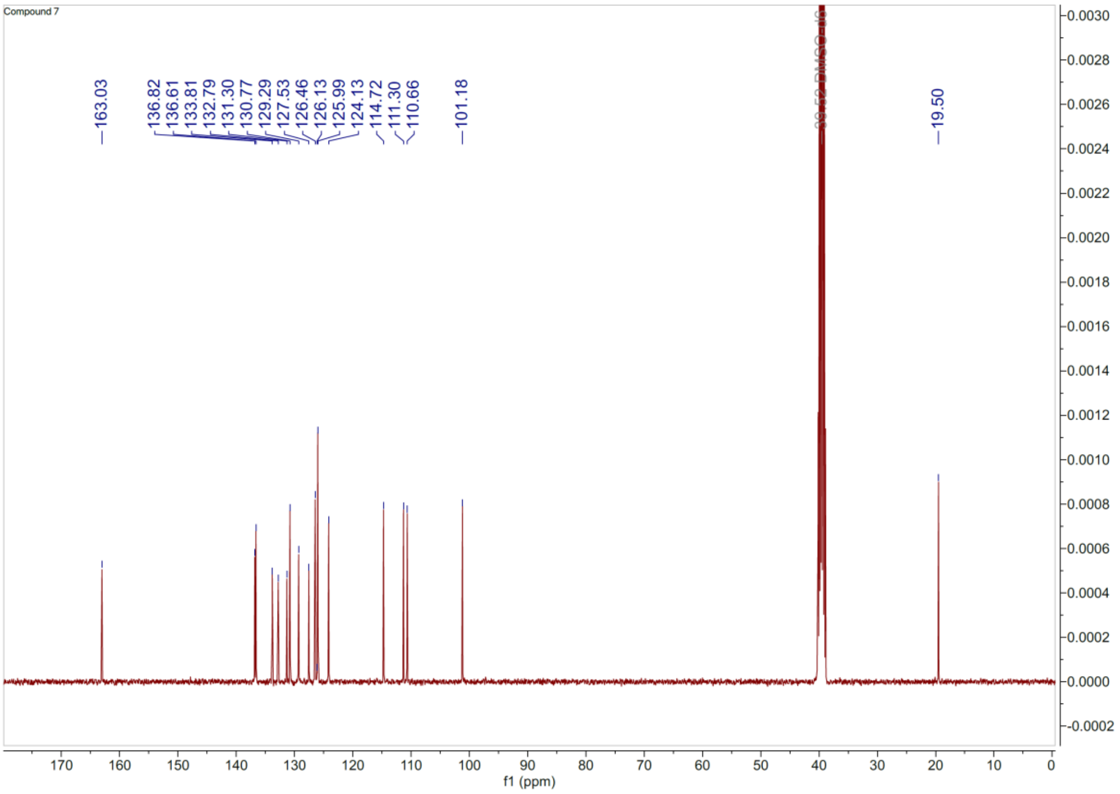

(*E*)-*N*-(1*H*-indol-5-yl)-3-(*m*-tolyl)acrylamide (8)

^{1}H NMR (400 MHz, DMSO)

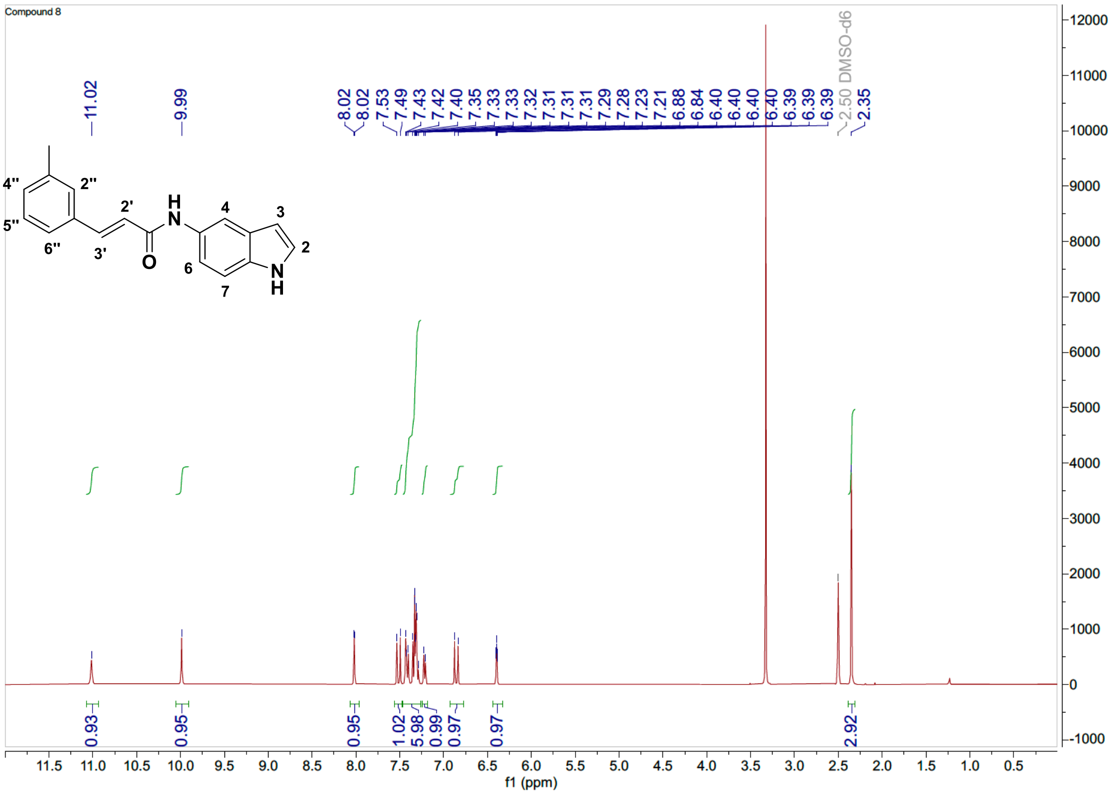

^{13}C NMR (75 MHz, DMSO)

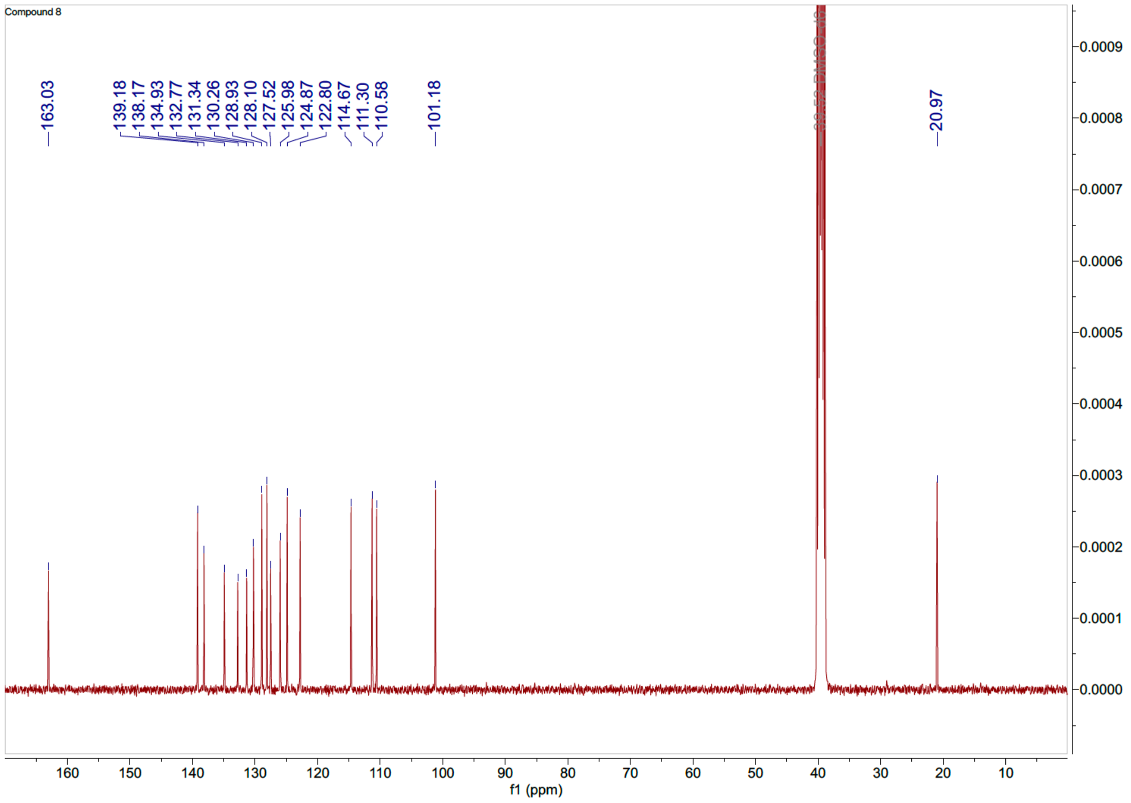

(*E*)-3-(2-chlorophenyl)-*N*-(1*H*-indol-5-yl)acrylamide (9)

^{1}H NMR (400 MHz, DMSO)

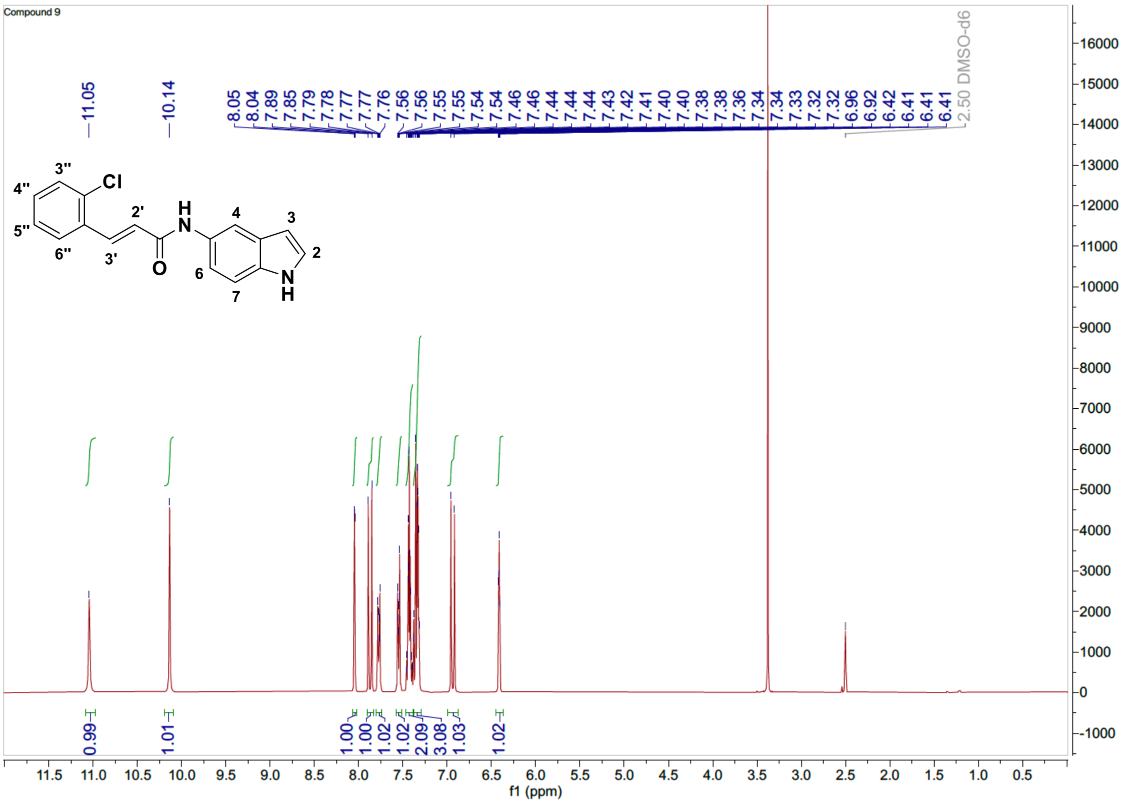

^{13}C NMR (100 MHz, DMSO)

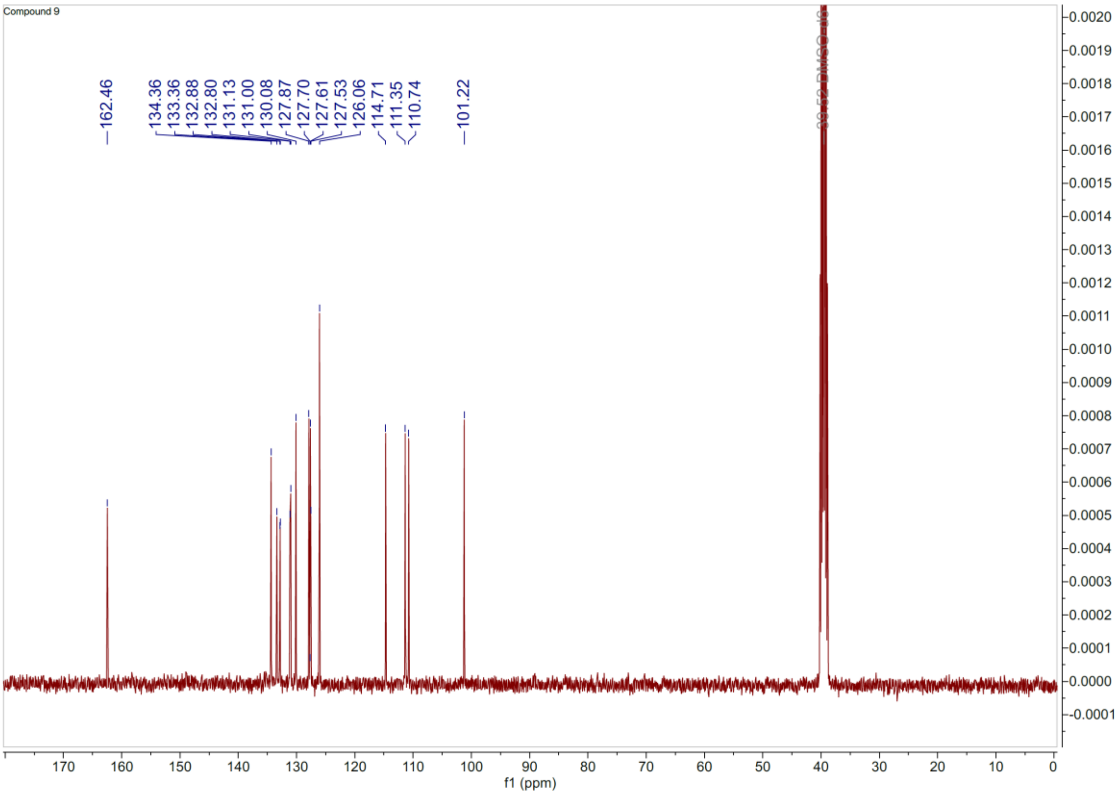

(*E*)-3-(2,6-dichlorophenyl)-*N*-(1*H*-indol-5-yl)acrylamide (10)

^{1}H NMR (400 MHz, DMSO)

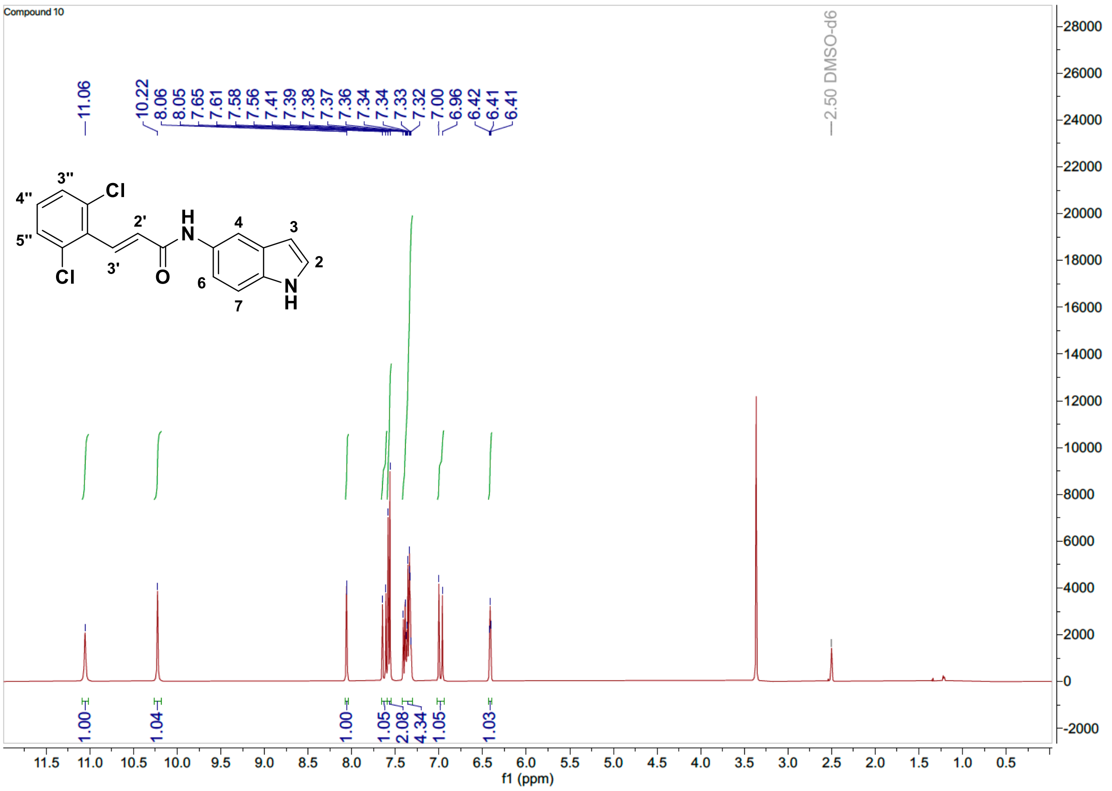

^{13}C NMR (100 MHz, DMSO)

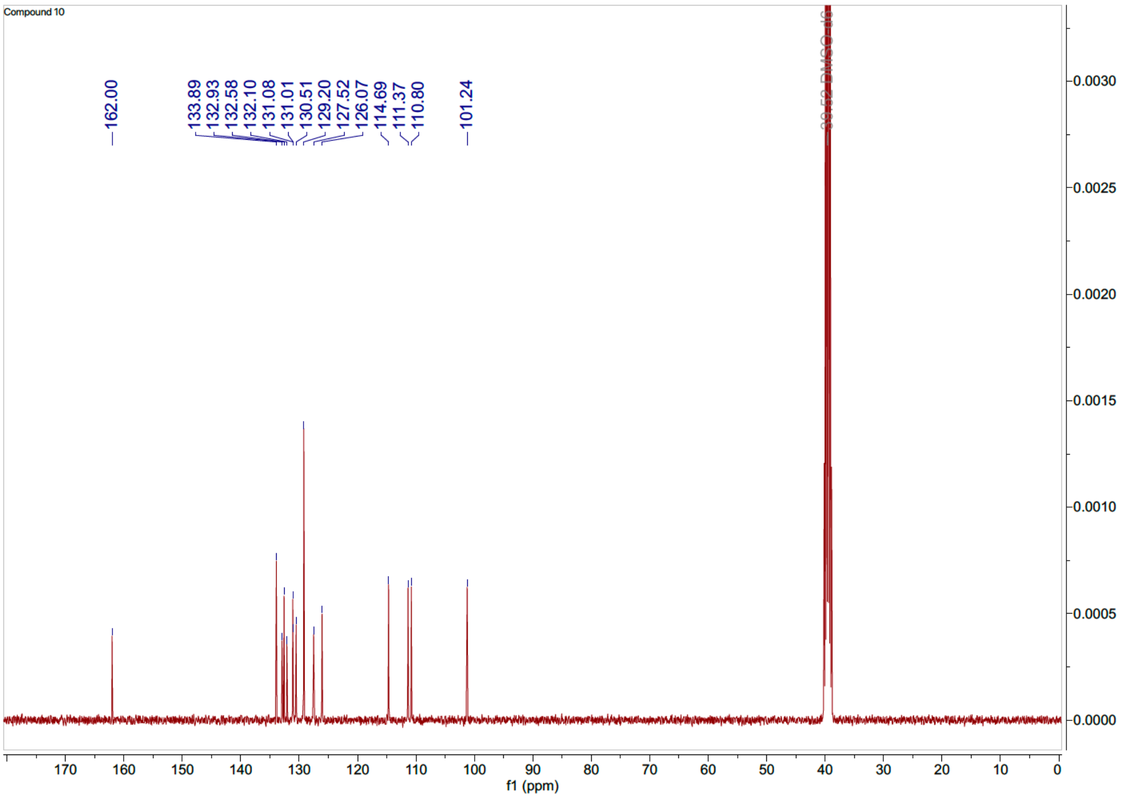

(*E*)-3-(2-fluorophenyl)-*N*-(1*H*-indol-5-yl)acrylamide (11)

^{1}H NMR (400 MHz, DMSO)

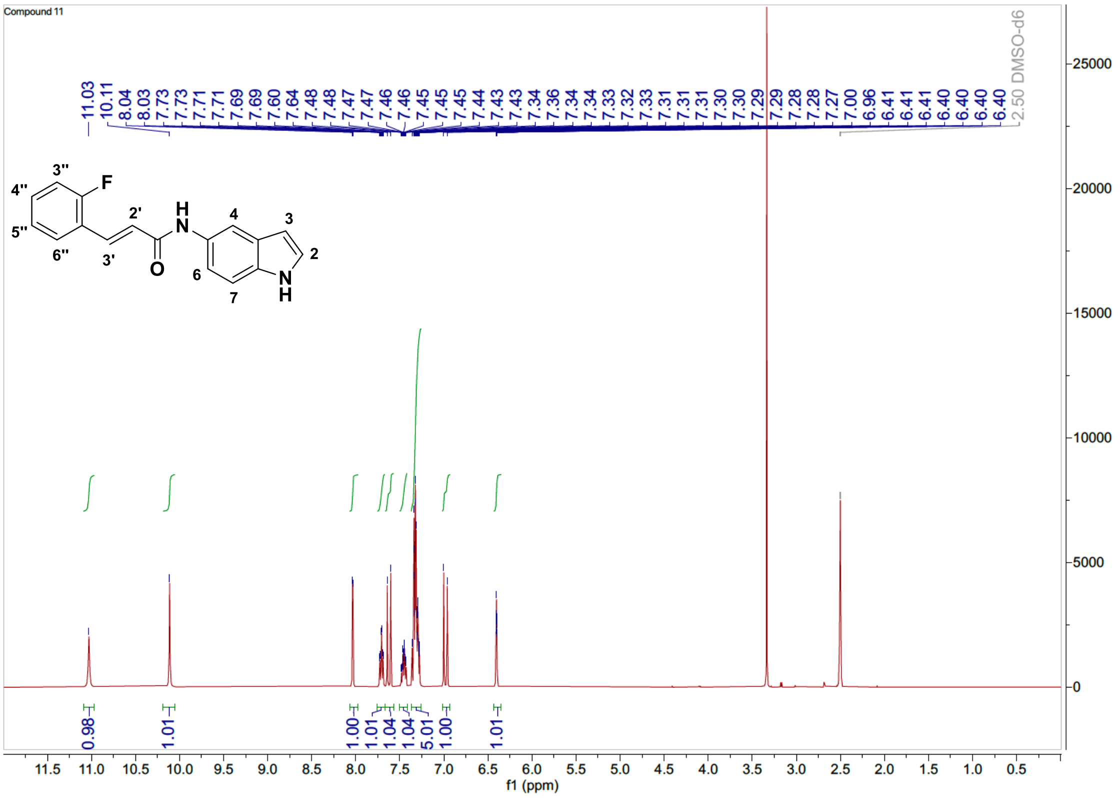

^{13}C NMR (100 MHz, DMSO)

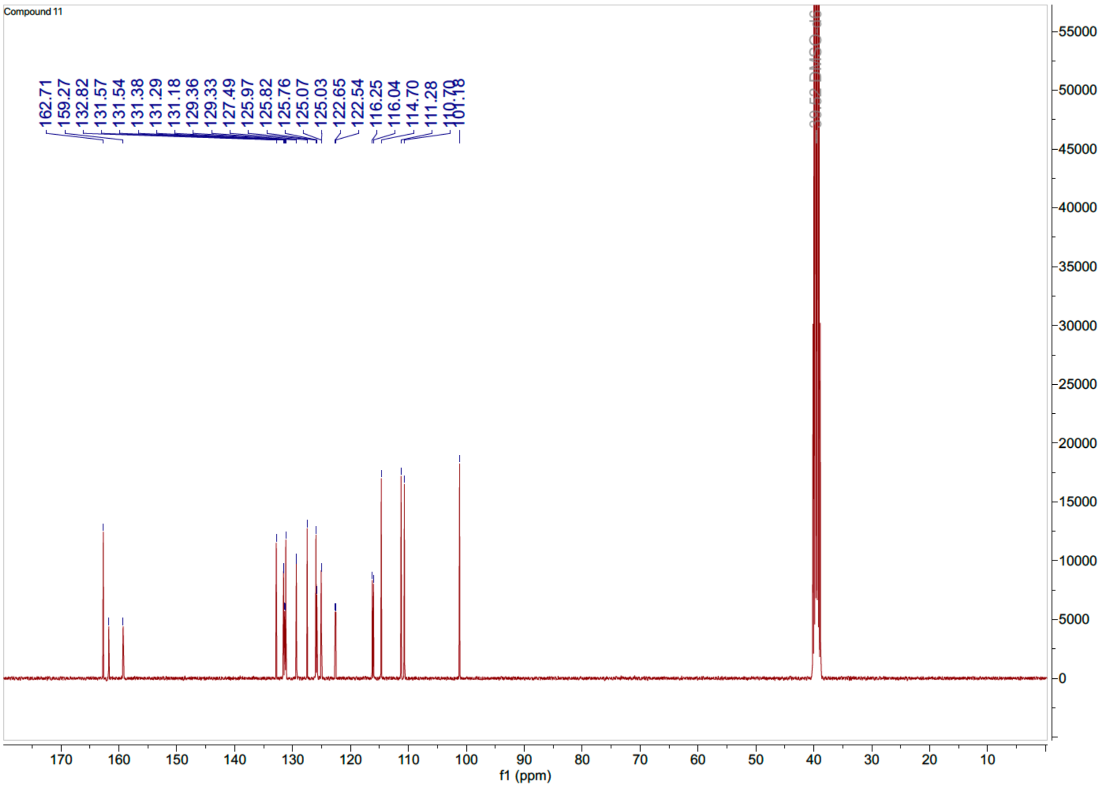

(*E*)-3-(3-fluorophenyl)-*N*-(1*H*-indol-5-yl)acrylamide (12)

^{1}H NMR (400 MHz, DMSO)

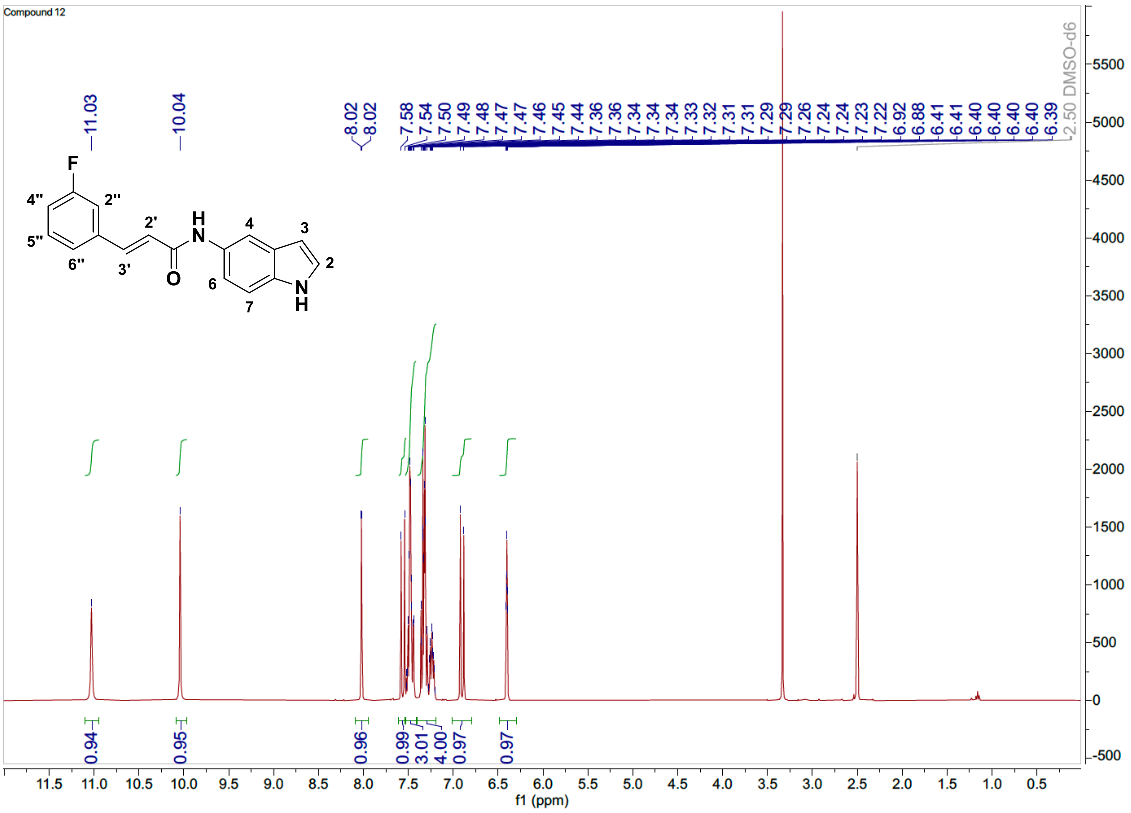

^{13}C NMR (100 MHz, DMSO)

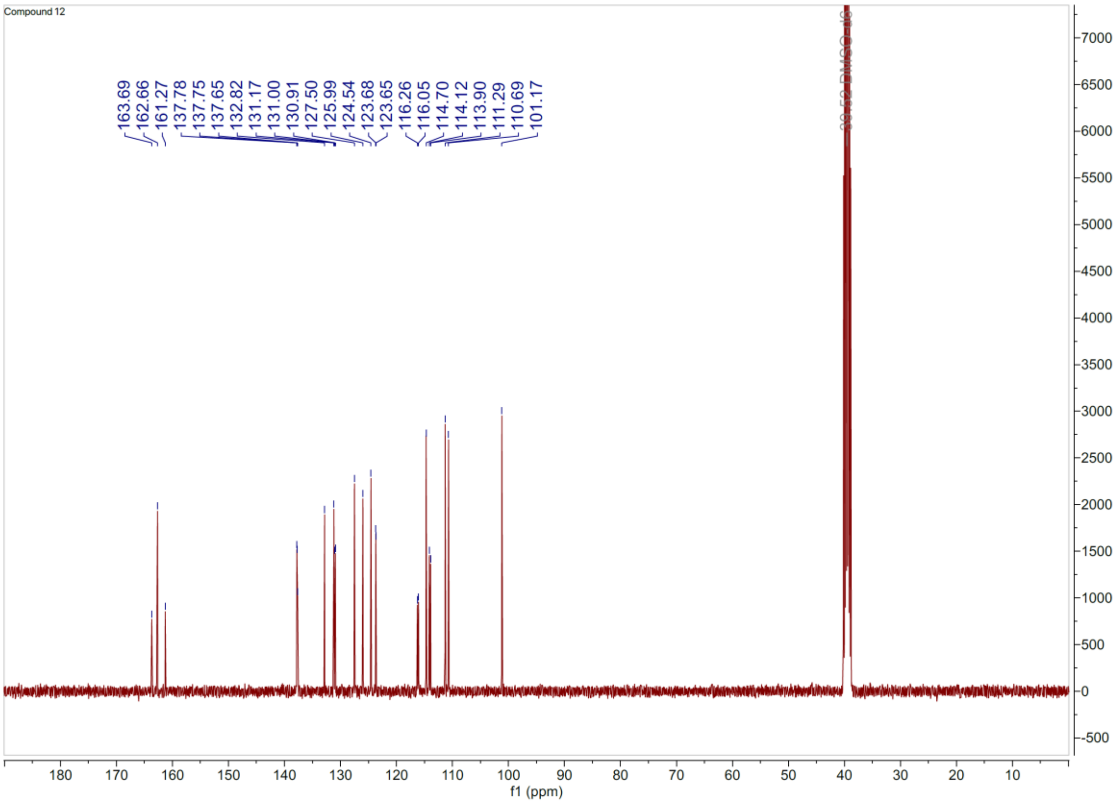

(*E*)-3-(4-fluorophenyl)-*N*-(1*H*-indol-5-yl)acrylamide (13)

^{1}H NMR (400 MHz, DMSO)

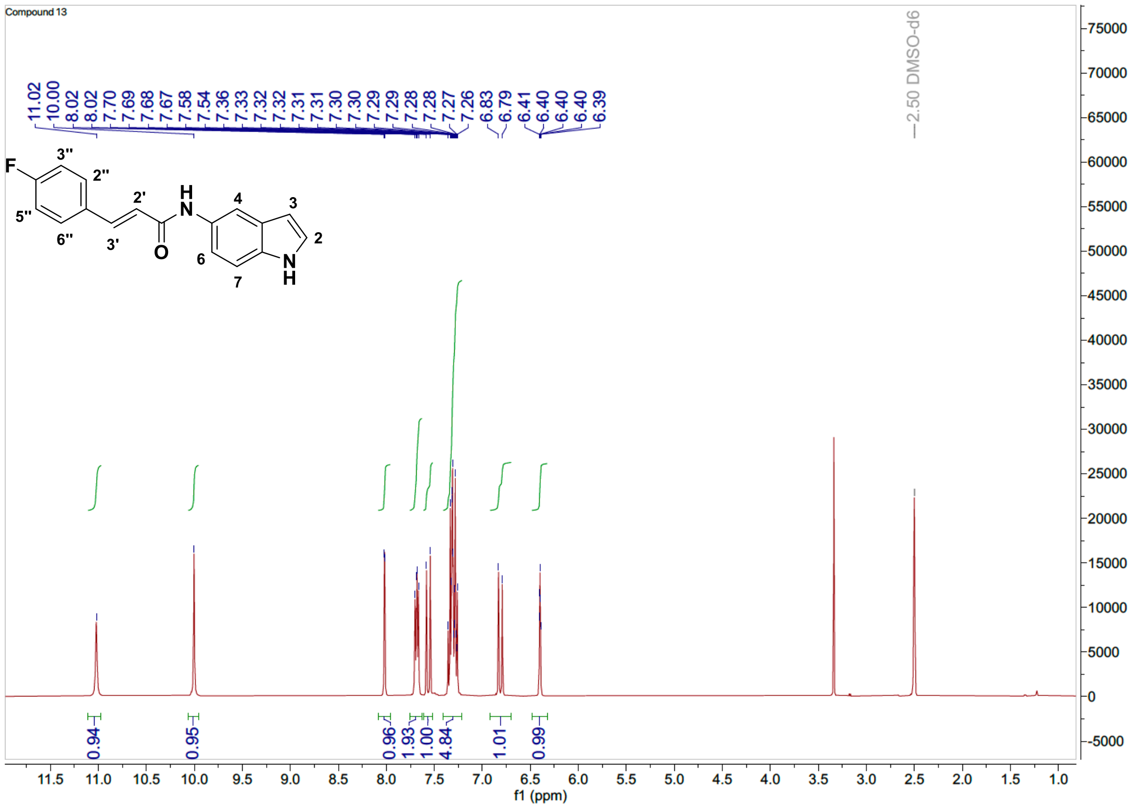

^{13}C NMR (100 MHz, DMSO)

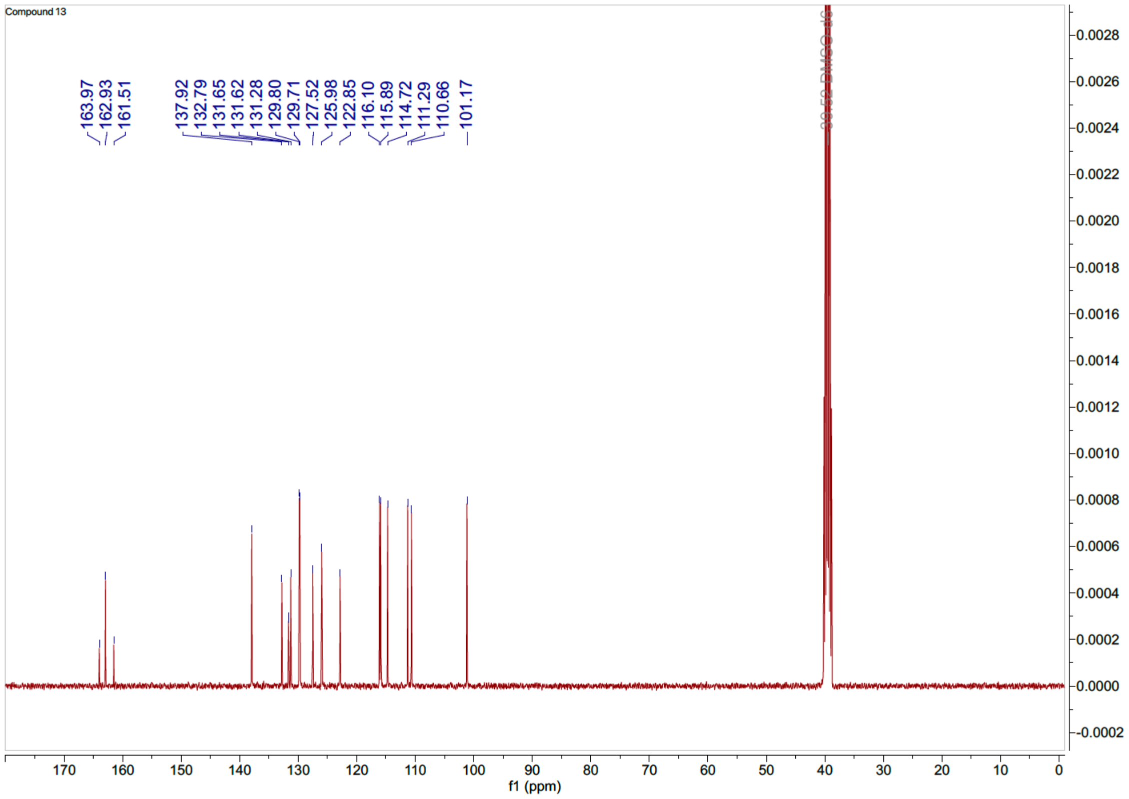

(*E*)-3-(2,3-difluorophenyl)-*N*-(1*H*-indol-5-yl)acrylamide (14)

^{1}H NMR (400 MHz, DMSO)

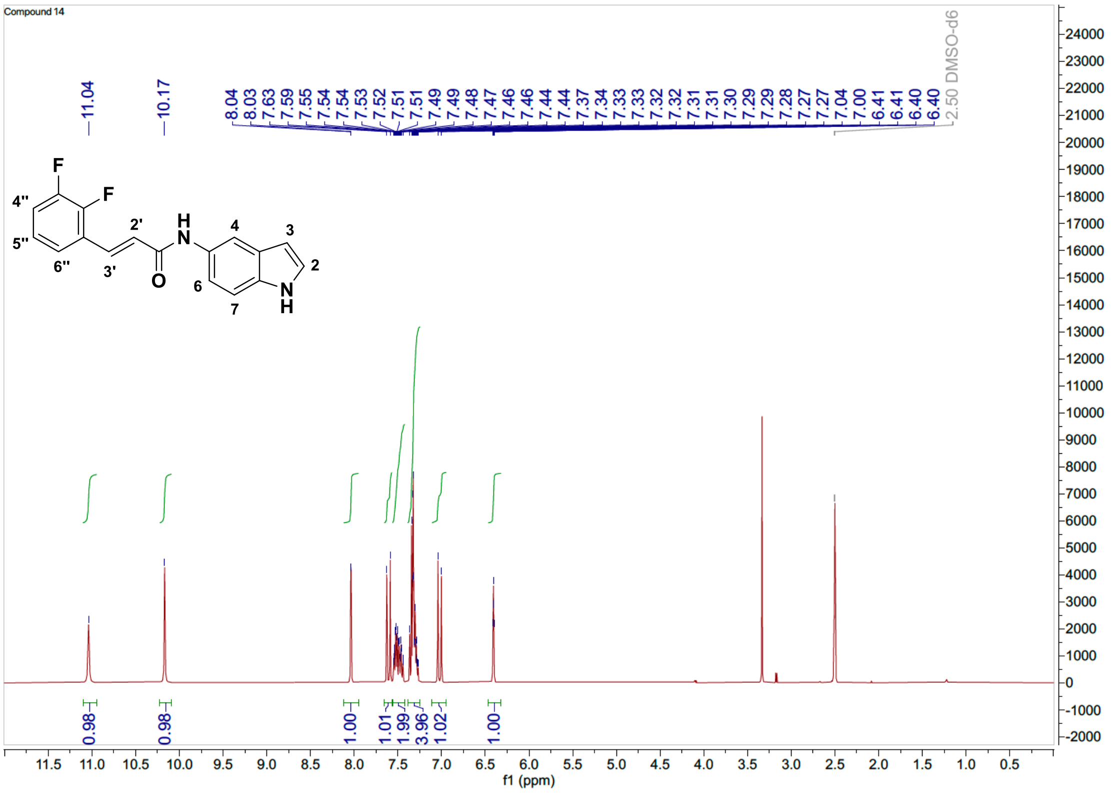

^{13}C NMR (100 MHz, DMSO)

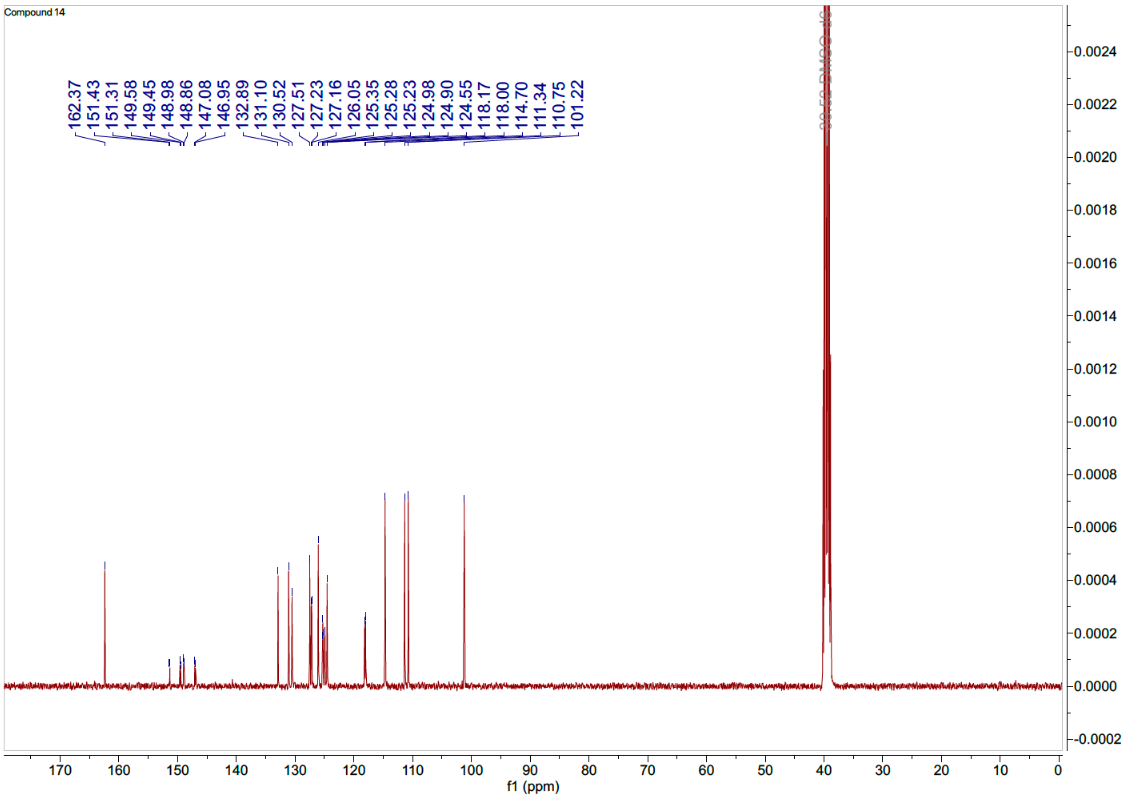

(*E*)-3-(2,4-difluorophenyl)-*N*-(1*H*-indol-5-yl)acrylamide (15)

^{1}H NMR (400 MHz, DMSO)

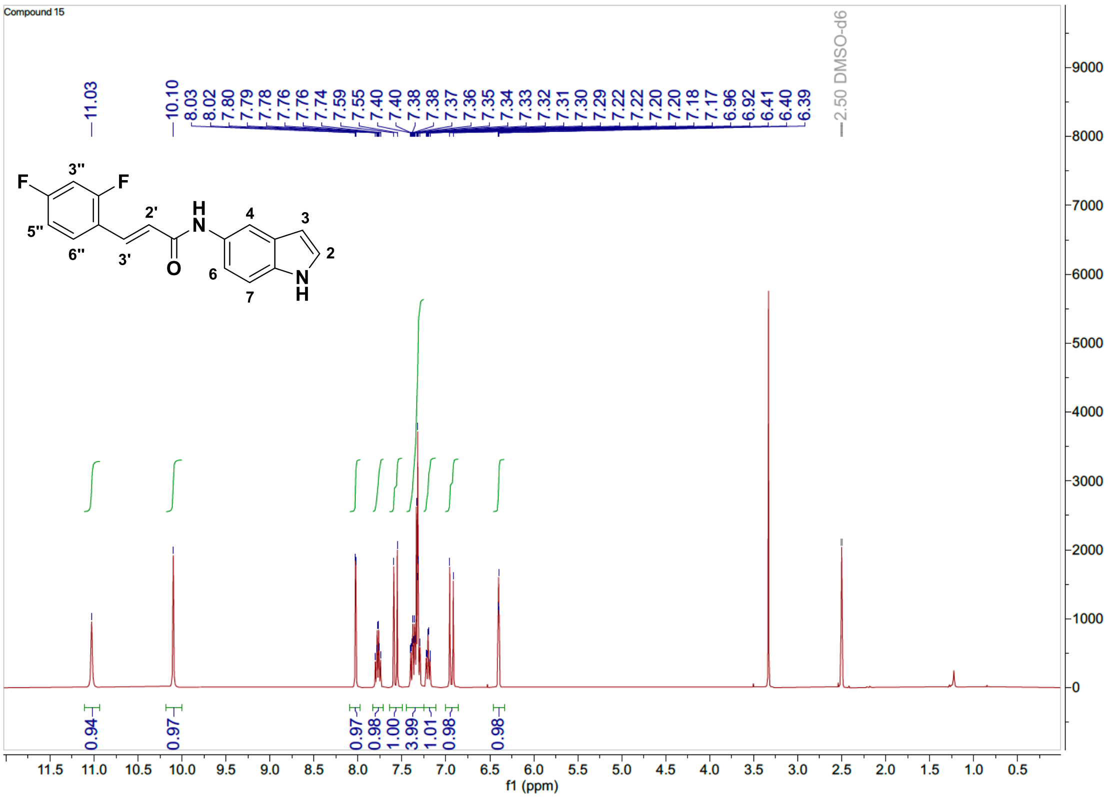

^{13}C NMR (100 MHz, DMSO)

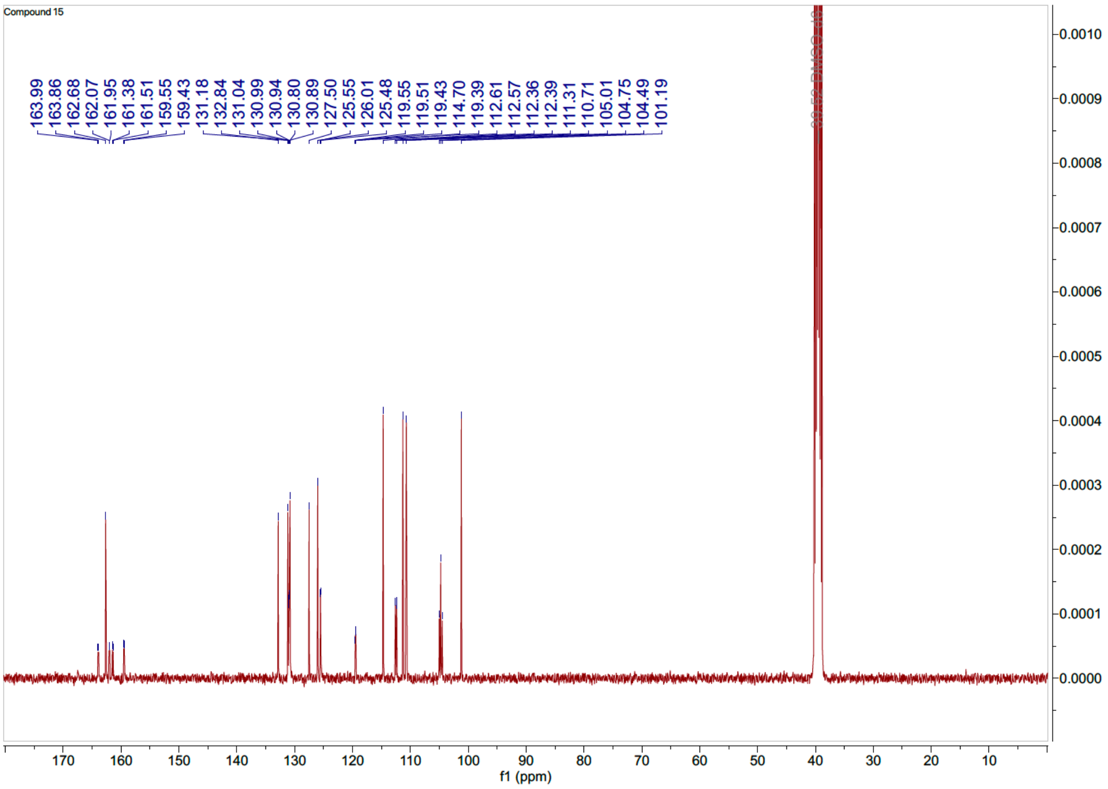

(*E*)-3-(2,5-difluorophenyl)-*N*-(1*H*-indol-5-yl)acrylamide (16)

^{1}H NMR (400 MHz, DMSO)

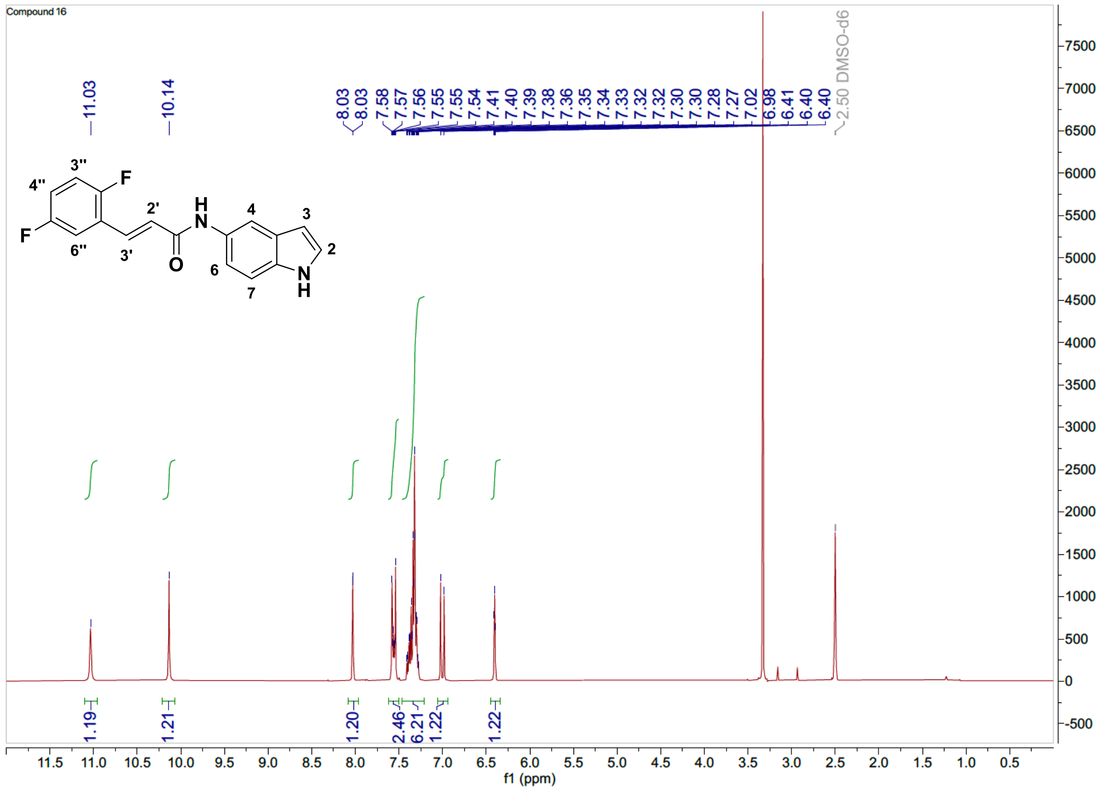

^{13}C NMR (100 MHz, DMSO)

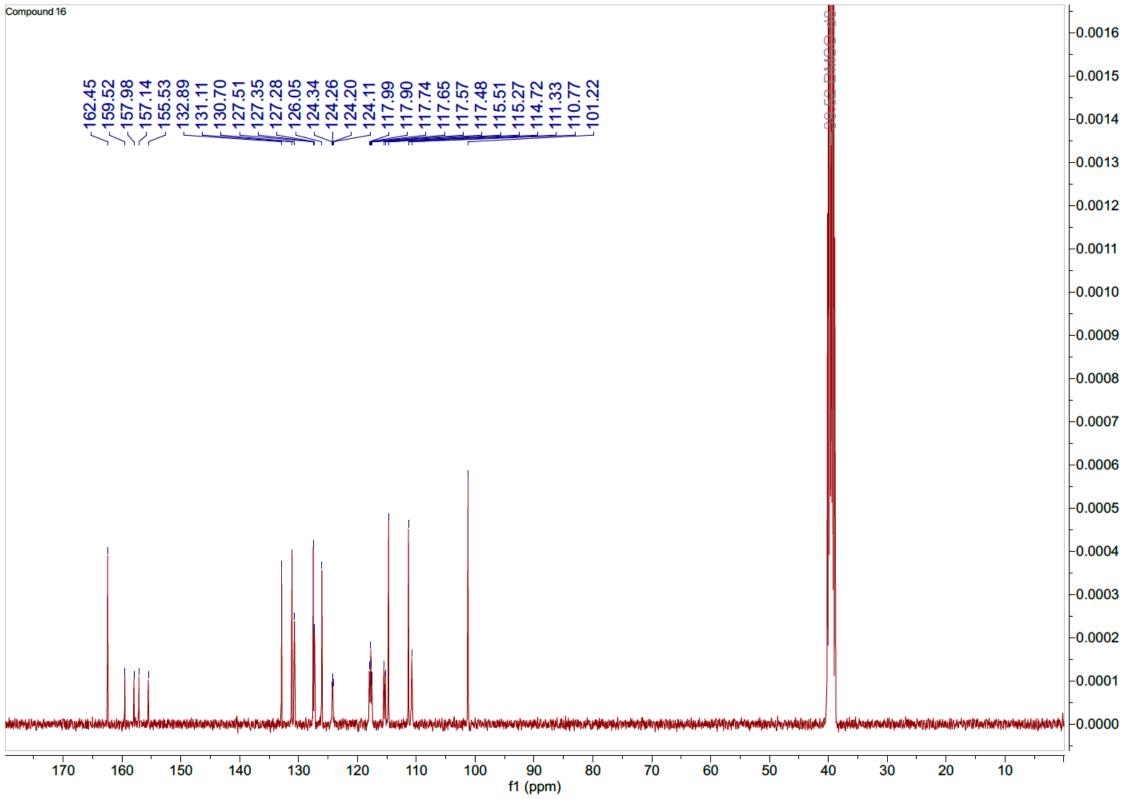

(*E*)-3-(2,6-difluorophenyl)-*N*-(1*H*-indol-5-yl)acrylamide (17)

^{1}H NMR (400 MHz, DMSO)

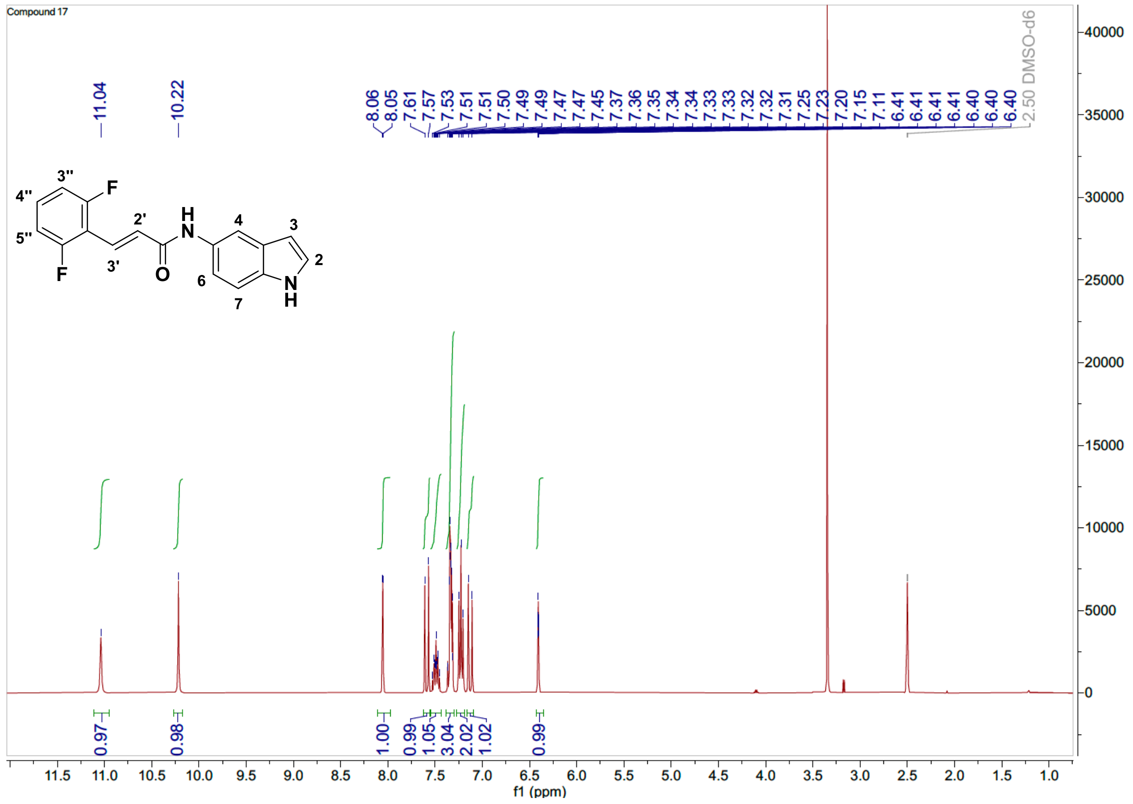

^{13}C NMR (100 MHz, DMSO)

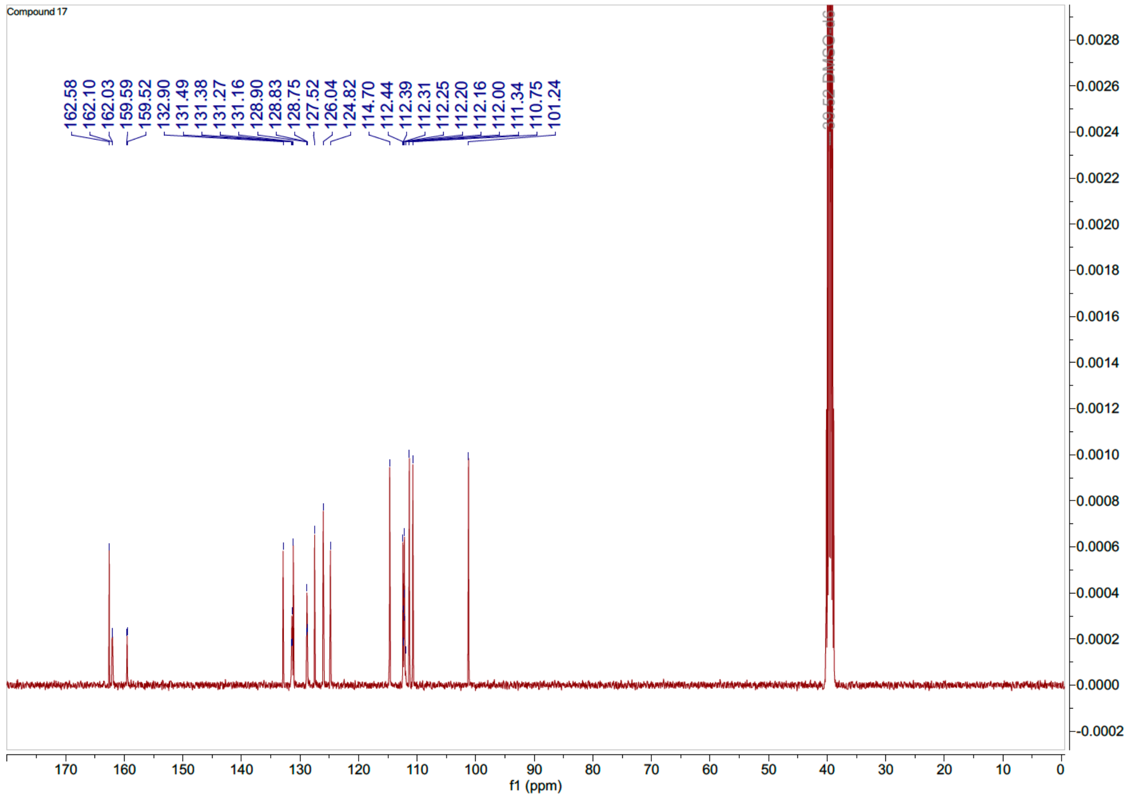

(*E*)-3-(3,4-difluorophenyl)-*N*-(1*H*-indol-5-yl)acrylamide (18)

^{1}H NMR (400 MHz, DMSO)

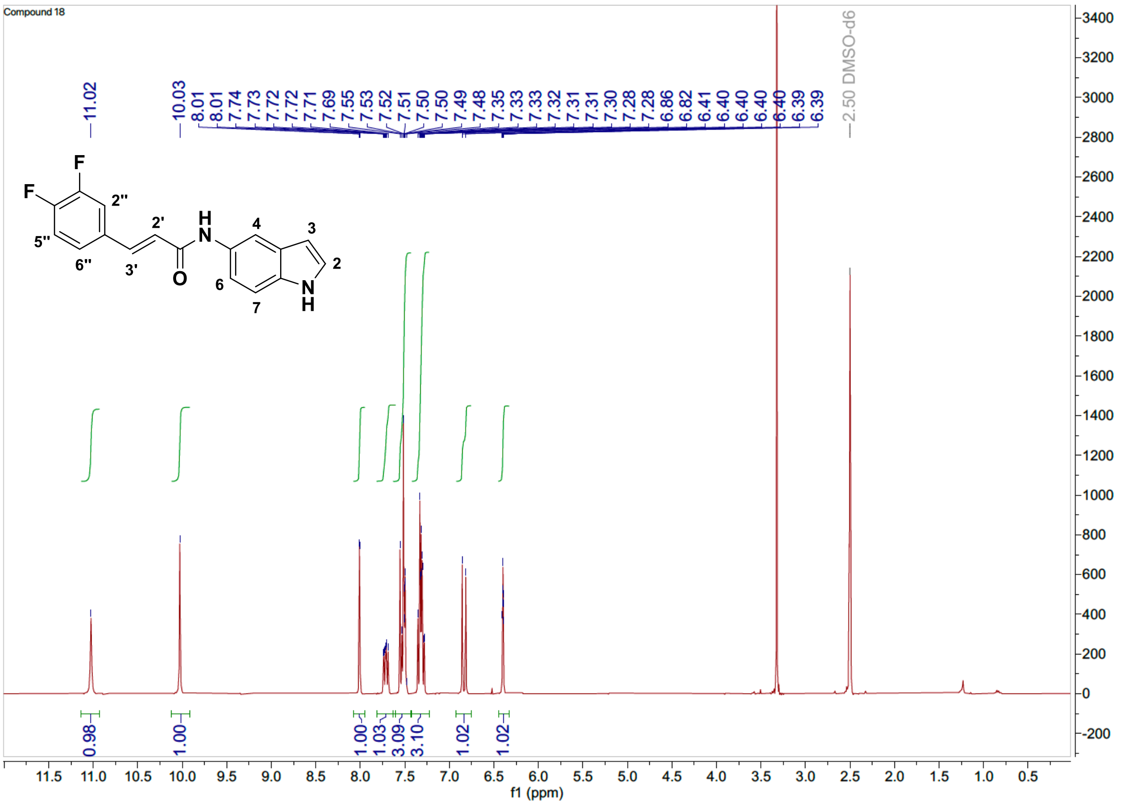

^{13}C NMR (100 MHz, DMSO)

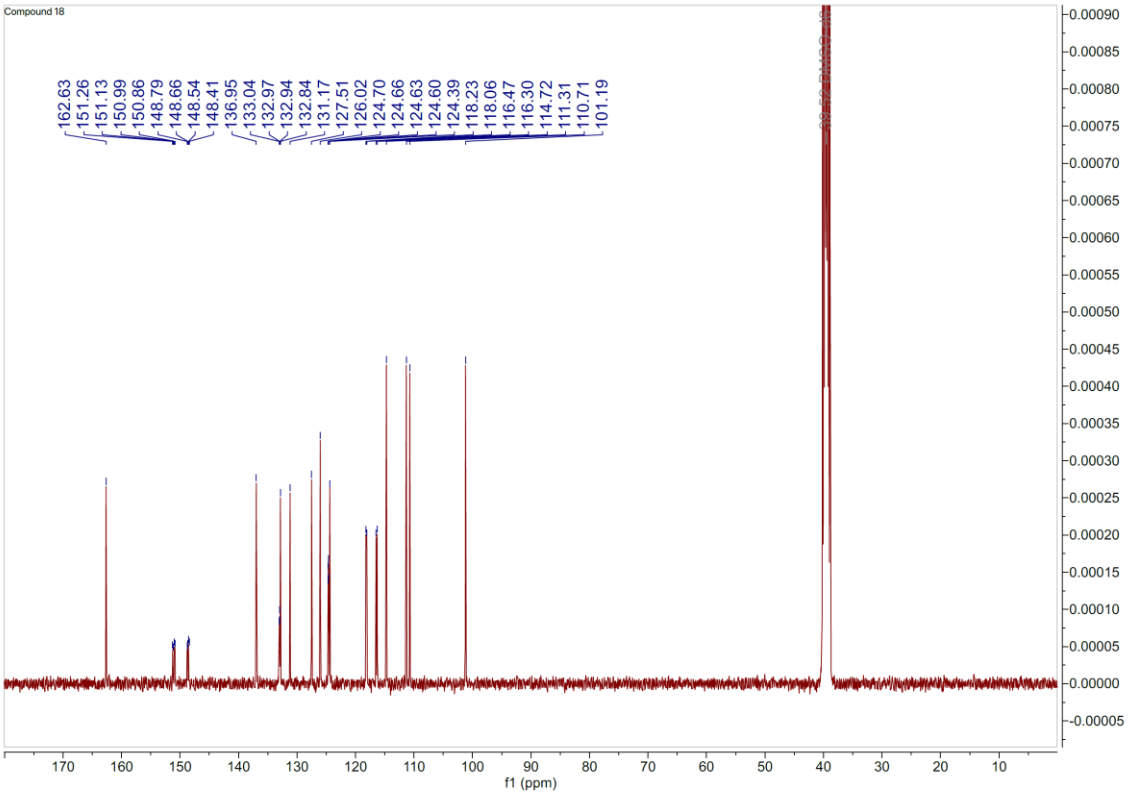

(*E*)-3-(3,5-difluorophenyl)-*N*-(1*H*-indol-5-yl)acrylamide (19)

^{1}H NMR (400 MHz, DMSO)

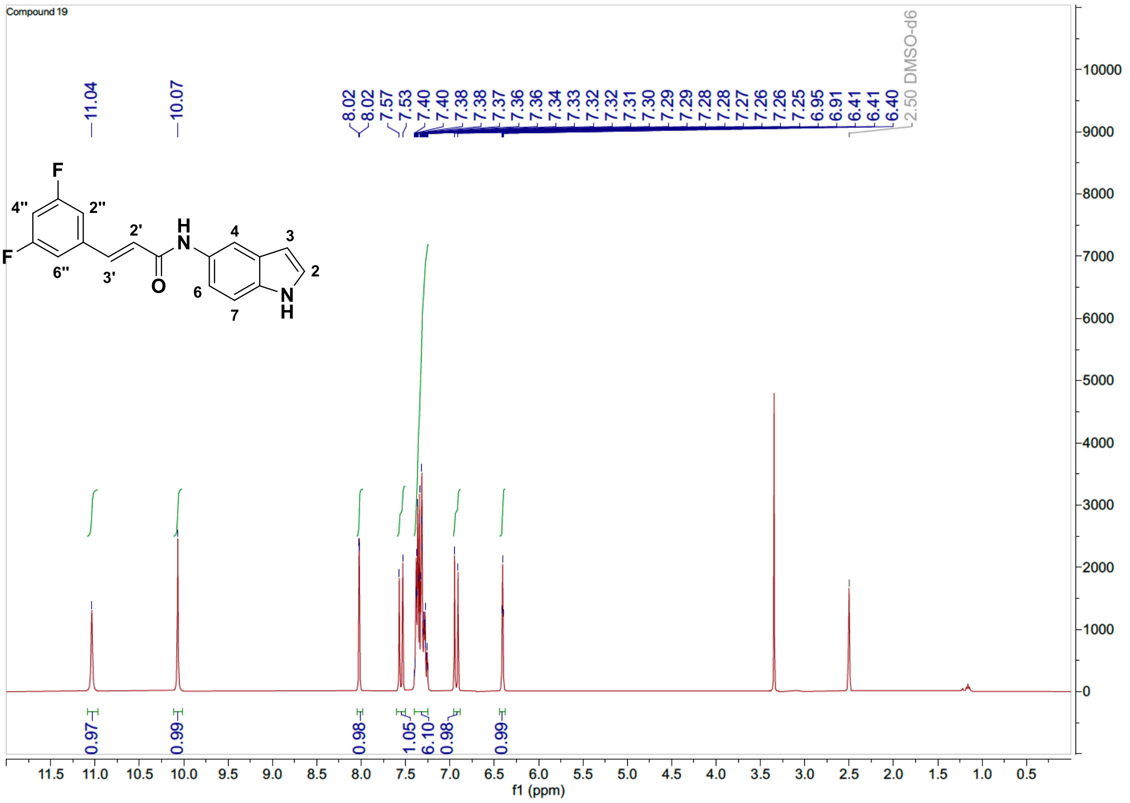

^{13}C NMR (100 MHz, DMSO)

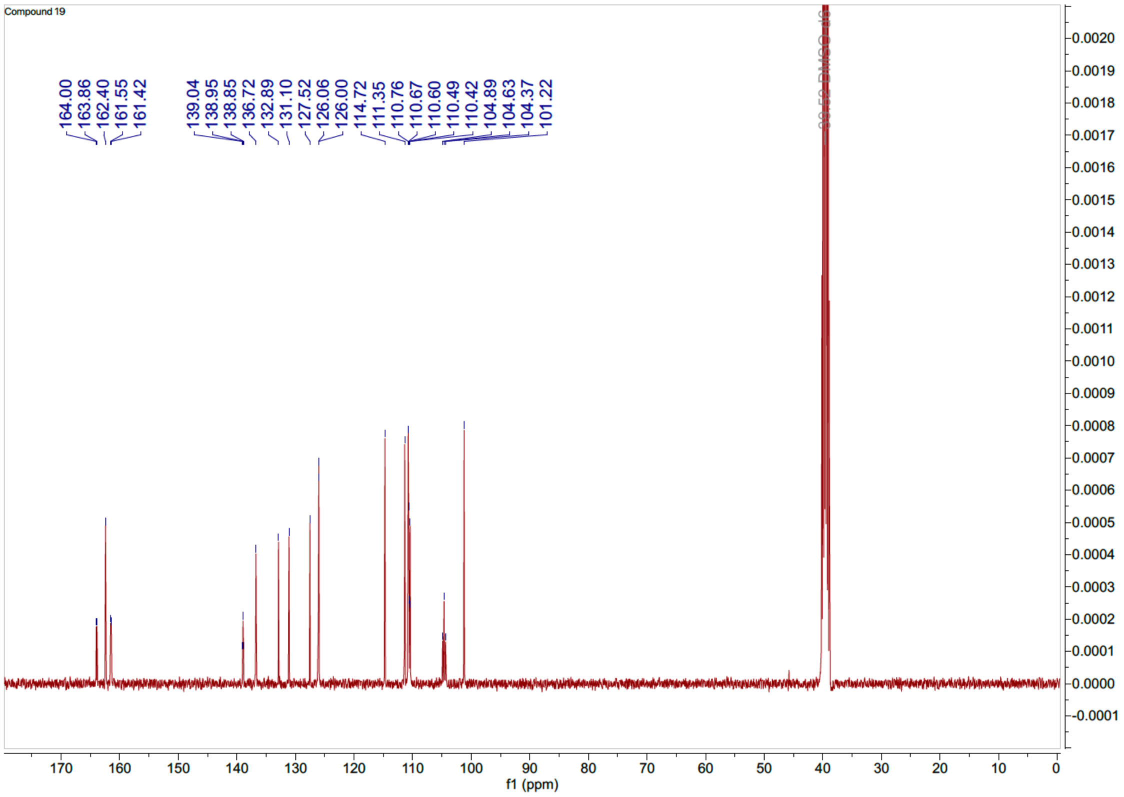

(*E*)-*N*-(1*H*-indol-5-yl)-3-(2-(trifluoromethyl)phenyl)acrylamide (20)

^{1}H NMR (400 MHz, DMSO)

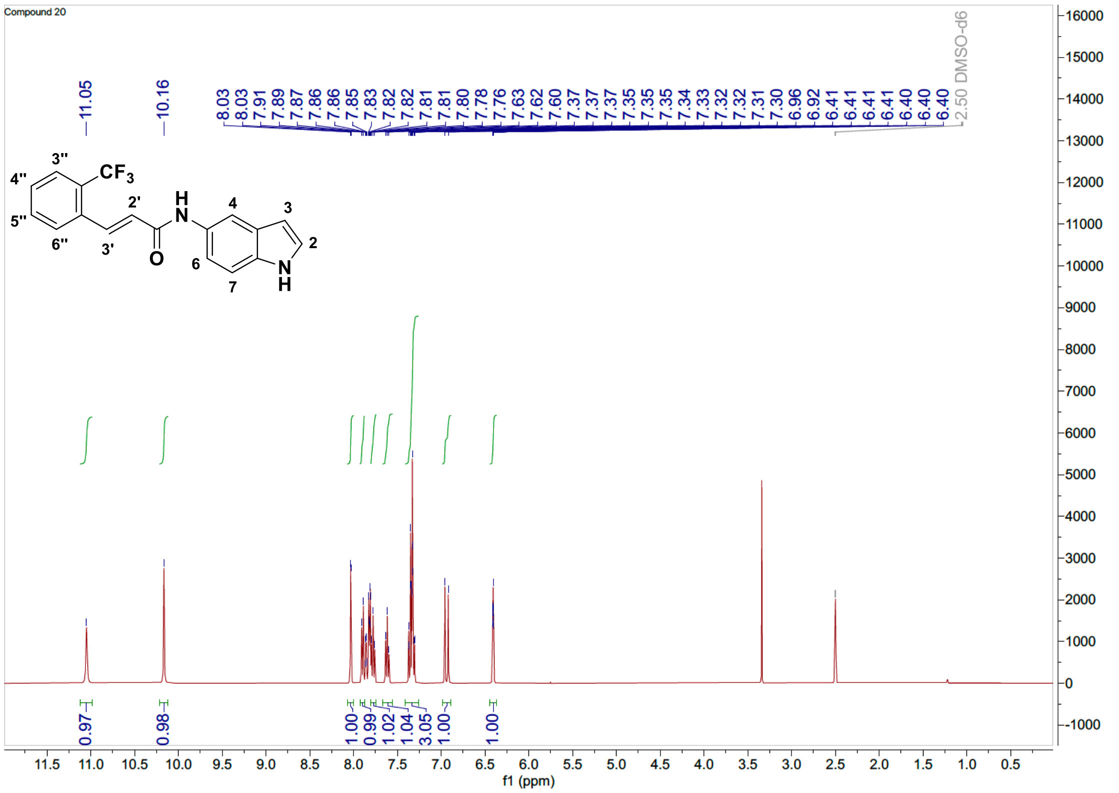

^{13}C NMR (100 MHz, DMSO)

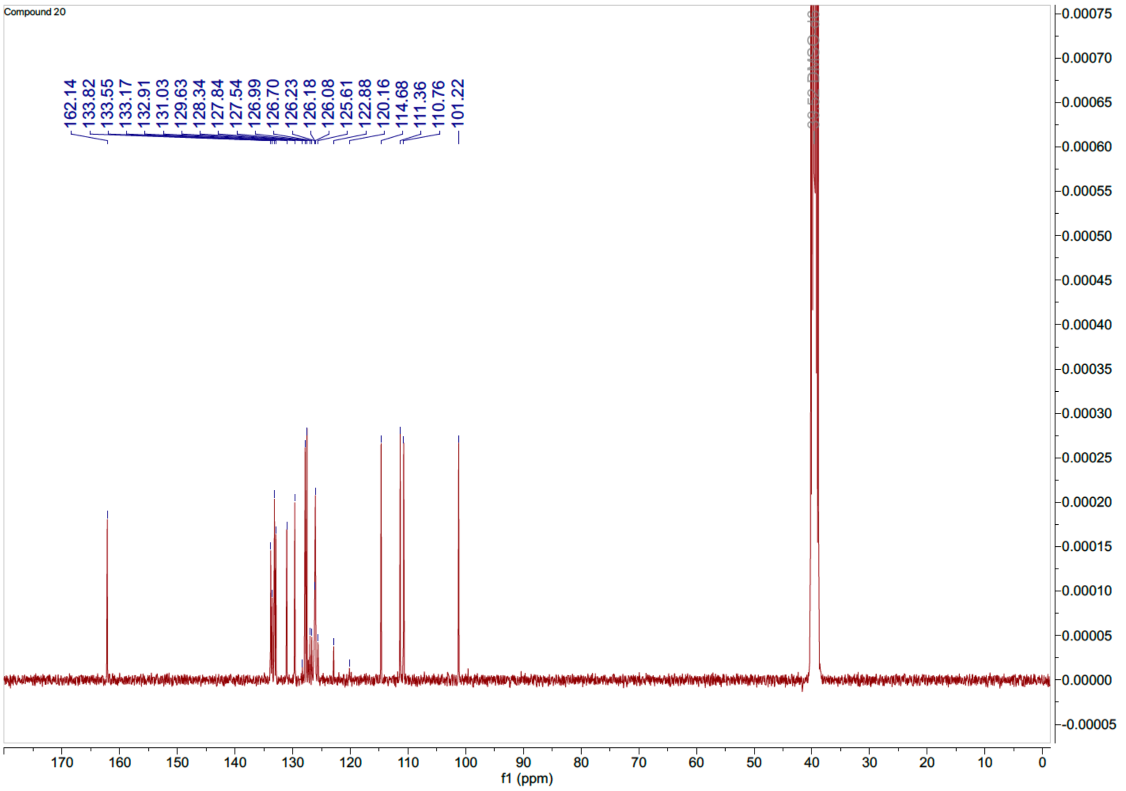

(*E*)-*N*-(1*H*-indol-5-yl)-3-(3-(trifluoromethyl)phenyl)acrylamide (21)

^{1}H NMR (400 MHz, DMSO)

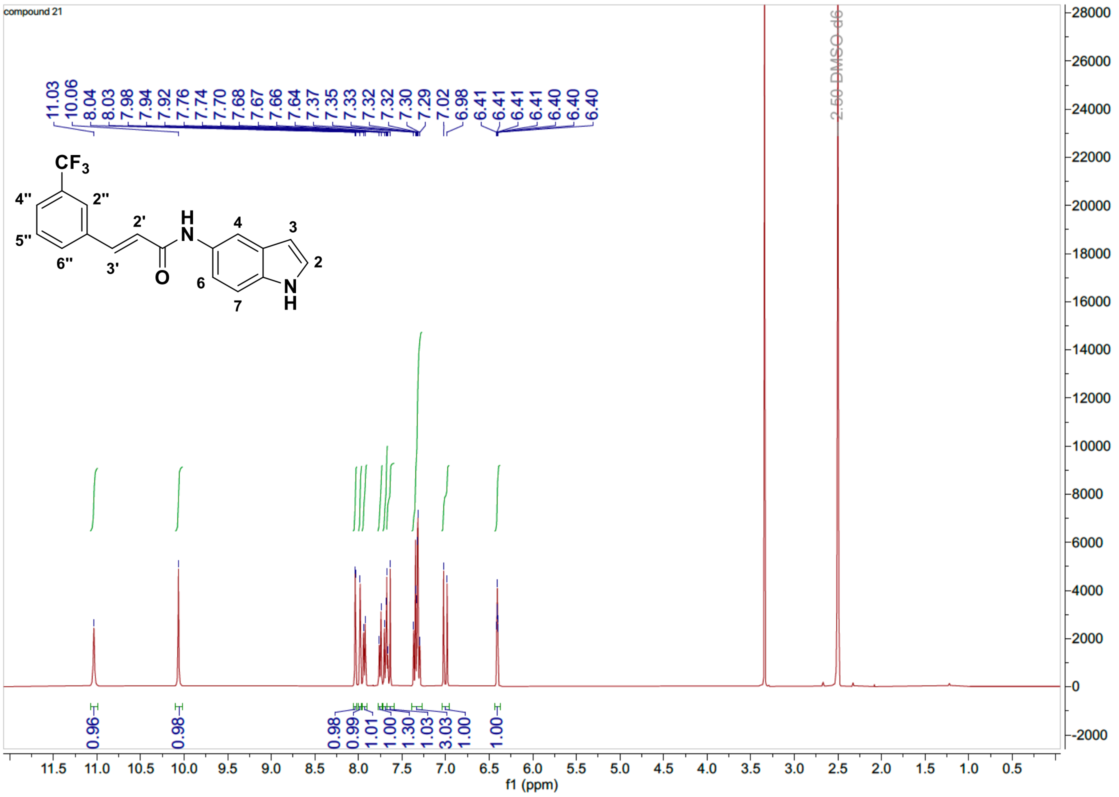

^{13}C NMR (100 MHz, DMSO)

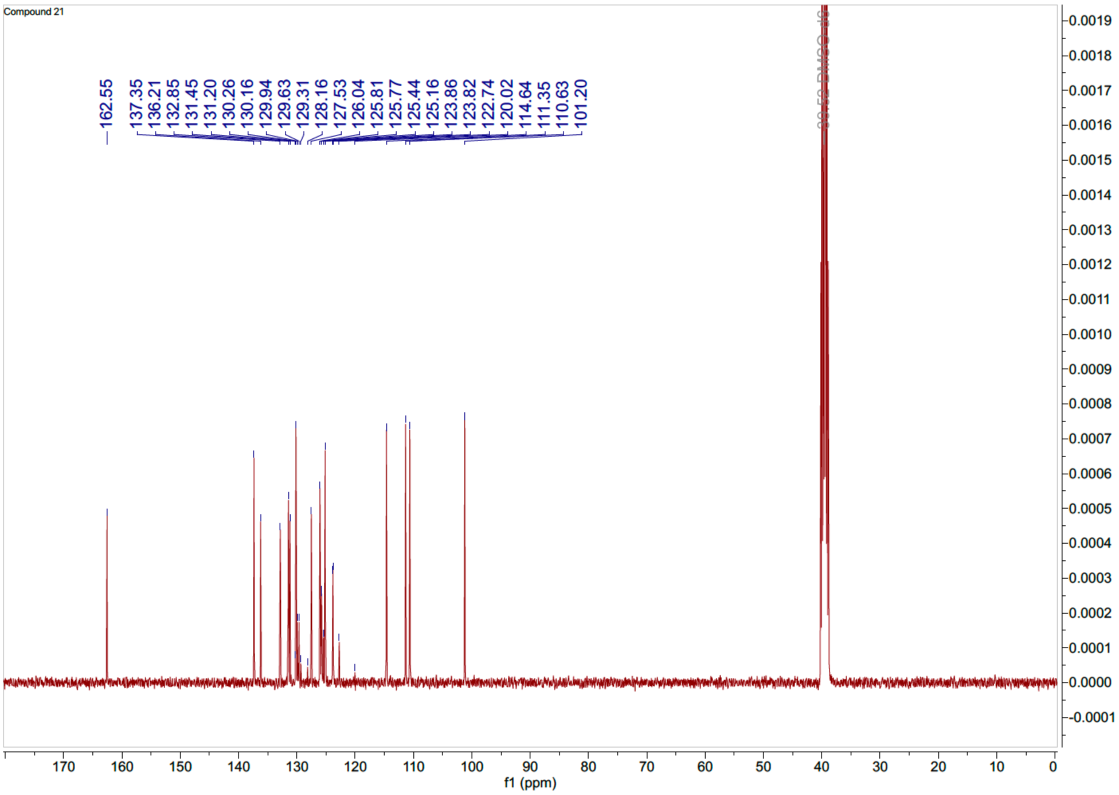

2,2,2-trifluoro-1-(5-nitro-1*H*-indol-3-yl)ethan-1-one (23)

^{1}H NMR (500 MHz, DMSO)

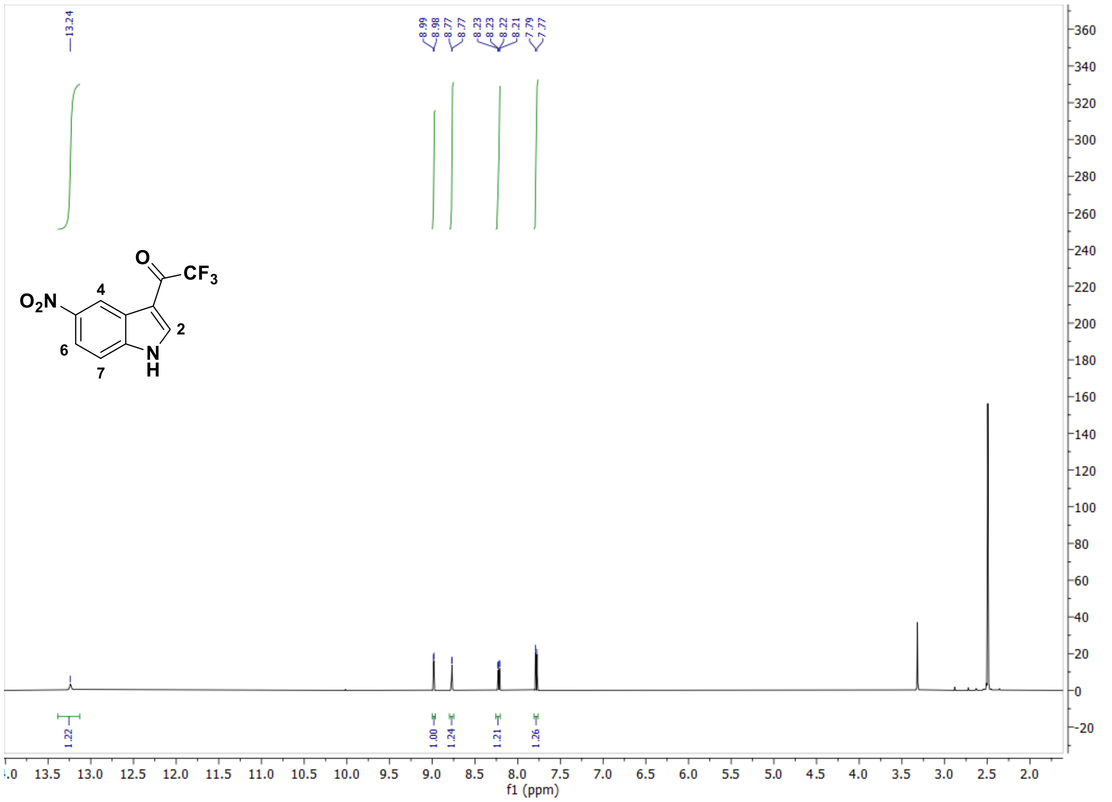

^{13}C NMR (125 MHz, DMSO)

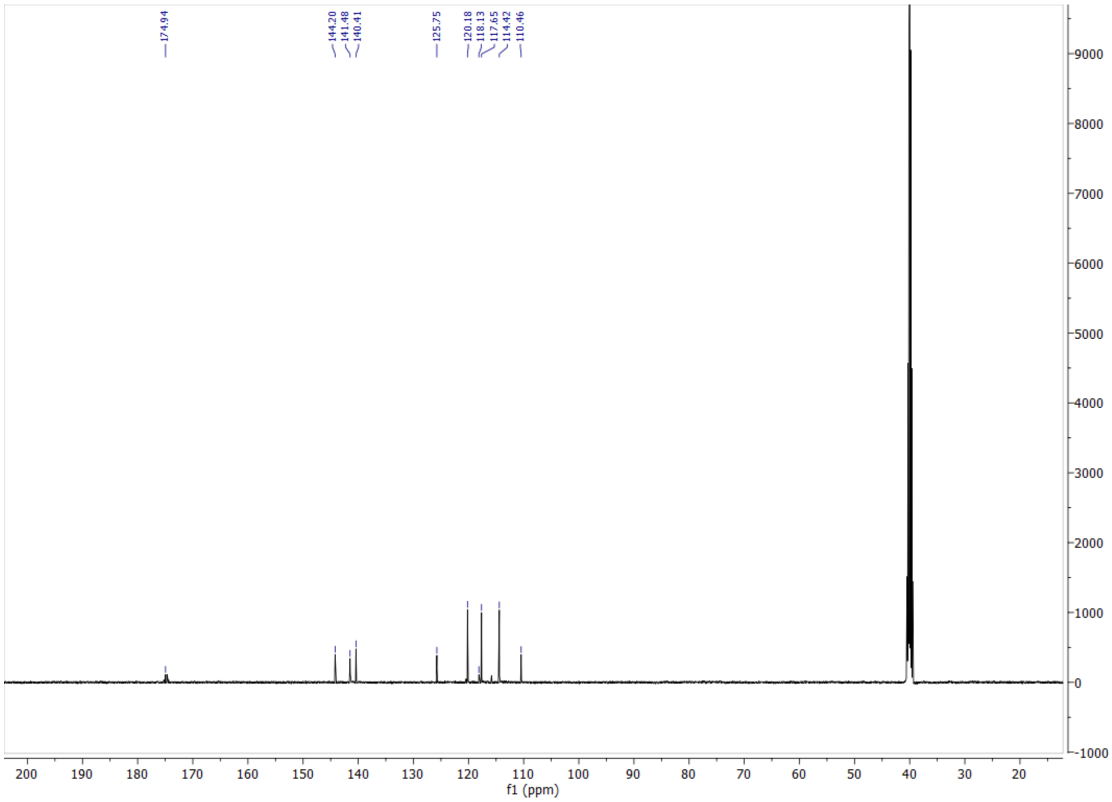

5-nitro-1*H*-indole-3-carboxylic acid (24)

^{1}H NMR (500 MHz, DMSO)

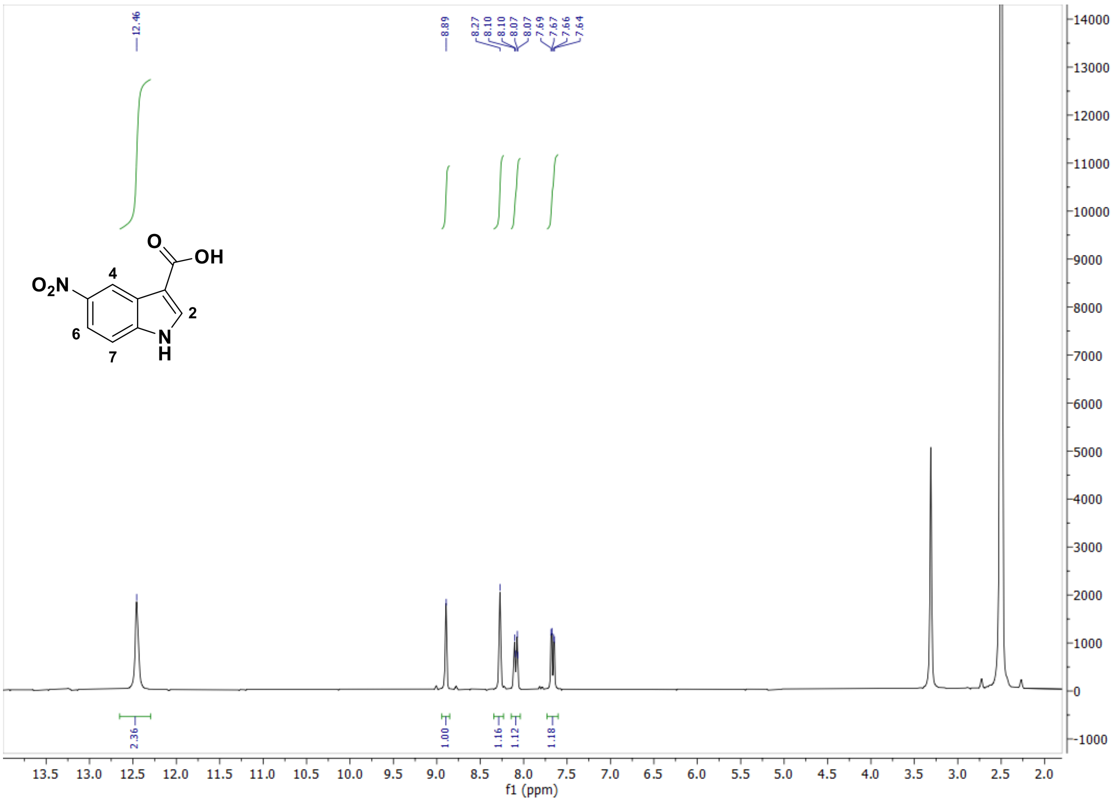

^{13}C NMR (125 MHz, DMSO)

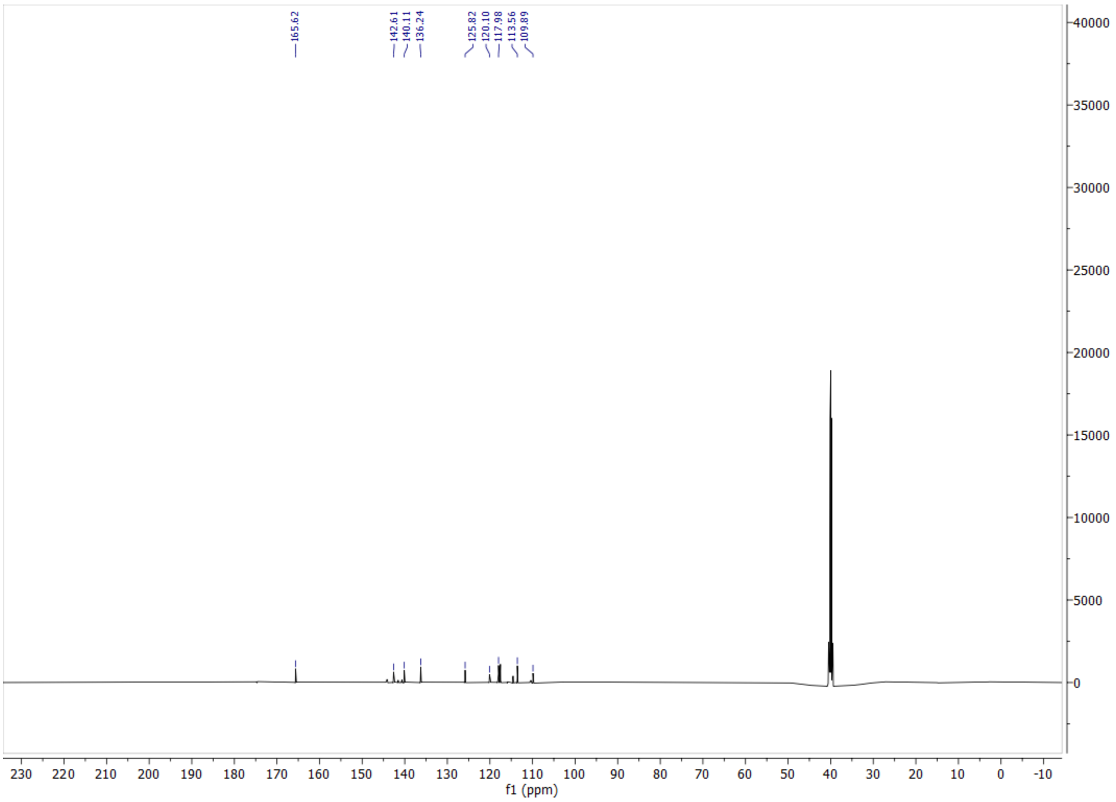

5-nitro-1*H*-indole-3-carboxamide (25)

^{1}H NMR (500 MHz, DMSO)

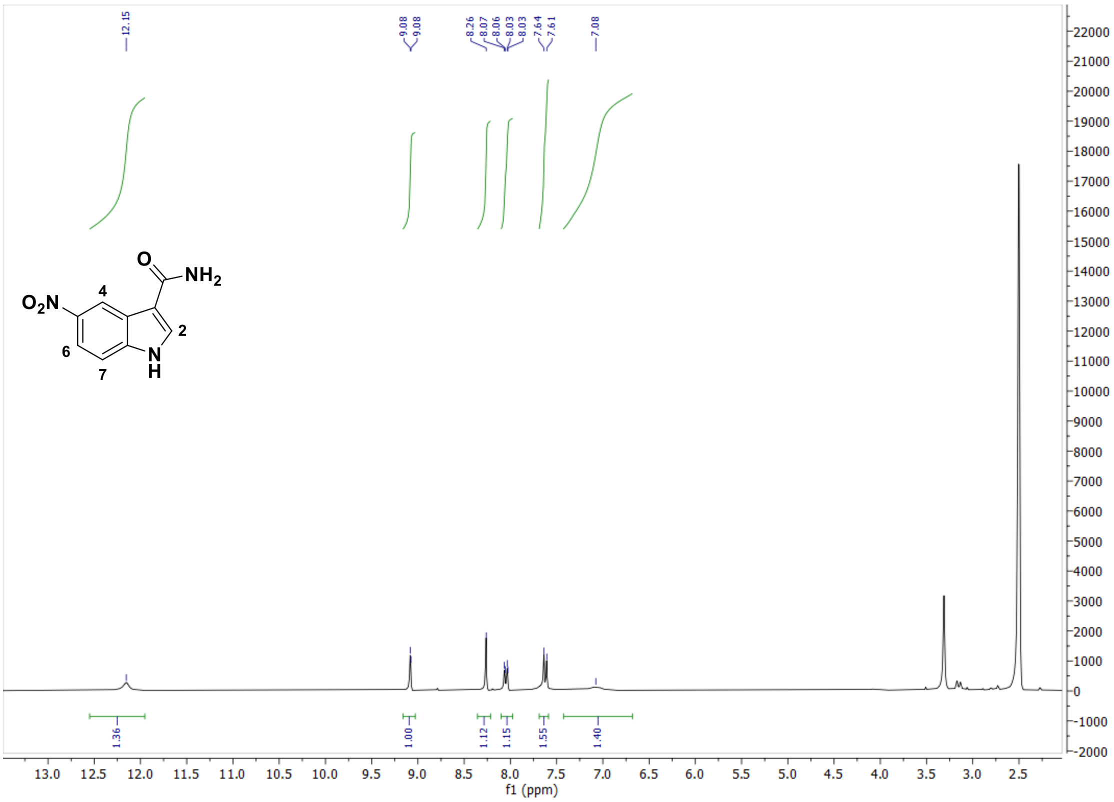

^{13}C NMR (125 MHz, DMSO)

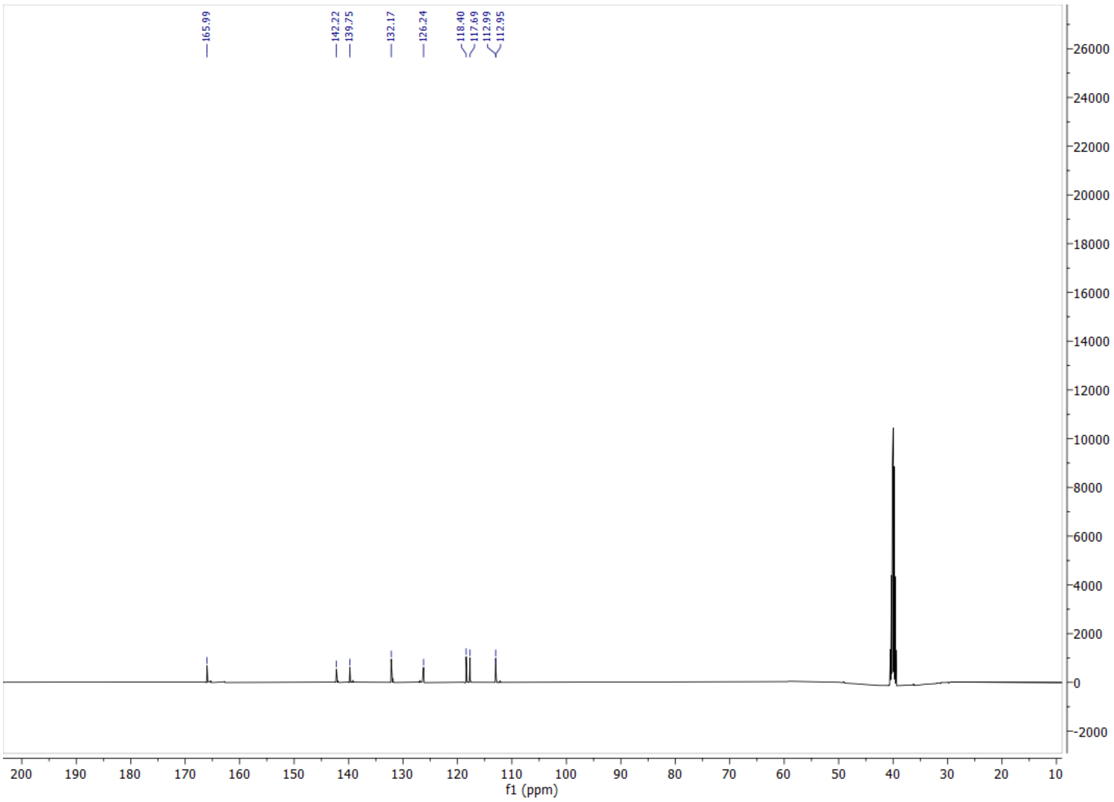

5-amino-1*H*-indole-3-carboxamide (26)

^{1}H NMR (500 MHz, DMSO)

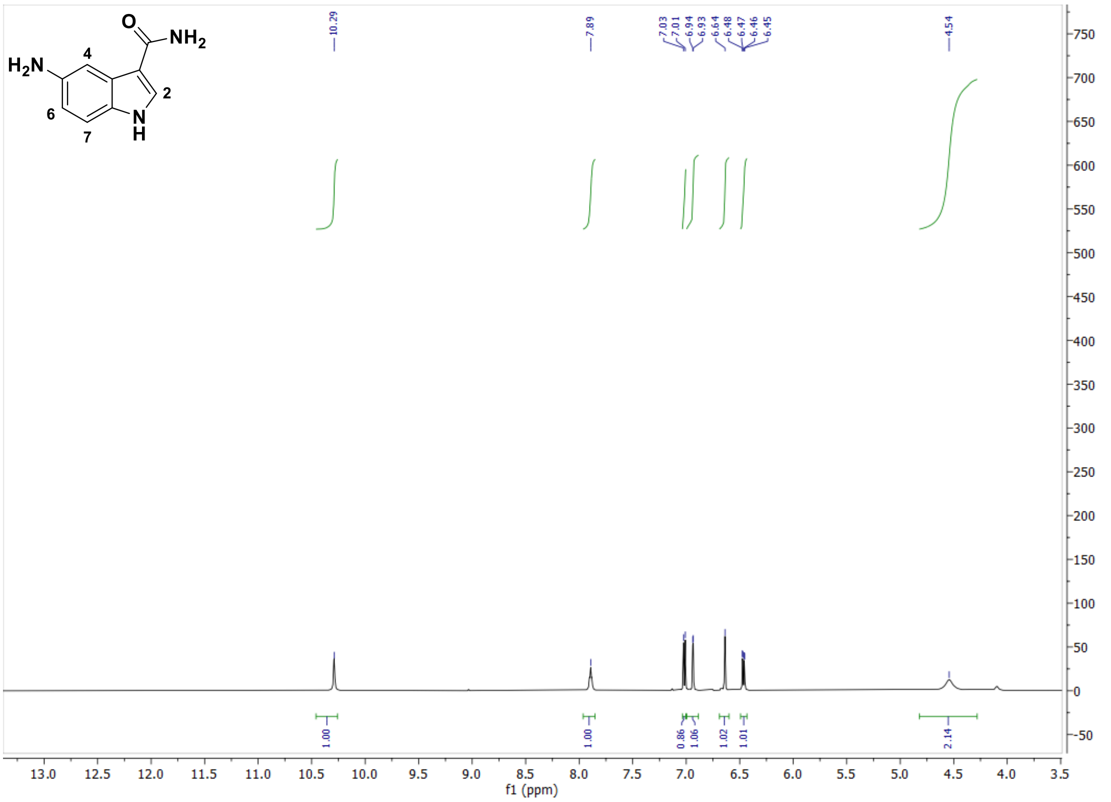

^{13}C NMR (125 MHz, DMSO)

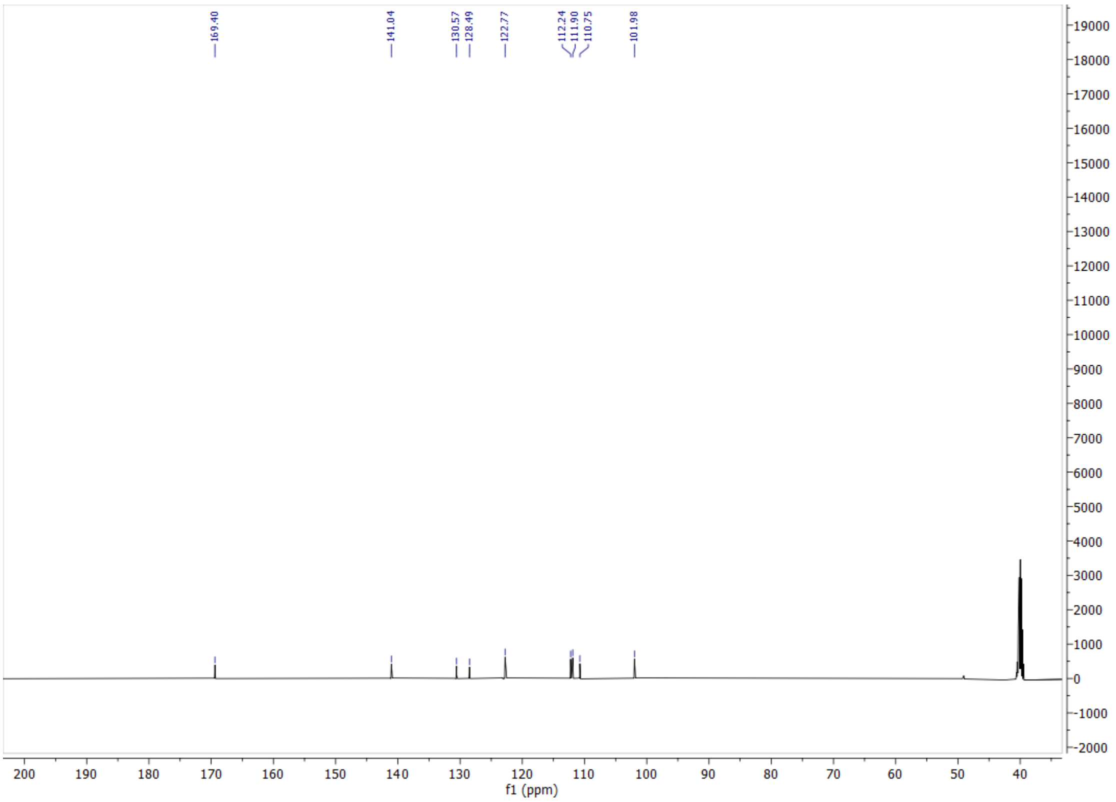

(*E*)-5-(3-(*o*-tolyl)acrylamido)-1*H*-indole-3-carboxamide (27)

^{1}H NMR (400 MHz, DMSO)

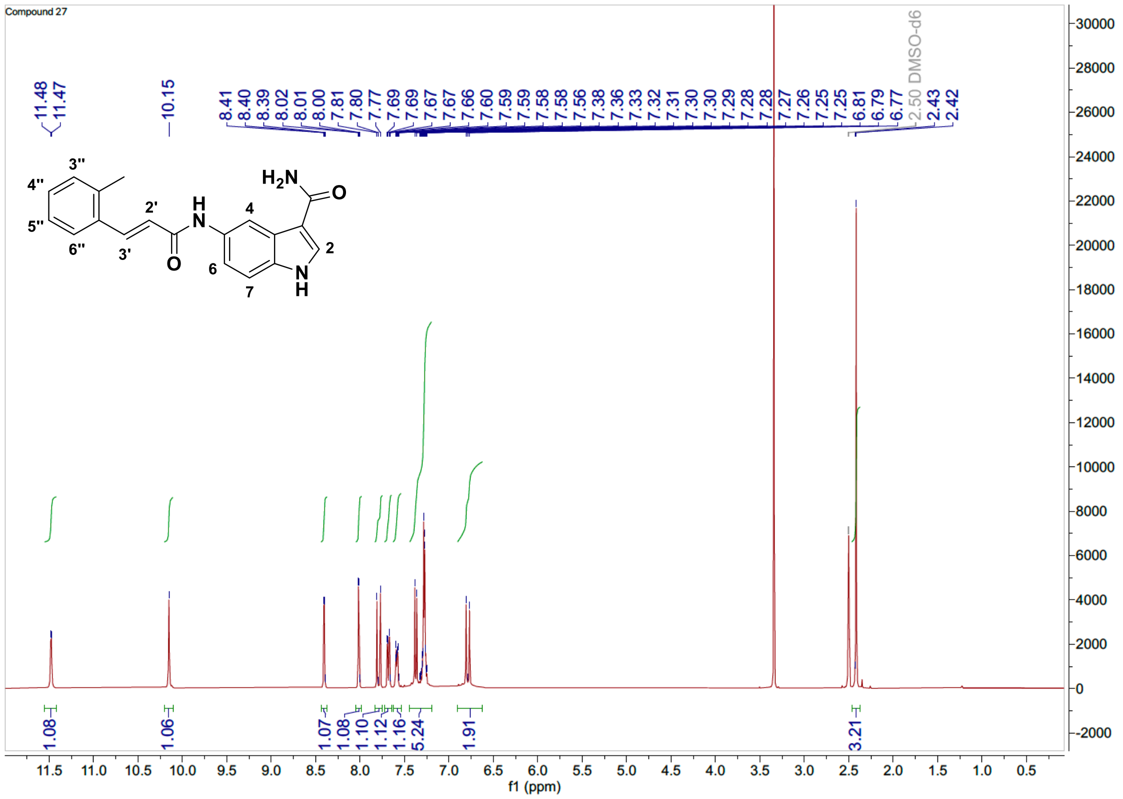

^{13}C NMR (100 MHz, DMSO)

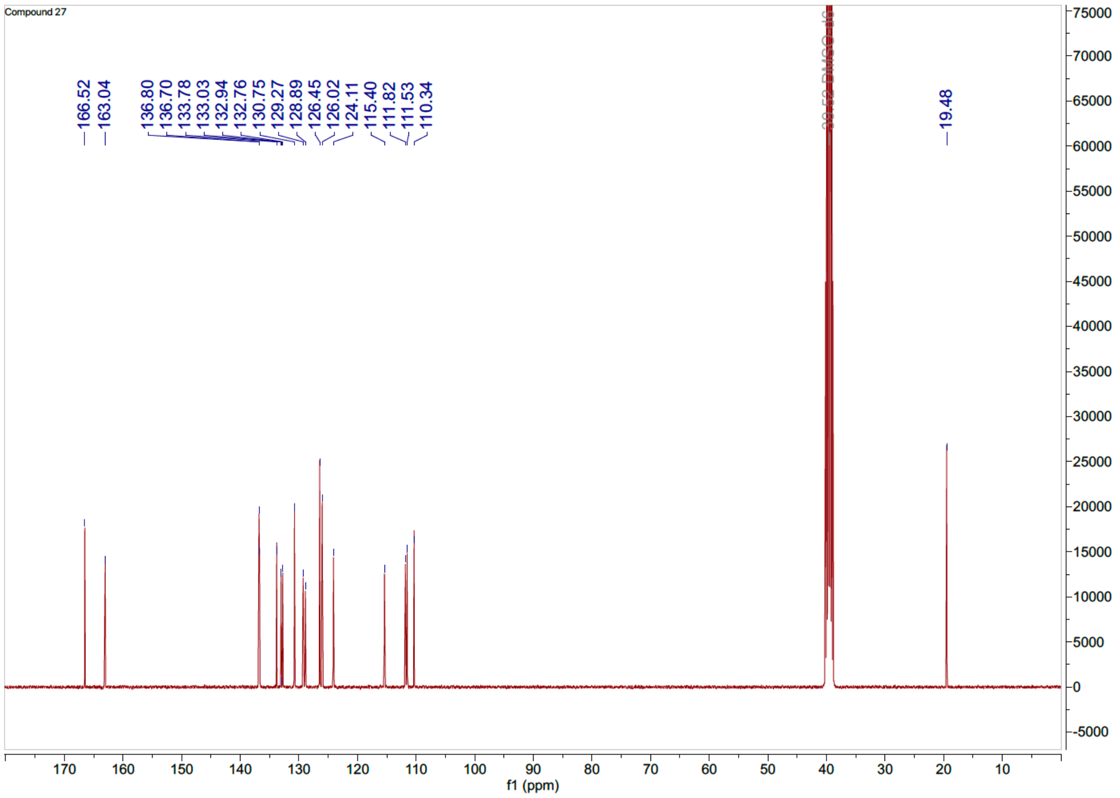

(*E*)-5-(3-(*m*-tolyl)acrylamido)-1*H*-indole-3-carboxamide (28)

^{1}H NMR (400 MHz, DMSO)

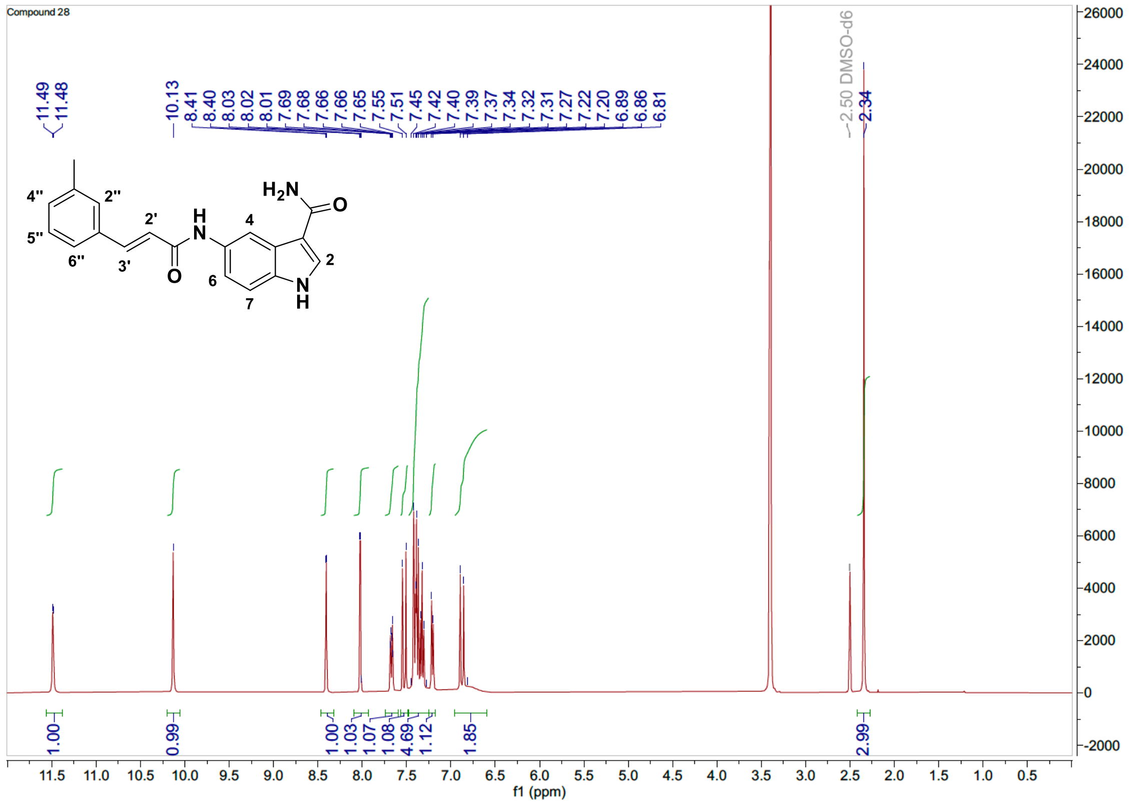

^{13}C NMR (100 MHz, DMSO)

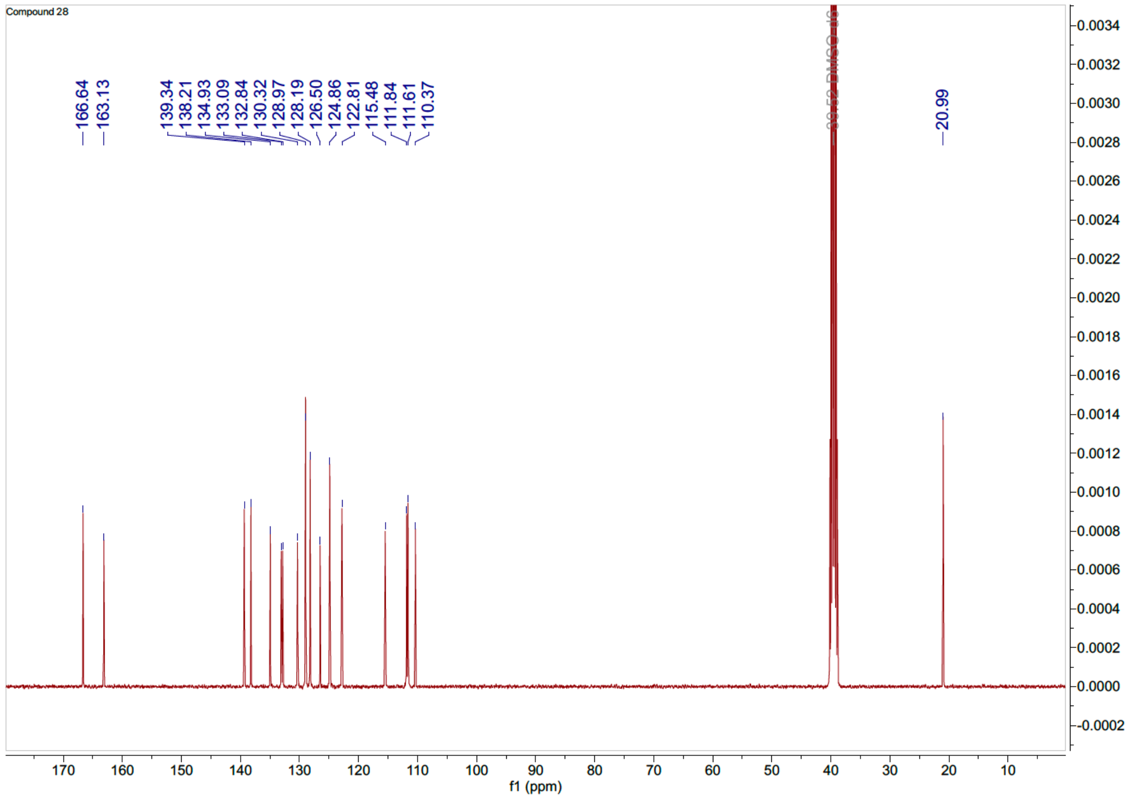

(*E*)-5-(3-(2-chlorophenyl)acrylamido)-1*H*-indole-3-carboxamide (29)

^{1}H NMR (400 MHz, DMSO)

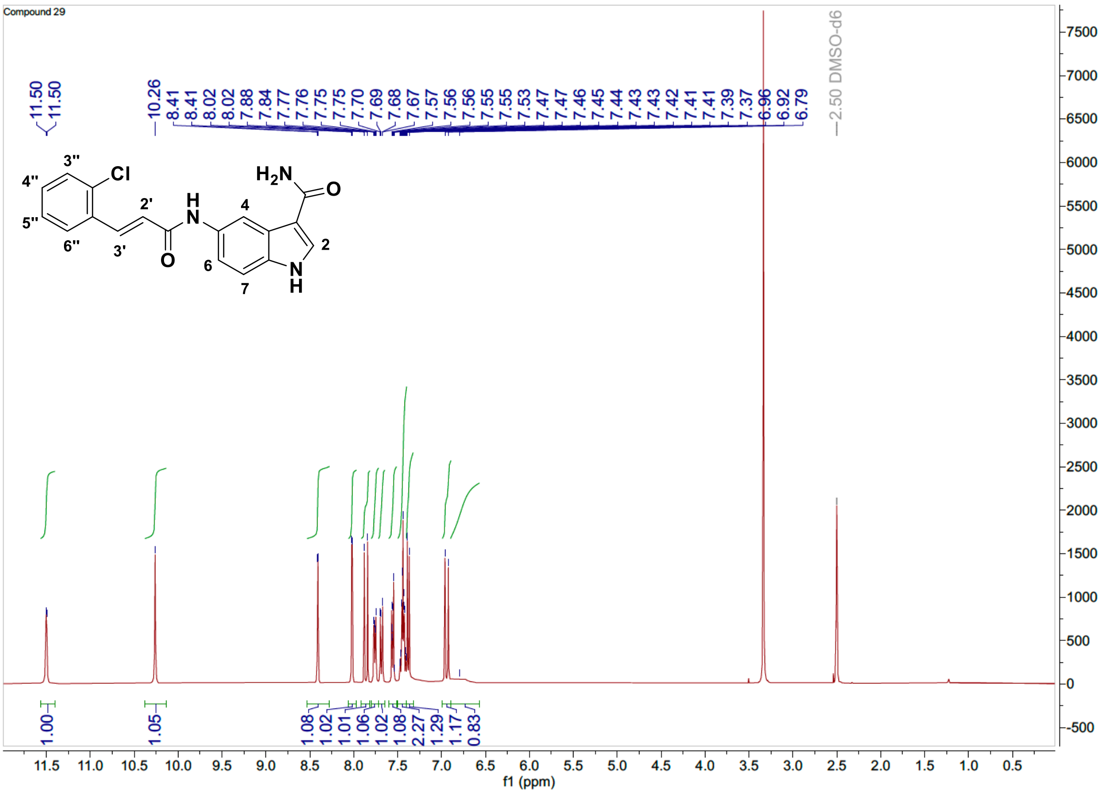

^{13}C NMR (100 MHz, DMSO)

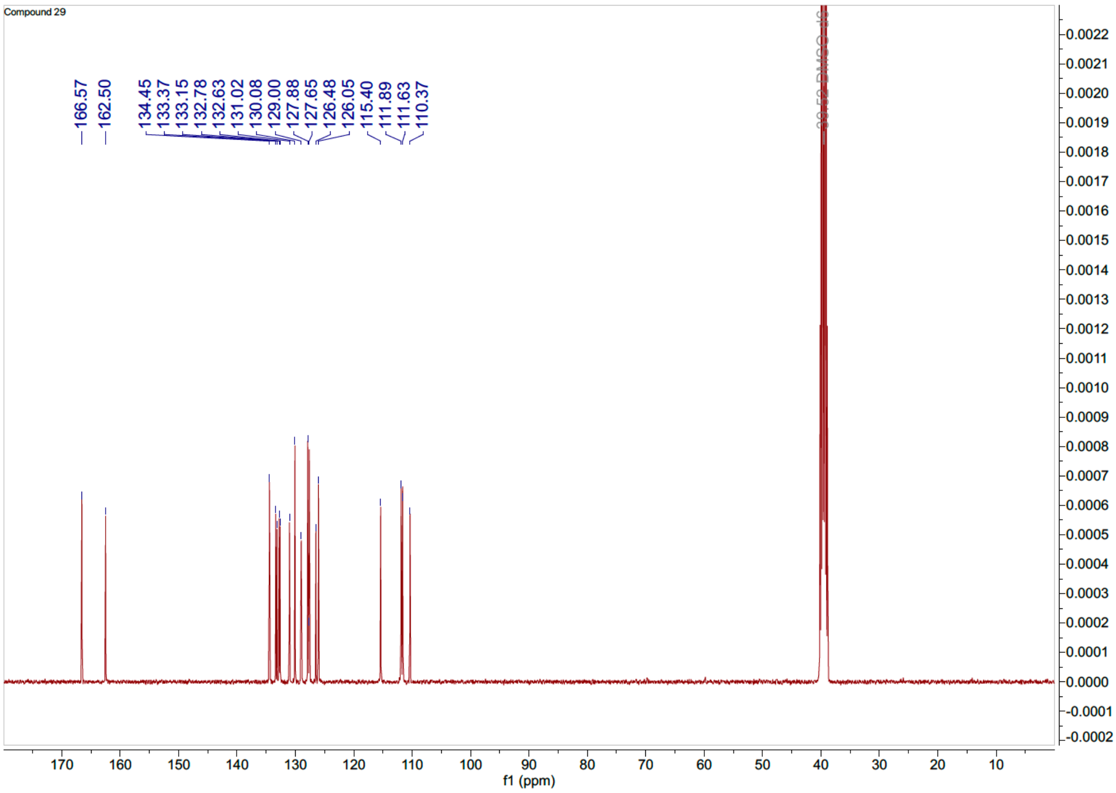

(*E*)-5-(3-(2,6-dichlorophenyl)acrylamido)-1*H*-indole-3-carboxamide (30)

^{1}H NMR (400 MHz, DMSO)

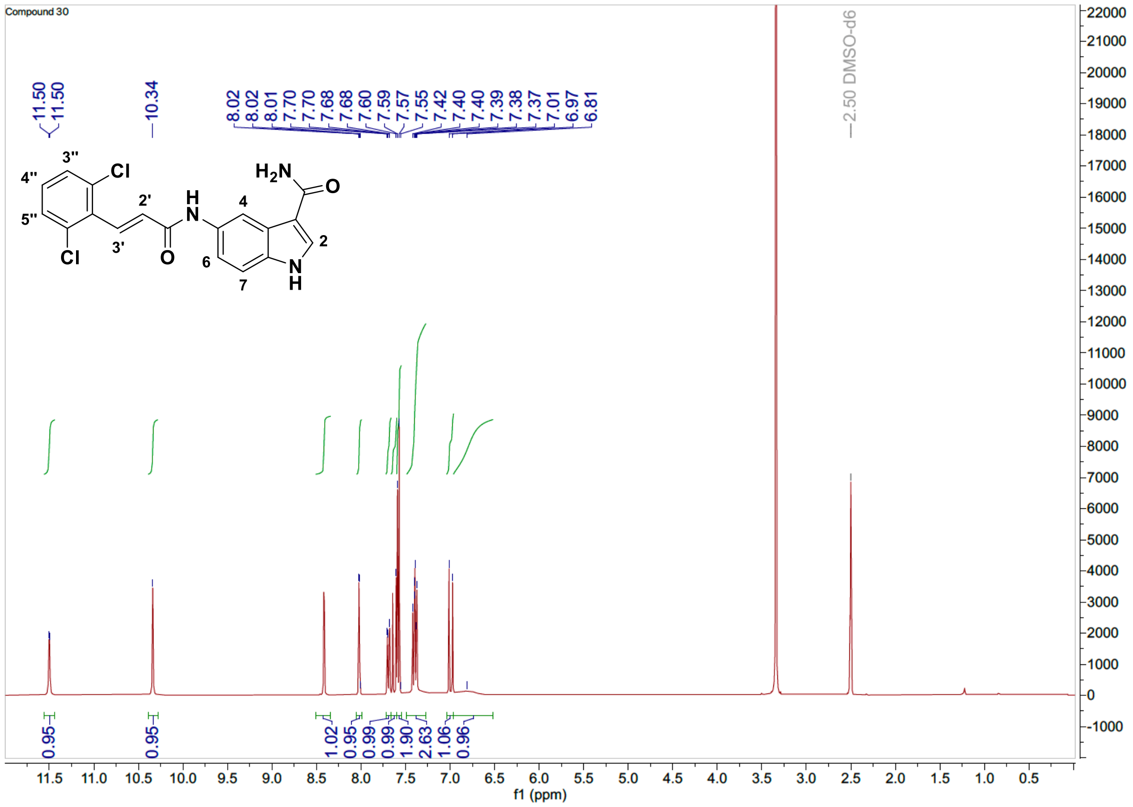

^{13}C NMR (100 MHz, DMSO)

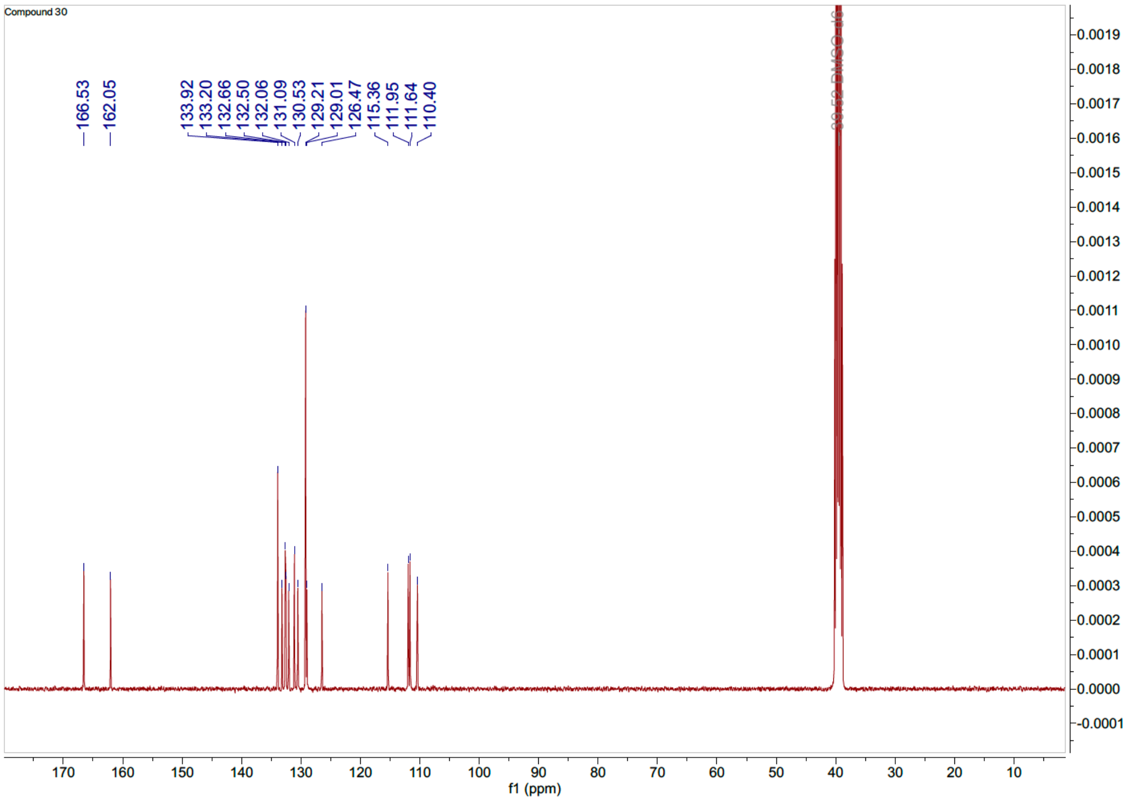

(*E*)-5-(3-(2-fluorophenyl)acrylamido)-1*H*-indole-3-carboxamide (31)

^{1}H NMR (400 MHz, DMSO)

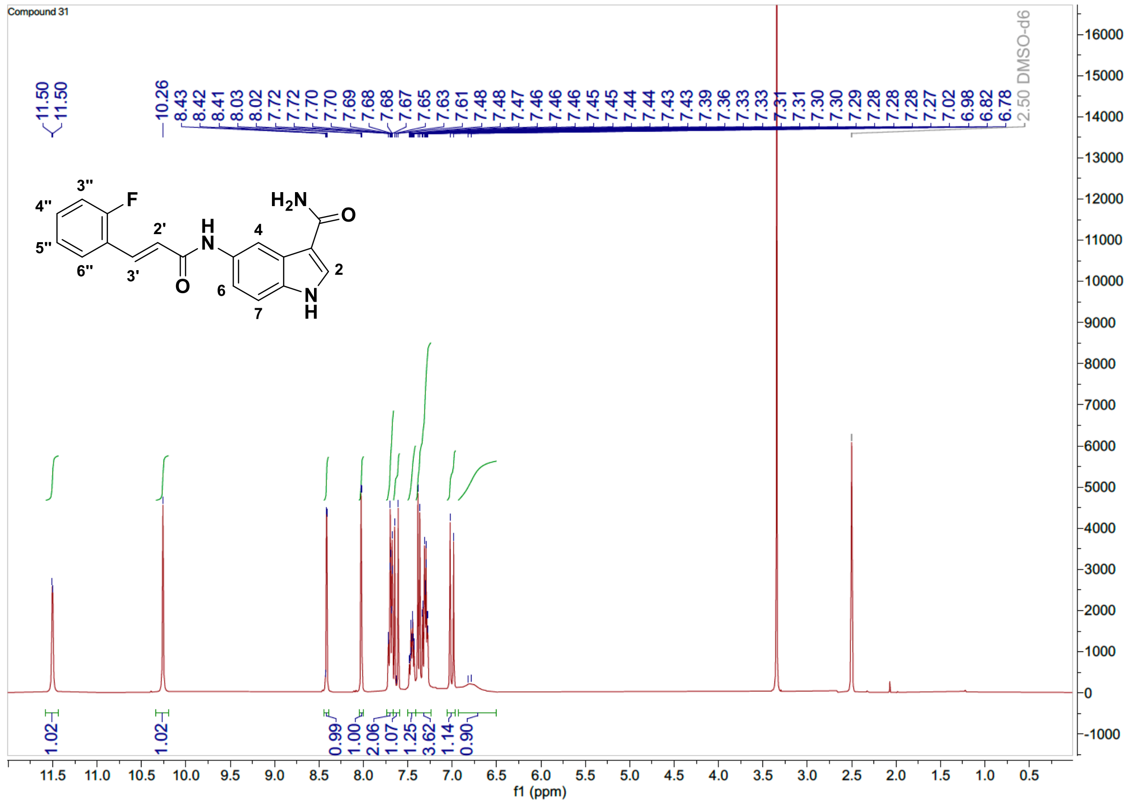

^{13}C NMR (100 MHz, DMSO)

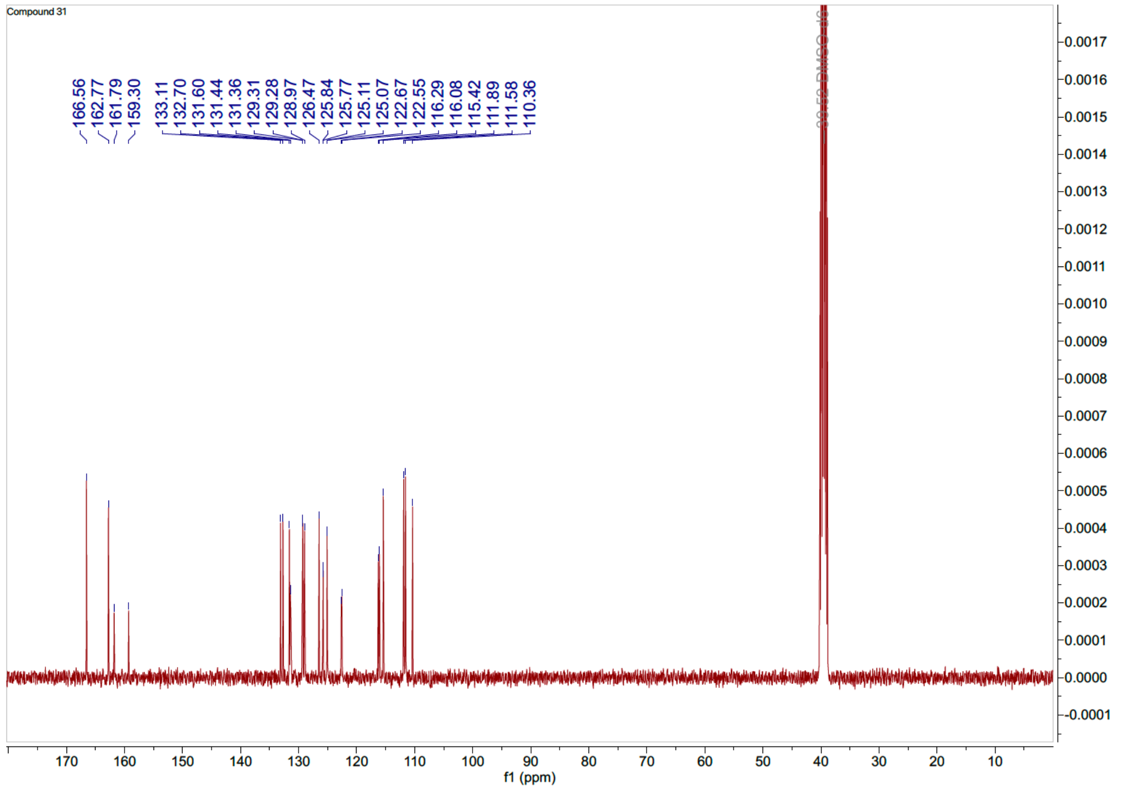

(*E*)-5-(3-(3-fluorophenyl)acrylamido)-1*H*-indole-3-carboxamide (32)

^{1}H NMR (400 MHz, DMSO)

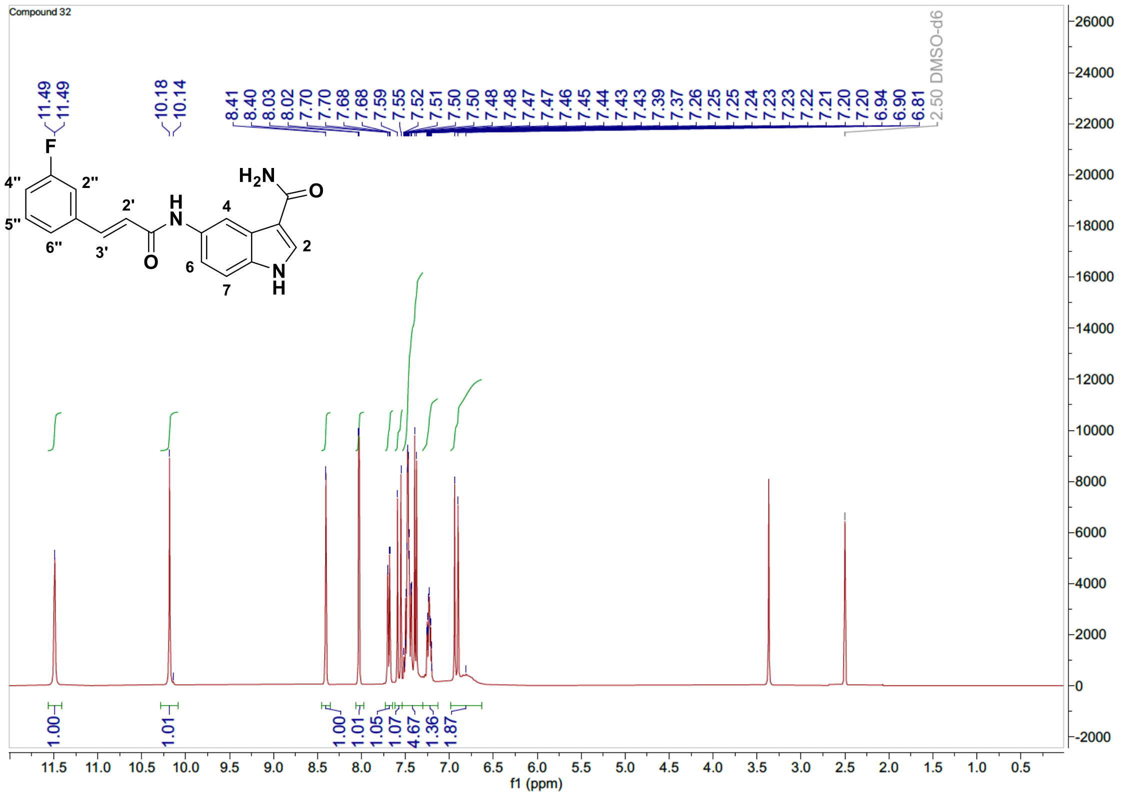

^{13}C NMR (100 MHz, DMSO)

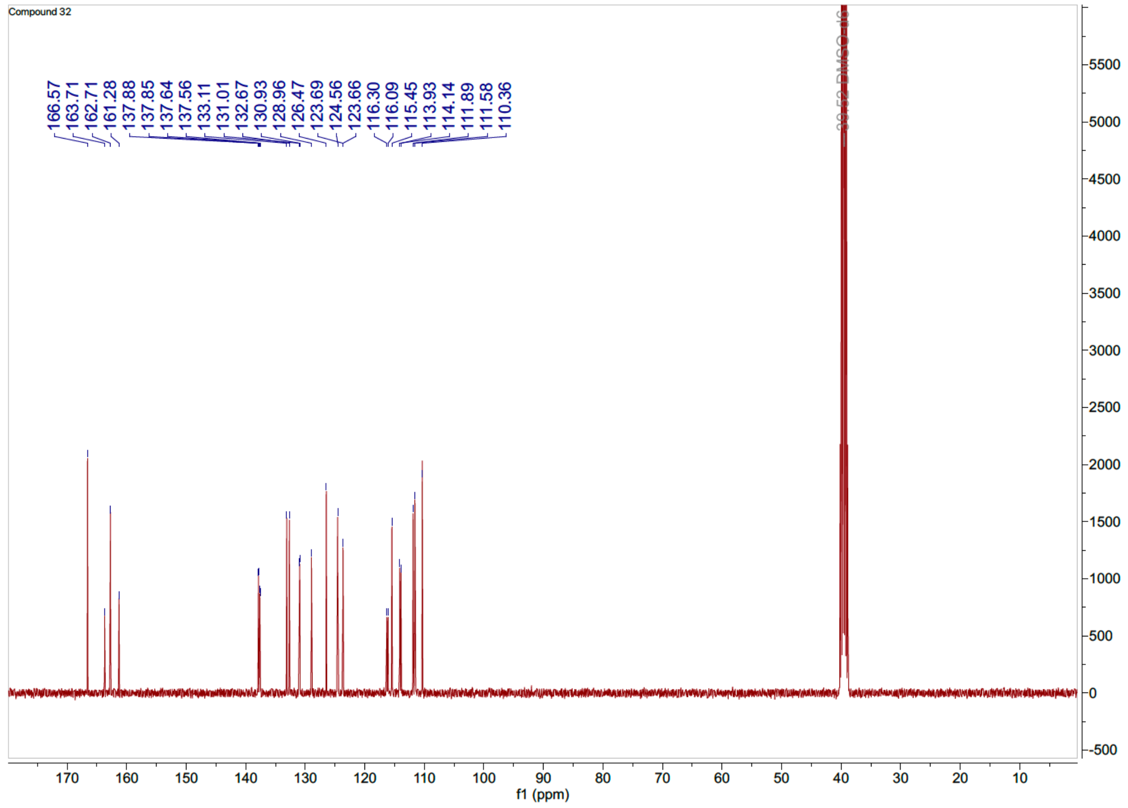

(*E*)-5-(3-(4-fluorophenyl)acrylamido)-1*H*-indole-3-carboxamide (33)

^{1}H NMR (400 MHz, DMSO)

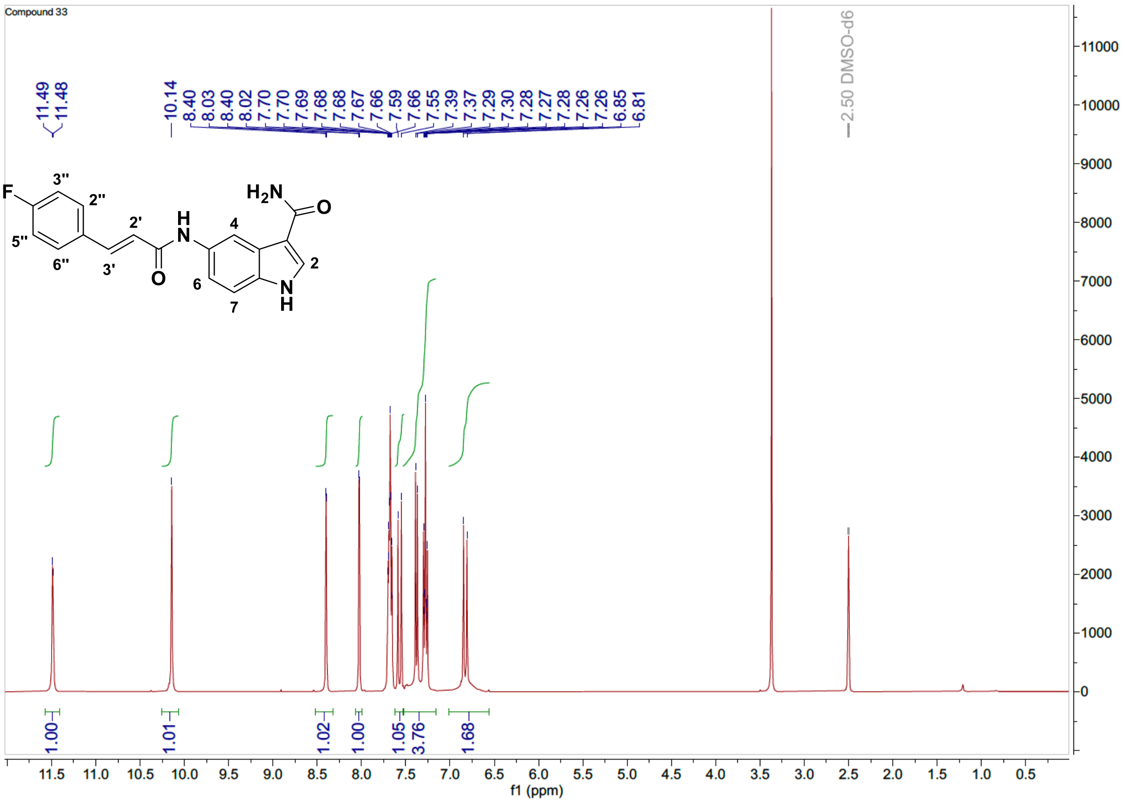

^{13}C NMR (100 MHz, DMSO)

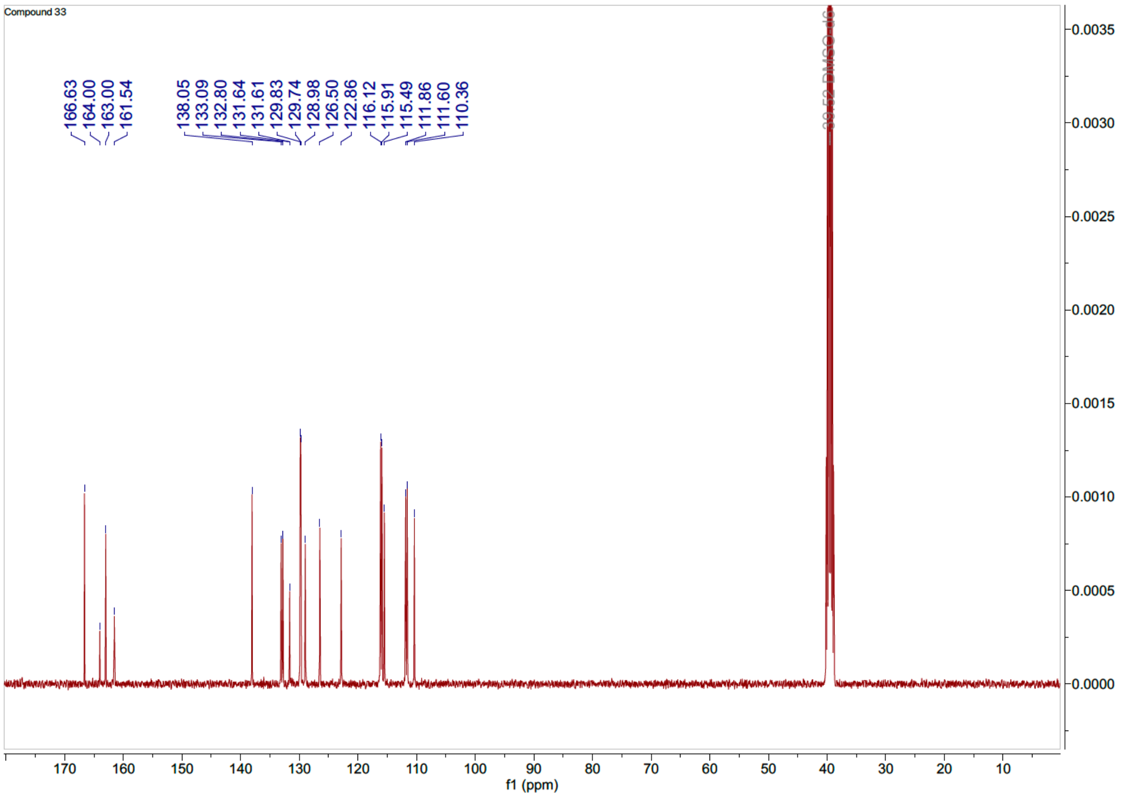

(*E*)-5-(3-(2,3-difluorophenyl)acrylamido)-1*H*-indole-3-carboxamide (34)

^{1}H NMR (400 MHz, DMSO)

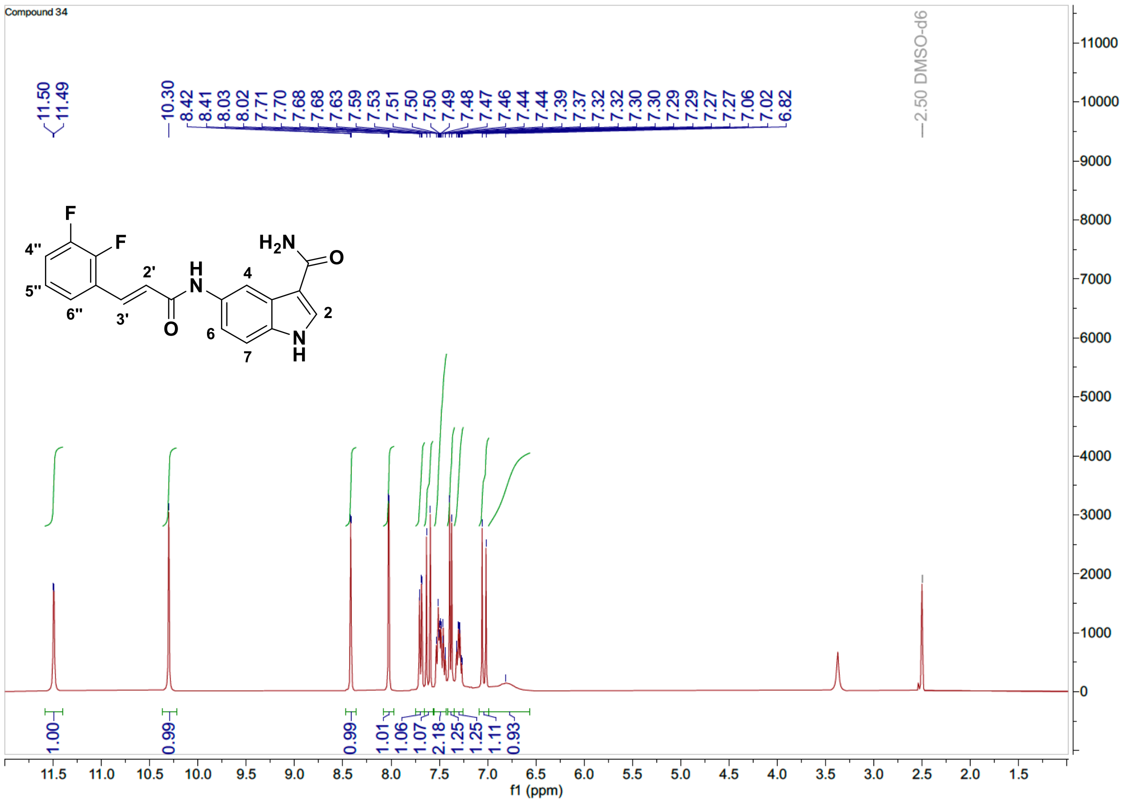

^{13}C NMR (125 MHz, DMSO)

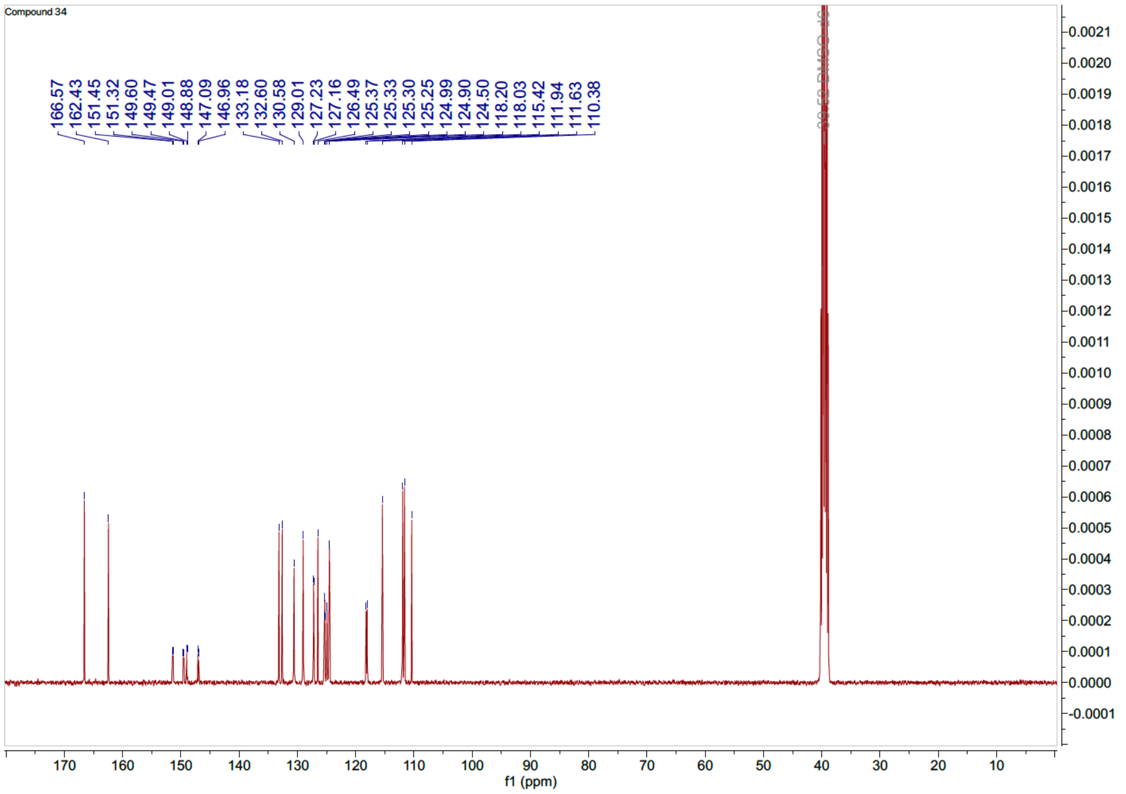

(*E*)-5-(3-(2,4-difluorophenyl)acrylamido)-1*H*-indole-3-carboxamide (35)

^{1}H NMR (400 MHz, DMSO)

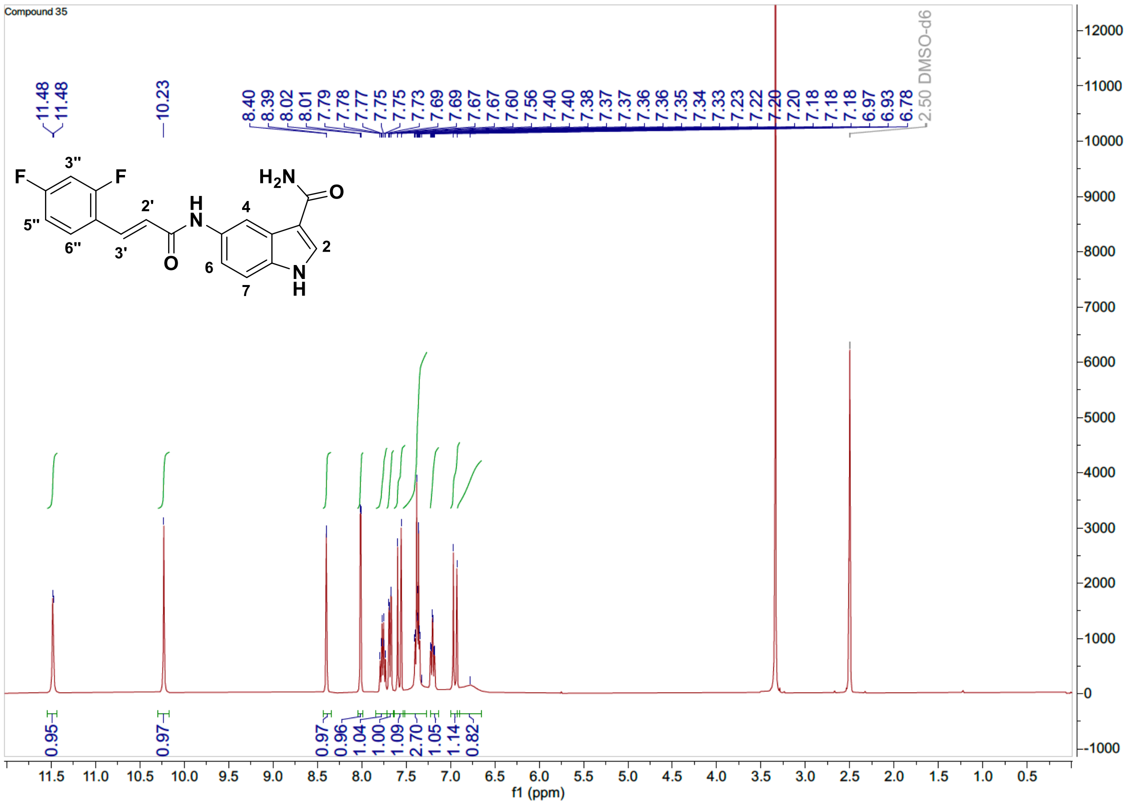

^{13}C NMR (100 MHz, DMSO)

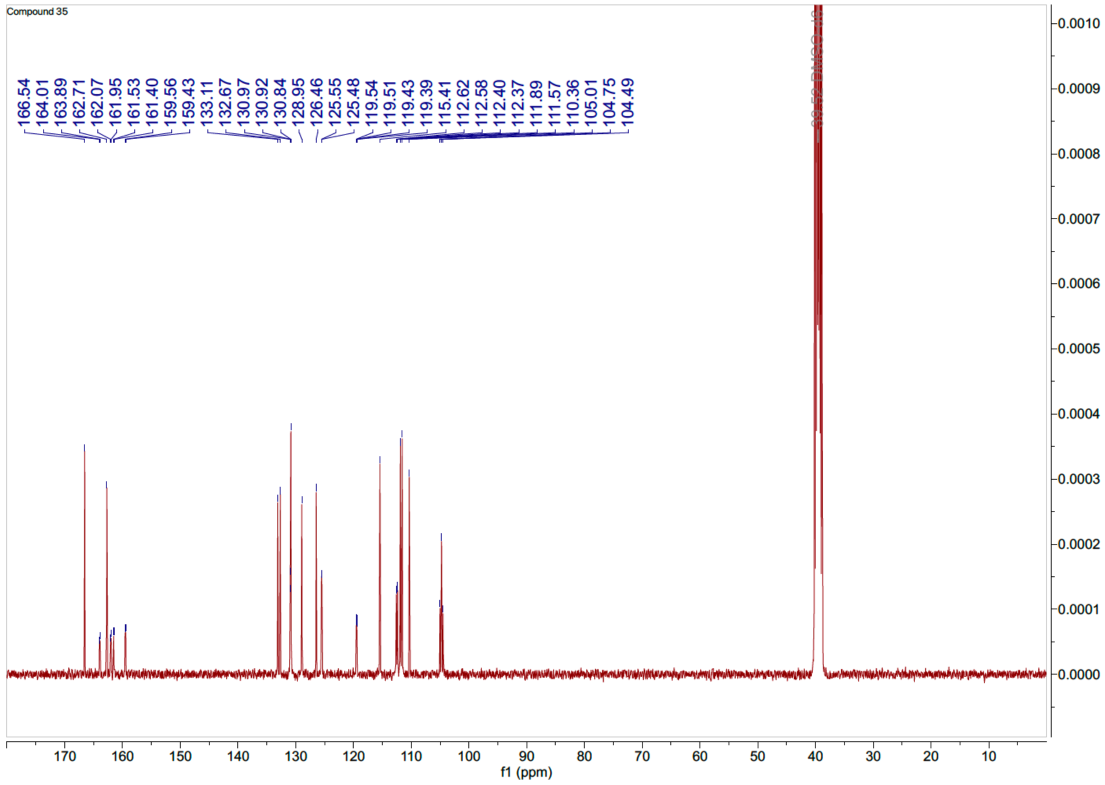

(*E*)-5-(3-(2,5-difluorophenyl)acrylamido)-1*H*-indole-3-carboxamide (36)

^{1}H NMR (400 MHz, DMSO)

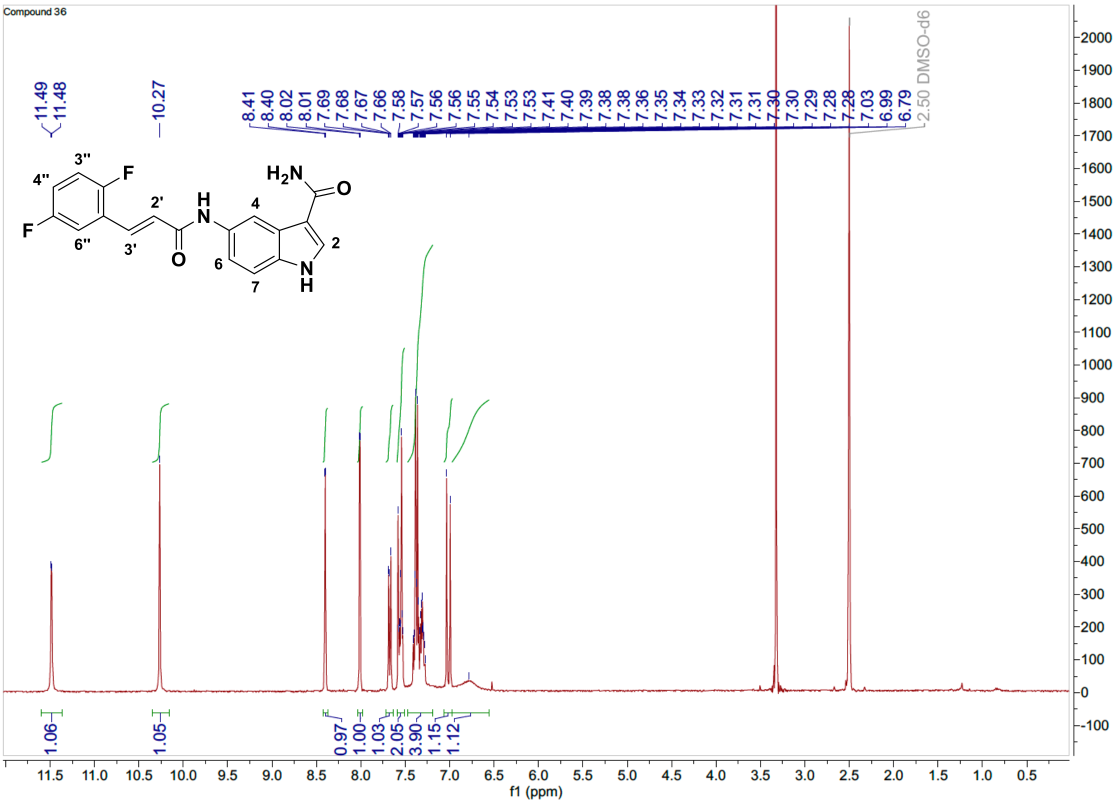

^{13}C NMR (100 MHz, DMSO)

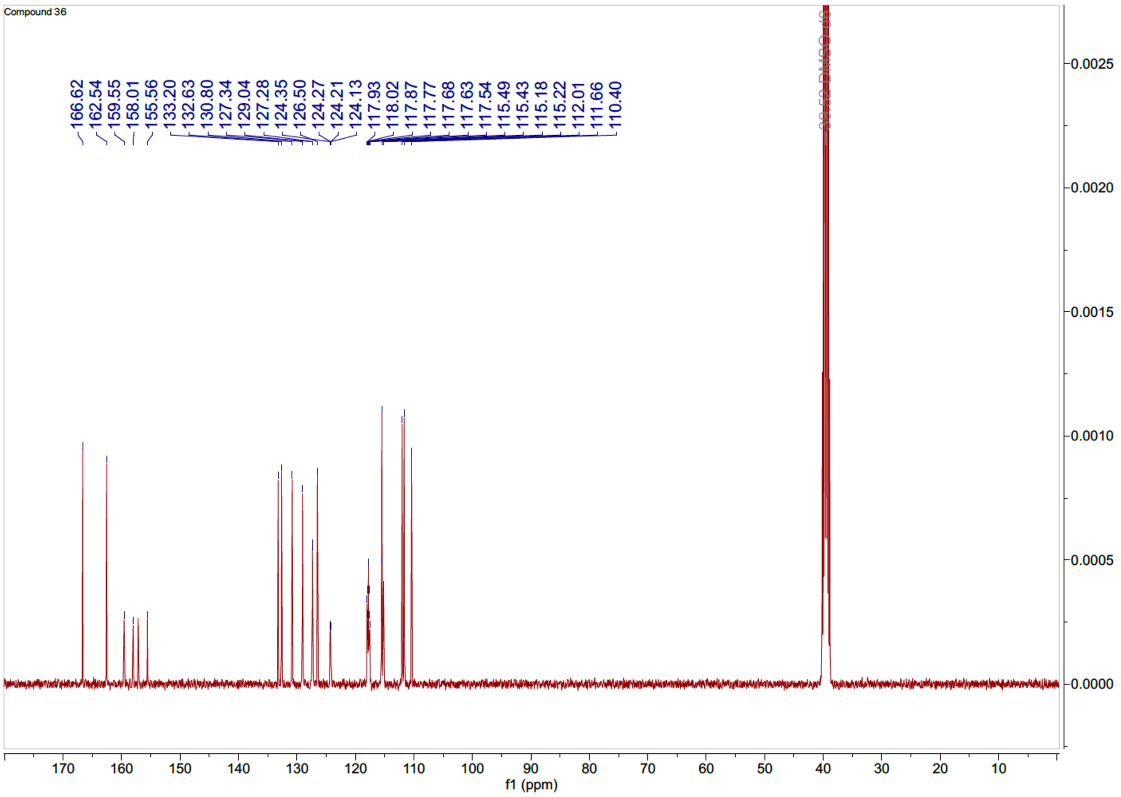

(*E*)-5-(3-(2,6-difluorophenyl)acrylamido)-1*H*-indole-3-carboxamide (37)

^{1}H NMR (400 MHz, DMSO)

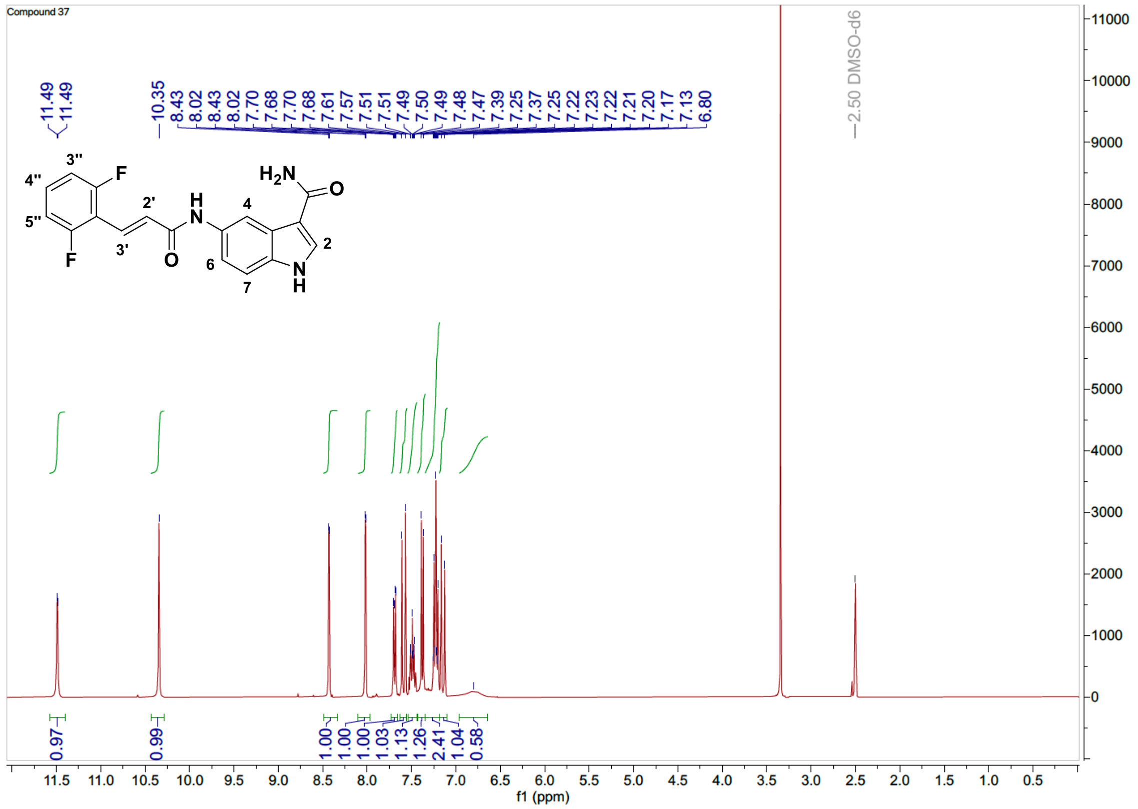

^{13}C NMR (100 MHz, DMSO)

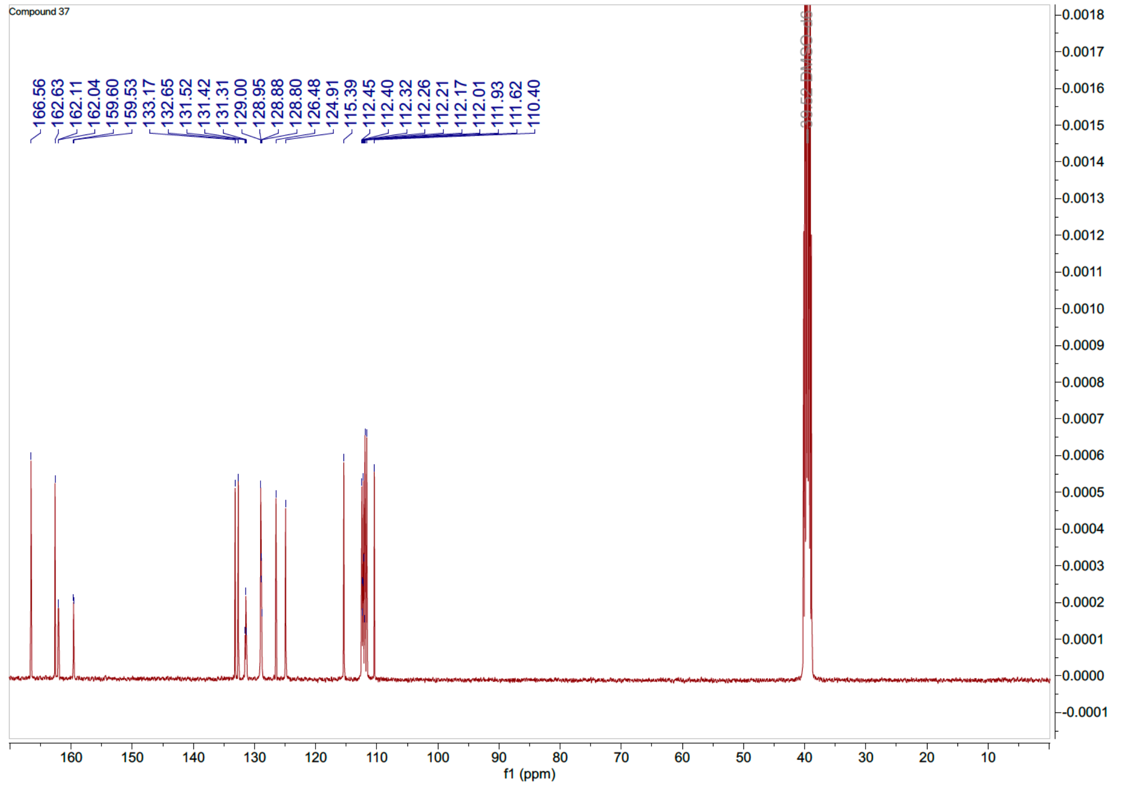

(*E*)-5-(3-(3,4-difluorophenyl)acrylamido)-1*H*-indole-3-carboxamide (38)

^{1}H NMR (400 MHz, DMSO)

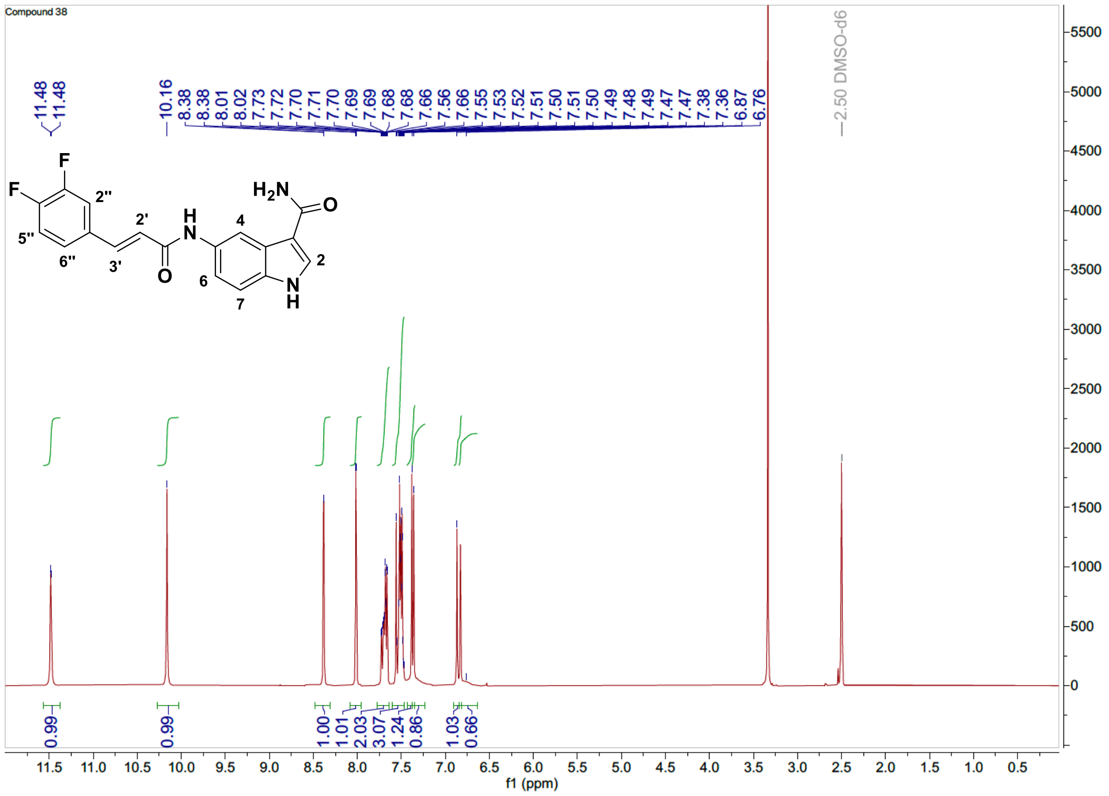

^{13}C NMR (100 MHz, DMSO)

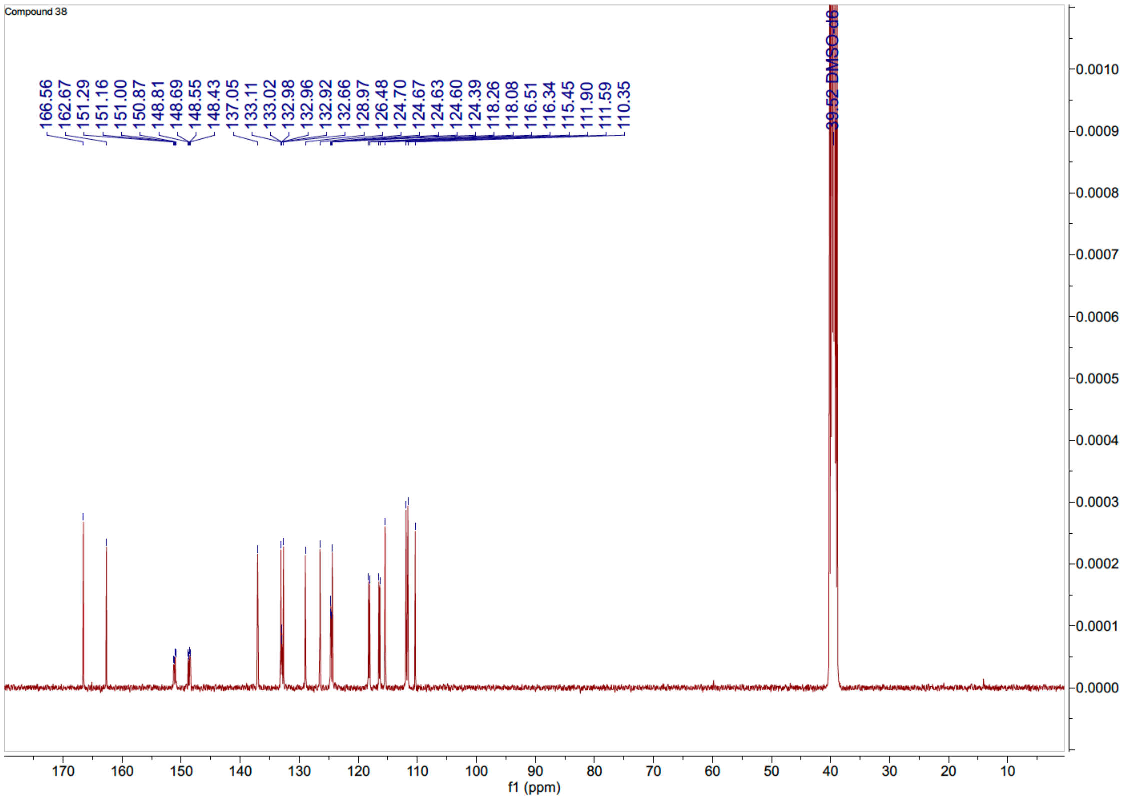

(*E*)-5-(3-(3,5-difluorophenyl)acrylamido)-1*H*-indole-3-carboxamide (39)

^{1}H NMR (400 MHz, DMSO)

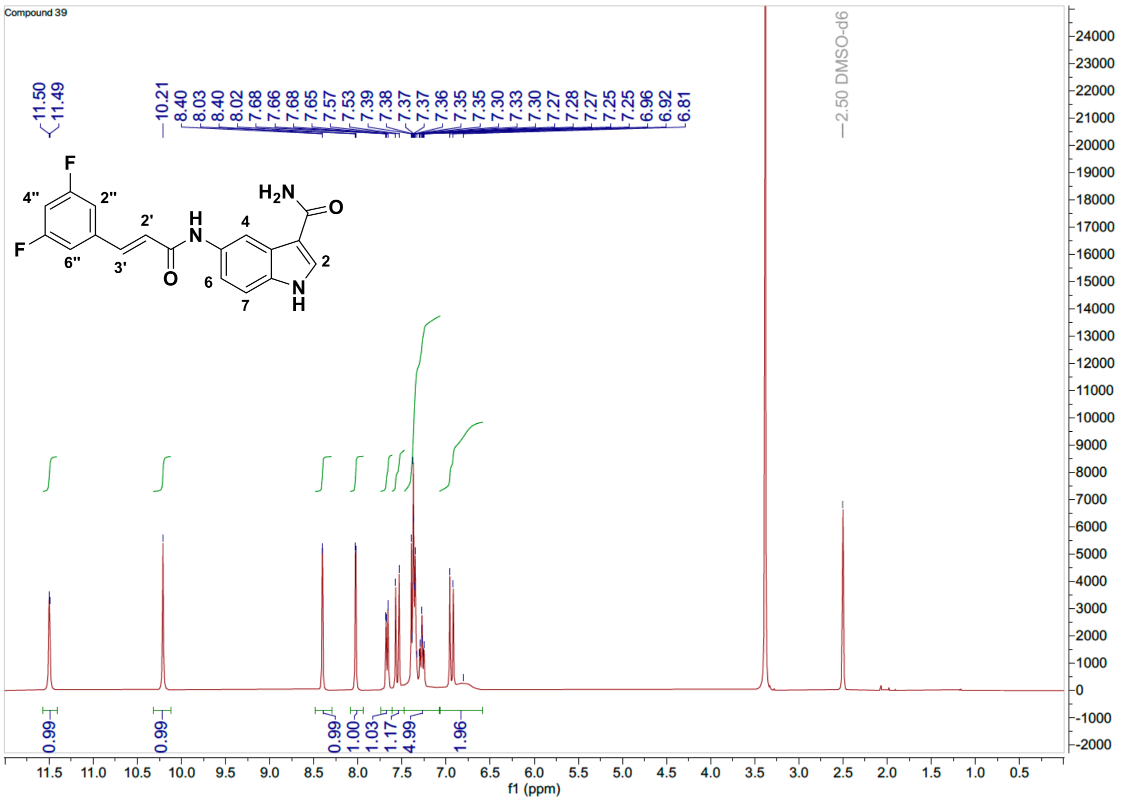

^{13}C NMR (100 MHz, DMSO)

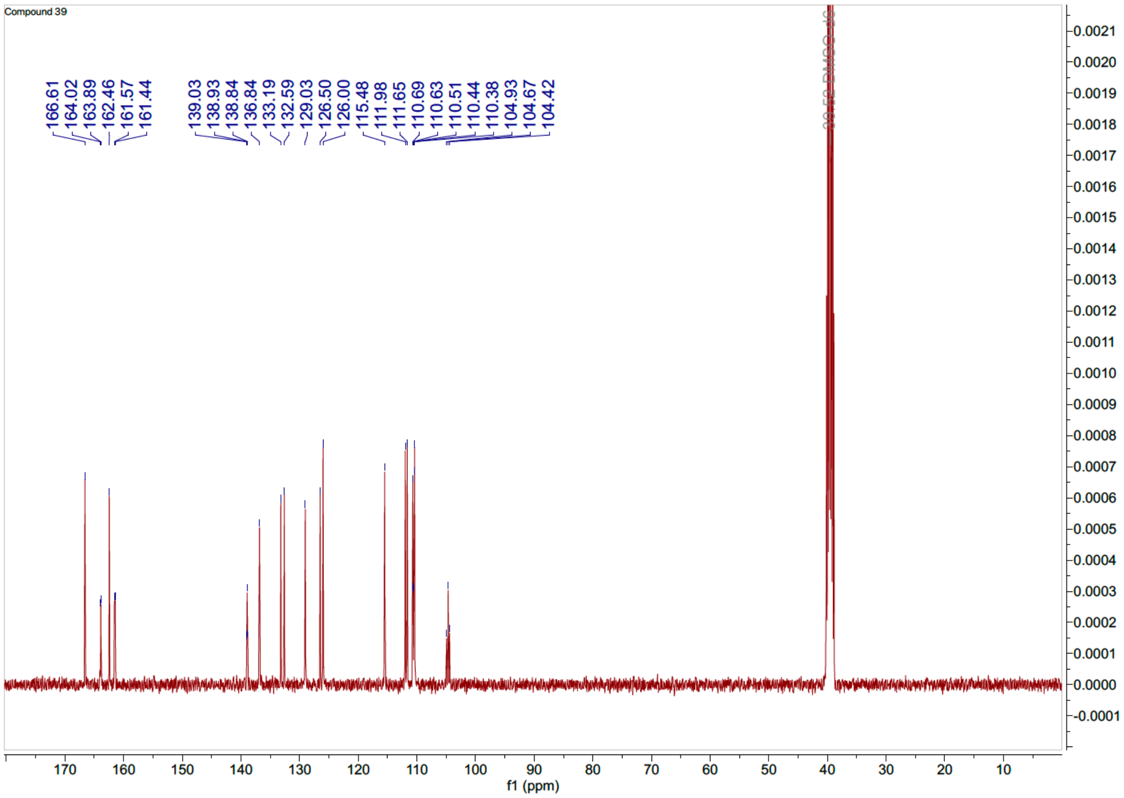

(*E*)-5-(3-(2-(trifluoromethyl)phenyl)acrylamido)-1*H*-indole-3-carboxamide (40)

^{1}H NMR (400 MHz, DMSO)

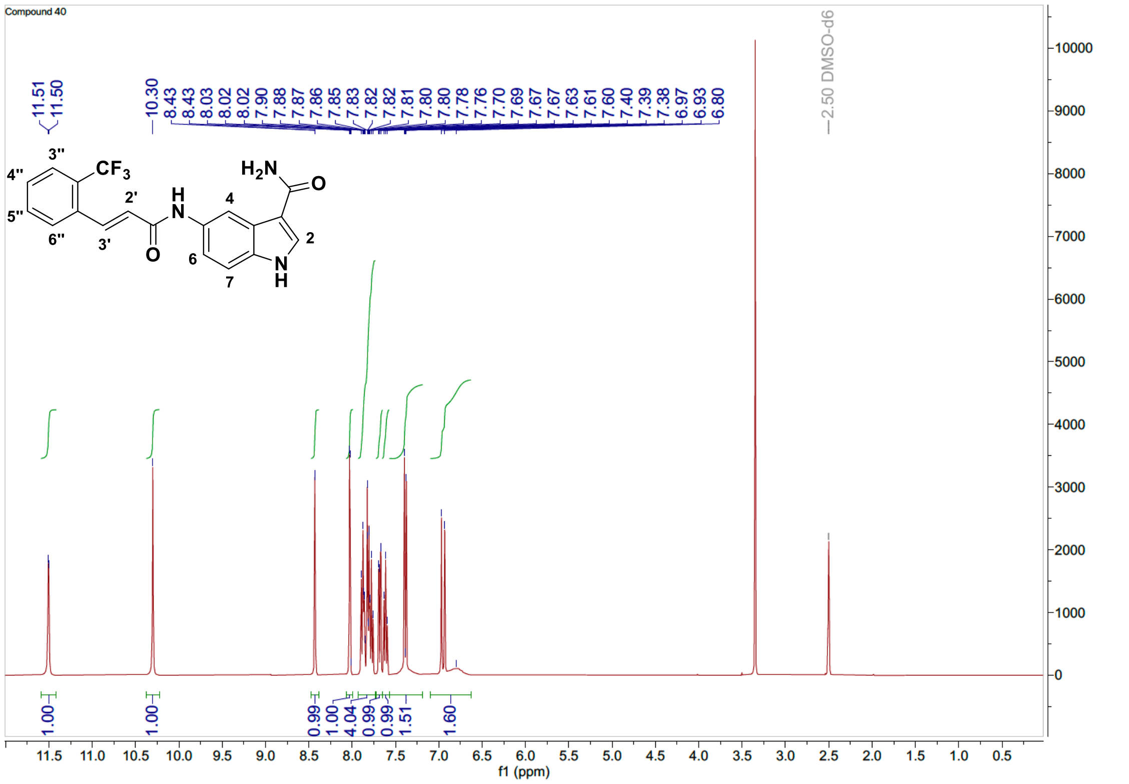

^{13}C NMR (100 MHz, DMSO)

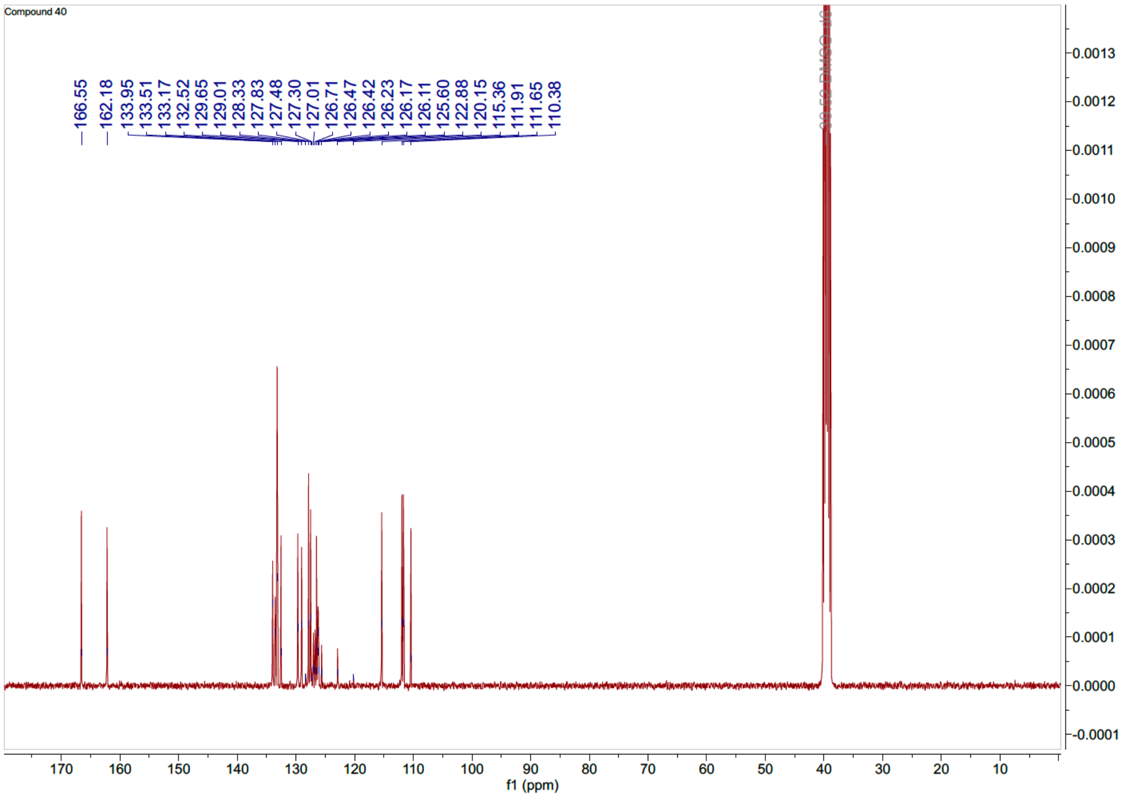

Copies of spectra (HPLC traces and exact mass)

(*E*)-*N*-(1*H*-indol-5-yl)-3-(*o*-tolyl)acrylamide (7)

Equipo MAXIS II

Nombre muestra	LC1330
Nombre registro	D:\Data\2021\HPLC\02_FEBRERO\LC1330_31_01_2847 .d
Metodo	ESI Positive hplc1100_ 40 min_50-3000 t=200 6l-min.m
Comentarios	PD43 (1mg/ml en metanol. Dilucion 1:50 en metanol)

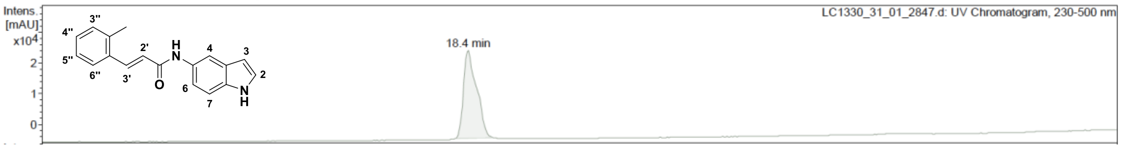

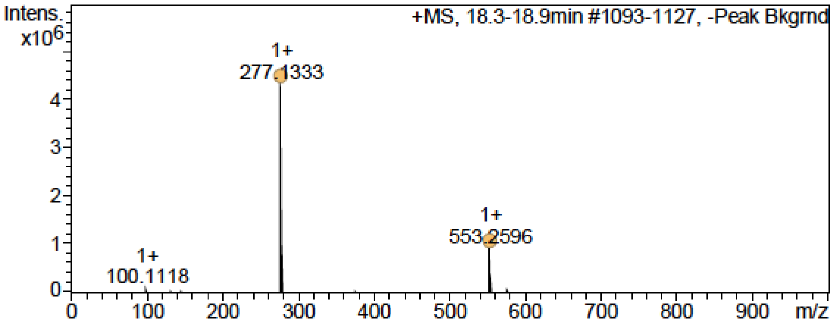

(*E*)-*N*-(1*H*-indol-5-yl)-3-(*m*-tolyl)acrylamide (8)

Equipo MAXIS II

Nombre muestra	LC1338
Nombre registro	D:\Data\2021\HPLC\02_FEBRERO\LC1338_71_01_2855 .d
Metodo	ESI Positive hplc1100_ 40 min_50-3000 t=200 6l-min.m
Comentarios	PD69 (1mg/ml en metanol. Dilucion 1:50 en metanol)

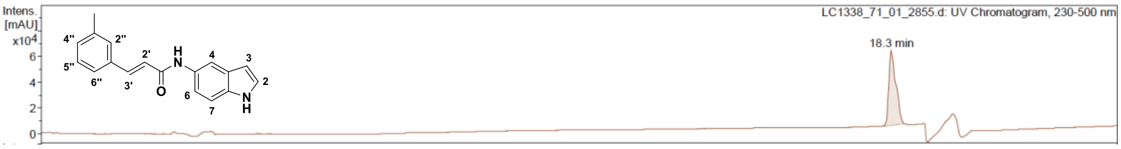

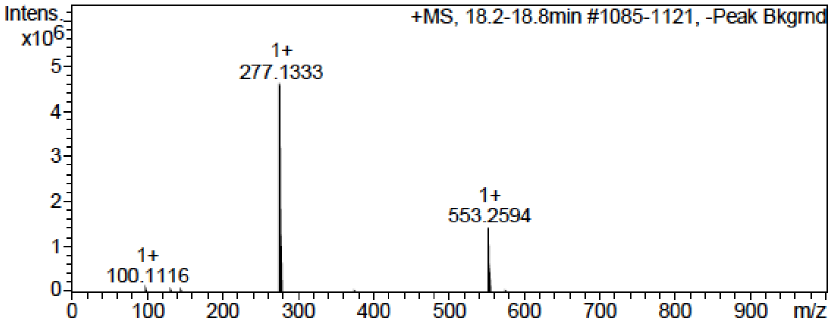

(*E*)-3-(2-chlorophenyl)-*N*-(1*H*-indol-5-yl)acrylamide (9)

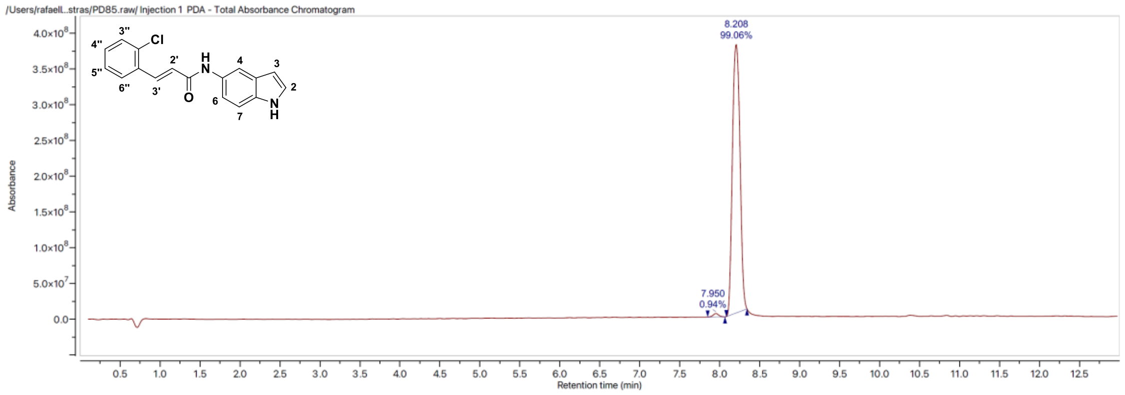

Compound Spectra (overlaid)

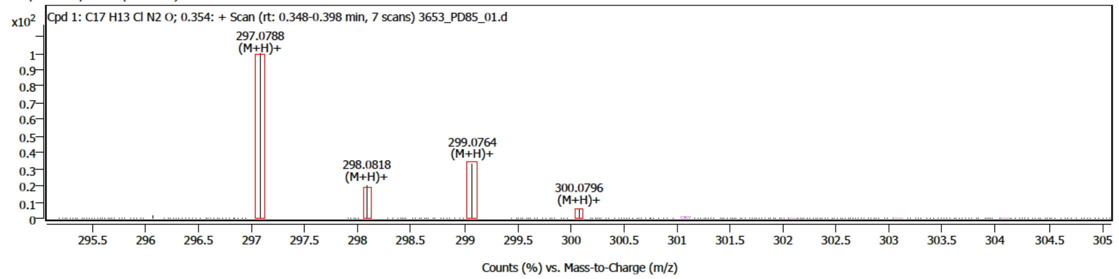

Compound Details

Cpd. 1: C17 H13 Cl N2 O

Compound ID Table

Name	Formula	Species	RT	RT Diff	Mass	Mass (Tgt)	ID Source	Score	Diff (ppm)	Score (MFG)
	C17 H13 Cl N2 O	(M+Na)+	1.179		296.0715	296.0716	FBF	99.76	-0.39	

Compound Spectra (overlaid)

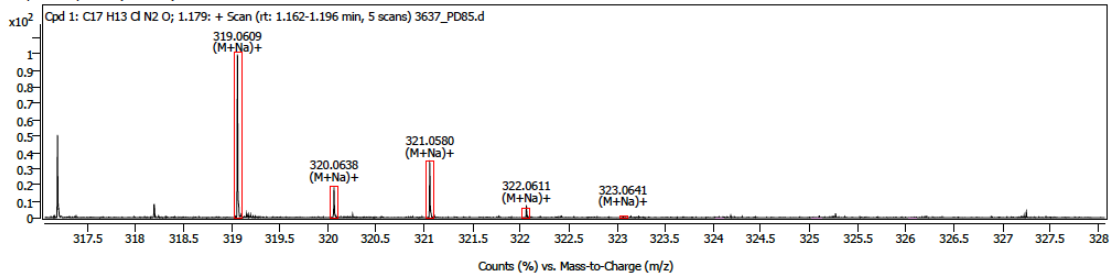

(*E*)-3-(2,6-dichlorophenyl)-*N*-(1*H*-indol-5-yl)acrylamide (10)

Equipo MAXIS II

Nombre muestra	LC1205
Nombre registro	\\150.244.121.174\Data\2020\HPLC\11_NOVIEMBRE\LC1205_41_01_2722.d
Metodo	ESI Positive hplc1100_ 40 min_50-3000 t=200 6l-min.m
Comentarios	PD59 en 0.5 ul MeOH (Dil 1:40 con MeOH)

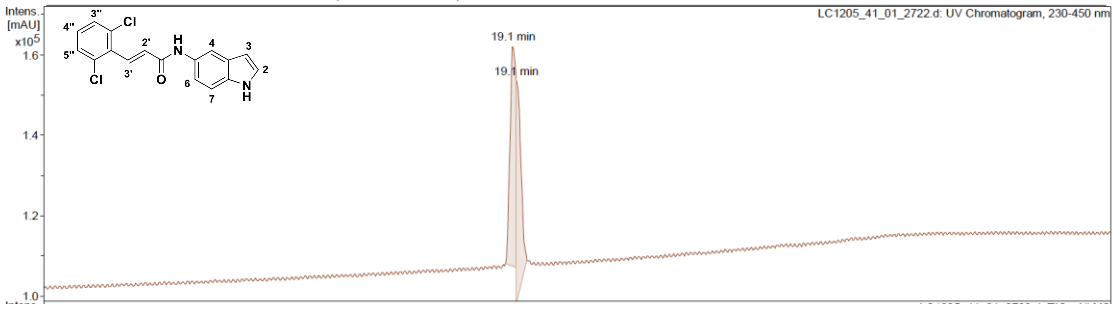

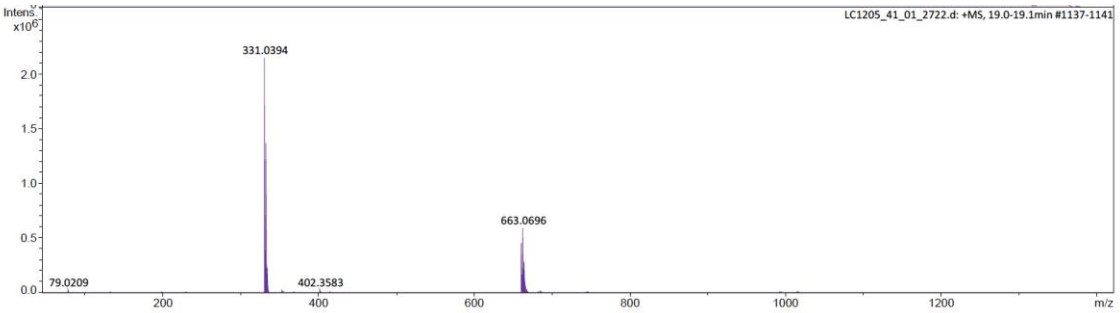

(*E*)-3-(2-fluorophenyl)-*N*-(1*H*-indol-5-yl)acrylamide (11)

Equipo MAXIS II

Nombre muestra	LC1258
Nombre registro	D:\Data\2020\HPLC\12_DICIEMBRE\LC1258_41_01_27<br>75.d
Metodo	ESI Positive hplc1100_ 40 min_50-3000 t=200 6l-min.m
Comentarios	PD46 (0.5 mg/ml en MeOH. Dil 1:20 con MeOH)

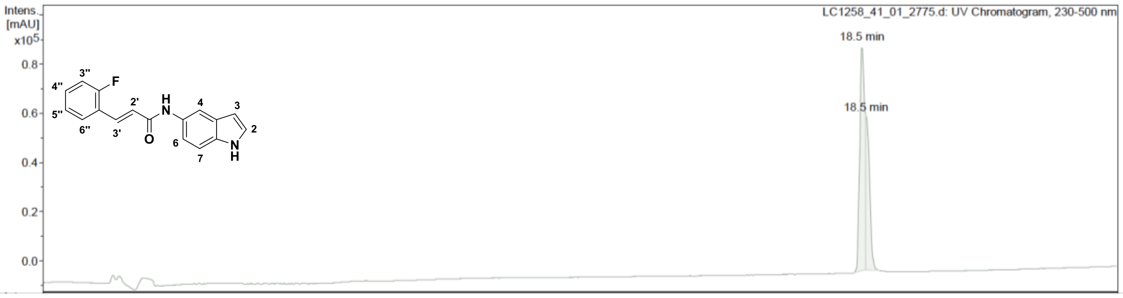

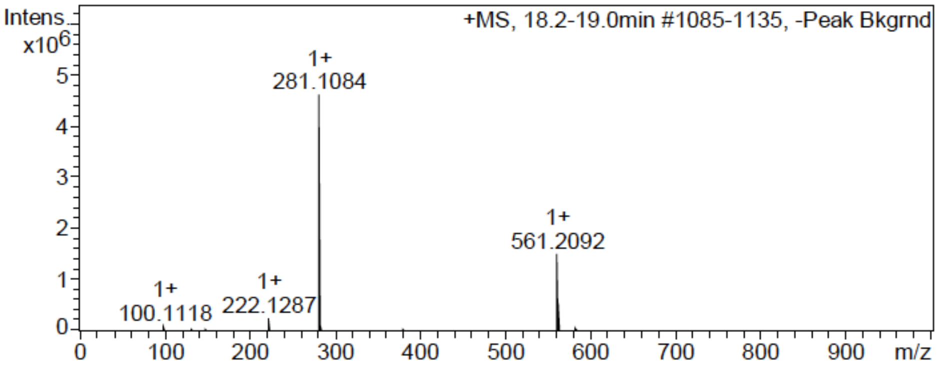

(*E*)-3-(3-fluorophenyl)-*N*-(1*H*-indol-5-yl)acrylamide (12)

Equipo MAXIS II

Nombre muestra	LC1256
Nombre registro	D:\Data\2020\HPLC\12_DICIEMBRE\LC1256_31_01_27 73.d
Metodo	ESI Positive hplc1100_ 40 min_50-3000 t=200 6l-min.m
Comentarios	PD60 (0.5 mg/ml en MeOH; Dil 1:20 con MeOH)

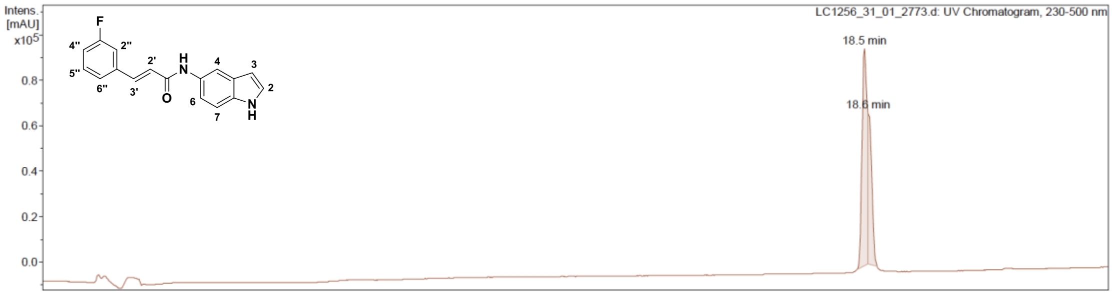

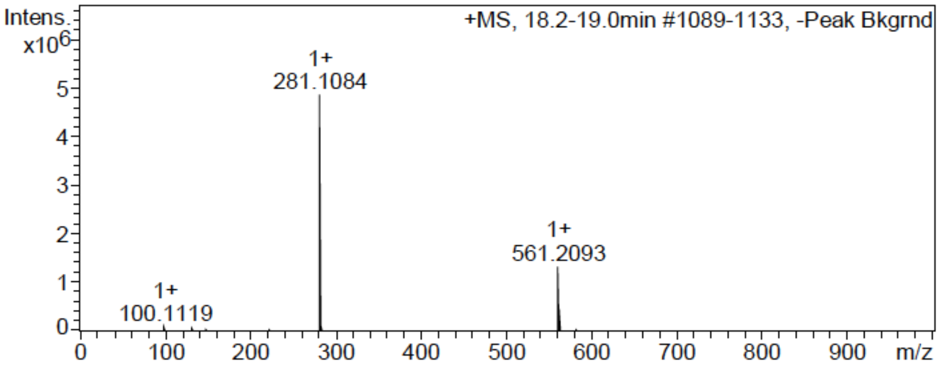

(*E*)-3-(4-fluorophenyl)-*N*-(1*H*-indol-5-yl)acrylamide (13)

Equipo MAXIS II

Nombre muestra	LC1203
Nombre registro	\\150.244.121.174\Data\2020\HPLC\11_NOVIEMBRE\LC1203_31_01_2720.d
Metodo	ESI Positive hplc1100_ 40 min_50-3000 t=200 6l-min.m
Comentarios	PD61 en 0.5 ul MeOH (Dil 1:40 con MeOH)

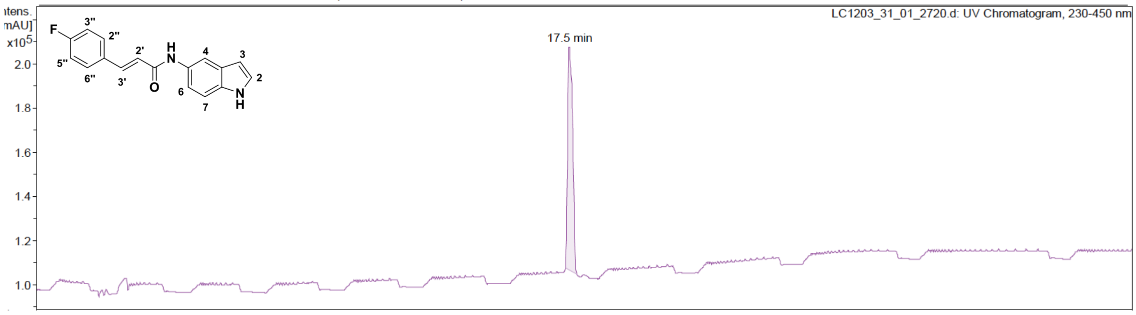

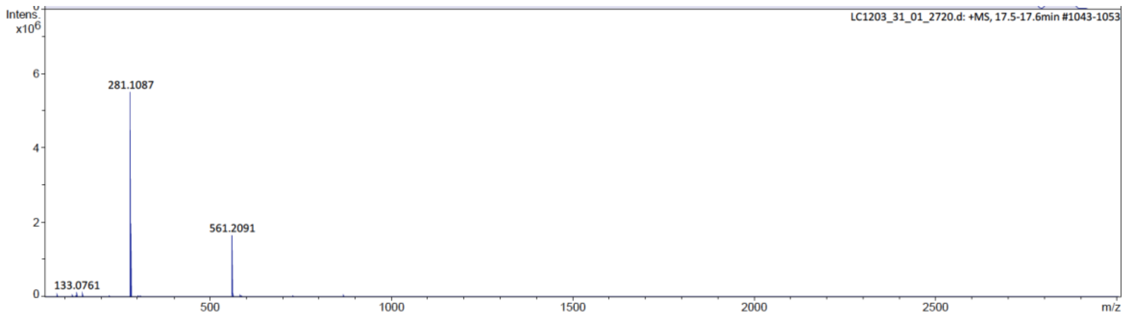

(*E*)-3-(2,3-difluorophenyl)-*N*-(1*H*-indol-5-yl)acrylamide (14)

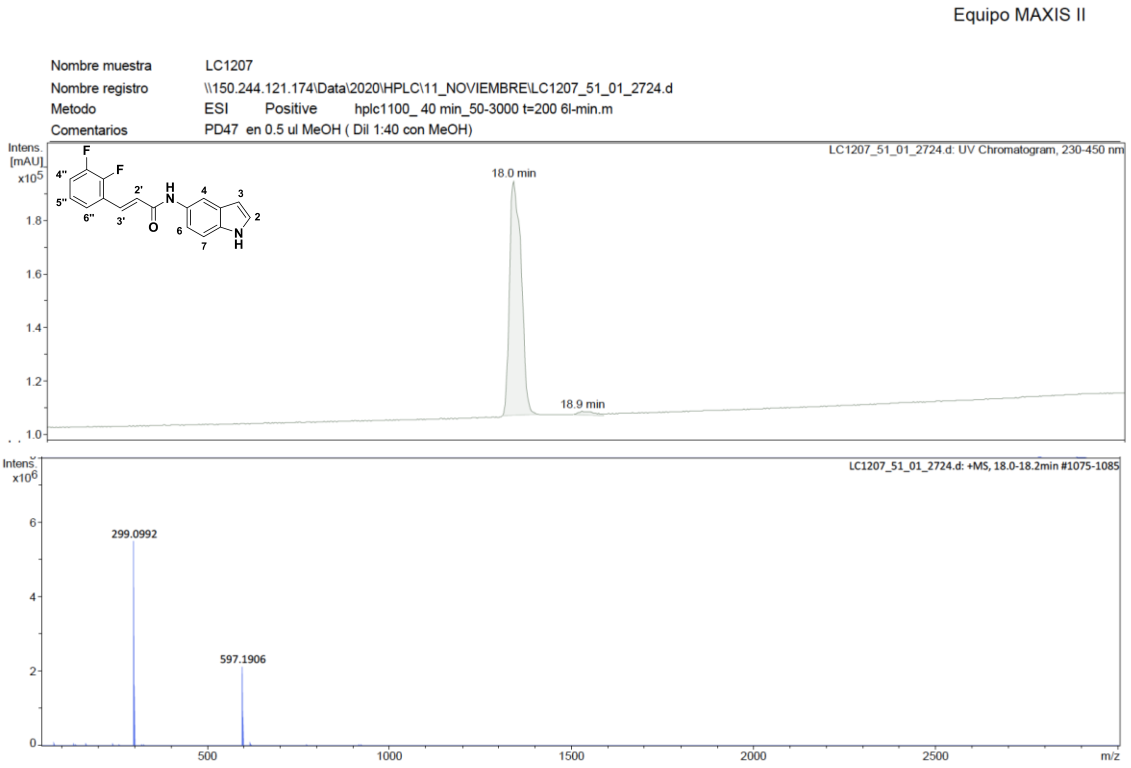

(*E*)-3-(2,4-difluorophenyl)-*N*-(1*H*-indol-5-yl)acrylamide (15)

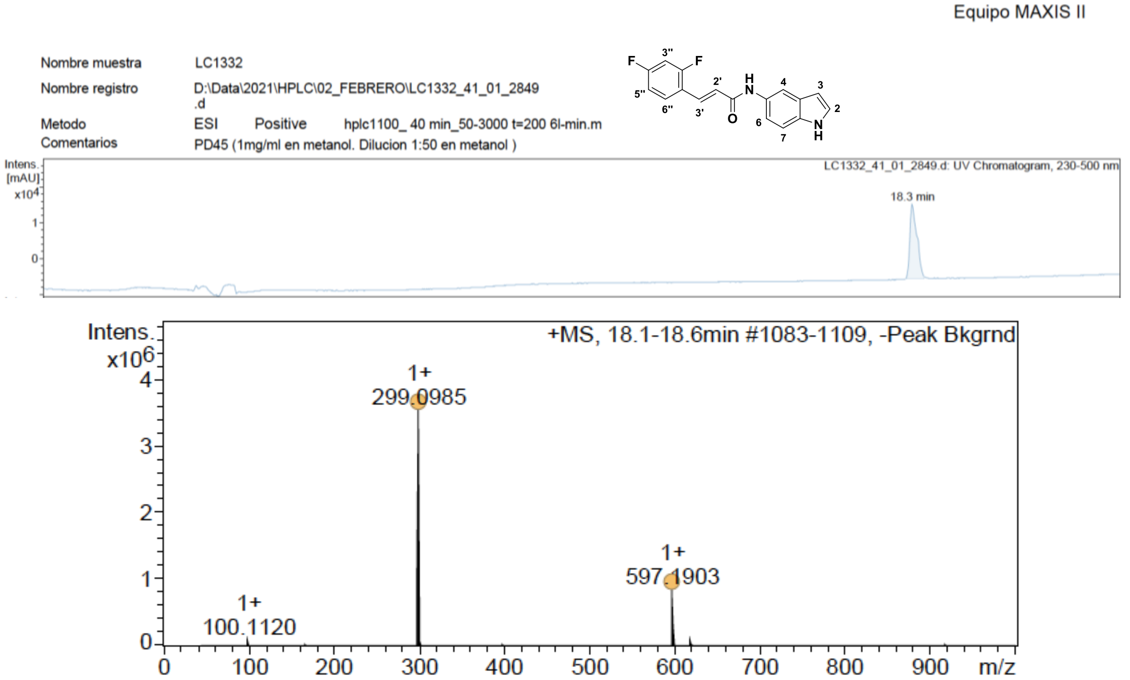

(*E*)-3-(2,5-difluorophenyl)-*N*-(1*H*-indol-5-yl)acrylamide (16)

Equipo MAXIS II

Nombre muestra	LC1260	
Nombre registro	D:\Data\2020\HPLC\12_DICIEMBRE\LC1260_51_01_2777.d	
Metodo	ESI Positive	hplc1100_ 40 min_50-3000 t=200 6l-min.m
Comentarios	PD44 (0.5 mg/ml en MeOH. Dil 1:20 con MeOH)	

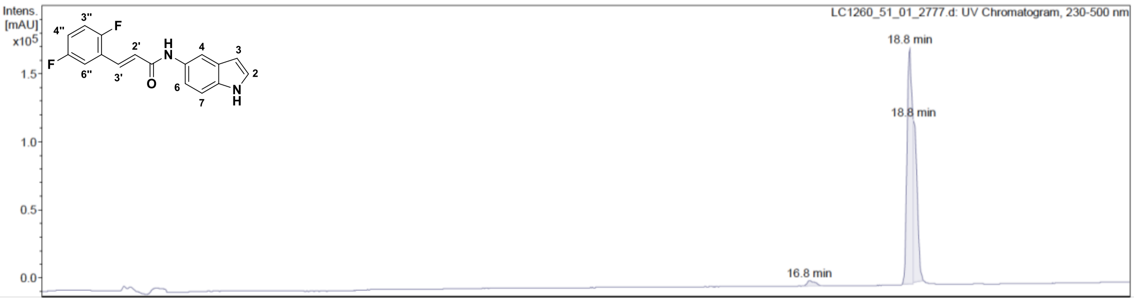

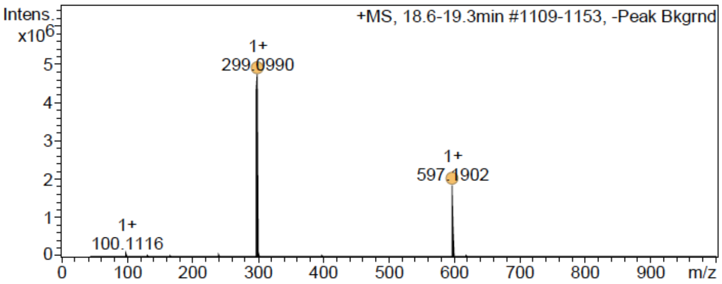

(*E*)-3-(2,6-difluorophenyl)-*N*-(1*H*-indol-5-yl)acrylamide (17)

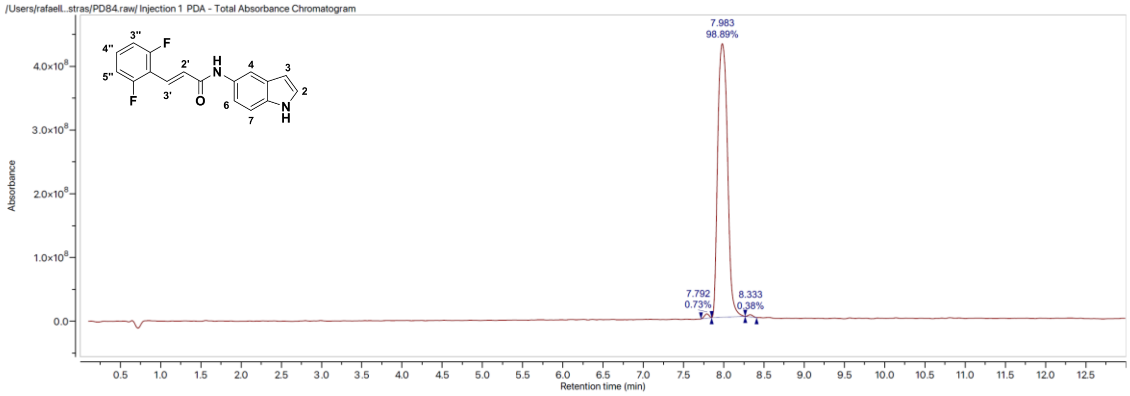

Compound Details

Cpd. 1: C17 H12 F2 N2 O

Compound ID Table

Name	Formula	Species	RT	RT Diff	Mass	Mass (Tgt)	ID Source	Score	Diff (ppm)	Score (MFG)
	C17 H12 F2 N2 O	(M+H)+	0.466		298.0917	298.0918	FBF	99.64	-0.30	

Compound Spectra (overlaid)

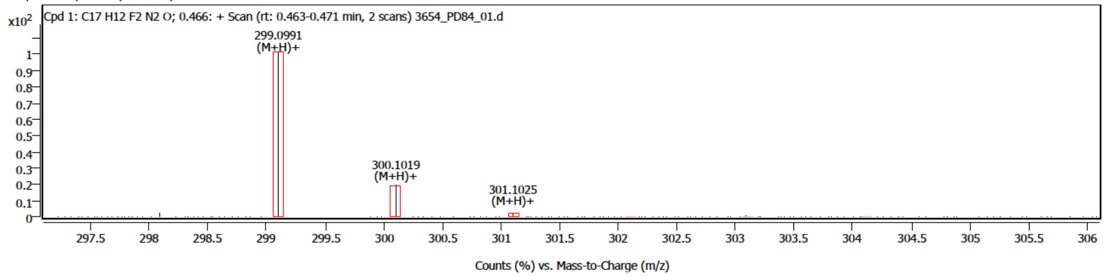

Compound Details

Cpd. 1: C17 H12 F2 N2 O

Compound ID Table

Name	Formula	Species	RT	RT Diff	Mass	Mass (Tgt)	ID Source	Score	Diff (ppm)	Score (MFG)
	C17 H12 F2 N2 O	(M+Na)+	0.234		298.0918	298.0918	FBF	99.93	0.04	

Compound Spectra (overlaid)

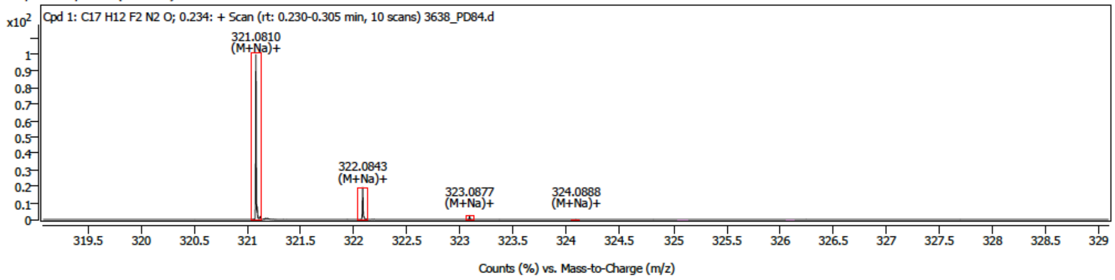

(*E*)-3-(3,4-difluorophenyl)-*N*-(1*H*-indol-5-yl)acrylamide (18)

Equipo MAXIS II

Nombre muestra	LC1254
Nombre registro	D:\Data\2020\HPLC\12_DICIEMBRE\LC1254_21_01_2771.d
Metodo	ESI Positive hplc1100_ 40 min_50-3000 t=200 6l-min.m
Comentarios	PD62 (0.5mg/ml en MeOH. Dil 1:20 con MeOH)

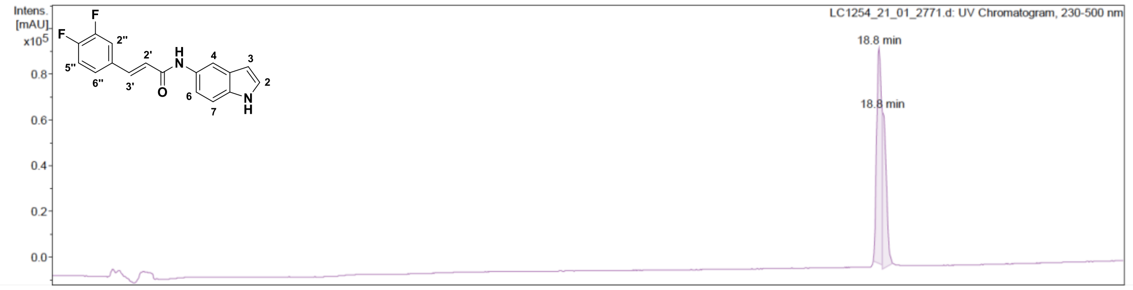

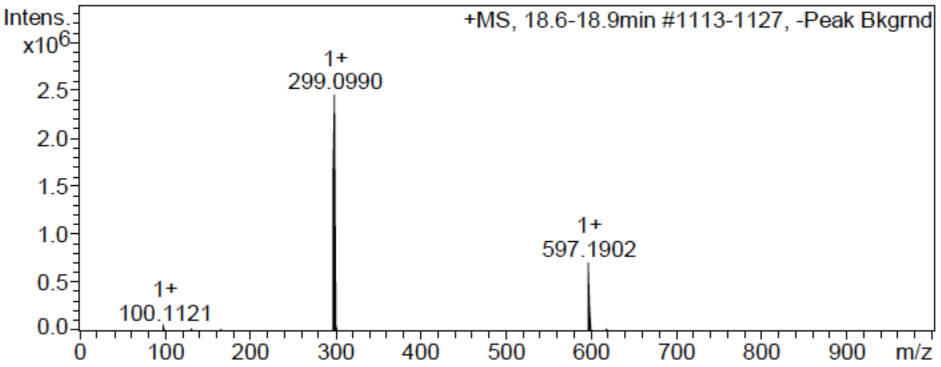

(*E*)-3-(3,5-difluorophenyl)-*N*-(1*H*-indol-5-yl)acrylamide (19)

Equipo MAXIS II

Nombre muestra	LC1201
Nombre registro	\\150.244.121.174\Data\2020\HPLC\11_NOVIEMBRE\LC1201_21_01_2718.d
Metodo	ESI Positive hplc1100_ 40 min_50-3000 t=200 6l-min.m
Comentarios	PD63 en 0.5 ul MeOH (Dil 1:40 con MeOH)

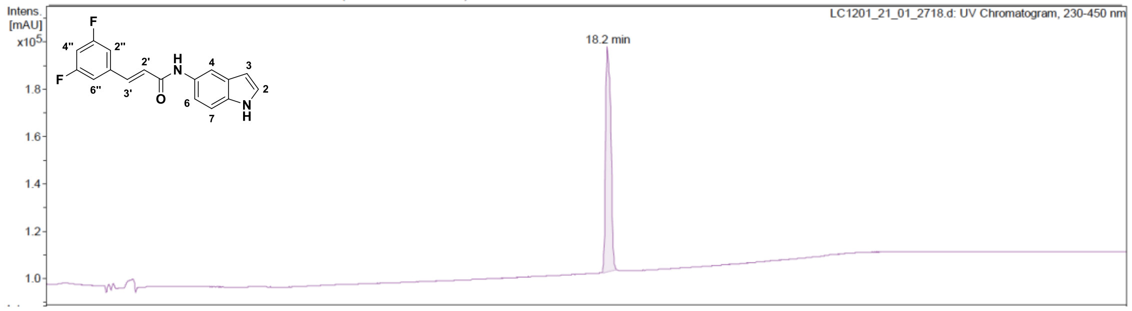

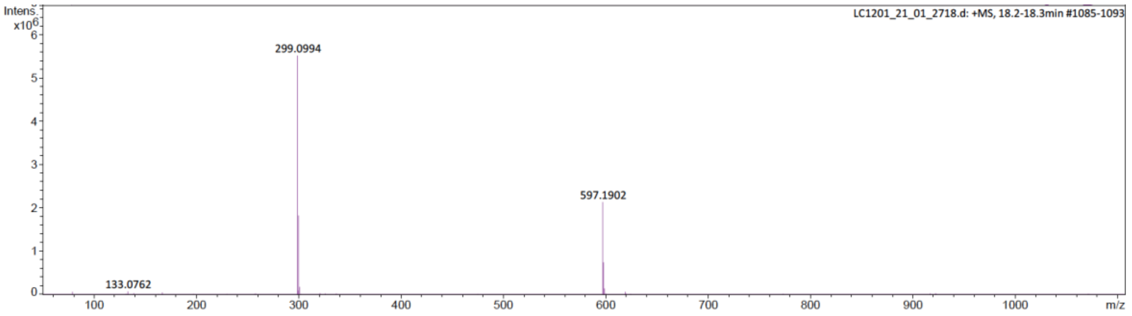

(*E*)-*N*-(1*H*-indol-5-yl)-3-(2-(trifluoromethyl)phenyl)acrylamide (20)

Equipo MAXIS II

Nombre muestra	LC1262
Nombre registro	D:\Data\2020\HPLC\12_DICIEMBRE\LC1262_11_01_2779.d
Metodo	ESI Positive hplc1100_ 40 min_50-3000 t=200 6l-min.m
Comentarios	PD64 (0.5 mg/ml en MeOH. Dil 1:20 con MeOH)

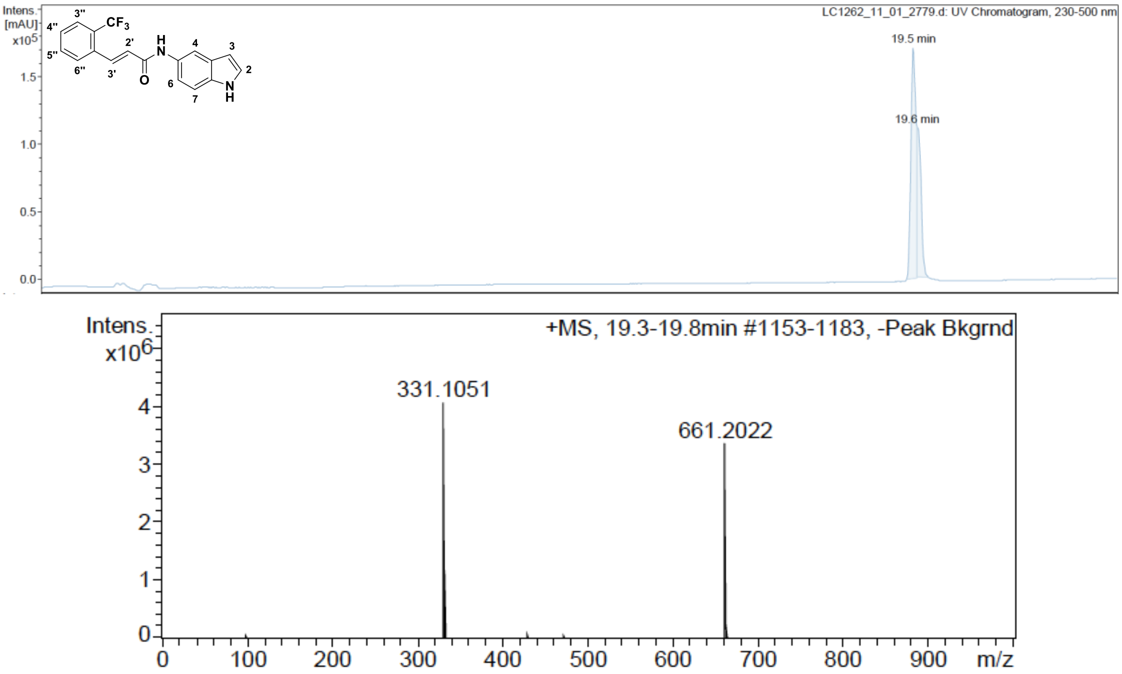

(*E*)-*N*-(1*H*-indol-5-yl)-3-(3-(trifluoromethyl)phenyl)acrylamide (21)

Equipo MAXIS II

Nombre muestra	LC1199
Nombre registro	\\150.244.121.174\Data\2020\HPLC\11_NOVIEMBRE\LC1199_11_01_2716.d
Metodo	ESI Positive hplc1100_ 40 min_50-3000 t=200 6l-min.m
Comentarios	PD65 en 0.5 ul MeOH (Dil 1:40 con MeOH)

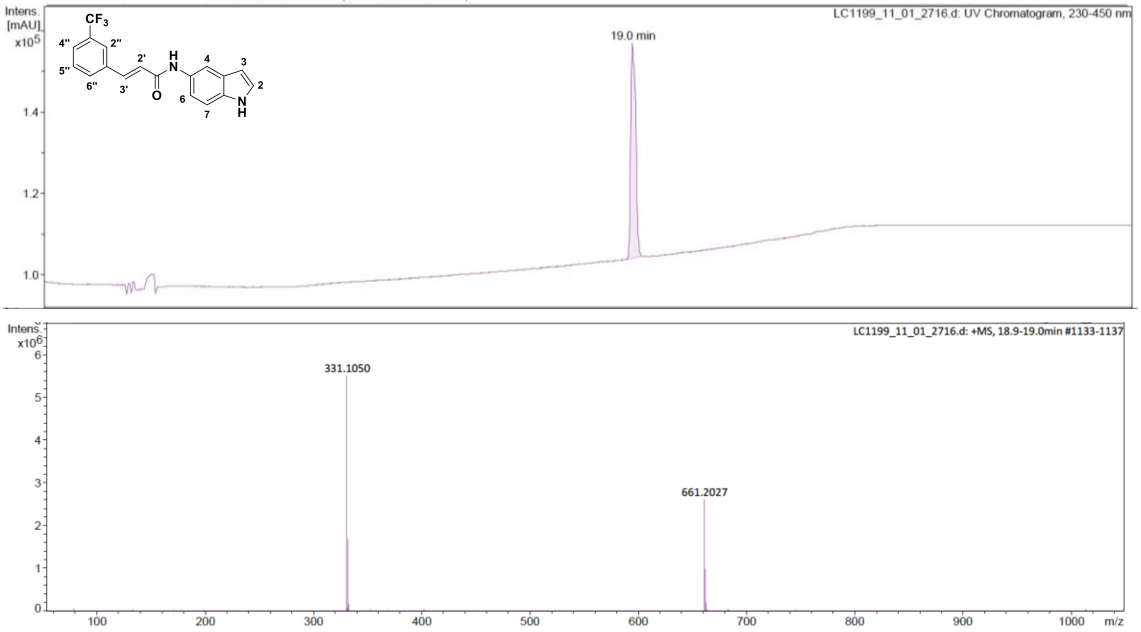

(*E*)-5-(3-(*o*-tolyl)acrylamido)-1*H*-indole-3-carboxamide (27)

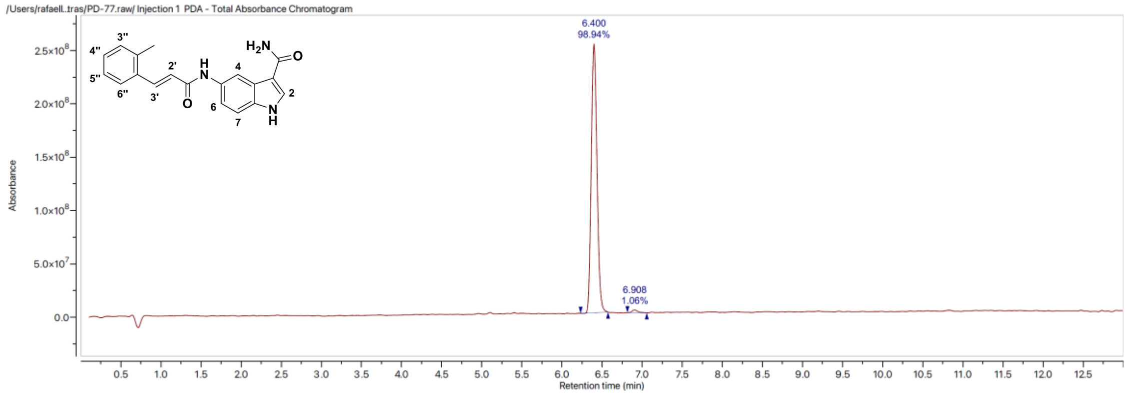

Compound Details

Cpd. 1: C19 H17 N3 O2

Compound ID Table

Name	Formula	Species	RT	RT Diff	Mass	Mass (Tgt)	ID Source	Score	Diff (ppm)	Score (MFG)
	C19 H17 N3 O2	(M+H)+	0.229		319.1317	319.1321	FBF	99.20	-1.33	

Compound Spectra (overlaid)

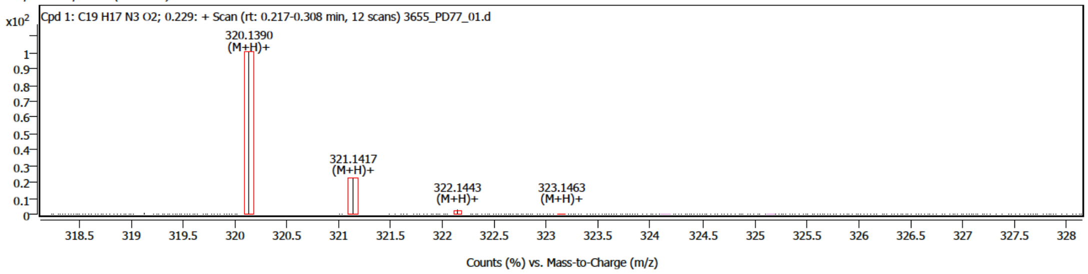

Compound Details

Cpd. 1: C19 H17 N3 O2

Compound ID Table

Name	Formula	Species	RT	RT Diff	Mass	Mass (Tgt)	ID Source	Score	Diff (ppm)	Score (MFG)
	C19 H17 N3 O2	(M+Na)+	0.326		319.1321	319.1321	FBF	99.67	0.04	

Compound Spectra (overlaid)

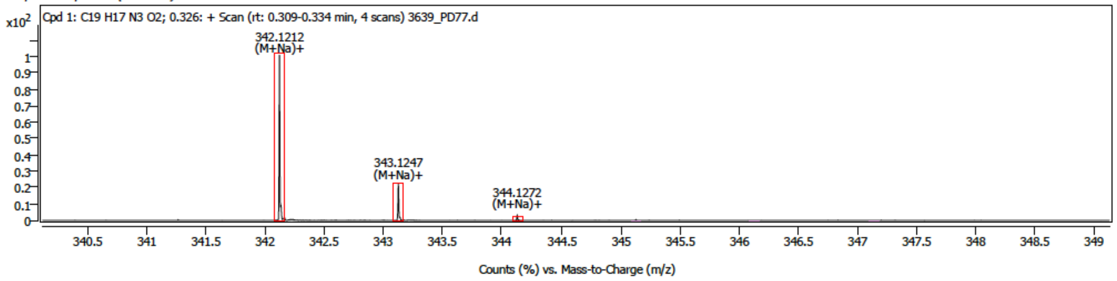

(*E*)-5-(3-(*m*-tolyl)acrylamido)-1*H*-indole-3-carboxamide (28)

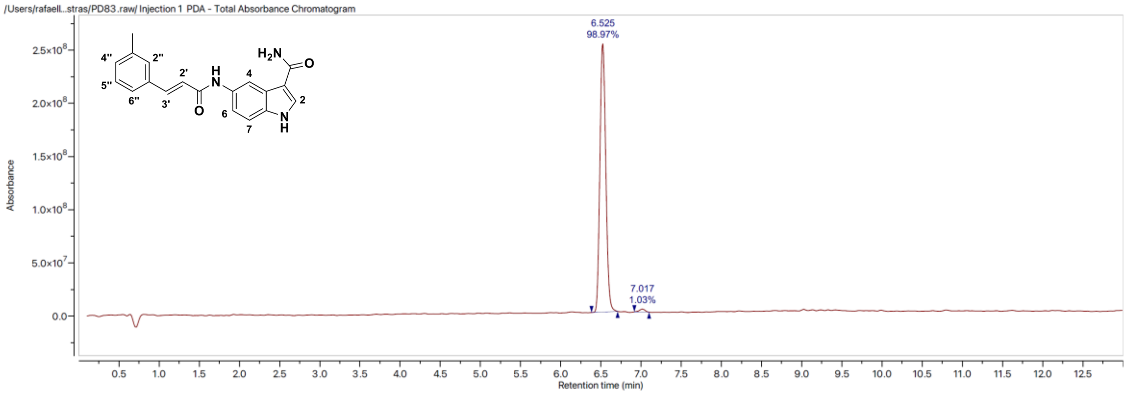

Compound Details

Cpd. 1: C19 H17 N3 O2

Compound ID Table

Name	Formula	Species	RT	RT Diff	Mass	Mass (Tgt)	ID Source	Score	Diff (ppm)	Score (MFG)
	C19 H17 N3 O2	(M+H)+	0.256		319.1313	319.1321	FBF	95.86	-2.45	

Compound Spectra (overlaid)

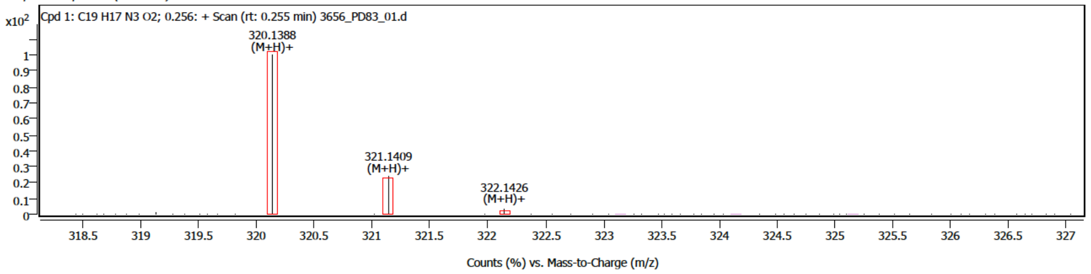

Compound Details

Cpd. 1: C19 H17 N3 O2

Compound ID Table

Name	Formula	Species	RT	RT Diff	Mass	Mass (Tgt)	ID Source	Score	Diff (ppm)	Score (MFG)
	C19 H17 N3 O2	(M+Na)+	0.355		319.1322	319.1321	FBF	99.92	0.30	

Compound Spectra (overlaid)

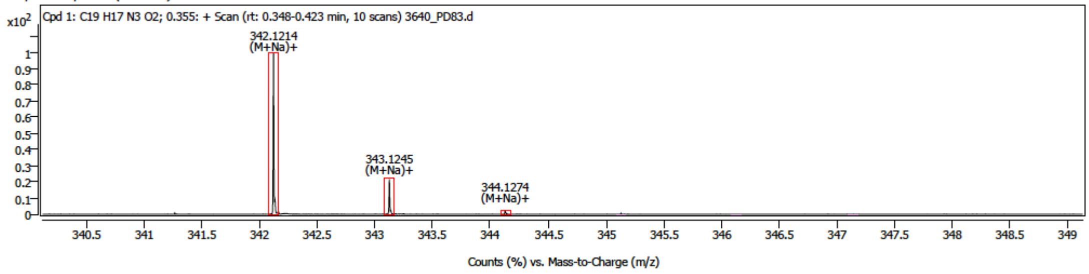

(*E*)-5-(3-(2-chlorophenyl)acrylamido)-1*H*-indole-3-carboxamide (29)

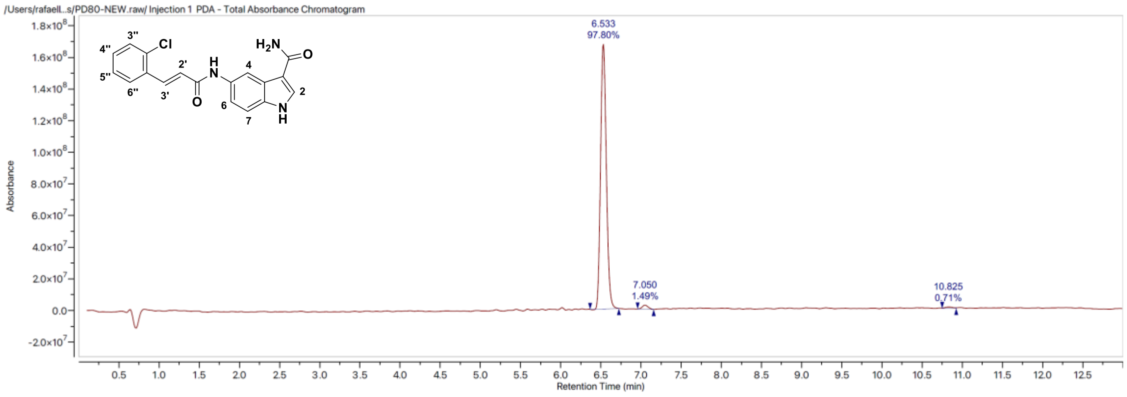

Compound Details

Cpd. 1: C18 H14 Cl N3 O2

Compound ID Table

Name	Formula	Species	RT	RT Diff	Mass	Mass (Tgt)	ID Source	Score	Diff (ppm)	Score (MFG)
	C18 H14 Cl N3 O2	(M+H)+	0.583		339.0775	339.0775	FBF	70.70	0.23	

Compound Spectra (overlaid)

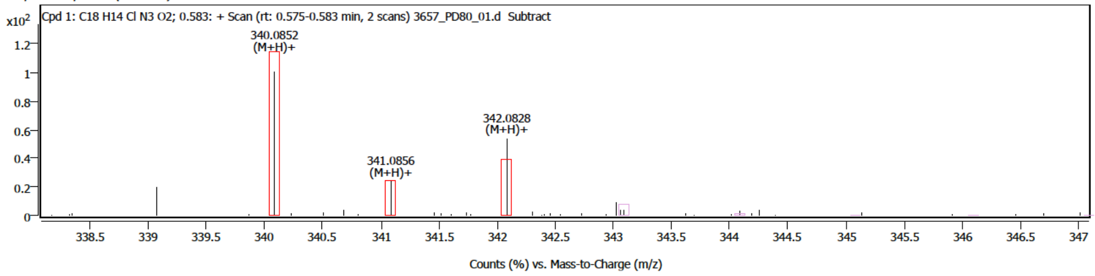

Compound Details

Cpd. 1: C18 H14 Cl N3 O2

Compound ID Table

Name	Formula	Species	RT	RT Diff	Mass	Mass (Tgt)	ID Source	Score	Diff (ppm)	Score (MFG)
	C18 H14 Cl N3 O2	(M+Na)+	0.195		339.0776	339.0775	FBF	99.68	0.36	

Compound Spectra (overlaid)

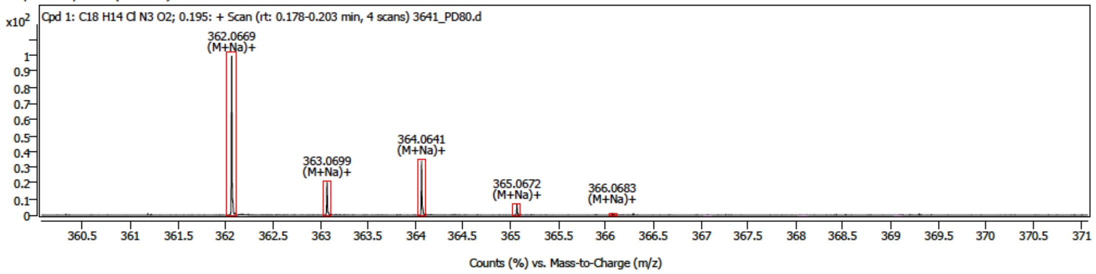

(*E*)-5-(3-(2,6-dichlorophenyl)acrylamido)-1*H*-indole-3-carboxamide (30)

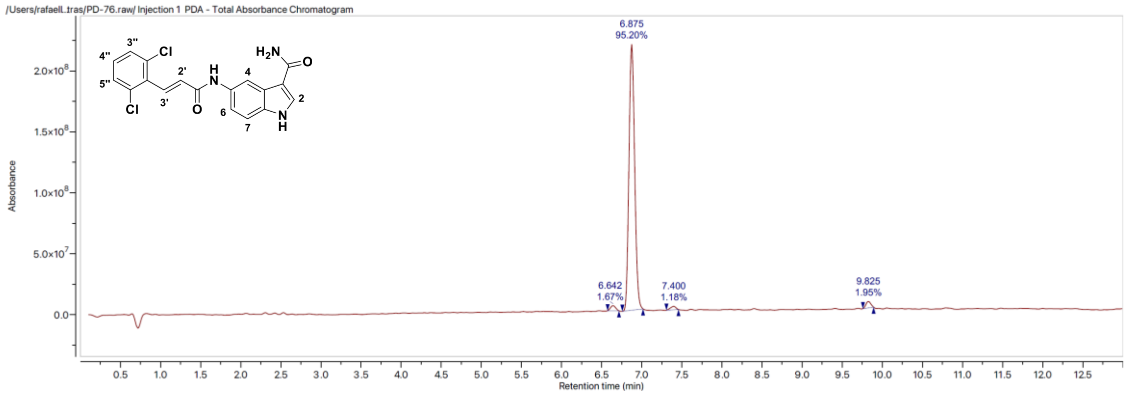

Compound Details

Cpd. 1: C18 H13 Cl2 N3 O2

Compound ID Table

Name	Formula	Species	RT	RT Diff	Mass	Mass (Tgt)	ID Source	Score	Diff (ppm)	Score (MFG)
	C18 H13 Cl2 N3 O2	(M+H)+	0.332		373.0384	373.0385	FBF	94.86	-0.31	

Compound Spectra (overlaid)

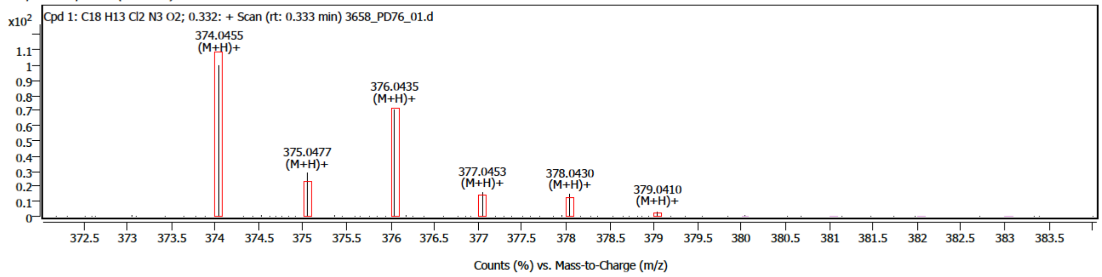

Compound Details

Cpd. 1: C18 H13 Cl2 N3 O2

Compound ID Table

Name	Formula	Species	RT	RT Diff	Mass	Mass (Tgt)	ID Source	Score	Diff (ppm)	Score (MFG)
	C18 H13 Cl2 N3 O2	(M+Na)+	0.202		373.0382	373.0385	FBF	99.47	-0.71	

Compound Spectra (overlaid)

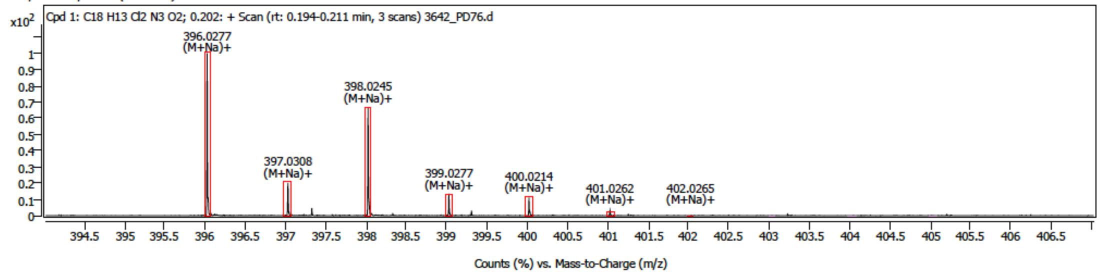

(*E*)-5-(3-(2-fluorophenyl)acrylamido)-1*H*-indole-3-carboxamide (31)

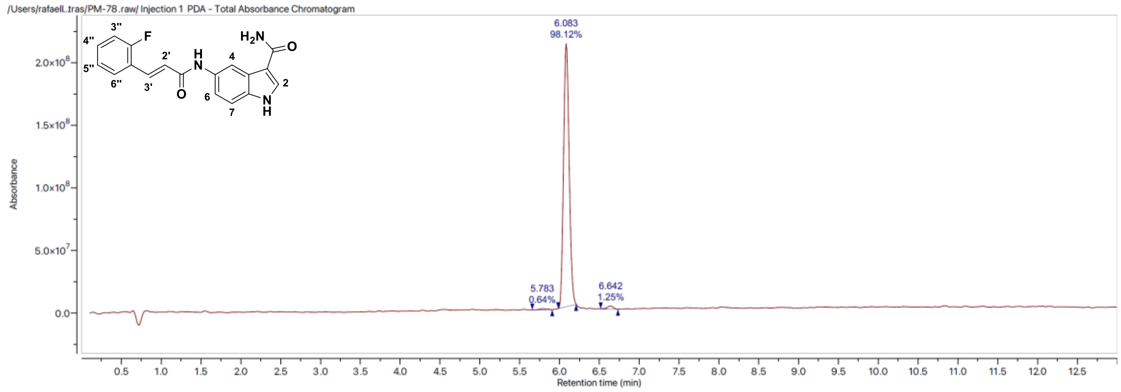

Compound Details

Cpd. 1: C18 H14 F N3 O2

Compound ID Table

Name	Formula	Species	RT	RT Diff	Mass	Mass (Tgt)	ID Source	Score	Diff (ppm)	Score (MFG)
	C18 H14 F N3 O2	(M+H)+	0.373		323.1060	323.1070	FBF	96.89	-2.98	

Compound Spectra (overlaid)

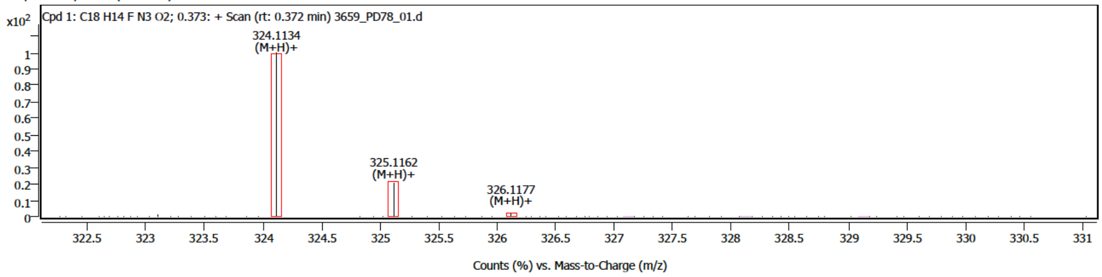

Compound Details

Cpd. 1: C18 H14 F N3 O2

Compound ID Table

Name	Formula	Species	RT	RT Diff	Mass	Mass (Tgt)	ID Source	Score	Diff (ppm)	Score (MFG)
	C18 H14 F N3 O2	(M+Na)+	0.121		323.1071	323.1070	FBF	99.89	0.41	

Compound Spectra (overlaid)

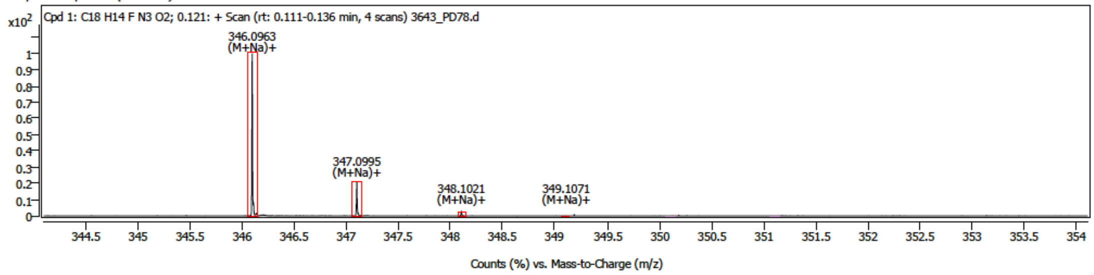

(*E*)-5-(3-(3-fluorophenyl)acrylamido)-1*H*-indole-3-carboxamide (32)

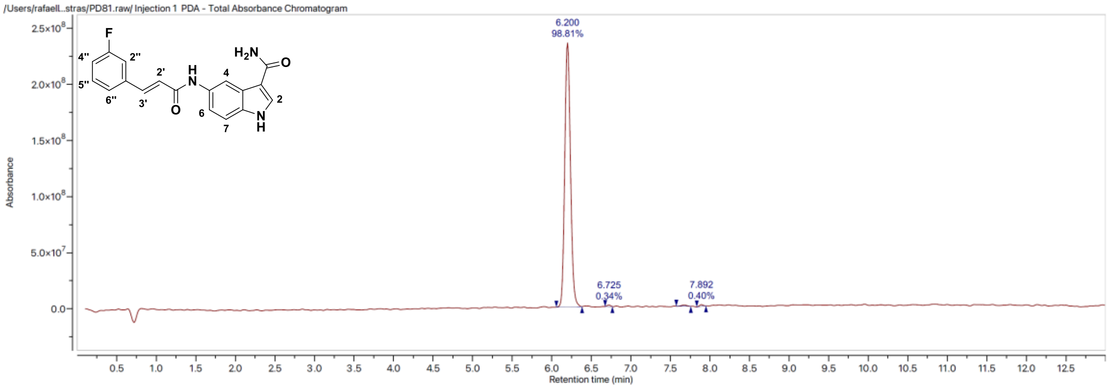

Compound Details

Cpd. 1: C18 H14 F N3 O2

Compound ID Table

Name	Formula	Species	RT	RT Diff	Mass	Mass (Tgt)	ID Source	Score	Diff (ppm)	Score (MFG)
	C18 H14 F N3 O2	(M+H)+	0.315		323.1072	323.1070	FBF	98.47	0.50	

Compound Spectra (overlaid)

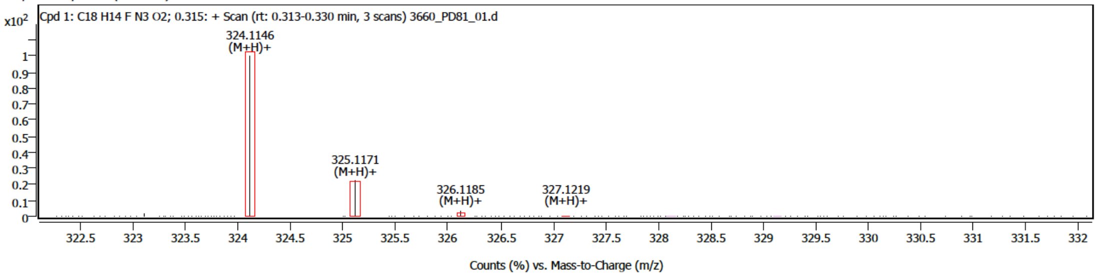

Compound Details

Cpd. 1: C18 H14 F N3 O2

Compound ID Table

Name	Formula	Species	RT	RT Diff	Mass	Mass (Tgt)	ID Source	Score	Diff (ppm)	Score (MFG)
	C18 H14 F N3 O2	(M+Na)+	0.332		323.1071	323.1070	FBF	99.88	0.22	

Compound Spectra (overlaid)

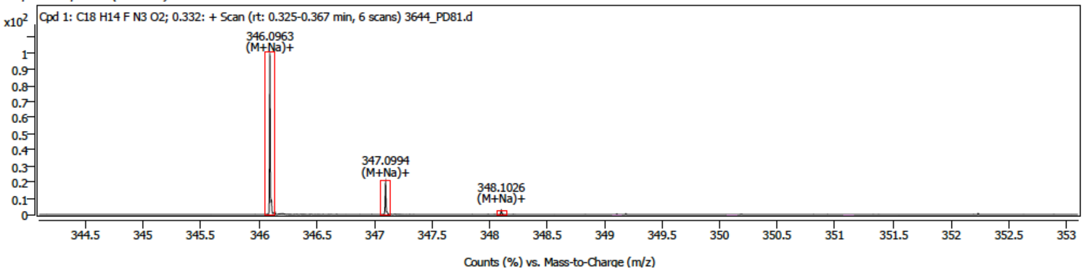

(*E*)-5-(3-(4-fluorophenyl)acrylamido)-1*H*-indole-3-carboxamide (33)

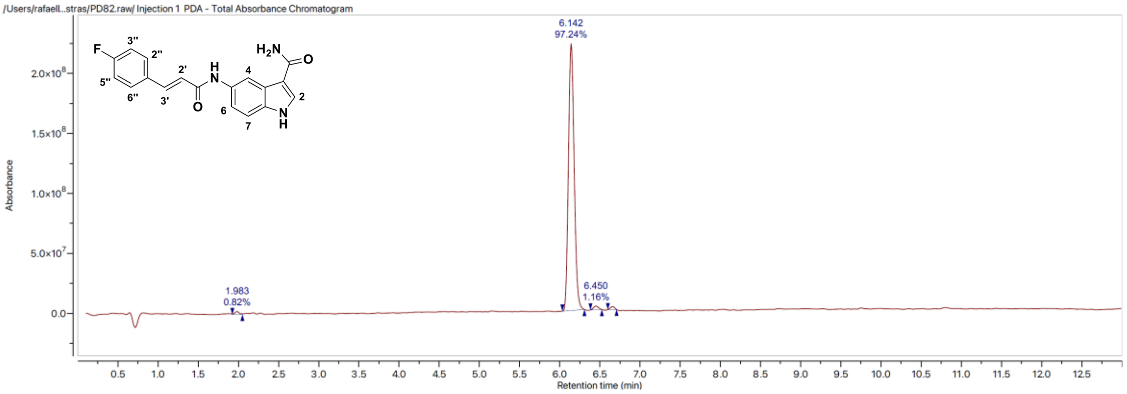

Compound Details

Cpd. 1: C18 H14 F N3 O2

Compound ID Table

Name	Formula	Species	RT	RT Diff	Mass	Mass (Tgt)	ID Source	Score	Diff (ppm)	Score (MFG)
	C18 H14 F N3 O2	(M+H)+	0.175		323.1080	323.1070	FBF	96.51	3.16	

Compound Spectra (overlaid)

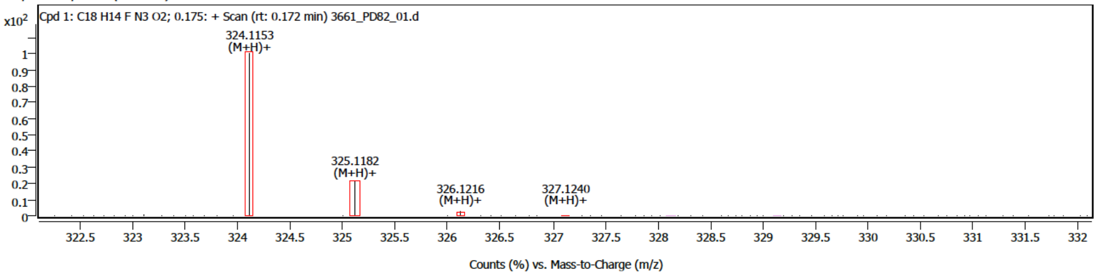

Compound Details

Cpd. 1: C18 H14 F N3 O2

Compound ID Table

Name	Formula	Species	RT	RT Diff	Mass	Mass (Tgt)	ID Source	Score	Diff (ppm)	Score (MFG)
	C18 H14 F N3 O2	(M+Na)+	0.351		323.1071	323.1070	FBF	99.90	0.23	

Compound Spectra (overlaid)

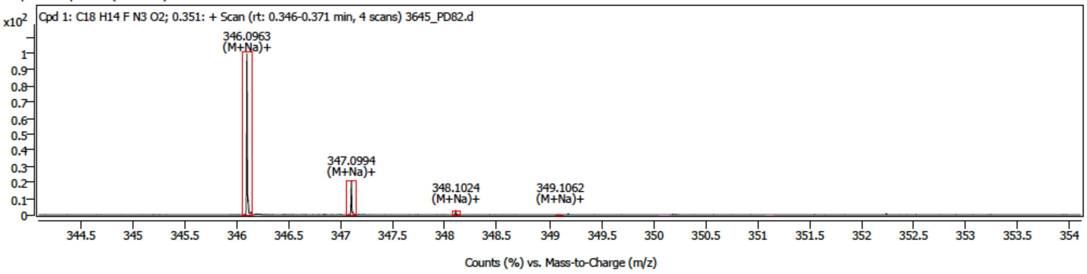

(*E*)-5-(3-(2,3-difluorophenyl)acrylamido)-1*H*-indole-3-carboxamide (34)

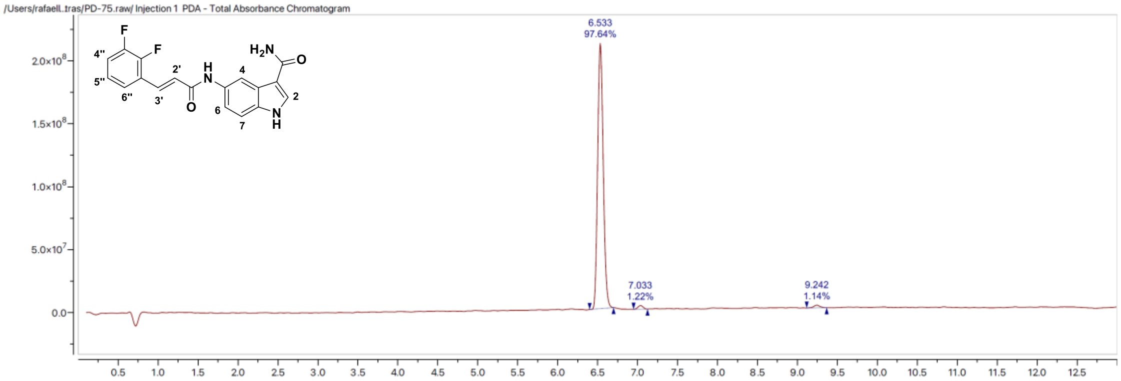

Compound Details

Cpd. 1: C18 H13 F2 N3 O2

Compound ID Table

Name	Formula	Species	RT	RT Diff	Mass	Mass (Tgt)	ID Source	Score	Diff (ppm)	Score (MFG)
	C18 H13 F2 N3 O2	(M+H)+	0.200		341.0969	341.0976	FBF	98.40	-1.94	

Compound Spectra (overlaid)

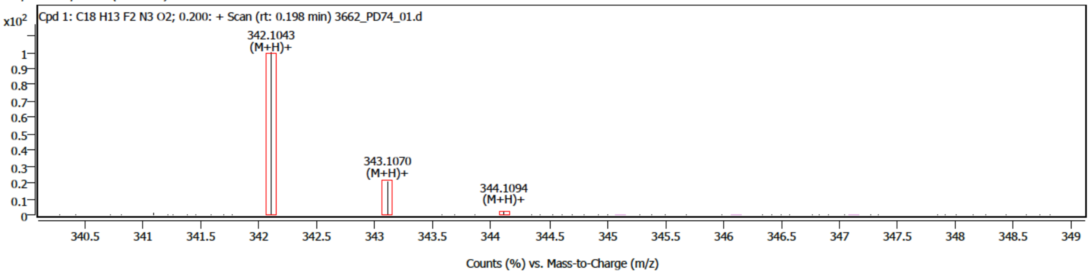

Compound Details

Cpd. 1: C18 H13 F2 N3 O2

Compound ID Table

Name	Formula	Species	RT	RT Diff	Mass	Mass (Tgt)	ID Source	Score	Diff (ppm)	Score (MFG)
	C18 H13 F2 N3 O2	(M+Na)+	0.435		341.0977	341.0976	FBF	99.78	0.27	

Compound Spectra (overlaid)

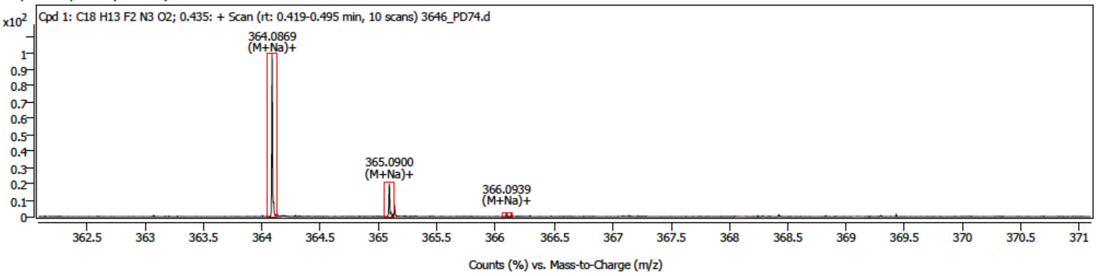

(*E*)-5-(3-(2,4-difluorophenyl)acrylamido)-1*H*-indole-3-carboxamide (35)

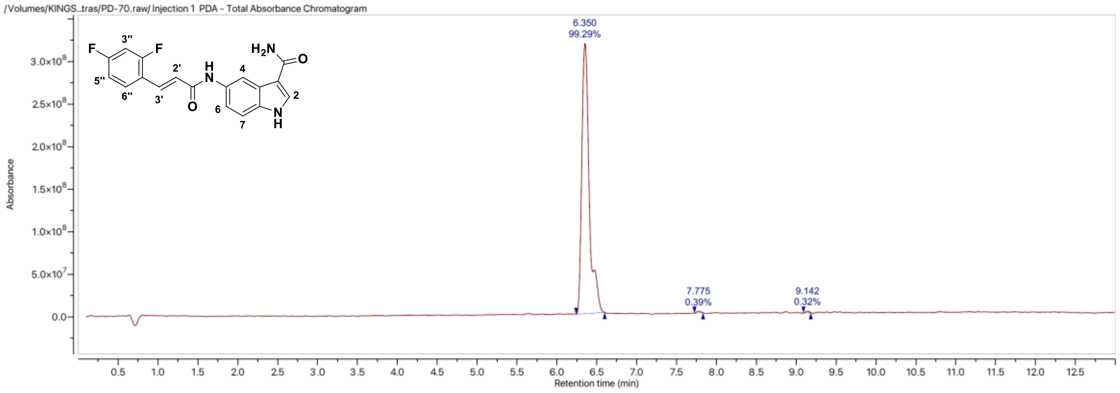

Compound Details

Cpd. 1: C18 H13 F2 N3 O2

Compound ID Table

Name	Formula	Species	RT	RT Diff	Mass	Mass (Tgt)	ID Source	Score	Diff (ppm)	Score (MFG)
	C18 H13 F2 N3 O2	(M+H)+	0.167		341.0972	341.0976	FBF	98.02	-1.18	

Compound Spectra (overlaid)

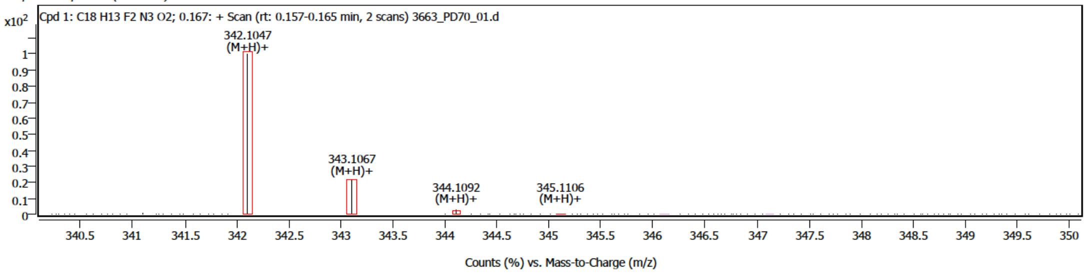

Compound Details

Cpd. 1: C18 H13 F2 N3 O2

Compound ID Table

Name	Formula	Species	RT	RT Diff	Mass	Mass (Tgt)	ID Source	Score	Diff (ppm)	Score (MFG)
	C18 H13 F2 N3 O2	(M+Na)+	0.414		341.0978	341.0976	FBF	99.60	0.49	

Compound Spectra (overlaid)

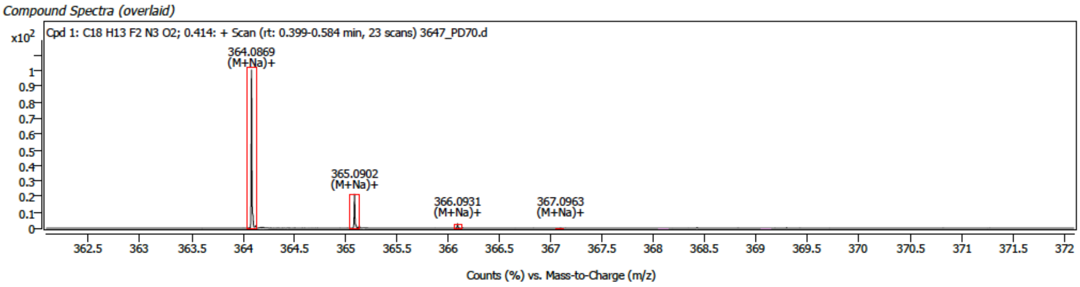

(*E*)-5-(3-(2,5-difluorophenyl)acrylamido)-1*H*-indole-3-carboxamide (36)

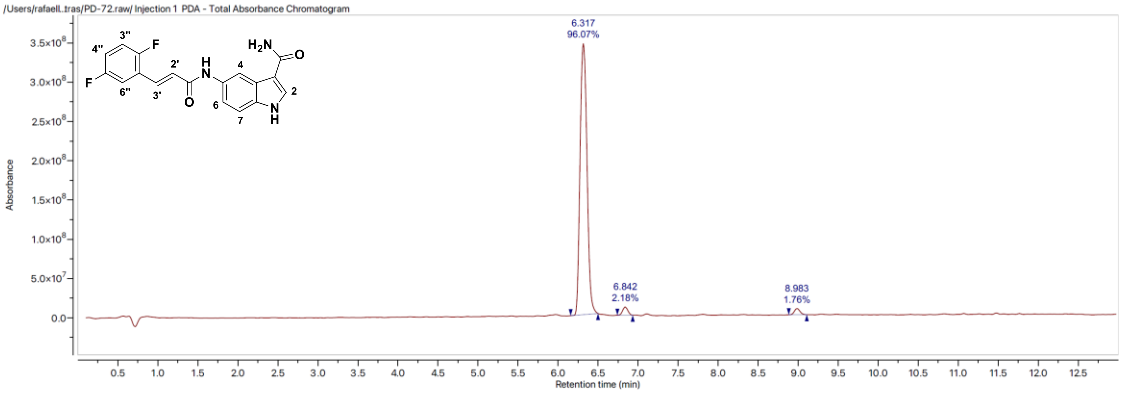

Compound Details

Cpd. 1: C18 H13 F2 N3 O2

Compound ID Table

Name	Formula	Species	RT	RT Diff	Mass	Mass (Tgt)	ID Source	Score	Diff (ppm)	Score (MFG)
	C18 H13 F2 N3 O2	(M+H)+	0.602		341.0969	341.0976	FBF	76.15	-2.02	

Compound Spectra (overlaid)

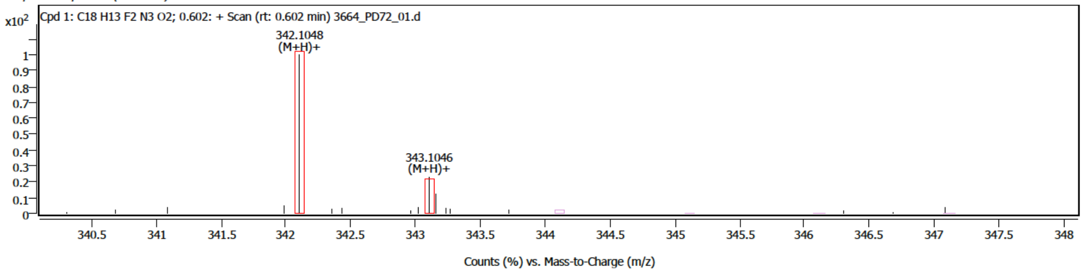

Compound Details

Cpd. 1: C18 H13 F2 N3 O2

Compound ID Table

Name	Formula	Species	RT	RT Diff	Mass	Mass (Tgt)	ID Source	Score	Diff (ppm)	Score (MFG)
	C18 H13 F2 N3 O2	(M+Na)+	0.245		341.0976	341.0976	FBF	99.89	0.07	

Compound Spectra (overlaid)

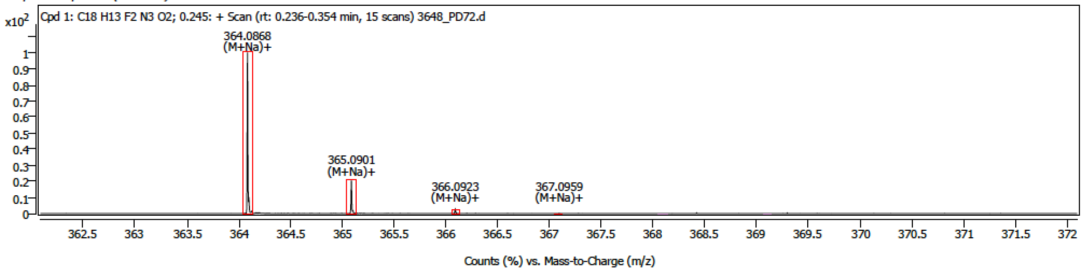

(*E*)-5-(3-(2,6-difluorophenyl)acrylamido)-1*H*-indole-3-carboxamide (37)

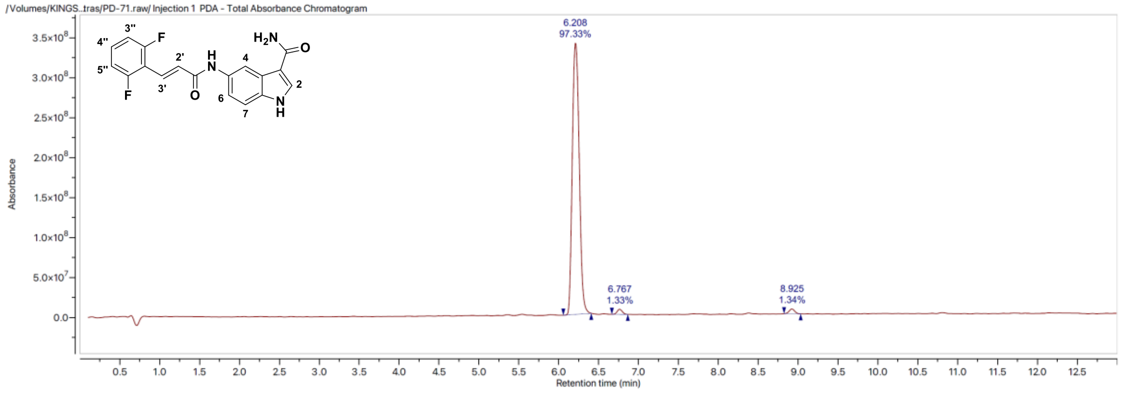

Compound Details

Cpd. 1: C18 H13 F2 N3 O2

Compound ID Table

Name	Formula	Species	RT	RT Diff	Mass	Mass (Tgt)	ID Source	Score	Diff (ppm)	Score (MFG)
	C18 H13 F2 N3 O2	(M+H)+	0.782		341.0964	341.0976	FBF	79.19	-3.34	

Compound Spectra (overlaid)

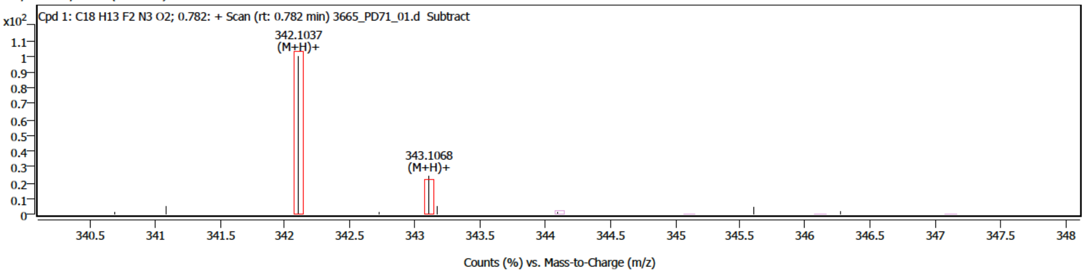

Compound Details

Cpd. 1: C18 H13 F2 N3 O2

Compound ID Table

Name	Formula	Species	RT	RT Diff	Mass	Mass (Tgt)	ID Source	Score	Diff (ppm)	Score (MFG)
	C18 H13 F2 N3 O2	(M+Na)+	0.806		341.0977	341.0976	FBF	99.68	0.34	

Compound Spectra (overlaid)

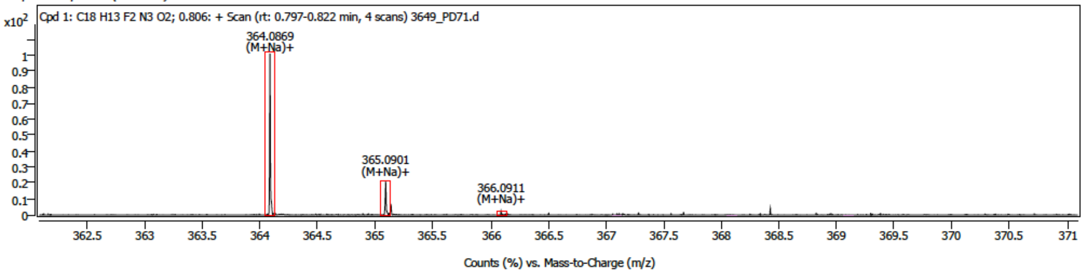

(*E*)-5-(3-(3,4-difluorophenyl)acrylamido)-1*H*-indole-3-carboxamide (38)

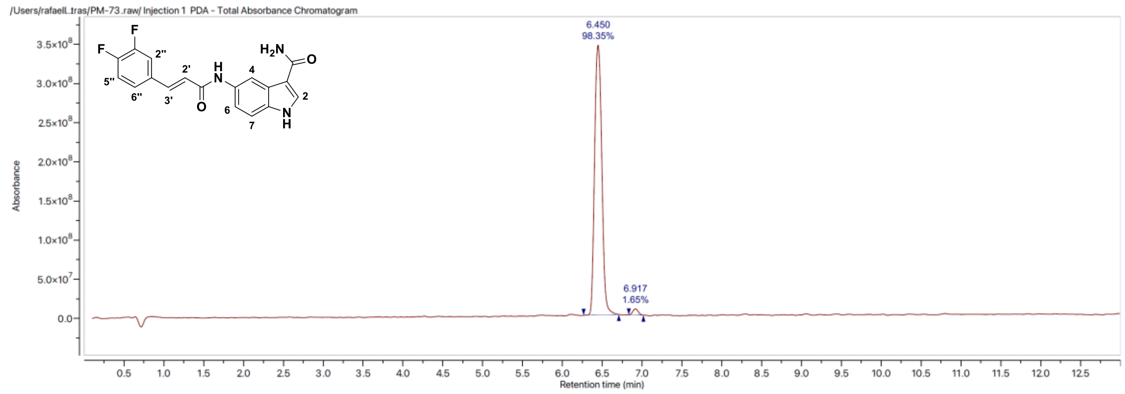

Compound Details

Cpd. 1: C18 H13 F2 N3 O2

Compound ID Table

Name	Formula	Species	RT	RT Diff	Mass	Mass (Tgt)	ID Source	Score	Diff (ppm)	Score (MFG)
	C18 H13 F2 N3 O2	(M+H)+	0.198		341.0972	341.0976	FBF	99.12	-1.02	

Compound Spectra (overlaid)

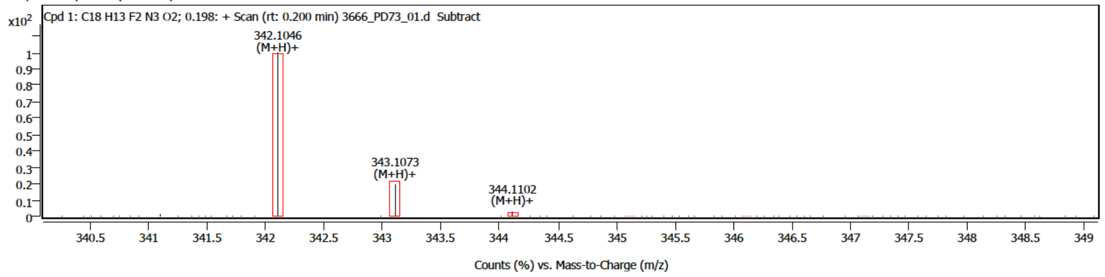

Compound Details

Cpd. 1: C18 H13 F2 N3 O2

Compound ID Table

Name	Formula	Species	RT	RT Diff	Mass	Mass (Tgt)	ID Source	Score	Diff (ppm)	Score (MFG)
	C18 H13 F2 N3 O2	(M+Na)+	0.441		341.0978	341.0976	FBF	99.84	0.61	

Compound Spectra (overlaid)

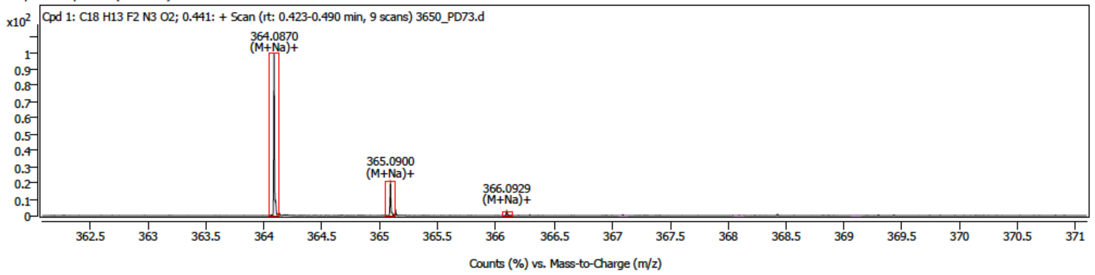

(*E*)-5-(3-(3,5-difluorophenyl)acrylamido)-1*H*-indole-3-carboxamide (39)

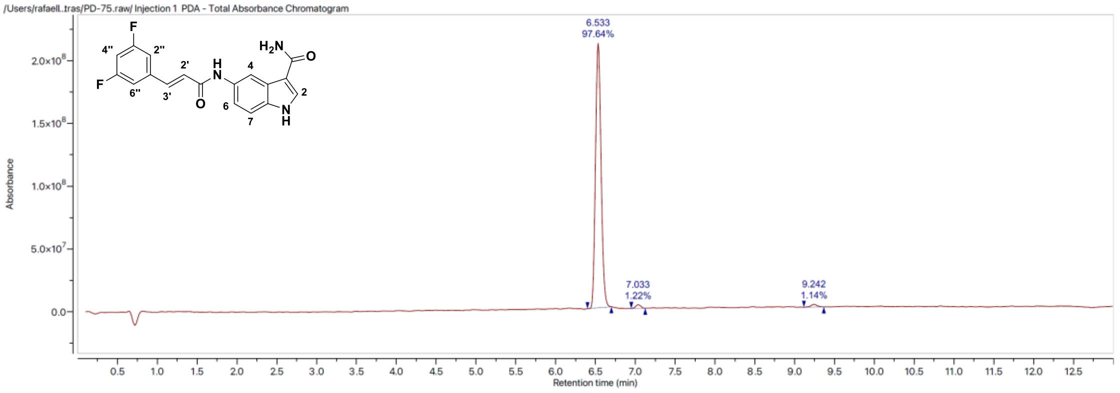

Compound Details

Cpd. 1: C18 H13 F2 N3 O2

Compound ID Table

Name	Formula	Species	RT	RT Diff	Mass	Mass (Tgt)	ID Source	Score	Diff (ppm)	Score (MFG)
	C18 H13 F2 N3 O2	(M+H)+	0.321		341.0973	341.0976	FBF	96.55	-0.97	

Compound Spectra (overlaid)

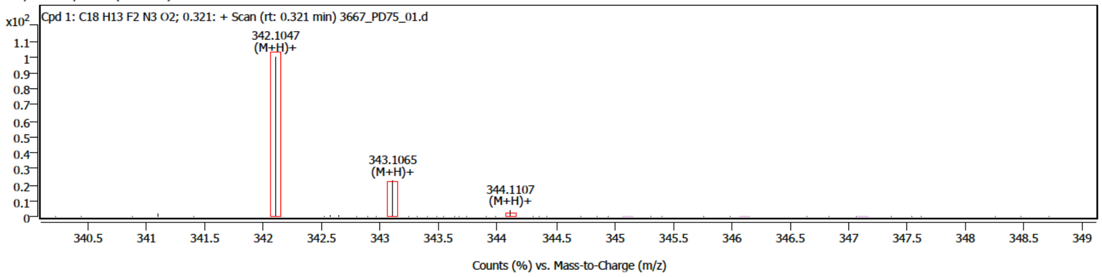

Compound Details

Cpd. 1: C18 H13 F2 N3 O2

Compound ID Table

Name	Formula	Species	RT	RT Diff	Mass	Mass (Tgt)	ID Source	Score	Diff (ppm)	Score (MFG)
	C18 H13 F2 N3 O2	(M+Na)+	0.563		341.0977	341.0976	FBF	99.85	0.23	

Compound Spectra (overlaid)

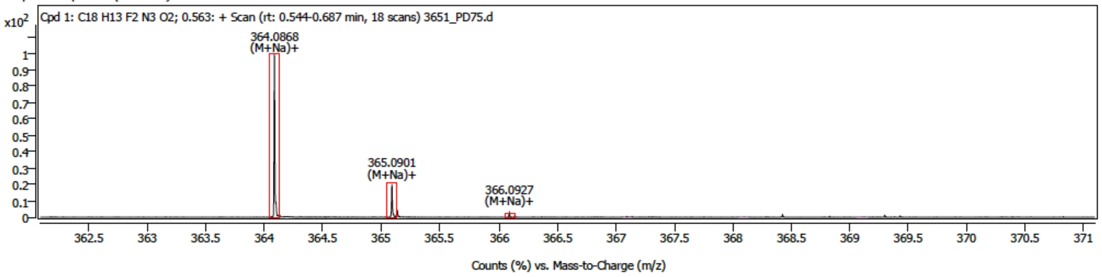

(*E*)-5-(3-(2-(trifluoromethyl)phenyl)acrylamido)-1*H*-indole-3-carboxamide (40)

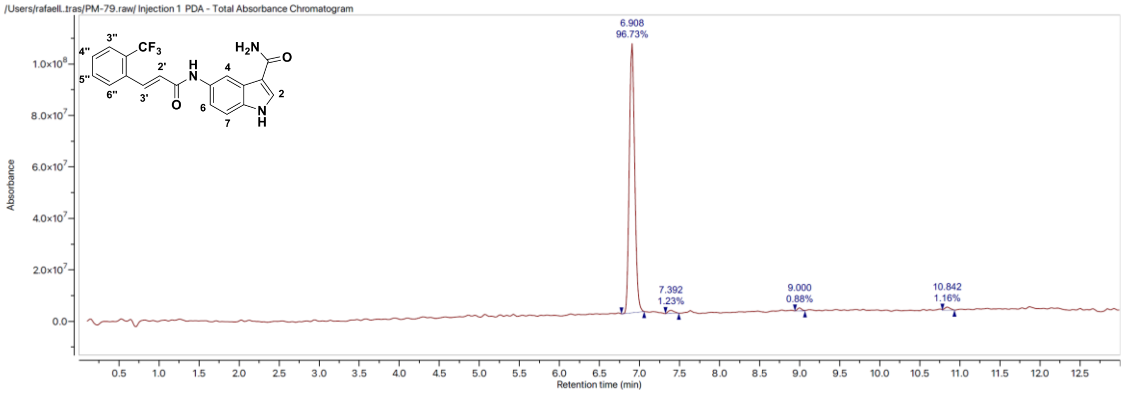

Compound Details

Cpd. 1: C19 H14 F3 N3 O2

Compound ID Table

Name	Formula	Species	RT	RT Diff	Mass	Mass (Tgt)	ID Source	Score	Diff (ppm)	Score (MFG)
	C19 H14 F3 N3 O2	(M+H)+	0.204		373.1032	373.1038	FBF	98.63	-1.74	

Compound Spectra (overlaid)

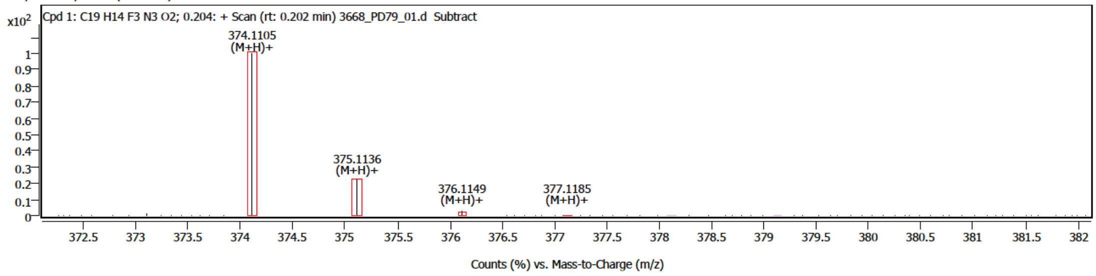

Compound Details

Cpd. 1: C19 H14 F3 N3 O2

Compound ID Table

Name	Formula	Species	RT	RT Diff	Mass	Mass (Tgt)	ID Source	Score	Diff (ppm)	Score (MFG)
	C19 H14 F3 N3 O2	(M+Na)+	0.363		373.1037	373.1038	FBF	99.71	-0.41	

Compound Spectra (overlaid)

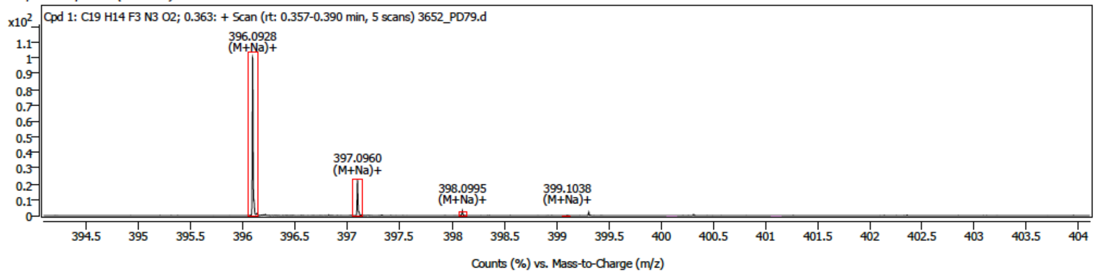

Supplement: Supplementary file 1 — jm4c02659_si_001.pdf [file jm4c02659_si_001.pdf]
